# Synthesis of sterically hindered 4,5-diarylphenanthrenes *via* acid-catalyzed bisannulation of benzenediacetaldehydes with alkynes†
†Electronic supplementary information (ESI) available: Syntheses, NMR, UV-vis-near IR absorption, CV and crystallographic table. CCDC 1865671 (**3c**), 1865672 (**3x**), 1865673 (**7a**). For ESI and crystallographic data in CIF or other electronic format see DOI: 10.1039/c9sc00334g


**DOI:** 10.1039/c9sc00334g

**Published:** 2019-04-17

**Authors:** Yuanming Li, Akiko Yagi, Kenichiro Itami

**Affiliations:** a Institute of Transformative Bio-Molecules (WPI-ITbM) , Nagoya University , Chikusa , Nagoya 464-8602 , Japan . Email: itami@chem.nagoya-u.ac.jp; b Graduate School of Science , Nagoya University , Chikusa , Nagoya 464-8602 , Japan; c JST-ERATO , Itami Molecular Nanocarbon Project , Nagoya University , Chikusa , Nagoya 464-8602 , Japan

## Abstract

The synthesis of sterically hindered phenanthrenes *via* acid-catalyzed bisannulation reaction is described. Treatment of 1,4-benzenediacetaldehyde with terminal aryl alkynes in the presence of B(C_6_F_5_)_3_ provides 4,5-diarylphenanthrenes in good yields with excellent regioselectivity. The use of internal alkyne substrates enabled the synthesis of sterically hindered 3,4,5,6-tetrasubstituted phenanthrenes displaying augmented backbone helicity. Furthermore, 1,5-disubstituted, 1,8-disubstituted, 1,2,5,6-tetrasubstituted, and 1,2,7,8-tetrasubstituted phenanthrenes can be obtained through the reaction of alkynes with 1,3-benzenediacetaldehyde or 1,2-benzenediacetaldehyde disilyl acetal.

## Abstract 2

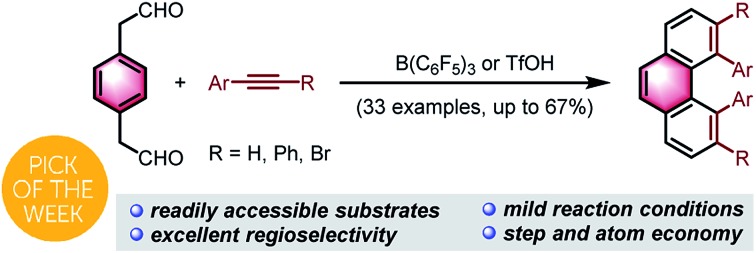
Acid-catalyzed bisannulation of benzenediacetaldehydes with alkynes provided a rapid access to sterically hindered 4,5-diarylphenanthrenes and multisubstituted phenanthrenes regioselectively.

## Introduction

**Fig. 1:**
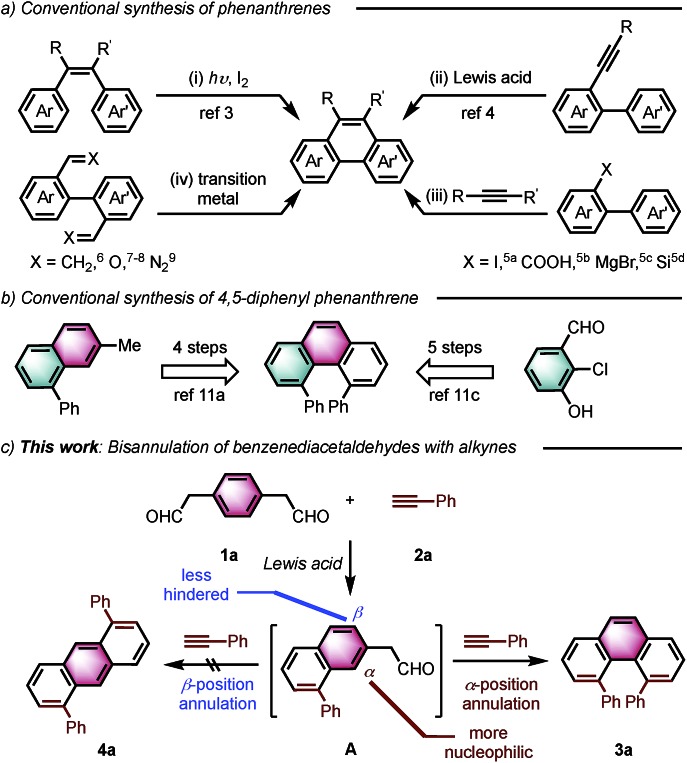
Synthetic strategies leading to phenanthrenes.

Angularly fused polycyclic aromatics (AF-PAHs; *e.g.*, phenacenes and helicenes) are unique structural motifs that are utilized in a wide array of materials ranging from organic electronics^1^ to medicinal chemistry.^2^ Phenanthrene, the simplest AF-PAH, is the distinct fundamental subunit of higher AF-PAHs and the development of synthetic methods for the preparation of functionalized phenanthrenes has received extensive attention over the years (Fig. 1a). The Mallory cyclization of stilbene derivatives (i)^3^ and the annulation of alkynylated biaryls (ii)^4^ are useful methods for the synthesis of phenanthrene derivatives. Additionally, transition metal catalyzed annulations of 2-substituted biphenyl with alkynes have also been reported (iii).^5^ Other methods, such as ring-closing olefin metathesis,^6^ carbonyl-olefin metathesis,^7^ McMurry cyclization,^8^ and carbene dimerization,^9^ have also been used (iv). However, synthetic methods for the construction of sterically hindered 4,5-disubstituted phenanthrenes are particularly limited.^10^ For example, known preparations of 4,5-diphenylphenanthrene require either a four- or five-step synthesis and provide the product in low overall yields (Fig. 1b).^11^ Herein, we report the acid-catalyzed bisannulation reaction of 1,4-benzenediacetaldehyde with aryl alkynes that can access sterically hindered, twisted 4,5-disubstituted and 3,4,5,6-tetrasubstituted phenanthrenes. Furthermore, reactions of isomeric 1,3-benzenediacetaldehyde and 1,2-benzenediacetaldehyde disilyl acetal with aryl alkynes gave 1,5-disubstituted, 1,8-disubstituted, 1,2,5,6-tetrasubstituted and 1,2,7,8-tetrasubstituted phenanthrenes, respectively.

Inspired by the synthesis of naphthalene derivatives through the annulation of phenylacetaldehydes with alkynes,^12^ we envisioned that the acid-catalyzed reaction of 1,4-benzenediacetaldehyde (**1a**)^13^ with phenylacetylene (**2a**) could provide the intermediate naphthalene **A** (Fig. 1c).

We initially anticipated that the second annulation with another equivalent of alkyne could take place at the less hindered β-position of **A** to give 1,5-diphenylanthracene (**4a**). Surprisingly, however, we observed instead an unprecedented α-position annulation to give isomeric 4,5-diphenylphenanthrene (**3a**), which is an even more interesting, otherwise difficult-to-access AF-PAH.

## Results and discussion

Thus, we investigated the effect of acids and other reaction parameters in the reaction of **1a** and 1-ethyl-4-ethynylbenzene (**2b**) as a model system (Table 1). Though the use of Brønsted acid HNTf_2_,12*g* Lewis acids CuCl_2_/AgSbF_6_ (ref. 12*c*) or TiCl_4_ (ref. 12*b*) failed to give more than trace amounts of target products (entries 1–3), FeCl_3_ (ref. 12*d*) and GaCl_3_ (ref. 12*a*) were able to achieve the phenanthrene transformation in 16% and 35% yield, respectively (entries 4 and 5). A catalytic amount of BF_3_·Et_2_O12*f* led to the formation of **3b** in 41% yield at 80 °C (entry 6). Subsequently, B(C_6_F_5_)_3_ was found to give a higher yield (45%) under milder conditions (entry 7). The best result was achieved upon reacting **1a** (1.0 equiv.) with **2b** (2.5 equiv.) in the presence of 20 mol% B(C_6_F_5_)_3_ and MS4Å in dichloromethane (DCM) at room temperature for 15 h, giving **3b** in 65% isolated yield with excellent regioselectivity (entry 8). Notably, the anthracene product **4b** was not detected under any of the conditions tested. However, the insoluble material formed during the reaction was isolated, and we presume that the self-polymerization of **1a** is the major side reaction (see ESI†). The use of MS4Å was necessary to suppress the hydration of the alkyne. A solvent screening revealed that the initially tested DCM was the optimal medium for this reaction (entries 9–12).

**Table 1:** The reaction optimization^*a*^

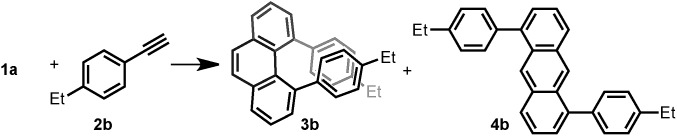						
Entry	Additive (equiv.)	Temp (°C)	Time (h)	Solvent	**3b** ^*d*^ (%)	**4b** ^*d*^ (%)
1	HNTf_2_ (0.15)	rt	15	DCE	n.d.	n.d.
2	CuCl_2_/AgSbF_6_^*b*^	55	22	DCM	n.d.	n.d.
3	TiCl_4_ (2.0)	rt	3	DCM	Trace	n.d.
4	FeCl_3_ (0.10)	80	15	DCE	16	n.d.
5	GaCl_3_ (0.20)	40	6	DCM	35	n.d.
6	BF_3_·Et_2_O (0.05)	80	15	DCE	41	n.d.
7	B(C_6_F_5_)_3_ (0.20)	rt	15	DCM	45	n.d.
8^*c*^	B(C_6_F_5_)_3_ (0.20)	rt	15	DCM	67	n.d.
9^*c*^	B(C_6_F_5_)_3_ (0.20)	rt	15	DCE	59	n.d.
10^*c*^	B(C_6_F_5_)_3_ (0.20)	rt	15	toluene	48	n.d.
11^*c*^	B(C_6_F_5_)_3_ (0.20)	rt	15	CHCl_3_	7	n.d.
12^*c*^	B(C_6_F_5_)_3_ (0.20)	rt	15	THF	Trace	n.d.

^*a*^Reaction conditions: **1a** (0.10 mmol) and **2b** (0.25 mmol) in solvent (2.5 mL).

^*b*^CuCl_2_ (0.08)/AgSbF_6_ (0.16).

^*c*^100 mg MS4Å (1/16) were added.

^*d*^Yield was determined by ^1^H NMR spectroscopy. n.d. = not detected. rt = room temperature.

**Scheme 1:**
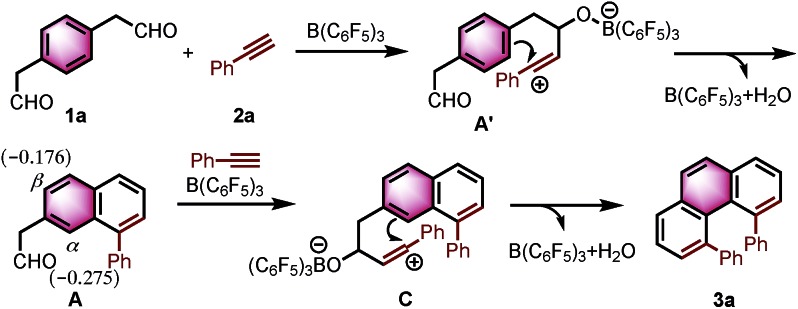
A possible reaction mechanism.

Based on the reported reaction mechanism of phenylacetaldehydes with alkynes,^12^ we suggest the following mechanism (Scheme 1, for details, see ESI†). Initially, B(C_6_F_5_)_3_ coordinates with the carbonyl oxygen,^14^ which would trigger the nucleophilic attack of the alkyne partner to give the vinyl carbocation **A′**. Subsequently, intermediate **A′** undergoes an intramolecular electrophilic aromatic substitution, followed by aromatization to provide the naphthalene **A**. A DFT calculation revealed that the Mulliken atomic charge at α-position is actually more negative than β-position in the naphthalene **A** (–0.275 compared to –0.176, see ESI† for details). The electronic bias of the C–C bond formation is overcoming the steric hindrance between the two phenyl rings in C, providing α-position annulation product **3a** exclusively.

**Scheme 2:**
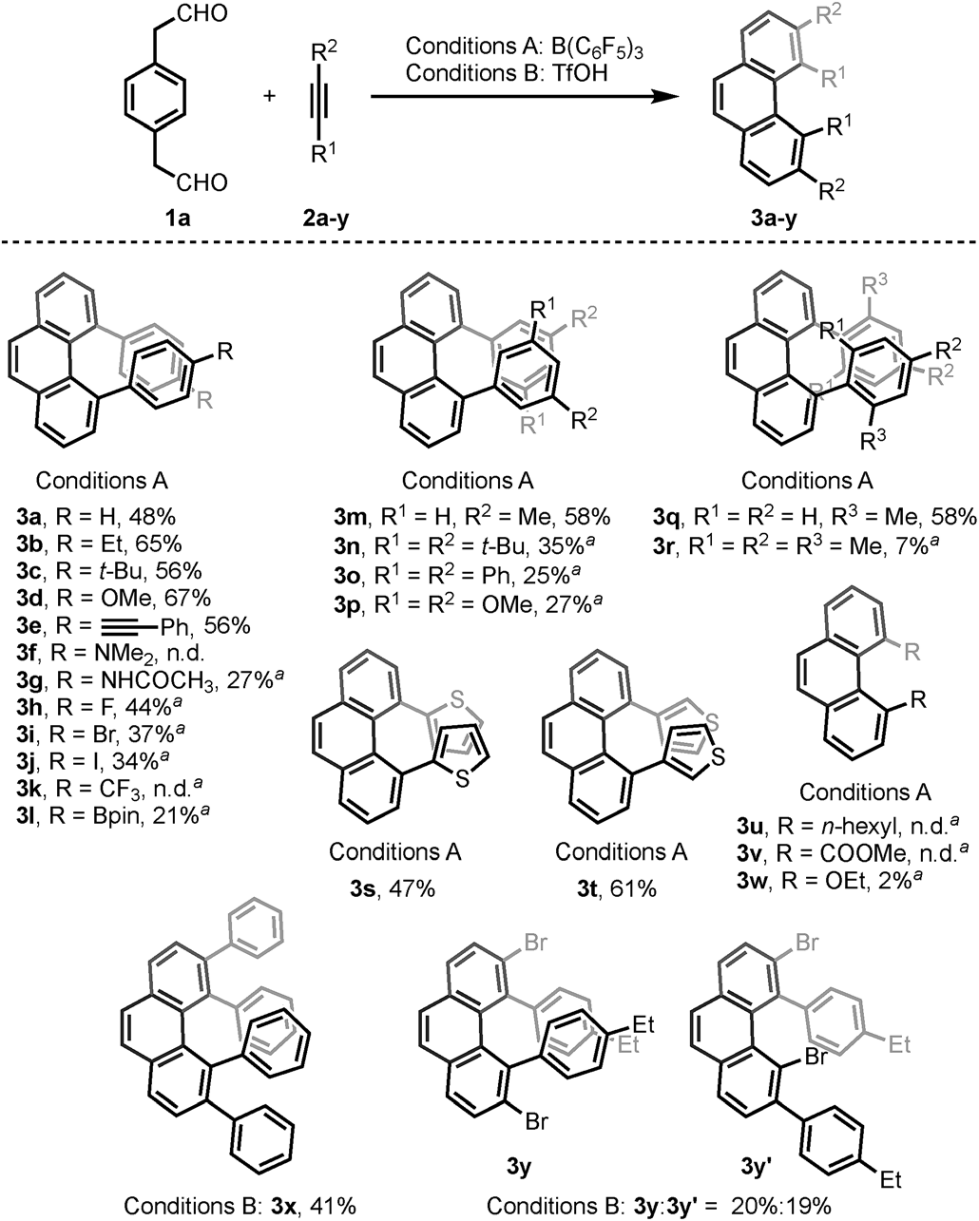
The reaction of **1a** with alkynes. Reaction conditions: **1a** (0.10 mmol), **2** (0.25 mmol). Conditions A: 20 mol% B(C_6_F_5_)_3_/MS4Å, DCM, rt, 15 h. Conditions B: 150 mol% TfOH, CF_3_Ph/HFIP = 10 : 1, –30 °C, 15 h. Yields given correspond to isolated yields. Note: ^a^Solvent : DCM/HFIP = 10 : 1.

With the optimized conditions in hand, we investigated the substrate scope with respect to the alkyne coupling partner (Scheme 2). Installing electron-donating groups on the *para*-position of the phenylacetylene aromatic ring did not significantly alter the reaction outcome (**3a–d**). Interestingly, the reaction of *p*-ethynyltoluene (containing both terminal and disubstituted alkyne moieties) with **1a** selectively gave **3e** in 56% yield. With the dimethylamino substituent, the desired product **3f** was not formed. This may be due to the formation of N/B frustrated Lewis pairs.^15^ However, acetamide substituent was tolerated and target product **3g** was obtained in 27% yield. Under the optimized reaction conditions, the reaction of **1a** with alkyne bearing electron-withdrawing groups at the *para*-position gave the target products **3h–j** in low yield. Further optimization revealed that the yields of **3h–j** increased to 44%, 37%, and 34%, respectively, in the presence of 0.25 mL hexafluoroisopropanol (HFIP). This is likely because of the effect of HFIP on the stabilization of vinyl cations (see the proposed mechanism).^16^ An aryl alkyne bearing a strong electron-withdrawing trifluoromethyl group in the *para*-position did not afford the desired product **3k**. The *para*-boronic acid pinacol (Bpin) ester-substituted aryl alkyne provided the product **3l** in 21% yield. The *meta*-substituents of the aryl acetylene moiety were also tolerated to give the corresponding products **3m–p**. To our delight, the *ortho*-methyl-substituted aryl alkyne afforded the product **3q** in good yield as well. Due to the higher rotational barrier,^17^ product **3q** exists in solution as a mixture of multiple rotamers as confirmed by variable temperature ^1^H NMR and 1D NOE experiments.^18^ Even the more hindered substrate (2,4,6-trimethylphenyl)acetylene underwent bisannulation to give the target product **3r** in 7% yield. Moreover, the reactions of **1a** with heteroaromatic alkynes such as 2-ethynylthiophene and 3-ethynylthiophene gave the products **3s** and **3t** in 47% and 61%, respectively. Unfortunately, aliphatic alkynes (1-octyne) and methyl acetylenecarboxylate were not tolerated under the reaction conditions and the corresponding products **3u** and **3v** were not detected, which emphasized the effect of the aromatic moiety of the phenylacetylenes on the stabilization of vinyl cations (see the proposed mechanism). However, the reaction of **1a** with ethoxyacetylene provided a trace amount of 4,5-diethoxyphenanthrene (**3w**), and the mono-annulation product 2-(8-ethoxynaphthalen-2-yl)acetaldehyde was obtained in 8% yield. To show practicality, we ran a 2.0 mmol large-scale experiment with **2b** to give **3b** in 55% isolated yield (425 mg prepared). To our delight, the 2.0 mmol large-scale reaction of **2l** with **1a** gave the target product **3l** at comparable yield (25%) using 2.0 equivalent BF_3_·Et_2_O instead of 20 mol% B(C_6_F_5_)_3_ (for details, see ESI†).

The scope of the bisannulation reaction with internal alkyne substrates such as diphenylacetylene is also shown in Scheme 2. Further optimization revealed that this transformation is better conducted in the presence of 150 mol% triflic acid (TfOH) using CF_3_Ph and HFIP as solvent at –30 °C for 15 h. Sterically hindered 3,4,5,6-tetraphenylphenanthrene (**3x**) was thus readily synthesized in 41% yield. This protocol could be extended to the use of bromide-substituted phenylacetylene to give a 1.1 : 1.0 regioisomeric mixture of **3y** and **3y′** (for details, see ESI†).

**Fig. 2:**
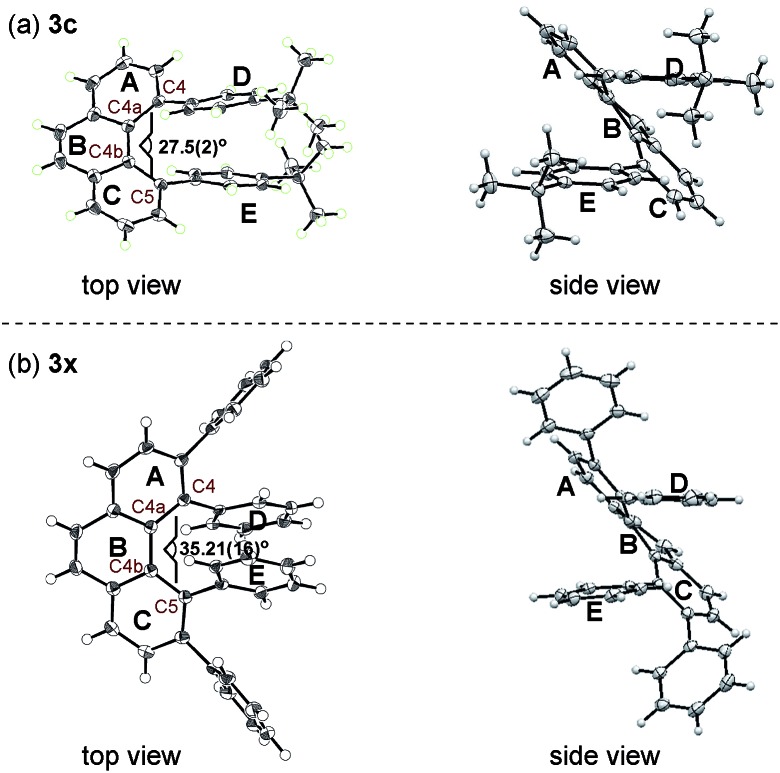
X-ray structure of (a) **3c** and (b) **3x** at 50% thermal probability.

The structures of **3c** and **3x** were unambiguously determined by X-ray crystallographic analysis (Fig. 2). The torsion angle of the phenanthrene moiety of **3c** was 27.5(2)° (measured through the C4–C4a–C4b–C5 torsion). The two *para-tert*-butyl substituted phenyl rings (ring D and E) are oriented roughly parallel to each other (see Fig. 2a, side view, within 13.2°). On the other hand, the increased steric demands caused by the 3,4,5,6-tetraphenyl substitution in **3x** led to greater twisting of the phenanthrene backbone with a C4–C4a–C4b–C5 torsion angle of 35.21(16)°. In addition, the two phenyl rings (ring D and E) are oriented roughly parallel to each other as well (see Fig. 2b, side view, within 11.1°). These nonplanar features of overcrowded phenanthrenes should be beneficial in some of device-oriented applications, such as organic light emitting diodes, where the strong π–π intermolecular interactions cause detrimental effects.

**Scheme 3:**
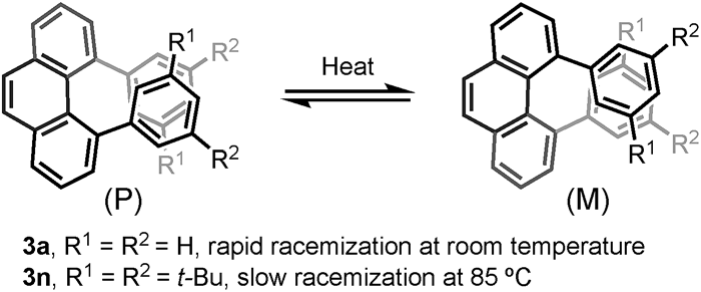
The racemization of 4,5-diarylphenanthrenes.

Furthermore, the chiral resolution of **3b**, **3n**, and **3x** were achieved by HPLC at 25 °C. The racemization of **3b** and **3x** was observed at elevated temperature (see ESI†). The configurational stability of 4,5-diarylphenanthrene with *ortho*- or *meta*-substituents in the aryl moiety (such as **3m–r**) are comparatively higher than **3a** (Scheme 3).^17^ A kinetic study of the thermal racemization of **3n** was performed, corresponding to a racemization barrier of 126 kJ mol^–1^ at 100 °C. Using the solution of highly enantioenriched **3n** in 1,2-dichloroethane, only a small drop in e.r. was observed after heating at 85 °C for 3 h (from 96.8 : 3.2 to 94.0 : 6.0). These results (for details, see ESI†) demonstrate that the configurational stability of these helically chiral compounds can in fact be tuned by judicious substituent choice – an important advantage with respect towards their potential use as chiral materials. The preliminary investigation of the direct asymmetric bisannulation of 1,4-benzenediacetaldehyde with alkynes were conducted as well. However, the use of BINOL-phosphoric acid and dl-10-camphorsulfonic acid failed to give the target products (for details, see ESI†).

**Scheme 4:**
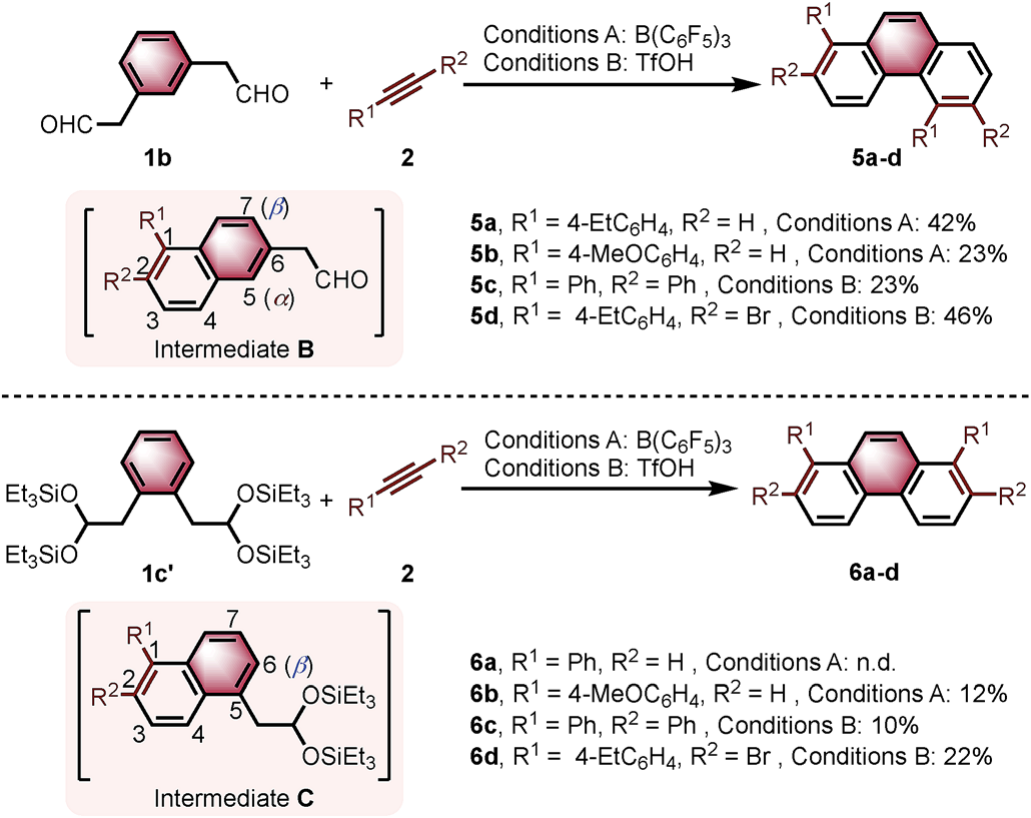
The reaction of **1b** and **1c′** with alkynes. Reaction conditions: **1b** or **1c′** (0.10 mmol), **2** (0.25 mmol). Conditions A: 20 mol% B(C_6_F_5_)_3_/MS4Å, DCM/HFIP = 10 : 1, rt, 15 h. Conditions B: 150 mol% TfOH, CF_3_Ph/HFIP = 10 : 1, –30 °C, 15 h. Yields given correspond to isolated yields. n.d. = not detected.

Interestingly, we observed a different outcome when using other benzenediacetaldehyde positional isomers (*e.g.*, 1,3-benzenediacetaldehyde (**1b**)) as substrates in the bisannulation reaction (Scheme 4). Under the optimized reaction conditions, the reaction of **1b** with terminal alkynes provided unsymmetrical 1,5-disubstituted phenanthrenes **5a** and **5b** with excellent regioselectivity.^19^ The reaction with diphenylacetylene still occurred with excellent regioselectivity to give 1,2,5,6-tetraphenylphenanthrene (**5c**) in 23% yield. The corresponding 2,6-dibromo-1,5-bis(4-ethylphenyl)phenanthrene (**5d**) was obtained in 46% yield with excellent regioselectivity. Noteworthy, **5d** could serve as a valuable building block in cross-coupling reactions to prepare multisubstituted phenanthrenes. The observed outcome is consistent with the reaction going through an intermediate 1,2,6-trisubstituted naphthalene (Scheme 4, Intermediate **B**), where the α-position (5 position) again presents a more favorable pathway for the second annulation than the β-position (7 position).

We further continued our studies by investigating the scope with 1,2-benzenediacetaldehyde (**1c**). However, **1c** is unstable and cannot be isolated. The disilyl acetal **1c′**, which is the precursor of **1c**, was used as its synthetic equivalent (Scheme 4). The reactions of **1c′** with **2a** did not provide 1,8-diphenylphenanthrene (**6a**). With more reactive alkyne (4-ethynylanisole), the target product **6b** was isolated in 12% yield. Diphenylacetylene was also prone to react to give the target product 1,2,7,8-tetraphenylphenanthrene (**6c**) in 10% yield. The reaction with bromide-substituted phenylacetylene gave **6d** in 22% yield with excellent regioselectivity. The second annulation of the intermediate 1,2,5-trisubstituted naphthalene (Scheme 4, Intermediate **C**) with alkynes only underwent through the β-position (6 position).12*d* Some α-aryl-substituted diacetaldehyde and diketones were also tested, but no corresponding products were formed under the current conditions (see ESI†).

**Scheme 5:**
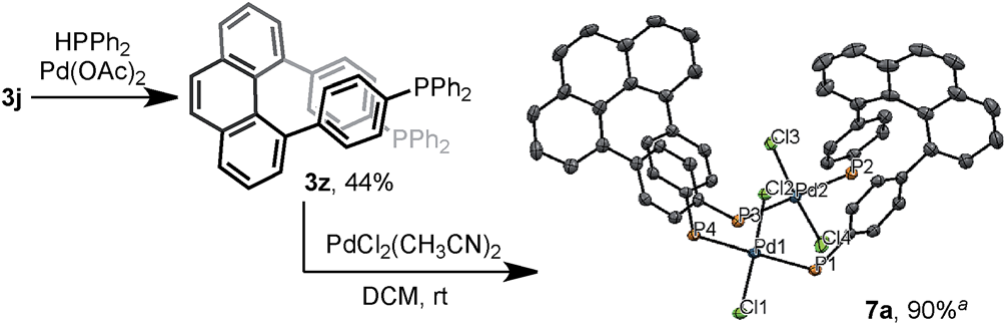
The synthesis of a unique chiral diphosphine ligand **3z** and binuclear Pd complex **7a**. Note: ^a^X-ray structure of binuclear Pd complex **7a** at 50% thermal probability. Hydrogen atoms, phenyl groups, and solvent molecules are omitted for clarity.

The multisubstituted phenanthrenes derived from bisannulation reactions can serve as useful building blocks for the synthesis of organic materials and ligands. To demonstrate the utility of this method, the new helical, chiral diphosphine ligand^20^**3z** was prepared *via* the coupling reaction of **3j** with HPPh_2_ (Scheme 5). When treating **3z** with [PdCl_2_(CH_3_CN)_2_], interesting binuclear palladium complex **7a** was generated in 90% yield.^21^ The X-ray structural analysis showed that **7a** consists of two molecules of **3z** with same chiral sense. There is no bonding between two Pd atoms and the complex features a *trans*-spanned chelating coordination with slightly distorted Cl–Pd–Cl angles (171.38(4)° and 170.76(4)°). The P1–P2 distance (6.9277(12) Å) is much larger than the P1–P4 distance (4.6528(12) Å). Such a large P–P distance and the helical structure of **3z** might provide the opportunity to interact with metal ions^22^ in unique coordination patterns to give a good catalytic activity.^23^

## Conclusions

In summary, we have developed a concise acid-catalyzed cascade annulation to prepare sterically hindered 4,5-disubstituted phenanthrenes with high regioselectivity from readily available 1,4-benzenediacetaldehyde and terminal aryl alkynes. However, the terminal aryl alkynes with strong electron-withdrawing substituents are not reactive. With internal aryl alkynes, 3,4,5,6-tetrasubstituted phenanthrenes are provided in moderate yield. Furthermore, this method could be extended to reactions of alkynes with isomeric 1,3-benzenediacetaldehyde and 1,2-benzenediacetaldehyde disilyl acetal to provide regioselective multisubstituted phenanthrenes. In addition, a new helical chiral diphosphine ligand was prepared. The reaction presented here is not only interesting in terms of the regioselective annulation, but it is also valuable for the preparation of twisted phenanthrenes and multisubstituted phenanthrenes.

## Conflicts of interest

There are no conflicts to declare.

## Supplementary Material

Supplementary information

Crystal structure data
