# Supplementary material for: Synthesis of sterically hindered 4,5-diarylphenanthrenes via acid-catalyzed bisannulation of benzenediacetaldehydes with alkynes
Source: Chem Sci. 2019 Apr 17;10(21):5470–5. doi: 10.1039/c9sc00334g (PMC6552489; doi:10.1039/c9sc00334g)
Supplement: Supplementary file 1 [file SC-010-C9SC00334G-s001.pdf]

Electronic Supplementary Material (ESI) for Chemical Science.
This journal is © The Royal Society of Chemistry 2019

Supporting information

Synthesis of Sterically Hindered 4,5-Diarylphenanthrenes via Acid-catalyzed Bisannulation of Benzenediacetaldehydes with Alkynes

Yuanming Li[a], Akiko Yagi[ab], and Kenichiro Itami*[abc]

[a]Institute of Transformative Bio-Molecules (WPI-ITbM), Nagoya University, Chikusa, Nagoya 464-8602, Japan

[b]Graduate School of Science, Nagoya University, Chikusa, Nagoya 464-8602, Japan

[c]JST-ERATO Itami Molecular Nanocarbon Project, Chikusa, Nagoya 464-8602, Japan

E-mail: itami@chem.nagoya-u.ac.jp (K.I.).

Table of Contents

1. Instrumentation and chemicals 3

2. Preparation of the diacetaldehyde compounds **1** 4

3. The self-polymerization of the diacetaldehyde compound **1a** 6

4. Reaction of diacetaldehyde compounds with alkynes 6

5. The optimization of scale-up reaction 23

6. The optimization of asymmetric bisannulation 26

7. The formation of regioisomers (**3y** and **3y'**) 27

8. The detailed mechanism 29

9. The synthesis of diphosphine ligand 30

10. Variable temperature ^{1}H NMR spectrum (**3m** and **3q**) and 1D NOE spectrum (**3q**) 32

11. Reaction of α-aryl-substituted diacetaldehyde and diketones with alkynes 35

12. Mulliken charges for the intermediate **A** 36

13. X-ray data 37

14. The resolution of **3b** and **3x** 39

15. Free energy of activation for racemization of **3n** 41

16. Erosion in e.r. of **3n** at 70 °C in hexane and 85 °C in 1,2-dichloroethane 43

17. The optical rotation and CD spectrum of **3n**. 45

18. References 46

19. NMR spectra of all unknown compounds 46

1. Instrumentation and chemicals

Unless otherwise noted, all reactants or reagents including dry solvents were obtained from commercial suppliers and used as received. Unless otherwise noted, all reactions were performed with dry solvents under an atmosphere of argon in dried glassware using standard vacuum-line techniques. All work-up and purification procedures were carried out with reagent-grade solvents in air.

Analytical thin-layer chromatography (TLC) was performed using E. Merck silica gel 60 F254 precoated plates (0.25 mm); detection with UV light or by dipping into a solution of $KMnO_4$ (1.5 g in 400 mL H_2O, 5 g $NaHCO_3$), followed by heating. Flash column chromatography was performed with E. Merck silica gel 60 (230–400 mesh). The developed chromatogram was analyzed by UV lamp (254 nm). Medium Pressure liquid chromatography (MPLC) was performed using Yamazen W-prep 2XY. Preparative thin-layer chromatography (PTLC) was performed using Wakogel B5-F silica coated plates (0.75 mm) prepared in our laboratory. Preparative gel permeation chromatography (GPC) was performed with a JAI LC-9204 instrument equipped with JAIGEL-1H/JAIGEL-2H columns using chloroform as an eluent. Gas chromatography (GC) analysis was conducted on a Shimadzu GC-2010 instrument equipped with a HP-5 column (30 m · 0.25 mm, Hewlett-Packard) with dodecane as an internal standard.

The high-resolution mass spectra (HRMS) were conducted on Thermo Fisher Scientific Exactive. Infrared spectra were recorded on a JASCO FTIR-6100 spectrometer. Nuclear magnetic resonance (NMR) spectra were recorded on a JEOLJNM-ECA-600 (^{1}H 600 MHz, ^{13}C 150 MHz) spectrometer and a JEOL JNM-ECA-400 (^{1}H 400 MHz, ^{13}C 100 MHz) spectrometer. Chemical shifts for ^{1}H NMR are expressed in parts per million (ppm) relative to tetramethylsilane (δ 0.00 ppm) or residual peak of acetone-d_6 (δ 2.05 ppm). Chemical shifts for ^{13}C NMR are expressed in ppm relative to $CDCl_3$ (δ 77.16 ppm) or acetone-d_6 (δ 29.84 ppm). Data are reported as follows: chemical shift, multiplicity (s = singlet, d = doublet, dd = doublet of doublets, ddd = doublet of doublets of doublets, t = triplet, dt = doublet of triplets, td = triplet of doublets, q = quartet, p = quintet, m = multiplet, brs = broad singlet, brd = broad doublet), coupling constant (Hz), and integration.

2. Preparation of the diacetaldehyde compounds 1

General procedure 1 (GP1):
The synthesis of benzenediacetaldehyde 1

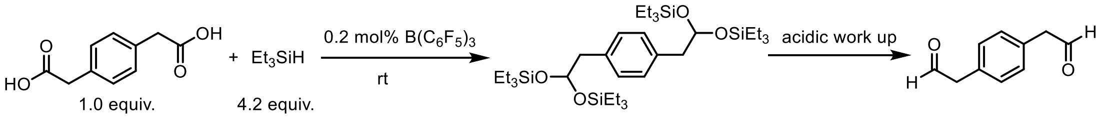

A dry Schlenk tube equipped with a magnetic stir bar and a septum was charged with 1,4-phenylenediacetic acid (1.94 g, 10.0 mmol) and $B(C_6F_5)_3$ (10.2 mg, 20.0 μmol) in a glovebox. C_6H_6 (10 mL) was then added under argon atmosphere. After that, Et_3SiH (4.88 g/6.71 mL, 42.0 mmol) was added dropwise at 23 °C. The resulting mixture was monitored by ^1H NMR spectroscopy. When the reaction was completed (about 6 hours), all volatiles (solvent and excess of Et_3SiH) were removed under vacuum to give the crude product of **1a'**. Subsequently, 40 mL of THF was added, followed by 40 mL of 1 M HCl (aq) with vigorous stirring. The reaction mixture was further stirred for 3 h at room temperature and was extracted with ethyl acetate (3×30 mL). The combined organic layers were dried over anhydrous $MgSO_4$, filtered and concentrated under vacuum. The residue was then purified by silica gel column chromatography.

1,4-Benzenediacetaldehyde (1a)

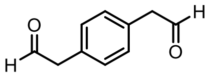

1a was prepared according to GP1 with 1,4-phenylenediacetic acid (1.94 g, 10.0 mmol, purchased from Aldrich, white powder) and Et_3SiH (4.88 g/6.71 mL, 42.0 mmol). Purification by silica gel column chromatography (ethyl acetate/hexane =1/1) gave **1a** (840 mg, 5.7 mmol, 57%) as a white solid. **R*f*** (ethyl acetate/hexane = 1/3): 0.4; ^1H NMR (600 MHz, $CDCl_3$) δ 9.76 (t, J = 2.3 Hz, 2H), 7.23 (s, 4H), 3.71 (d, J = 2.3 Hz, 4H). ^{13}C NMR (75 MHz, $CDCl_3$) δ 199.3, 131.2, 130.4, 50.3. **HRMS (ESI)**: Exact mass calculated for $C_{10}H_{10}NaO_2$ ($[M+Na]^+$): 185.0573, mass found: 185.0576. The product can be stored at –30 °C for at least three months without decomposition.

1,3-Benzenediacetaldehyde (1b)

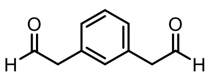

1b was prepared according to GP1 with 1,3-phenylenediacetic acid (0.97 g, 5.0 mmol) and Et_3SiH (2.4 g/3.4 mL, 21 mmol). The reaction time was about 12 hours. Purification by silica gel column chromatography (ethyl acetate/hexane =1/2) gave **1b** (150 mg, 1.0 mmol, 20%) as a colorless liquid. **R*f*** (ethyl acetate/hexane = 1/2): 0.4; ^{1}H NMR (392 MHz, $CDCl_3$) δ 9.76 (t, *J* = 2.3 Hz, 2H), 7.38 (t, *J* = 7.6 Hz, 1H), 7.17 (dd, *J* = 7.6, 1.7 Hz, 2H), 7.08 (s, 1H), 3.71 (d, *J* = 2.2 Hz, 4H). ^{13}C NMR (151 MHz, $CDCl_3$) δ 199.1, 132.8, 131.0, 129.8, 128.9, 50.5. **HRMS (ESI)**: Exact mass calculated for $C_{10}H_{10}NaO_2$ ($[M+Na]^+$): 185.0573, mass found: 185.0571. The product can be stored at –30 °C for at least three months without decomposition.

The synthesis of benzenediacetaldehyde disilyl acetal 1c'

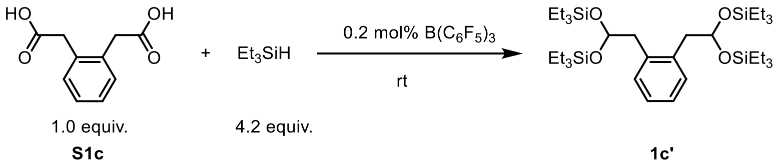

A dry Schlenk tube equipped with a magnetic stir bar and a septum was charged with 1,2-phenylenediacetic acid (0.97 g, 5.0 mmol) and $B(C_6F_5)_3$ (5.1 mg, 10.0 μmol) in a glovebox. C_6H_6 (5 mL) was then added under argon atmosphere. After that, Et_3SiH (2.4 g/3.4 mL, 21.0 mmol) was added dropwise at 23 °C. The resulting mixture was monitored by ^{1}H NMR spectroscopy. When the reaction was completed (about 12 hours), all volatiles (solvent and excess of Et_3SiH) were removed under vacuum to give the crude product of **1c'**. Purification by silica gel column chromatography (DCM/hexane =1/3) gave **1c'** (2.0 g, 3.0 mmol, 60%) as a colorless liquid. **R*f*** (DCM/hexane = 1/3): 0.4; ^{1}H NMR (600 MHz, $CDCl_3$) δ 7.16-6.99 (m, 4H), 5.24 (t, *J* = 5.5 Hz, 2H), 2.97 (d, *J* = 5.5 Hz, 4H), 0.89 (t, *J* = 8.0 Hz, 36H), 0.54 (qd, *J* = 7.9, 2.3 Hz, 24H). ^{13}C NMR (151 MHz, $CDCl_3$) δ 136.8, 130.9, 126.2, 94.3, 44.5, 6.9, 5.3. **HRMS (ESI)**: Exact mass calculated for $C_{34}H_{70}NaO_4Si_4$ ($[M+Na]^+$): 677.4243, mass found: 677.4179. The product can be stored at –30 °C for at least three months without decomposition.

3. The self-polymerization of the diacetaldehyde compound 1a

For example, based on the crude ^{1}H NMR data, the reaction of 1,4-benzenediacetaldehyde (1.0 equiv.) with 4-bromophenylacetylene (2.5 equiv.) gave the corresponding product in 44% NMR yield and 1.3 equiv. of 4-bromophenylacetylene was recovered. 1,4-benzenediacetaldehyde was consumed completely. The crude 1H NMR spectra was very clear, we could only see the peaks derived from the product and the 4-bromophenylacetylene in the aromatic region. However, when we removed the solvent of the reaction mixture after the reaction was finished; the residual of the reaction could not dissolve totally in $CHCl_3$, acetone or water. We presume the insoluble residual is derived from the self-polymerization of 1,4-benzenediacetaldehyde. To prove the hypothesis, when the reaction was run without alkynes, 1,4-benzenediacetaldehyde was consumed, and the insoluble solid was formed.

4. Reaction of diacetaldehyde compounds with alkynes

General procedure 2 (GP2):

Reaction of diacetaldehyde compounds with terminal alkynes

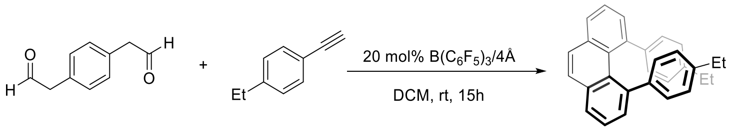

1a 2b 3b

A dry Schlenk tube equipped with a magnetic stir bar and a septum was charged with activated molecular sieve (100 mg, 4Å, 1/16 in., pellets), diacetaldehyde compound **1a** (16 mg, 0.1 mmol) and $B(C_6F_5)_3$ (10 mg, 20 μmol) in a glovebox. DCM (2.5 mL) was then added under argon atmosphere. After that, alkyne **2b** (33 mg, 0.25 mmol) was added at room temperature. After stirring at room temperature for 15 h, the reaction mixture was quenched by water. And then the resulting mixture was extracted with DCM three times. The organic extracts were washed with water, brine, dried over $MgSO_4$, and concentrated. The residue was then purified by silica gel column chromatography directly.

General procedure 3 (GP3):

Reaction of diacetaldehyde compounds with internal alkynes

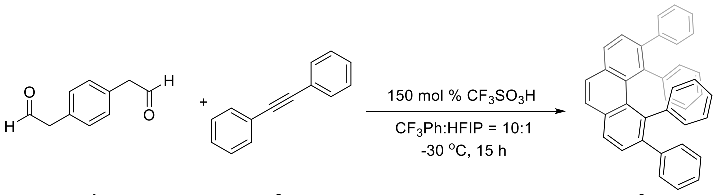

1a **2x** **3x**

A dry Schlenk tube equipped with a magnetic stir bar and a septum was charged with diacetaldehyde compound **1a** (16 mg, 0.10 mmol) and internal alkyne **2x** (45 mg, 2.5 mmol) under air. CF_3Ph (2.5 mL) and hexafluoroisopropanol (HFIP) (0.25 mL) were then added under argon atmosphere. Subsequently, the reaction mixture was cooled to –30 °C. After 15 minutes, the freshly prepared solution of CF_3SO_3H (0.5 M in HFIP, 300 μL, 0.15 mmol) was added dropwise. After stirring at –30 °C for 15 h, the resulting mixture was warmed to room temperature and 1.0 M Na_2CO_3 solution (10 mL) was added. The result solution was extracted with DCM (3×10 mL). The combined organic layers were dried over anhydrous $MgSO_4$, filtered and concentrated under vacuum. The residue was then purified by silica gel column chromatography.

4,5-Diphenylphenanthrene (3a)

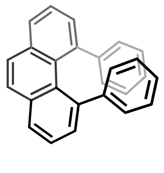

3a was prepared according to GP2 with **1a** (16 mg, 0.10 mmol) and phenylacetylene (**2a**) (31 mg, 0.25 mmol). Purification by silica gel chromatography (DCM/hexane = 1/10) gave **3a** (16 mg, 0.048 mmol, 48%). **R*f*** (DCM/hexane = 1/10): 0.4; ^{1}H NMR (600 MHz, $CDCl_3$) δ 7.76 (dd, *J* = 7.8, 1.4 Hz, 2H), 7.70 (s, 2H), 7.51 (t, *J* = 7.5 Hz, 2H), 7.13 (dd, *J* = 7.2, 1.3 Hz, 2H), 7.07 (tt, *J* = 7.3, 1.2 Hz, 2H), 6.99 (t, *J* = 6.7 Hz, 2H), 6.94 (t, *J* = 7.0 Hz, 2H), 6.60 (d, *J* = 7.4 Hz, 2H), 6.52 (d, *J* = 7.2 Hz, 2H). ^{13}C NMR (151 MHz, $CDCl_3$) δ 143.6, 142.1, 134.8, 129.7, 129.6, 128.3, 128.0, 127.17, 127.15, 126.9, 126.8, 126.7, 125.6. The spectroscopic data is in agreement with this previously reported in the literature.[1]

4,5-Bis(4-ethylphenyl)phenanthrene (3b)

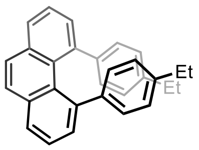

3b was prepared according to GP2 with **1a** (16 mg, 0.10 mmol) and 1-ethyl-4-ethynylbenzene (**2b**) (33 mg, 0.25 mmol) as yellow liquid. Purification by silica gel chromatography (DCM/hexane = 1/10) gave **3b** (25 mg, 0.065 mmol, 65%). **R*f*** (DCM/hexane = 1/5): 0.5; ^{1}H NMR (600 MHz, $CDCl_3$) δ 7.75 (dd, *J* = 7.8, 1.4 Hz, 2H), 7.69 (s, 2H), 7.51 (dd, *J* = 7.3, 7.6 Hz, 2H), 7.15 (dd, *J* = 7.2, 1.4 Hz, 2H), 6.84 (d, *J* = 7.0 Hz, 2H), 6.80 (d, *J* = 7.2 Hz, 2H), 6.55 (d, *J* = 7.1 Hz, 2H), 6.46 (d, *J* = 6.9 Hz, 2H), 2.66-2.56 (m, 4H), 1.32 (t, *J* = 7.6 Hz, 6H). ^{13}C NMR (151 MHz, $CDCl_3$) δ 142.0, 141.6, 141.1, 134.7, 129.4, 129.2, 128.1, 127.9, 127.1, 126.73, 126.69, 126.62, 126.57, 28.8, 16.3. It should be noted that broad peaks appeared for the bay region aryl protons in the ^{1}H NMR spectra of **3b**. This is due to the restricted free rotation of the bay region aryl substituents at room temperature. **HRMS (APCI)**: Exact mass calculated for $C_{30}H_{27}$ ($[M+H]^+$): 387.2107, mass found: 387.2102.

4,5-Bis(4-(tert-butyl)phenyl)phenanthrene (3c)

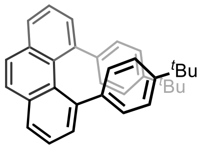

3c was prepared according to GP2 with **1a** (16 mg, 0.10 mmol) and 1-*tert*-butyl-4-ethynylbenzene (**2c**) (33 mg, 0.25 mmol). Purification by silica gel chromatography (DCM/hexane = 1/10) gave **3c** (25 mg, 0.056 mmol, 56%) as a white solid. **R*f*** (DCM/hexane = 1/10): 0.3; ^{1}H NMR (600 MHz, $CDCl_3$) δ 7.75 (dd, *J* = 7.7, 1.3 Hz, 2H), 7.69 (s, 2H), 7.50 (t, *J* = 7.5 Hz, 2H), 7.16 (dd, *J* = 7.2, 1.3 Hz, 2H), 7.03-6.95 (m, 4H), 6.58 (d, *J* = 7.0 Hz, 2H), 6.42 (d, *J* = 7.1 Hz, 2H), 1.37 (s, 18H). ^{13}C NMR (151 MHz, $CDCl_3$) δ 148.1, 141.8, 140.8, 134.8, 129.5, 128.1, 127.4, 127.2, 126.7, 126.7, 126.6, 126.3, 124.0, 34.5, 31.6. **HRMS (APCI)**: Exact mass calculated for $C_{34}H_{35}$ ($[M+H]^+$): 443.2733, mass found: 443.2736.

4,5-Bis(4-methoxyphenyl)phenanthrene (3d)

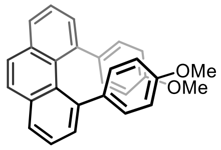

3d was prepared according to GP2 with **1a** (16 mg, 0.10 mmol) and 1-methoxyl-4-ethynylbenzene (**2d**) (33 mg, 0.25 mmol) as a white solid. Purification by silica gel chromatography (ethyl acetate/hexane = 1/10) gave **3d** (26 mg, 0.067 mmol, 67%). **R*f*** (ethyl acetate/hexane = 1/10): 0.3; ^{1}H NMR (600 MHz, $CDCl_3$) δ 7.72 (dd, *J* = 7.8, 1.4 Hz, 2H), 7.66 (s, 2H), 7.51-7.45 (m, 2H), 7.11 (dd, *J* = 7.2, 1.4 Hz, 2H), 6.66-6.48 (m, 8H), 3.81 (s, 6H).· ^{13}C NMR (151 MHz, $CDCl_3$) δ 158.1, 141.5, 136.7, 134.7, 129.2, 128.9, 128.1, 127.7, 127.1, 126.6, 126.4, 115.0, 112.9, 55.5. **HRMS (APCI)**: Exact mass calculated for $C_{28}H_{23}O_2$ ($[M+H]^+$): 391.1693, mass found: 391.1686.

4,5-Bis(4-(phenylethynyl)phenyl)phenanthrene (3e)

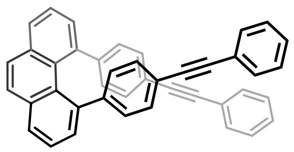

3e was prepared according to GP2 with **1a** (16 mg, 0.10 mmol) and 1-ethynyl-4-(phenylethynyl)benzene (**2e**) (51 mg, 0.25 mmol). Purification by silica gel chromatography (DCM/hexane = 1/10) gave **3e** (30 mg, 0.056 mmol, 56%) as a white solid. **R*f*** (DCM/hexane = 1/5): 0.4; ^{1}H NMR (600 MHz, $CDCl_3$) δ 7.80 (dd, *J* = 7.8, 1.3 Hz, 2H), 7.72 (s, 2H), 7.59 (dd, *J* = 8.0, 1.4 Hz, 4H), 7.54 (t, *J* = 7.5 Hz, 2H), 7.41-7.33 (m, 6H), 7.31 (brd, *J* = 7.9 Hz, 2H), 7.18 (dd, *J* = 7.2, 1.3 Hz, 2H), 7.16 (brd, *J* = 7.9 Hz, 2H), 6.67 (brd, *J* = 7.9 Hz, 2H), 6.62 (brd, *J* = 7.9 Hz, 2H). ^{13}C NMR (151 MHz, $CDCl_3$) δ 143.4, 141.2, 134.8, 133.1, 131.8, 130.6, 129.4, 128.5, 128.4, 128.3, 127.7, 127.4, 127.3, 126.9, 126.9, 123.6, 120.6, 90.1, 89.6. **HRMS (APCI)**: Exact mass calculated for $C_{42}H_{27}$ ($[M+H]^+$): 531.2107, mass found: 531.2104.

N,N'-(phenanthrene-4,5-diylbis(4,1-phenylene))diacetamide (3g)

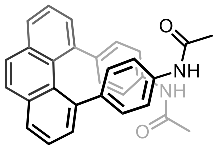

3g was prepared according to GP2 with **1a** (16 mg, 0.10 mmol) and 4-ethynyl acetanilide (**2g**) (40 mg, 0.25 mmol) in the mixture of DCM/HFIP (10:1 v/v). Purification by silica gel chromatography (hexane/ethyl acetate = 1/1, and then DCM/hexane = 1/5) with short column gave **3g** (12 mg, 0.027 mmol, 27%) as a yellow solid. **R_f** (hexane/ethyl acetate = 1/1): 0.1; ^{1}H NMR (600 MHz, $CDCl_3$) δ 7.74-7.69 (m, 4H), 7.66 (s, 2H), 7.45 (t, J = 7.5 Hz, 2H), 7.14 (d, J = 7.9 Hz, 2H), 7.11 (d, J = 7.6 Hz, 2H), 7.06 (dd, J = 7.3, 1.2 Hz, 2H), 6.53 (d, J = 8.8 Hz, 4H), 2.16 (s, 6H). ^{13}C NMR (151 MHz, $CDCl_3$) δ 168.6, 141.2, 139.9, 135.9, 134.8, 129.4, 128.5, 127.8, 127.3, 127.2, 126.9, 126.7, 121.4, 118.8, 24.8. **HRMS (ESI)**: Exact mass calculated for $C_{30}H_{24}N_2NaO_2$ ($[M+H]^+$): 467.1730, mass found: 467.1718.

4,5-Bis(4-fluorophenyl)phenanthrene (3h)

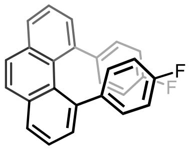

3h was prepared according to GP2 with **1a** (16 mg, 0.10 mmol) and 4-fluorophenylacetylene (**2h**) (30 mg, 0.25 mmol) in the mixture of DCM/HFIP (10:1 v/v). Purification by silica gel chromatography (DCM/hexane = 1/10) gave **3h** (16 mg, 0.044 mmol, 44%) as a white solid. **R_f** (DCM/hexane = 1/5): 0.4; ^{1}H NMR (600 MHz, $CDCl_3$) δ 7.77 (dd, *J* = 7.8, 1.4 Hz, 2H), 7.69 (s, 2H), 7.51 (dd, *J* = 7.7, 7.3 Hz, 2H), 7.11 (dd, *J* = 7.3, 1.4 Hz, 2H), 6.79 (t, *J* = 8.5 Hz, 2H), 6.68 (t, *J* = 8.3 Hz, 2H), 6.63-6.55 (m, 4H). ^{13}C NMR (151 MHz, $CDCl_3$) δ 161.7 (d, *J* = 245.2 Hz), 140.6, 139.7 (d, *J* = 3.2 Hz), 134.9, 129.53, 129.48 (d, *J* = 7.8 Hz), 128.2 (d, *J* = 8.6 Hz), 127.7, 127.3, 127.2, 126.8, 116.4 (d, *J* = 21.4 Hz), 114.3 (d, *J* = 20.9 Hz). ^{19}F NMR (564 MHz, $CDCl_3$) δ -117.3. **HRMS (EI)**: Exact mass calculated for $C_{26}H_{16}F_2$ ($[M]^+$): 366.1220, mass found: 366.1227.

4,5-Bis(4-bromophenyl)phenanthrene (3i)

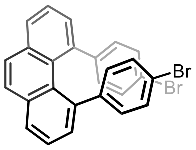

3i was prepared according to GP2 with **1a** (16 mg, 0.10 mmol) and 4-bromophenylacetylene (**2i**) (45 mg, 0.25 mmol) in the mixture of DCM/HFIP (10:1 v/v). Purification by silica gel chromatography (DCM/hexane = 1/10) gave **3i** (18 mg, 0.037 mmol, 37%) as a white solid. **R*f*** (DCM/hexane = 1/5): 0.4; ^{1}H NMR (600 MHz, $CDCl_3$) δ 7.79 (dd, *J* = 7.8, 1.4 Hz, 2H), 7.70 (s, 2H), 7.55-7.50 (m, 2H), 7.23 (d, *J* = 8.3 Hz, 2H), 7.12 (dd, *J* = 7.3, 1.4 Hz, 2H), 7.08 (d, *J* = 9.4 Hz, 2H), 6.51 (d, *J* = 8.3 Hz, 2H), 6.48 (d, *J* = 8.2 Hz, 2H). ^{13}C NMR (151 MHz, $CDCl_3$) δ 142.4, 140.4, 134.9, 132.7, 130.4, 129.8, 129.5, 128.6, 127.6, 127.44, 127.37, 127.0, 120.1. **HRMS (EI)**: Exact mass calculated for $C_{26}H_{16}Br_2$ ($[M]^+$): 485.9619, mass found: 485.9609.

4,5-Bis(4-iodophenyl)phenanthrene (3j)

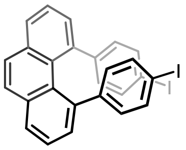

3j was prepared according to GP2 with **1a** (16 mg, 0.10 mmol) and 4-iodophenylacetylene (**2j**) (57 mg, 0.25 mmol) in the mixture of DCM/HFIP (10:1 v/v). Purification by silica gel chromatography (DCM/hexane = 1/10) gave **3j** (20 mg, 0.034 mmol, 34%) as a white solid. **R*f*** (DCM/hexane = 1/10): 0.4; ^{1}H NMR (600 MHz, $CDCl_3$) δ 7.79 (d, *J* = 7.7 Hz, 2H), 7.70 (s, 2H), 7.52 (t, *J* = 7.2 Hz, 2H), 7.44 (d, *J* = 8.1 Hz, 2H), 7.28 (d, *J* = 8.1 Hz, 2H), 7.12 (d, *J* = 7.5 Hz, 2H), 6.37 (t, *J* = 7.7 Hz, 4H). ^{13}C NMR (151 MHz, $CDCl_3$) δ 143.0, 140.5, 138.8, 136.4, 135.0, 130.2, 129.5, 129.1, 127.6, 127.39, 127.38, 127.0, 91.5. **HRMS (EI)**: Exact mass calculated for $C_{26}H_{16}I_2$ ($[M]^+$): 581.9341, mass found: 581.9340.

4,5-Bis(4-(4,4,5,5-tetramethyl-1,3,2-dioxaborolan-2-yl)phenyl)phenanthrene (3l)

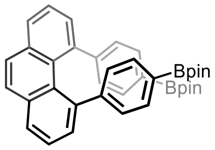

3l was prepared according to GP2 with **1a** (16 mg, 0.10 mmol) and 4-ethynylbenzeneboronic acid pinacol ester (**2l**) (57 mg, 0.25 mmol) in the mixture of DCM/HFIP (10:1 v/v). Purification by silica gel chromatography (ethyl acetate/hexane = 1/10) gave **3l** (12 mg, 0.021 mmol, 21%) as a white solid. $\mathbf{R_{\mathit{f}}}$ (ethyl acetate/hexane = 1/10): 0.4; ^{1}H NMR (600 MHz, $CDCl_3$) δ 7.76 (d, *J* = 7.7 Hz, 2H), 7.69 (s, 2H), 7.49 (t, *J* = 7.5 Hz, 2H), 7.39 (d, *J* = 7.8 Hz, 2H), 7.37 (d, *J* = 7.6 Hz, 2H), 7.12 (d, *J* = 7.2 Hz, 2H), 6.57 (d, *J* = 7.9 Hz, 2H), 6.53 (d, *J* = 7.8 Hz, 2H), 1.38 (s, 24H). ^{13}C NMR (151 MHz, $CDCl_3$) δ 146.4, 142.0, 136.2, 134.7, 133.6, 129.7, 127.9, 127.14, 127.10, 126.7, 126.4, 83.8, 25.1, 24.9. **HRMS (APCI)**: Exact mass calculated for $C_{38}H_{41}B_2O_4$ ($[M+H]^+$): 583.3185, mass found: 583.3192.

4,5-Di-m-tolylphenanthrene (3m)

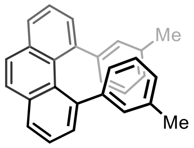

3m was prepared according to GP2 with **1a** (16 mg, 0.10 mmol) and 3-ethynyltoluene (**2m**) (29 mg, 0.25 mmol). Purification by silica gel chromatography (DCM/hexane = 1/10) gave **3m** (21 mg, 0.058 mmol, 58%) as a liquid. $\mathbf{R_{\mathit{f}}}$ (DCM/hexane = 1/5): 0.5; ^{1}H NMR (600 MHz, $CDCl_3$, multiple rotamers) δ 7.76 (d, *J* = 7.7 Hz, 2H), 7.69 (s, 2H), 7.51 (t, *J* = 7.4 Hz, 2H), 7.15 (d, *J* = 7.3 Hz, 2H), 7.00-6.80 (m, 4H), 6.51-6.21 (m, 4H), 2.20 (s, 4.2H), 2.07 (s, 1.8H). ^{13}C NMR (151 MHz, $CDCl_3$) δ 143.2, 142.23, 142.23, 138.8, 138.4, 136.6, 134.7, 129.5, 129.2, 128.9, 128.1, 127.9, 127.2, 127.1, 126.85, 126.76, 126.6, 126.3, 125.5, 124.2, 21.6, 21.4. ^{1}H NMR (600 MHz, tetrachloroethane-d_2 at 110 °C) δ 7.83-7.73 (m, 2H), 7.74-7.68 (m, 2H), 7.58-7.49 (m, 2H), 7.25-7.14 (m, 2H), 7.01-6.84 (m, 4H), 6.50-6.33 (m, 4H), 2.20 (s, 3H). ^{13}C NMR (151 MHz, tetrachloroethane-d_2 at 110 °C) δ 142.9, 142.0, 134.5, 129.0, 128.0, 126.7, 126.4, 126.2, 126.0, 124.9, 21.1 (Three carbons was unsolved). **HRMS (APCI)**: Exact mass calculated for $C_{28}H_{23}$ ($[M+H]^+$): 359.1794, mass found: 359.1795.

4,5-Bis(3,5-di-tert-butylphenyl)phenanthrene (3n)

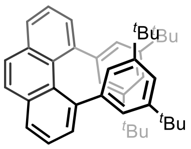

3n was prepared according to GP2 with **1a** (16 mg, 0.10 mmol) and 1,3-di-tert-butyl-5-ethynylbenzene (**2n**) (54 mg, 0.25 mmol) in the mixture of DCM/HFIP (10:1 v/v). Purification by silica gel chromatography (DCM/hexane = 1/10) gave **3n** (19 mg, 0.035 mmol, 35%) as a white solid. $\mathbf{R_f}$ (DCM/hexane = 1/10): 0.5; ^{1}H NMR (600 MHz, $CDCl_3$) δ 7.74 (dd, *J* = 7.7, 1.2 Hz, 2H), 7.69 (s, 2H), 7.51 (t, *J* = 7.5 Hz, 2H), 7.26 (dd, *J* = 7.3, 1.2 Hz, 2H), 7.05 (t, *J* = 1.7 Hz, 2H), 6.75 (s, 4H), 1.24 (s, 18H), 1.06 (s, 18H). ^{13}C NMR (151 MHz, $CDCl_3$) δ 150.3, 148.4, 142.4, 142.1, 134.8, 130.0, 128.2, 127.2, 126.7, 126.5, 122.7, 121.5, 119.7, 34.9, 34.5, 31.8, 31.4. **HRMS (EI)**: Exact mass calculated for $C_{42}H_{50}$ ($[M]^+$): 554.3907, mass found: 554.3907.

4,5-Di([1,1':3',1''-terphenyl]-5'-yl)phenanthrene (3o)

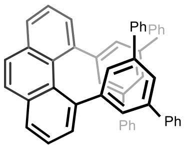

3o was prepared according to GP2 with **1a** (16 mg, 0.10 mmol) and 5'-ethynyl-1,1':3',1''-terphenyl (**2o**) (64 mg, 0.25 mmol) in the mixture of DCM/HFIP (10:1 v/v). Purification by silica gel chromatography (DCM/hexane = 1/5) gave **3o** (16 mg, 0.025 mmol, 25%) as a white solid. $\mathbf{R_f}$ (DCM/hexane = 1/5): 0.3; ^{1}H NMR (600 MHz, $CDCl_3$) δ 7.90 – 7.86 (m, 2H), 7.77 (s, 2H), 7.63 (t, *J* = 7.5 Hz, 2H), 7.46 (t, *J* = 1.6 Hz, 2H), 7.41 (dd, *J* = 7.2, 1.0 Hz, 2H), 7.32 (t, *J* = 7.3 Hz, 4H), 7.30 – 7.21 (m, 16H), 7.03 (s, 2H), 7.01 (s, 2H). ^{13}C NMR (151 MHz, $CDCl_3$) δ 143.8, 141.8, 141.5, 141.3, 140.4, 140.1, 135.0, 129.6, 128.7, 128.4, 128.2, 127.7, 127.4, 127.2, 126.9, 126.9, 126.7, 124.2, 123.4. **HRMS (EI)**: Exact mass calculated for $C_{50}H_{34}$ ($[M]^+$): 634.2655, mass found: 634.2666.

4,5-Bis(3,5-dimethoxyphenyl)phenanthrene (3p)

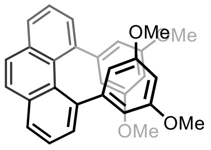

3p was prepared according to GP2 with **1a** (16 mg, 0.10 mmol) and 1-ethynyl-3,5-dimethoxybenzene (**2p**) (41 mg, 0.25 mmol) in the mixture of DCM/HFIP (10:1 v/v). Purification by silica gel chromatography (ethyl acetate/hexane = 1/10) gave **3p** (8.0 mg, 0.018 mmol, 18%) as a liquid. $\mathbf{R_f}$ (ethyl acetate/hexane = 1/5): 0.4; ^{1}H NMR (600 MHz, $CDCl_3$) δ 7.75 (dd, *J* = 7.8, 1.4 Hz, 2H), 7.68 (s, 2H), 7.52-7.46 (m, 2H), 7.24 (dd, *J* = 7.3, 1.4 Hz, 2H), 6.26 (t, *J* = 2.3 Hz, 2H), 5.92-5.87 (m, 2H), 5.79-5.77 (m, 2H), 3.77 (s, 6H), 3.34 (s, 6H). ^{13}C NMR (151 MHz, $CDCl_3$) δ 161.5, 159.8, 145.0, 142.2, 134.7, 128.6, 128.1, 127.2, 127.1, 126.7, 106.5, 105.3, 99.2, 55.2, 55.0. **HRMS (APCI)**: Exact mass calculated for $C_{30}H_{27}O_4$ ($[M+H]^+$): 451.1904, mass found: 451.1895.

4,5-Di-o-tolylphenanthrene (3q)

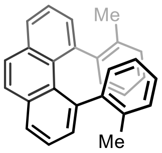

3q was prepared according to GP2 with **1a** (16 mg, 0.10 mmol) and 2-ethynyltoluene (**2q**) (29 mg, 0.25 mmol). Purification by silica gel chromatography (DCM/hexane = 1/10) gave **3q** (21 mg, 0.058 mmol, 58%) as a liquid. $\mathbf{R_f}$ (DCM/hexane = 1/5): 0.4; ^{1}H NMR (600 MHz, tetrachloroethane-d_2 at 25 °C, multiple rotamers) δ 7.86-7.76 (m, 2H), 7.75-7.66 (m, 2H), 7.59-7.44 (m, 2H), 7.26-6.99 (m, 4H), 6.98-6.73 (m, 4H), 6.46-6.28 (m, 2H), 1.85-0.8 (m, 6H). ^{13}C NMR (151 MHz, tetrachloroethane-d_2 at 25 °C, multiple rotamers) δ 143.85, 143.80, 142.7, 141.6, 140.3, 139.4, 135.8, 134.6, 134.1, 133.2, 133.0, 132.9, 131.9, 131.4, 131.2, 130.4, 130.2, 129.9, 129.8, 129.6, 129.4, 128.9, 128.5, 127.5, 127.1, 127.0, 126.8, 126.6, 126.54, 126.52, 126.4, 126.25, 126.21, 125.8, 125.6, 125.5, 124.7, 21.4, 20.1, 19.7, 19.5. **HRMS (APCI)**: Exact mass calculated for $C_{28}H_{23}$ ($[M+H]^+$): 359.1794, mass found: 359.1789

4,5-Dimesitylphenanthrene (3r)

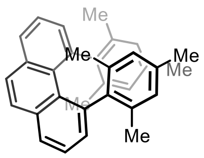

3r was prepared according to GP2 with **1a** (32 mg, 0.20 mmol) and 2-ethynyl-1,3,5-trimethylbenzene (**2r**) (72 mg, 0.50 mmol) in the mixture of DCM/HFIP (10:1 v/v). Purification by silica gel chromatography (DCM/hexane = 1/10) and then GPC to give **3r** (6 mg, 0.015 mmol, 7%) as a white solid. ^{1}H NMR (600 MHz, $CDCl_3$) δ 7.71 (dd, *J* = 7.7, 1.4 Hz, 2H), 7.61 (s, 2H), 7.42 (d, *J* = 7.5 Hz, 2H), 6.98 (dd, *J* = 7.3, 1.4 Hz, 2H), 6.78 (brs, 2H), 6.49 (brs, 2H), 2.25 (s, 6H), 1.56 (brs, 6H), 0.79 (brs, 6H). ^{13}C NMR (151 MHz, $CDCl_3$) δ 140.4, 139.2, 138.0, 136.0, 134.0, 132.3, 131.5, 129.8, 128.5 127.3, 126.3, 125.8, 21.2, 21.0, 20.8. **HRMS (EI)**: Exact mass calculated for $C_{32}H_{30}$ ($[M]^+$): 414.2342, mass found: 414.2355.

4,5-Di(thiophen-2-yl)phenanthrene (3s)

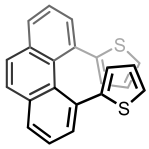

3s was prepared according to GP2 with **1a** (16 mg, 0.10 mmol) and 2-ethynylthiophene (**2s**) (27 mg, 0.25 mmol). Purification by silica gel chromatography (DCM/hexane = 1/5) gave **3s** (16 mg, 0.047 mmol, 47%) as a white solid. **R*f*** (DCM/hexane = 1/5): 0.4; ^{1}H NMR (600 MHz, $CDCl_3$) δ 7.73 (dd, *J* = 7.8, 1.3 Hz, 2H), 7.65 (s, 2H), 7.53-7.43 (m, 2H), 7.28 (dd, *J* = 7.3, 1.3 Hz, 2H), 7.09 (dd, *J* = 5.1, 1.2 Hz, 2H), 6.76 (dd, *J* = 5.0, 3.6 Hz, 2H), 6.43 (dd, *J* = 3.6, 1.2 Hz, 2H). ^{13}C NMR (151 MHz, $CDCl_3$) δ 145.9, 135.1, 134.8, 129.2, 128.0, 127.11, 127.09, 127.07, 126.9, 125.2, 123.9. **HRMS (APCI)**: Exact mass calculated for $C_{22}H_{15}S_2$ ($[M+H]^+$): 343.0610, mass found: 343.0611.

4,5-Di(thiophen-3-yl)phenanthrene (3t)

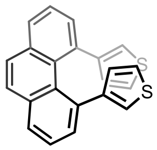

3t was prepared according to GP2 with **1a** (16 mg, 0.10 mmol) and 3-ethynylthiophene (**2t**) (27 mg, 0.25 mmol). Purification by silica gel chromatography (DCM/hexane = 1/5) gave **3t** (21 mg, 0.061 mmol, 61%) as a white solid. $\mathbf{R}_f$ (DCM/hexane = 1/5): 0.5; ^{1}H NMR (600 MHz, $CDCl_3$) δ 7.73 (dd, *J* = 7.8, 1.3 Hz, 2H), 7.66 (s, 2H), 7.53-7.45 (m, 2H), 7.24 (d, *J* = 7.2 Hz, 2H), 7.01-6.93 (m, 2H), 6.57 (s, 2H), 6.42 (s, 2H). ^{13}C NMR (151 MHz, $CDCl_3$) δ 144.5, 136.7, 134.6, 128.5, 128.4, 127.2, 127.0, 126.7, 126.6, 124.1, 119.6. **HRMS (APCI)**: Exact mass calculated for $C_{22}H_{15}S_2$ ($[M+H]^+$): 343.0610, mass found: 343.0610.

4,5-Diethoxyphenanthrene (3w)

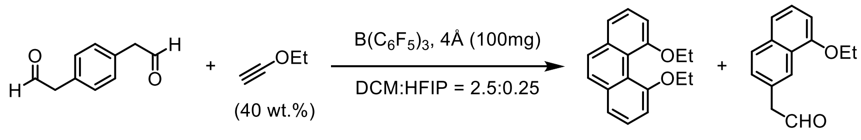

3w was prepared according to GP2 with **1a** (16 mg, 0.10 mmol) and 3-ethynylthiophene (**2w**) (~40 wt. % in hexanes, 60 μL, 0.25 mmol). Purification by silica gel chromatography (DCM/hexane = 1/3) gave **3w** (0.5 mg, 0.0019 mmol, isolated yield 2%, ^{1}H NMR yield 2%). **R*f*** (DCM/hexane = 1/5): 0.3; ^{1}H NMR (600 MHz, $CDCl_3$) δ 7.55 (s, 2H), 7.47 (t, *J* = 7.8 Hz, 2H), 7.39 (dd, *J* = 7.8, 1.1 Hz, 2H), 7.05 (dd, *J* = 7.9, 1.1 Hz, 2H), 4.16 (q, *J* = 7.0 Hz, 4H), 1.44 (t, *J* = 7.0 Hz, 6H). ^{13}C NMR (151 MHz, $CDCl_3$) δ 157.5, 134.7, 126.9, 126.7, 119.5, 119.3, 108.8, 63.7, 15.2. **HRMS (ESI)**: Exact mass calculated for $C_{18}H_{18}NaO_2$ ($[M+Na]^+$): 289.1199, mass found: 289.1196.

Purification by silica gel chromatography (ethyl acetate/hexane = 1/10) gave **3w'** (1.8 mg, 0.0084 mmol, isolated yield 8.4%, ^{1}H NMR yield 11%). **R*f*** (DCM/hexane = 1/5): 0.1; **R*f*** (DCM/hexane = 1/5): 0.1; ^{1}H NMR (600 MHz, $CDCl_3$) δ 9.82 (t, *J* = 2.5 Hz, 1H), 8.20-8.11 (m, 1H), 7.80 (d, *J* = 8.3 Hz, 1H), 7.41 (d, *J* = 8.2 Hz, 1H), 7.37 (dd, *J* = 8.2, 7.5 Hz, 1H), 7.32 (dd, *J* = 8.4, 1.8 Hz, 1H), 6.82 (dd, *J* = 7.4, 1.0 Hz, 1H), 4.22 (q, *J* = 7.0 Hz, 2H), 3.86 (d, *J* = 2.5 Hz, 2H), 1.56 (t, *J* = 7.0 Hz, 3H). ^{13}C NMR (151 MHz, $CDCl_3$) δ 199.8, 154.7, 133.8, 128.7, 128.5, 128.0, 126.3, 126.1, 123.1, 120.0, 105.3, 63.9, 51.1, 15.0. **HRMS (ESI)**: Exact mass calculated for $C_{14}H_{14}NaO_2$ ($[M+Na]^+$): 237.0886, mass found: 237.0887.

3,4,5,6-Tetraphenylphenanthrene (3x)

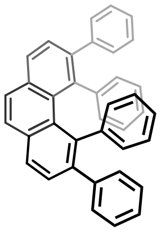

3x was prepared according to GP3 with **1a** (16 mg, 0.10 mmol) and diphenylacetylene (**2x**) (45 mg, 0.25 mmol). Purification by silica gel chromatography (DCM/hexane = 1/5) gave **3x** (20 mg, 0.041 mmol, 41%) as a white solid. **R*f*** (DCM/hexane = 1/5): 0.2; ^{1}H NMR (600 MHz, CDCl3) δ 7.79 (d, *J* = 8.0 Hz, 2H), 7.66 (s, 2H), 7.43 (d, *J* = 8.0 Hz, 2H), 7.07 (t, *J* = 7.4 Hz, 2H), 6.84 (t, *J* = 7.3 Hz, 2H), 7.65-5.50 (broad peak, 16H). ^{1}H NMR (600 MHz, $CDCl_3$ at 55 °C) δ 7.78 (d, *J* = 8.0 Hz, 2H), 7.65 (s, 2H), 7.42 (d, *J* = 8.0 Hz, 2H), 7.07 (t, *J* = 6.6 Hz, 7H), 6.84 (t, *J* = 7.9 Hz, 3H), 6.63 (brs, 6H), 6.04 (brs, 4H). ^{13}C NMR (151 MHz, $CDCl_3$) δ 143.4, 139.8, 139.6, 134.3, 132.3, 130.2, 130.1, 129.7, 127.6, 126.85, 126.84, 126.2, 125.8, 125.5 (three carbons unresolved). **HRMS (APCI)**: Exact mass calculated for $C_{38}H_{27}$ ($[M+H]^+$): 483.2107, mass found: 483.2112.

3,6-Dibromo-4,5-bis(4-ethylphenyl)phenanthrene (3y)
4-Bromo-3,5-bis(4-ethylphenyl)-6-methylphenanthrene (3y')

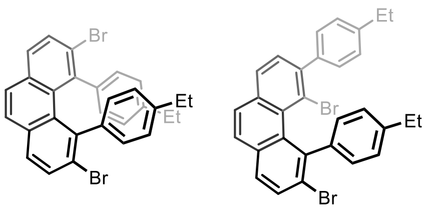

3y 3y'

3y and **3y'** were prepared according to GP3 with **1a** (16 mg, 0.10 mmol) and 1-(bromoethynyl)-4-ethylbenzene (**2y**) (52 mg, 0.25 mmol). Purification by silica gel chromatography (DCM/hexane = 1/5) gave **3y** (11 mg, 2.020 mmol, 20%) as a yellow liquid. **R_f** (DCM/hexane = 1/5): 0.4; ^{1}H NMR (600 MHz, $CDCl_3$) δ 7.76 (d, *J* = 8.4 Hz, 2H), 7.57 (s, 2H), 7.55 (d, *J* = 8.4 Hz, 2H), 6.91 (brs, 2H), 6.82 (brs, 2H), 6.61 (brs, 2H), 6.16 (brs, 2H), 2.70-2.60 (m, 4H), 1.32 (t, *J* = 7.6 Hz, 6H). ^{13}C NMR (151 MHz, $CDCl_3$) δ 143.2, 140.9, 136.9, 134.4, 133.2, 132.9, 131.8, 131.0, 127.6, 127.1, 126.9, 126.5, 123.5, 28.8, 15.9. **HRMS (ESI)**: Exact mass calculated for $C_{30}H_{24}Br_2Na$ ($[M+Na]^+$): 567.0117, mass found: 567.0115.

Purification by silica gel chromatography (DCM/hexane = 1/5) gave **3y'** (10 mg, 0.019 mmol, 19%) as a yellow liquid. **R_f** (DCM/hexane = 1/5): 0.5; ^{1}H NMR (600 MHz, $CDCl_3$) δ 7.91 (d, *J* = 8.4 Hz, 1H), 7.70 (d, *J* = 8.5 Hz, 1H), 7.57 (d, *J* = 9.2 Hz, 1H), 7.42 (d, *J* = 9.2 Hz, 1H), 7.40 (d, *J* = 7.9 Hz, 2H), 7.38 (d, *J* = 9.5 Hz, 1H), 7.35 (d, *J* = 7.8 Hz, 2H), 7.30 (d, *J* = 9.4 Hz, 1H), 7.23 (d, *J* = 8.0 Hz, 2H), 7.19 (d, *J* = 8.0 Hz, 2H), 2.83 (q, *J* = 7.6 Hz, 2H), 2.79 (q, *J* = 7.6 Hz, 2H), 1.39 (t, *J* = 7.6 Hz, 8H), 1.35 (t, *J* = 7.6 Hz, 3H). ^{13}C NMR (151 MHz, $CDCl_3$) δ 144.3, 143.9, 141.1, 140.2, 140.1, 137.6, 134.5, 132.2, 131.3, 130.8, 130.2, 129.7, 129.6, 129.1, 129.0, 128.8, 128.0, 127.8, 126.4, 126.1, 122.8, 28.9, 28.8, 15.8, 15.5 (one carbon unresolved). **HRMS (ESI)**: Exact mass calculated for $C_{30}H_{25}Br_2$ ($[M+H]^+$): 545.0297, mass found: 545.0299.

1,5-Bis(4-ethylphenyl)phenanthrene (5a)

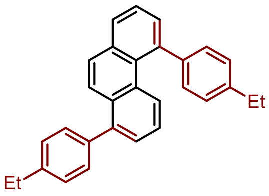

5a was prepared according to GP2 with **1b** (16 mg, 0.10 mmol) and 1-ethyl-4-ethynylbenzene (**2b**) (33 mg, 0.25 mmol) in the mixture of DCM/HFIP (10:1 v/v). Purification by silica gel chromatography (DCM/hexane = 1/5) gave **5a**(16 mg, 0.042 mmol, 42%) as a liquid which was dried by high vacuum at 60 °C for 2 h. **R*f*** (DCM/hexane = 1/5): 0.5; ^{1}H NMR (392 MHz, $CDCl_3$) δ 7.89-7.80 (m, 3H), 7.67 (d, *J* = 9.1 Hz, 1H), 7.59 (t, *J* = 7.5 Hz, 1H), 7.49 (dd, *J* = 7.3, 1.5 Hz, 1H), 7.44-7.38 (m, 3H), 7.40-7.27 (m, 6H), 7.13 (dd, *J* = 8.6, 7.2 Hz, 1H), 2.79 (q, J = 7.5 Hz, 2H), 2.78 (q, J = 7.6 Hz, 1H), 1.36 (t, J = 7.6 Hz, 3H). 1.35 (t, J = 7.6 Hz, 1H). ^{13}C NMR (151 MHz, $CDCl_3$) δ 143.25, 143.16, 142.9, 140.9, 140.6, 138.9, 133.5, 131.5, 131.1, 131.0, 130.4, 129.04, 128.99, 128.6, 128.2, 128.0, 127.9, 127.3, 127.3, 125.9, 125.4, 124.1, 28.8, 28.8, 15.8, 15.7. **HRMS (APCI)**: Exact mass calculated for $C_{30}H_{27}$ ([M+H]$^+$): 387.2107, mass found: 387.2104.

1,5-Bis(4-methoxyphenyl)phenanthrene (5b)

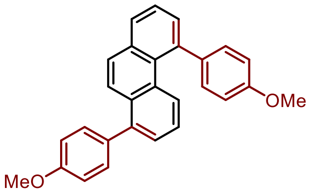

5b was prepared according to GP2 with **1b** (16 mg, 0.10 mmol) and 1-methoxyl-4-ethynylbenzene (**2d**) (33 mg, 0.25 mmol) in the mixture of DCM/HFIP (10:1 v/v). Purification by silica gel chromatography (ethyl acetate/hexane = 1/15) gave **5b** (8.0 mg, 0.021 mmol, 21%) as a solid. **R*f*** (ethyl acetate/hexane = 1/10): 0.3; ^{1}H NMR (600 MHz, $CDCl_3$) δ 7.86 (dt, *J* = 8.7, 0.9 Hz, 1H), 7.84-7.81 (m, 3H), 7.67 (d, *J* = 9.1 Hz, 1H), 7.60-7.55 (m, 1H), 7.47 (dd, *J* = 7.2, 1.4 Hz, 1H), 7.43-7.40 (m, 2H), 7.39-7.35 (m, 3H), 7.15 (dd, *J* = 8.7, 7.1 Hz, 1H), 7.08-6.99 (m, 4H), 3.92 (s, 3H), 3.91 (s, 3H). ^{13}C NMR (151 MHz, $CDCl_3$) δ 159.0, 158.9, 140.5, 140.2, 138.0, 134.0, 133.5, 131.6, 131.5, 131.2, 131.1, 130.2, 129.1, 128.1, 127.8, 127.4, 127.2, 125.9, 125.3, 124.1, 114.6, 113.8, 55.5. **HRMS (APCI)**: Exact mass calculated for $C_{28}H_{23}O_2$ ([M+H]$^+$): 391.1693, mass found: 391.1715.

1,2,5,6-Tetraphenylphenanthrene (5c)

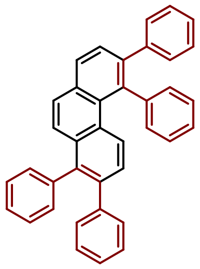

5c was prepared according to GP3 with **1b** (16 mg, 0.10 mmol) and diphenylacetylene (**2x**) (45 mg, 0.25 mmol). Purification by silica gel chromatography (DCM/hexane = 1/20) gave **5c** (11 mg, 0.023 mmol, 23%) as a white solid. If the isolated compound is not pure enough, it can be washed by hexane to give the pure compound. **R*f*** (DCM/hexane = 1/5): 0.3; ^{1}H NMR (600 MHz, $CDCl_3$) δ 7.91 (d, *J* = 8.0 Hz, 1H), 7.67 (d, *J* = 9.1 Hz, 1H), 7.63 (d, *J* = 8.0 Hz, 1H), 7.62-7.59 (m, 2H), 7.34-7.26 (m, 4H), 7.26-7.21 (m, 2H), 7.23-7.15 (m, 7H), 7.16-7.08 (m, 4H), 7.12-7.02 (m, 4H). ^{13}C NMR (151 MHz, $CDCl_3$) δ 142.83, 142.81, 141.8, 141.5, 139.9, 138.5, 138.4, 138.0, 132.9, 132.5, 131.9, 131.2, 130.5, 130.2, 130.1, 129.1, 129.0, 128.7, 128.1, 128.0, 127.6, 127.5, 127.3, 126.8, 126.5, 126.23, 126.15, 126.1. **HRMS (APCI)**: Exact mass calculated for $C_{38}H_{27}$ ($[M+H]^+$): 483.2107, mass found: 483.2106.

2,6-Dibromo-1,5-bis(4-ethylphenyl)phenanthrene (5d)

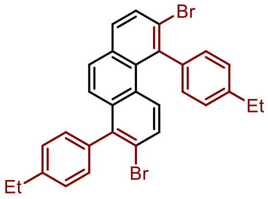

5d was prepared according to GP3 with **1b** (16 mg, 0.10 mmol) and 1-(bromoethynyl)-4-ethylbenzene (**2y**) (52 mg, 0.25 mmol). Purification by silica gel chromatography (DCM/hexane = 1/20) gave **5d** (25 mg, 0.046 mmol, 46%) as a yellow liquid. **R*f*** (DCM/hexane = 1/10): 0.3; ^{1}H NMR (600 MHz, $CDCl_3$) δ 7.91 (d, *J* = 8.4 Hz, 1H), 7.69 (d, *J* = 8.5 Hz, 1H), 7.57 (d, *J* = 9.2 Hz, 1H), 7.43 (dd, *J* = 9.1, 0.7 Hz, 1H), 7.41 (d, *J* = 8.4 Hz, 2H), 7.39 (dd, *J* = 9.4, 0.8 Hz, 1H), 7.35 (d, *J* = 7.8 Hz, 2H), 7.30 (d, *J* = 9.4 Hz, 1H), 7.24 (d, *J* = 8.2 Hz, 2H), 7.19 (d, *J* = 8.2 Hz, 2H), 2.83 (q, *J* = 7.6 Hz, 2H), 2.79 (q, *J* = 7.6 Hz, 2H), 1.39 (t, *J* = 7.6 Hz, 3H), 1.36 (t, *J* = 7.6 Hz, 3H). ^{13}C NMR (151 MHz, $CDCl_3$) δ 144.3, 143.8, 141.1, 140.3, 140.1, 137.6, 134.5, 132.2, 131.3, 130.8, 130.2, 129.71, 129.67, 129.6, 129.1, 129.0, 128.8, 128.0, 127.8, 126.4, 126.1, 122.8, 29.0, 28.8, 15.7, 15.5. **HRMS (EI)**: Exact mass calculated for $C_{30}H_{24}Br_2$ ($[M]^+$): 542.0245, mass found: 542.0245.

1,8-Bis(4-methoxyphenyl)phenanthrene (6b)

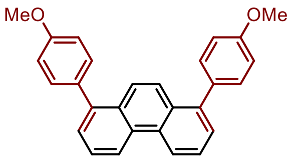

6b was prepared according to GP2 with **1c'** (66 mg, 0.10 mmol) and 1-methoxyl-4-ethynylbenzene (**2d**) (33 mg, 0.25 mmol) in the mixture of DCM/HFIP (10:1 v/v). Purification by silica gel chromatography (ethyl acetate/hexane = 1/15) gave **6b** (4.5 mg, 0.012 mmol, 12%) as a white solid. **R*f*** (ethyl acetate/hexane = 1/10): 0.3; ^{1}H NMR (600 MHz, $CDCl_3$) δ 8.78 (d, *J* = 8.5 Hz, 2H), 7.77 (s, 2H), 7.70 (dd, *J* = 8.4, 7.2 Hz, 2H), 7.54 (dd, *J* = 7.2, 1.1 Hz, 2H), 7.45-7.41 (m, 4H), 7.05-6.99 (m, 4H), 3.89 (s, 6H). ^{13}C NMR (151 MHz, $CDCl_3$) δ 159.1, 140.7, 133.5, 131.4, 131.0, 129.9, 128.1, 126.2, 124.7, 122.3, 113.8, 55.5. **HRMS (APCI)**: Exact mass calculated for $C_{28}H_{23}O_2$ ($[M+H]^+$): 391.1693, mass found: 391.1692.

1,2,7,8-Tetraphenylphenanthrene (6c)

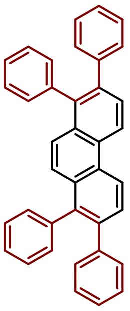

6c was prepared according to GP3 with **1c'** (66 mg, 0.10 mmol) and diphenylacetylene (**2x**) (45 mg, 0.25 mmol). Purification by silica gel chromatography (DCM/hexane = 1/15) gave **6c** (4.6 mg, 0.0095 mmol, 10 %) as a solid. **R*f*** (DCM/hexane = 1/5): 0.4; ^{1}H NMR (600 MHz, $CDCl_3$) δ 8.88 (d, *J* = 8.7 Hz, 2H), 7.78 (d, *J* = 8.6 Hz, 2H), 7.31-7.23 (m, 4H), 7.22-7.14 (m, 16H). ^{13}C NMR (151 MHz, $CDCl_3$) δ 142.0, 139.5, 139.3, 138.6, 131.6, 130.6, 130.3, 129.7, 128.8, 127.9, 127.8, 126.9, 126.4, 125.6, 122.5. **HRMS (APCI)**: Exact mass calculated for $C_{38}H_{27}$ ($[M+H]^+$): 483.2107, mass found: 483.2107.

2,7-Dibromo-1,8-bis(4-ethylphenyl)phenanthrene (6d)

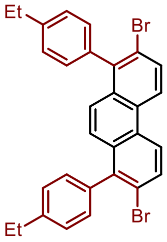

6d was prepared according to GP3 with **1c'** (66 mg, 0.10 mmol) and 1-(bromoethynyl)-4-ethylbenzene (**2y**) (52 mg, 0.25 mmol). Purification by silica gel chromatography (DCM/hexane = 1/20) gave **6d** (12 mg, 0.022 mmol, 22 %) as a solid. **R_f** (DCM/hexane = 1/10): 0.4; ^{1}H NMR (392 MHz, $CDCl_3$) δ 8.57 (d, *J* = 9.0 Hz, 2H), 7.92 (d, *J* = 8.9 Hz, 2H), 7.31 (d, *J* = 8.2 Hz, 4H), 7.28 (s, 2H), 7.18 (d, *J* = 8.1 Hz, 4H), 2.74 (q, *J* = 7.6 Hz, 4H), 1.31 (t, *J* = 7.6 Hz, 6H). ^{13}C NMR (151 MHz, $CDCl_3$) δ 144.0, 140.9, 136.9, 132.2, 130.8, 130.1, 129.2, 127.9, 126.2, 123.7, 123.0, 28.8, 15.6. **HRMS (EI)**: Exact mass calculated for $C_{30}H_{24}Br_2$ ($[M]^+$): 542.0245, mass found: 542.0242.

5. The optimization of scale-up reaction

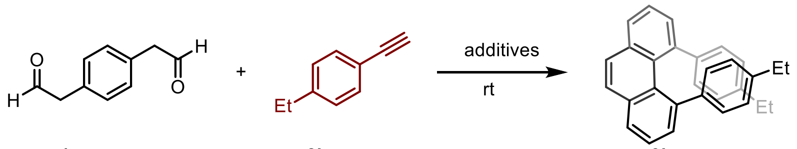

1a, 1.0 eq. **2b**, 2.5 eq. **3b**

**1a** (mmol)	Additive	4Å (pellet)	Time (hr)	Solvent (mL)	**1a** (recovered)	**2b** (recovered)	**1c** nmr yield (isolated yield)
0.10	20 mol% $B(C_6F_5)_3$	100 mg	15	DCM (2.5)	n.d.	0.4 eq.	67% (65%)
2.0	20 mol% $B(C_6F_5)_3$	2.0 g	15	DCM (50)	n.d.	0.9 eq.	55% (55%, 425 mg)
0.20	2.0 eq. $BF_3{\cdot}Et_2O$	200 mg	2	DCM (5.0)	trace	n.d.	46%
0.20	2.0 eq. $BF_3{\cdot}Et_2O$	200 mg	2	DCM/HFIP (5.0/0.5)	trace	n.d.	7%

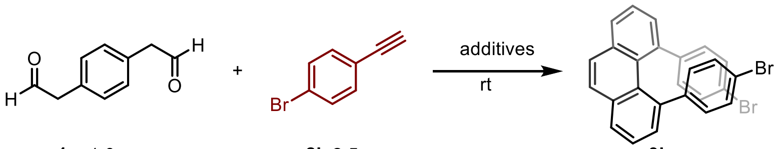

1a, 1.0 eq. **2i**, 2.5 eq. **3i**

**1a** (mmol)	Additive	4Å (pellet)	T (hr)	Solvent (mL)	**1a** (recovered)	**1b** (recovered)	**3i** nmr yield (isolated yield)
0.10	20 mol% $B(C_6F_5)_3$	100 mg	12	DCM/HFIP (2.5/0.25)	n.d.	1.3 eq.	44% (37%)
0.80	20 mol% $B(C_6F_5)_3$	100 mg	12	DCM/HFIP (20/2.0)	trace	1.6 eq.	21%
1.5	2.0 eq. $BF_3{\cdot}Et_2O$	1.5 g	3	DCM/HFIP (25/2.5)	n.d.	n.d.	41% (41%, 300 mg)

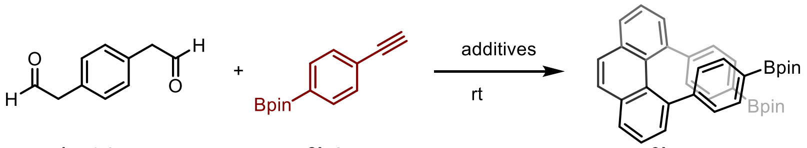

1a, 1.0 eq. **2l**, 2.5 eq. **3l**

**1a** (mmol)	Additive	4Å (pellet)	T (hr)	Solvent (mL)	**1a** (recovered)	**2l** (recovered)	**3i** nmr yield (isolated yield)
0.10	20 mol% $B(C_6F_5)_3$	100 mg	15	DCM/HFIP (2.5/0.25)	n.d.	1.5eq	25% (21%)
0.20	2.0 eq. $BF_3{\cdot}Et_2O$	200 mg	2	DCM (5.0)	trace	1.1eq	15%
2.0	2.0 eq. $BF_3{\cdot}Et_2O$	2.0 g	2	DCM/HFIP (50/5.0)	trace	n.d.	25%

General procedure (when $BF_3{\cdot}Et_2O$ was used as an additive):

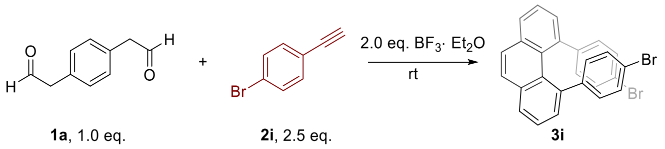

A dry Schlenk tube equipped with a magnetic stir bar and a septum was charged with activated molecular sieve, diacetaldehyde compound **1a** and **2i** under argon atmosphere. DCM and HFIP were then added. After that, $BF_3{\cdot}Et_2O$ was added dropwise at room temperature. After stirring at room temperature for 2 h, the reaction mixture was quenched by water. And then the resulting mixture was extracted with DCM three times. The organic extracts were washed with water, brine, dried over $MgSO_4$, and concentrated. The residue was then purified by silica gel column chromatography directly.

6. The optimization of asymmetric bisannulation

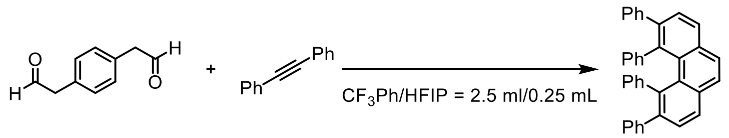

1a (0.1 mmol) **2x** (0.25 mmol) **3x**

Entry	Additives	°C (hours)	1a	2x	3x
**1**	50 mol% DL-10-Camphorsulfonic acid	rt (15)	55% recovered	2.4 equiv. recovered	n.d.
**2**	1.5 equiv. BINOL-phosphoric acid	rt (12) to 90 °C (3)	58% recovered	2.2 equiv. recovered	n.d.
**3**	1.5 equiv. H_3PO4	rt (12) to 90 °C (3)	30% recovered	2.5 equiv. recovered	n.d.

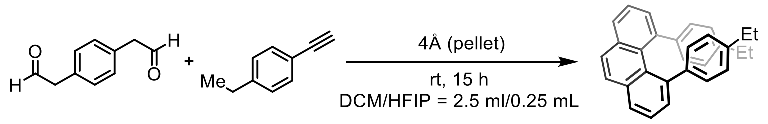

1a (0.1 mmol) **2b** (0.25 mmol) **3b**

Entry	Additives	1a	2b	3b
**1**	20 mol% DL-10-Camphorsulfonic acid	75% recovered	1.6 equiv. recovered	n.d.
**2**	1.5 equiv. BINOL-phosphoric acid	75% recovered	1.9 equiv. recovered	n.d.
**3**	1.5 equiv. H_3PO4	77% recovered	1.3 equiv. recovered	n.d.

7. The formation of regioisomers (3y and 3y')

Due to the α-phenyl-beta-halovinyl cation is more stable than β-phenyl-alfa-halovinyl cation (by 17.5 kcal/mol, reference *J. Org. Chem.* **2006**, *71*, 9643-9650, figure 1A). The first annulation of 1,4-benzenediacetaldehyde and bromide-substituted phenylacetylene gave the intermediate naphthalene with high regioselectivity, which was corresponding with the reaction of phenylacetaldehyde with bromide-substituted phenylacetylene to give 2-bromo-1-phenylnaphthalene with excellent regioselectivity (figure 1B). However, due to the steric encumbrance of the phenyl substituent in intermediate naphthalene under the second annulation, and the bromide is smaller than phenyl group, so the regioisomers (**3y** and **3y'**) was formed (figure 1C). Hence, without the steric encumbrance of the phenyl substituent, 1,3-benzenediacetaldehyde (**1b**) and 1,2-benzenediacetaldehyde disilyl acetal (**1c'**) reacted with bromide-substituted phenylacetylene can give the corresponding products with excellent regioselectivity (figure 1D).

Figure 1:

A) Gibbs free energies of halovinyl cation

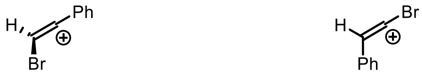

⊿G = -199.7 kcal/mol* alfa-phenyl-beta-halovinyl cation

⊿G = -182.2 kcal/mol* beta-phenyl-alfa-halovinyl cation

*Gibbs free energies relative to those of the corresponding 1-phenyl-haloacetylene
J. Org. Chem. **2006**, *71*, 9643-9650.

B) The reaction of phenylacetaldehyde with bromide-substituted phenylacetylene

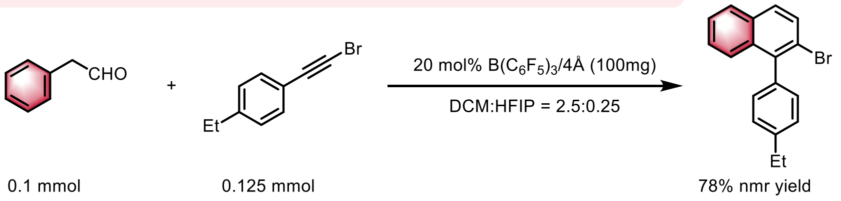

C) The reaction of 1,4-benzenediacetaldehyde and bromide-substituted phenylacetylene

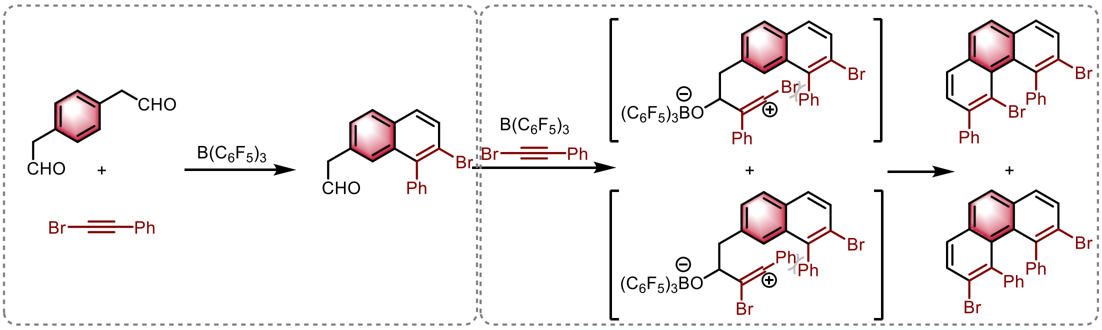

First annulation with high regioselectivity

Second annulation with low regioselectivity

D) The reaction of 1,3-benzenediacetaldehyde (1b) and 1,2-benzenediacetaldehyde disilyl acetal (1c') reacted with bromide-substituted phenylacetylene

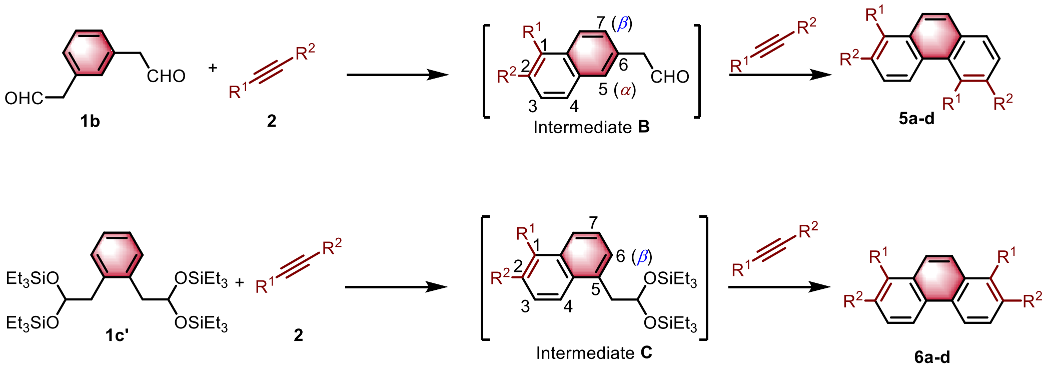

8. The detailed mechanism

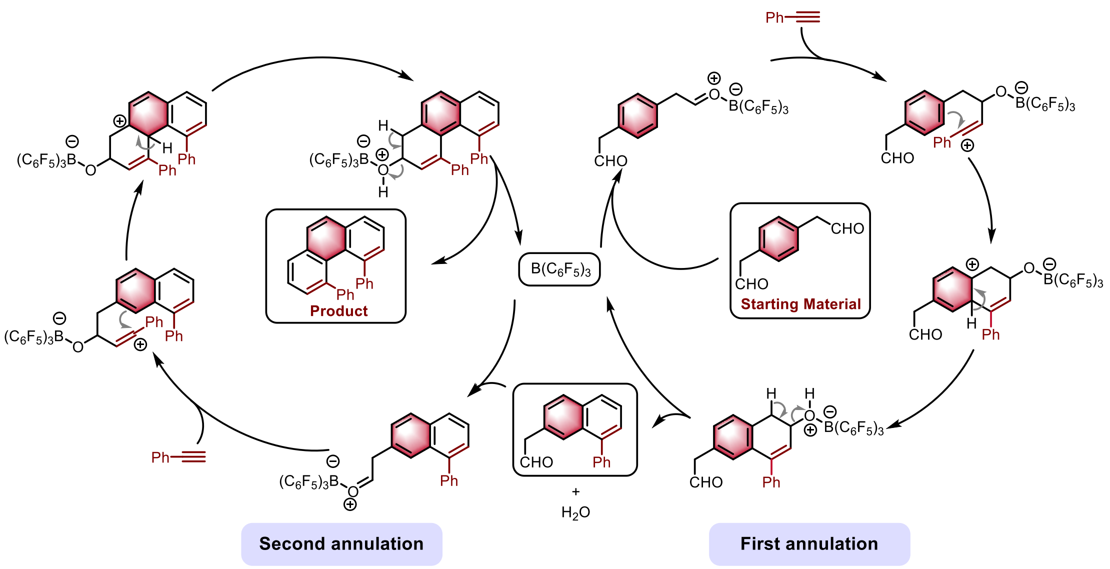

9. The synthesis of diphosphine ligand[2]

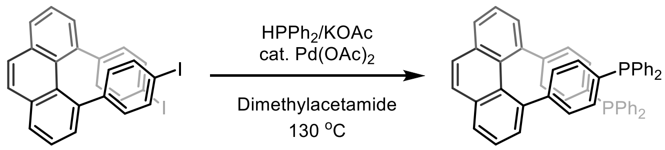

A dry and argon-flushed Schlenk tube, equipped with a magnetic stir bar and a septum, was charged with potassium acetate (150 μmol, 14.7 mg, 3.0 equiv). Subsequently, the Schlenk tube was dried over a heatgun (around 300 °C) for 5 min under high vacuum. After that, the Schlenk tube was purged with argon at ambient temperature. Then **3j** (50 μmol, 29 mg, 1.0 equiv), *N*,*N*-dimethylacetamide (0.5 mL), and diphenylphosphine (150 μmol, 28 mg, 26 μL, 3.0 equiv) were added under argon atmosphere. Subsequently, a solution of *N*,*N*-dimethylacetamide (0.5 mL) containing palladium acetate (0.56 mg, 5 mol%) was added. The solution turned red and was immediately placed in an oil bath at 130 °C for 4h. The reaction mixture was allowed to cool down to room temperature. Water (10 mL) was added and the aqueous phase was extracted with dichloromethane (3*20 mL). The combined organic layers were dried over sodium sulfate. Evaporation of the solvent followed by column chromatography on silica gel with hexane/DCM (2:1) afforded the corresponding product **3z** (15 mg, 0.022 mmol, 44 %) as a white solid. **R*f*** (DCM/hexane = 1/2): 0.3; ^{1}H NMR (600 MHz, $CDCl_3$) δ 7.74 (dd, *J* = 7.8, 1.3 Hz, 2H), 7.67 (s, 2H), 7.53-7.44 (m, 2H), 7.37-7.31 (m, 14H), 7.30-7.27 (m, 6H), 7.09 (dd, *J* = 7.3, 1.3 Hz, 2H), 6.96 (t, *J* = 8.2 Hz, 2H), 6.86 (t, *J* = 7.5 Hz, 2H), 6.56 (d, *J* = 7.8 Hz, 2H), 6.47 (d, *J* = 8.2 Hz, 2H). ^{31}P NMR (243 MHz, $CDCl_3$) δ −4.92. ^{13}C NMR (151 MHz, $CDCl_3$) δ 144.0, 141.4, 137.7 (d, $J_{C\text{-}P}$ = 10.8 Hz), 137.5 (d, $J_{C\text{-}P}$ = 10.6 Hz), 135.1 (d, $J_{C\text{-}P}$ = 18.8 Hz), 134.7, 134.5 (d, $J_{C\text{-}P}$ = 10.5 Hz), 133.7 (d, $J_{C\text{-}P}$ = 19.4 Hz), 133.6 (d, *J* = 19.4 Hz), 133.1 (d, $J_{C\text{-}P}$ = 23.2 Hz), 129.5, 128.9-128.7 (m), 128.6-128.5 (m), 127.7, 127.2, 126.8. **HRMS (APCI)**: Exact mass calculated for $C_{50}H_{37}P_2$ ($[M+H]^+$): 699.2365, mass found: 699.2361.

The synthesis of binuclear Pd complex[3]

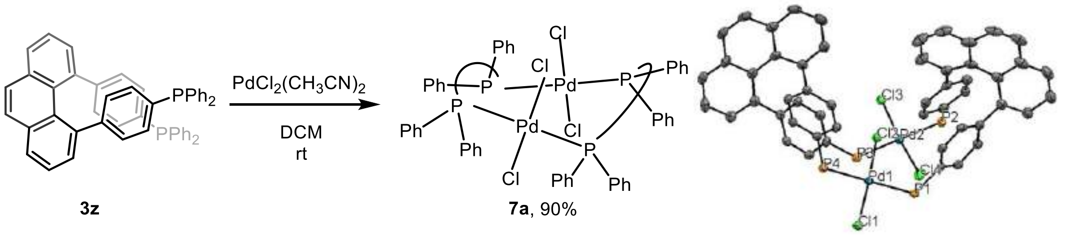

[a]Hydrogen atoms, phenyl groups, and solvent molecular are omitted for clarity.

To a Schlenk tube were added [$PdCl_2(CH_3CN)_2$] (3.7 mg, 14 μmol), **3z** (10 mg, 14 μmol) and CH_2Cl_2 (4 mL) under a stream of argon. The yellow solution was stirred for 12 h. Evaporation of the solvent followed by washed with hexane three times afforded the corresponding product **3x** (11 mg, 6.3 μmol, 90 %) as a yellow solid. Suitable crystals for diffraction study were grown from the solution of **7a** with mixed solvents of $CHCl_3$ and methanol at room temperature. ^{1}H NMR (600 MHz, $CDCl_3$) δ 7.99-7.92 (m, 4H), 7.90-7.82 (m, 4H), 7.78-7.72 (m, 2H), 7.67 (dd, *J* = 7.8, 1.2 Hz, 2H), 7.63 (s, 2H), 7.52 (t, *J* = 7.3 Hz, 4H), 7.48-7.42 (m, 2H), 7.40-7.35 (m, 4H), 7.22 (t, *J* = 8.6 Hz, 4H), 7.18-7.13 (m, 2H), 6.97 (dd, *J* = 7.3, 1.2 Hz, 2H), 6.69 (d, *J* = 9.2 Hz, 2H), 6.58 (d, *J* = 9.7 Hz, 2H). ^{31}P NMR (243 MHz, $CDCl_3$) δ 24.04. ^{13}C NMR (151 MHz, $CDCl_3$) δ 145.3, 140.8, 137.0 (t, J_{C-P} = 9.5 Hz), 135.6 (t, J_{C-P} = 7.0 Hz), 134.5, 134.3 (t, J_{C-P} = 6.5 Hz), 134.1-134.0 (m), 132.3-132.0 (m), 130.9-130.2 (m), 129.9, 128.8 (t, J_{C-P} = 5.5 Hz), 128.1 (t, J_{C-P} = 5.7 Hz), 127.8-127.6 (m), 127.5, 127.3, 127.2 (t, J_{C-P} = 14.0 Hz), 127.0-126.8 (m). **HRMS (ESI)**: Exact mass calculated for $C_{100}H_{72}Cl_3P_4Pd_2$ ([M−Cl]$^+$): 1713.1714, mass found: 1713.1723.

10. Variable temperature ^{1}H NMR spectrum (3m and 3q) and 1D NOE spectrum (3q)

a) High temperature ^{1}H NMR spectrum of 3m in 1,1,2,2-tetrachloroethane-d_2

3m was studied by variable temperature ^{1}H NMR spectroscopy between 25 and 110 °C. The broad peaks resulting from the bay region aryl and methyl groups gradually sharpen as a function of increasing temperature.

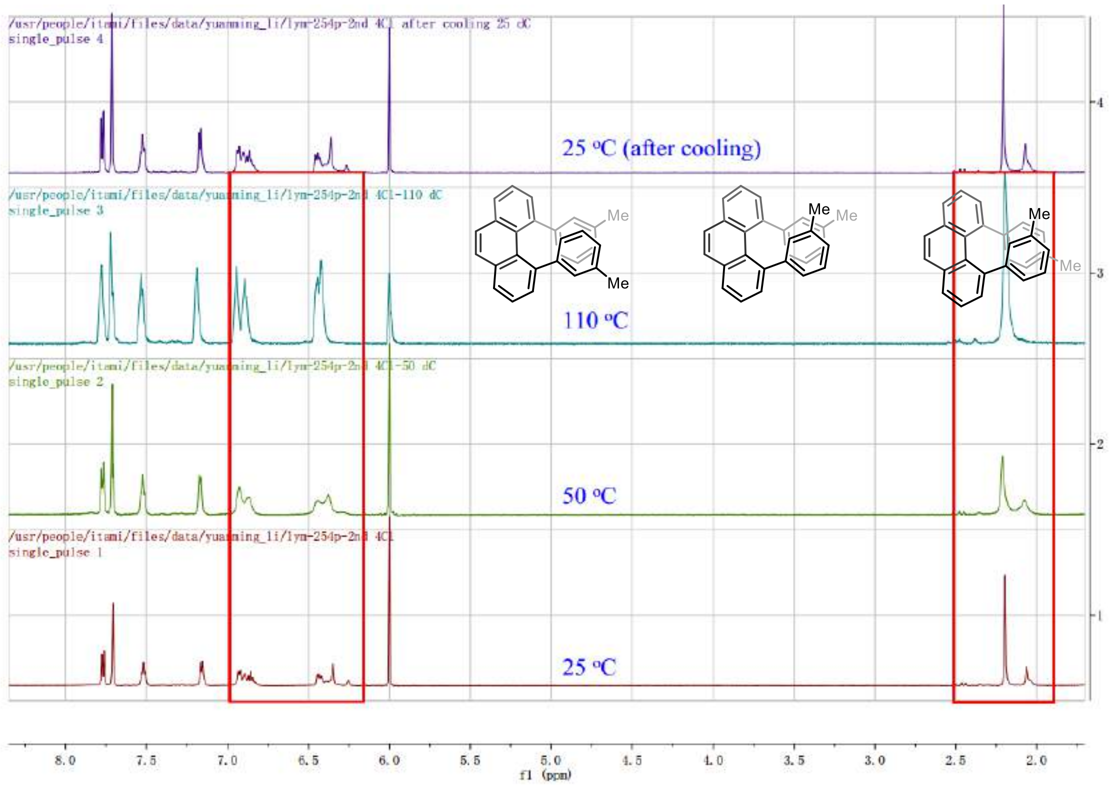

b) High temperature ^{1}H NMR spectrum of 3q in 1,1,2,2-tetrachloroethane-d_2

3q was studied by variable temperature ^{1}H NMR spectroscopy between 25 and 145 °C. The broad peaks resulting from the bay region aryl and methyl groups gradually sharpen as a function of increasing temperature.

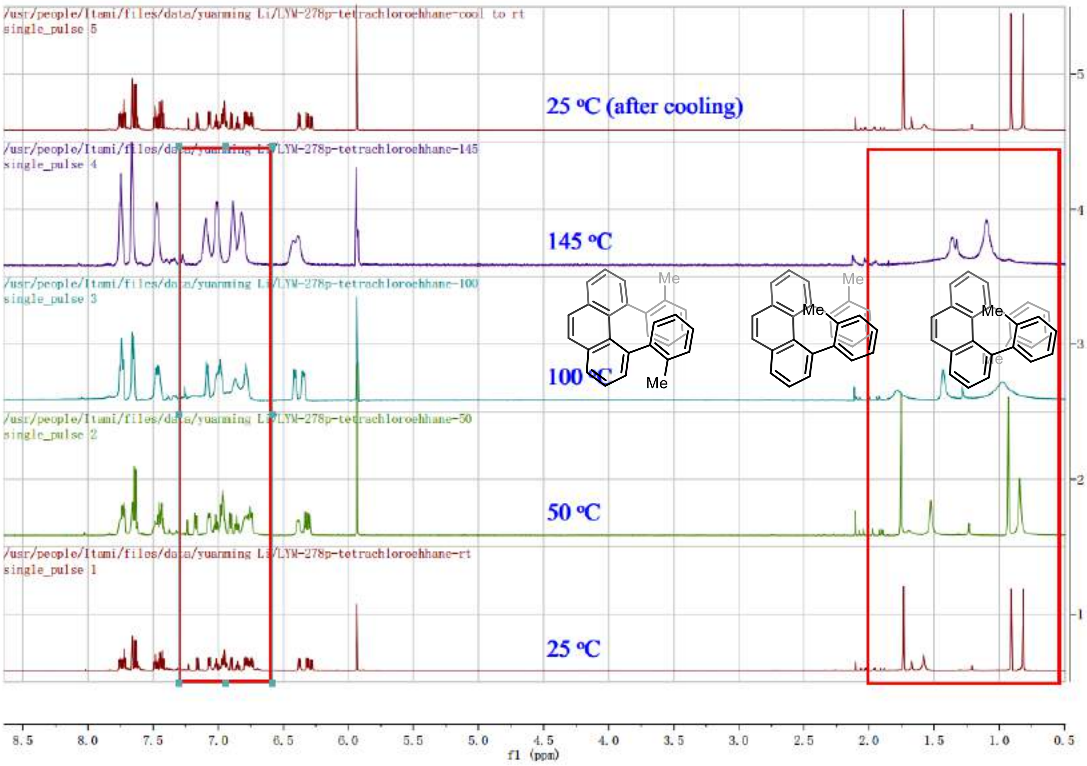

c) 1D DPFGSE NOE experiment of 3q in 1,1,2,2-tetrachloroethane-*d*₂

Furthermore, irradiation of the peak of **3q** using a 1D DPFGSE NOE experiment resulted in a spectrum which shows negative peaks, implying chemical exchange and thus the existence of rotamers in **3q**.[4]

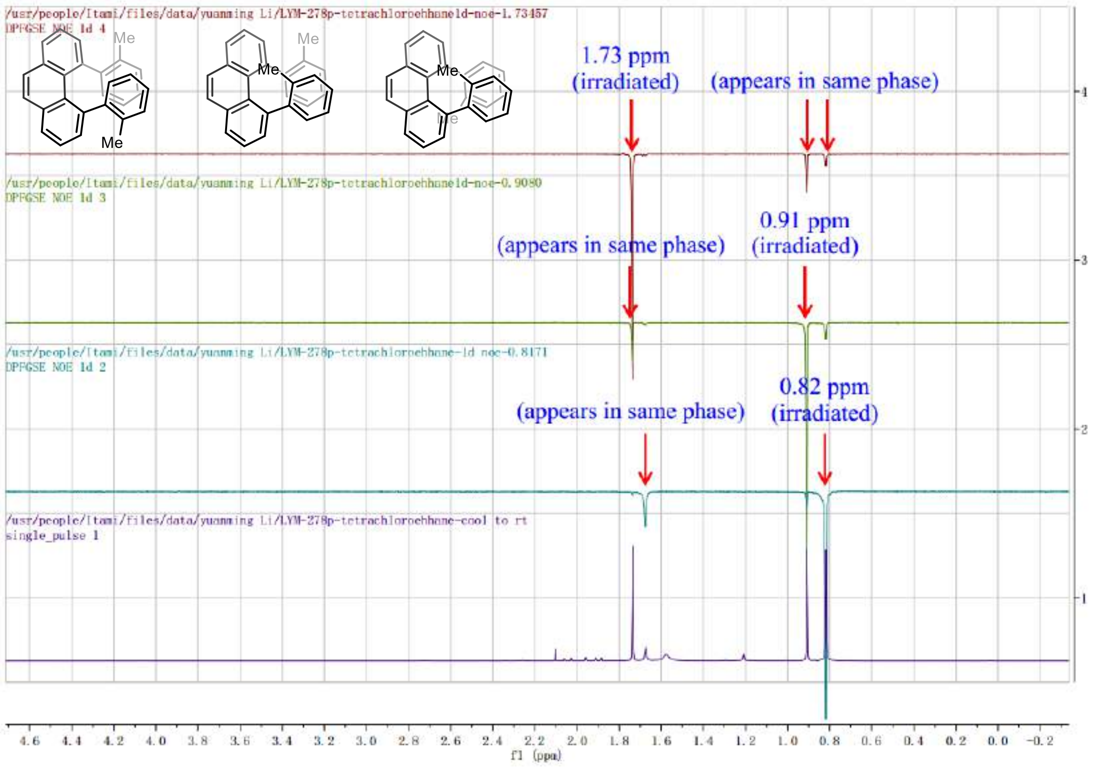

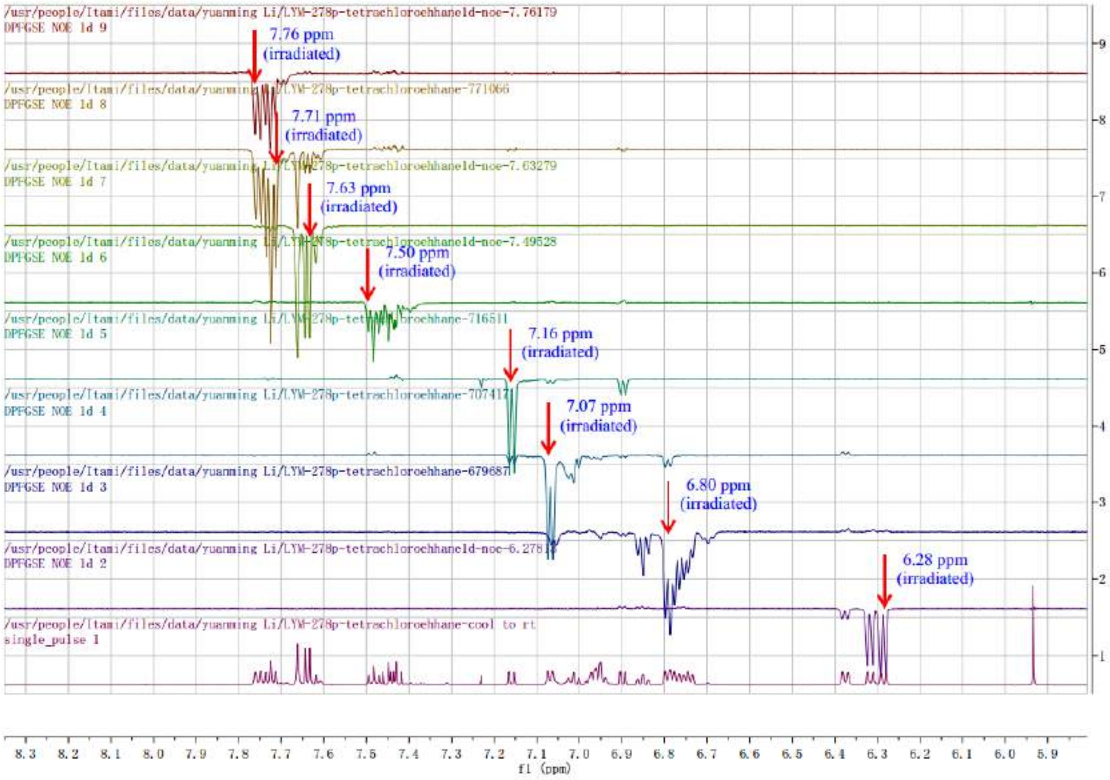

11. Reaction of α-aryl-substituted diacetaldehyde and diketones with alkynes

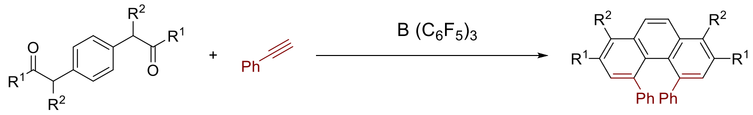

1 2a 8

1	8	yield[b]
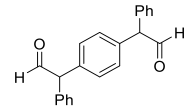	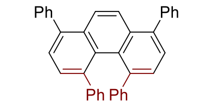	**8a** (n.d.)
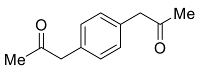	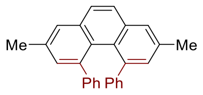	**8b** (n.d.)
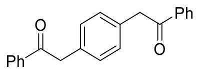	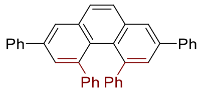	**8c** (n.d.)

[a]Reaction conditions: **1** (0.10 mmol), and **2a** (0.25 mmol) in DCM (2.5 mL). [b]NMR Yield. n.d. = not detected.

12. Mulliken charges for the intermediate A

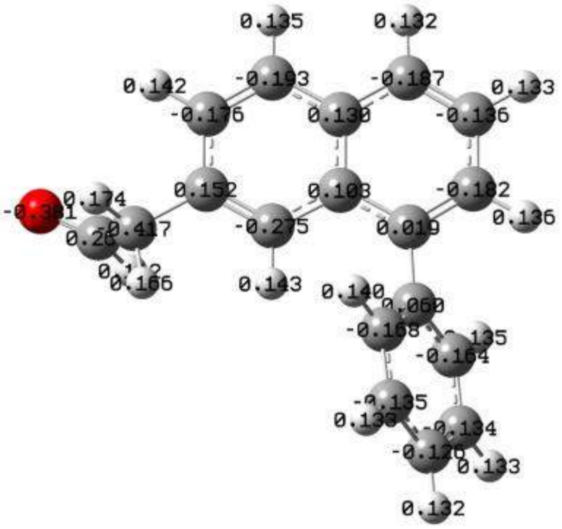

The intermediate **A** was subjected to geometry optimization using B3LYP and the 6-31G+d basis set using Gaussian 16. M. J. Frisch, G. W. Trucks, H. B. Schlegel, G. E. Scuseria, M. A. Robb, J. R. Cheeseman, G. Scalmani, V. Barone, G. A. Petersson, H. Nakatsuji, X. Li, M. Caricato, A. V. Marenich, J. Bloino, B. G. Janesko, R. Gomperts, B. Mennucci, H. P. Hratchian, J. V. Ortiz, A. F. Izmaylov, J. L. Sonnenberg, D. Williams-Young, F. Ding, F. Lipparini, F. Egidi, J. Goings, B. Peng, A. Petrone, T. Henderson, D. Ranasinghe, V. G. Zakrzewski, J. Gao, N. Rega, G. Zheng, W. Liang, M. Hada, M. Ehara, K. Toyota, R. Fukuda, J. Hasegawa, M. Ishida, T. Nakajima, Y. Honda, O. Kitao, H. Nakai, T. Vreven, K. Throssell, J. A. Montgomery, Jr., J. E. Peralta, F. Ogliaro, M. J. Bearpark, J. J. Heyd, E. N. Brothers, K. N. Kudin, V. N. Staroverov, T. A. Keith, R. Kobayashi, J. Normand, K. Raghavachari, A. P. Rendell, J. C. Burant, S. S. Iyengar, J. Tomasi, M. Cossi, J. M. Millam, M. Klene, C. Adamo, R. Cammi, J. W. Ochterski, R. L. Martin, K. Morokuma, O. Farkas, J. B. Foresman, and D. J. Fox, Gaussian, Inc., Wallingford CT, 2016.

13. X-ray data

Details of the crystal data and a summary of the intensity data collection parameters for **3c, 3x** and **7a** are listed in Table S1. In each case, a suitable crystal was mounted with mineral oil on a glass fiber and transferred to the goniometer of a Rigaku PILATUS diffractometer. Graphite-monochromated Mo Kα radiation (λ = 0.71075 Å) was used. The structures were solved by direct methods with (SIR-97)[5] or SHELXT and refined by full-matrix least-squares techniques against F^2 (SHELXL-2013/4)[6] with Yadokari-XG program.[7] The intensities were corrected for Lorentz and polarization effects. The non-hydrogen atoms were refined anisotropically. Hydrogen atoms were placed using AFIX instructions.

Table S1. Crystallographic data and structure refinement details for **3c, 3x, 7a**.

	**3c**	**3x**	**7a·$CHCl_3$**
CCDC deposition No.	1865671	1865672	1865673
formula	$C_{34}H_{34}$	$C_{38}H_{26}$	$C_{101}H_{73}Cl_7P_4Pd_2$
fw	442.61	482.59	1871.42
T (K)	123(2)	123(2)	123(2)
λ (Å)	0.71075	0.71073	0.71073
cryst syst	Monoclinic	Monoclinic	Triclinic
space group	$C2/c$	$P2_1/n$	P-1
a (Å)	14.0303(19)	8.9305(2)	14.5697(2)
b (Å)	23.979(4)	16.0425(3)	14.7169(2)
c (Å)	7.3735(11)	18.2272(3)	23.7580(4)
α (deg)	90	90	92.5220(10)
β (deg)	94.442(4)	101.3021(19)	105.129(2)
γ (deg)	90	90	116.309(2)
V (Å^3)	2473.2(6)	2560.73(9)	4332.56(14)
Z	4	4	2
D_{calc} (g/cm^3)	1.189	1.252	1.435
μ (mm^{-1})	0.067	0.071	0.753
F(000)	952	1016	1900
cryst size (mm)	0.15 × 0.10 × 0.10	0.15 × 0.10 × 0.10	0.10 × 0.10 × 0.10
θ range (deg)	3.146 to 24.999	2.279 to 24.998	2.604 to 24.999
reflns collected	13626	22958	47820
indep reflns/R_{int}	2188/0.0268	4498/0.0266	15188/0.0341
params	157	343	1027
GOF on F^2	1.027	1.041	1.044
R_1, wR_2 [I>2σ(I)]	0.0337, 0.0872	0.0318, 0.0814	0.0437, 0.1188
R_1, wR_2 (all data)	0.0413, 0.0913	0.0371, 0.0847	0.0561, 0.1260

14. The resolution of 3b and 3x

Chiral resolution of **3b and 3x** were performed on a Shimadzu HPLC Prominence chromatograph equipped with a CHIRALPAK® ID-3 column (eluent: n-hexane/dichloromethane = 95:5, 1.0 mL·s^{-1}, Detector: PDA Ch2 315nm 4nm).

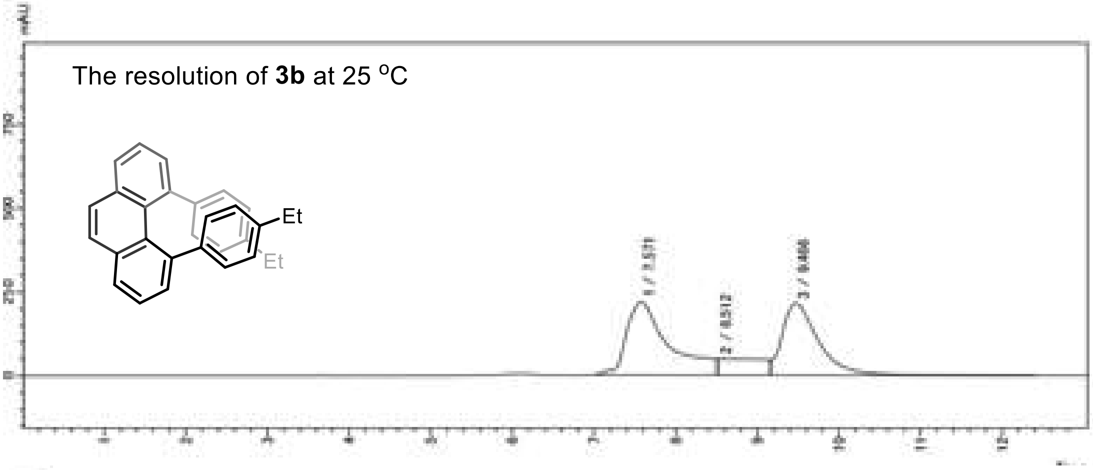

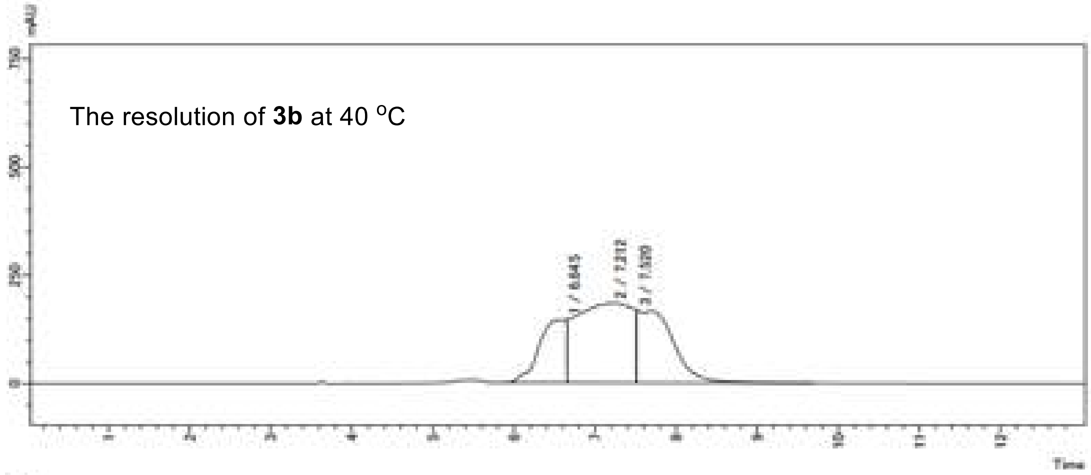

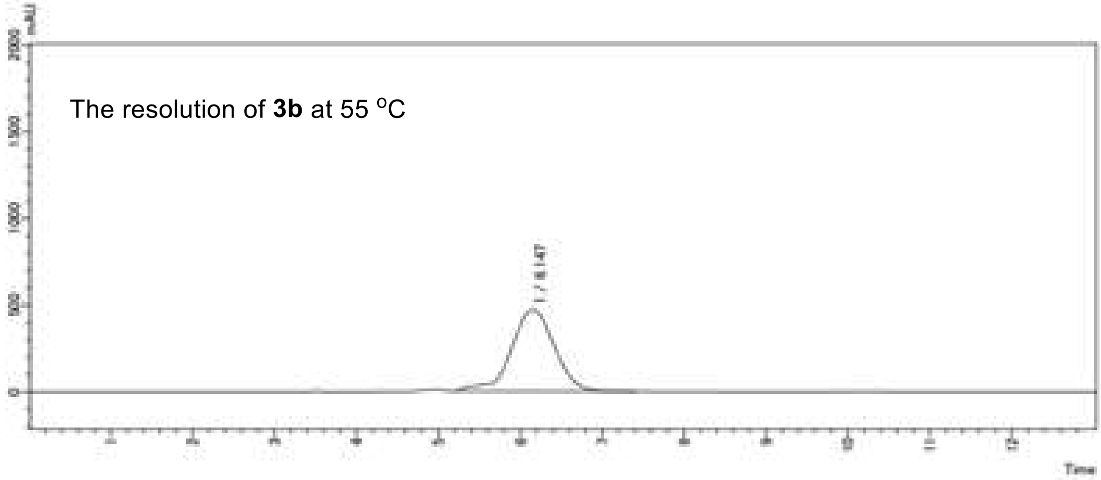

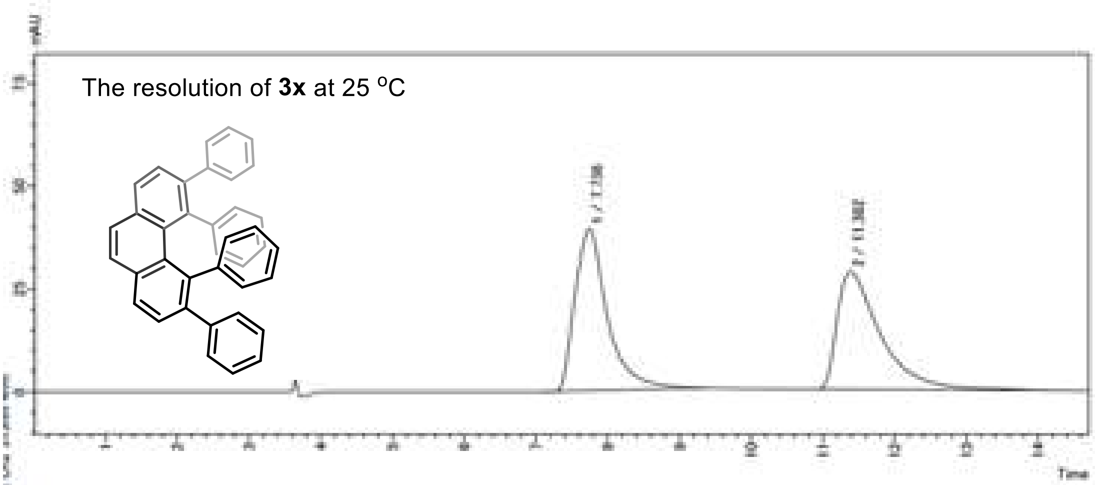
The resolution of **3x** at 25 °C

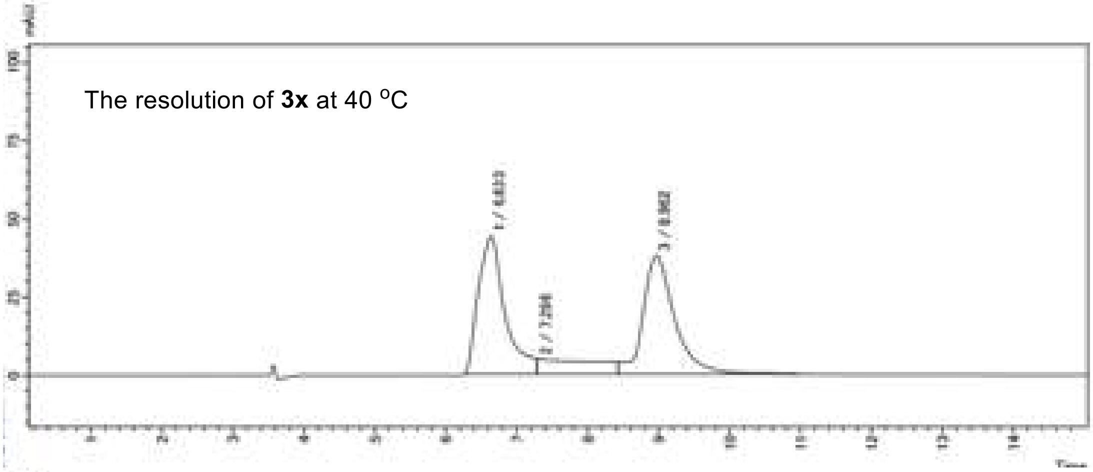
The resolution of **3x** at 40 °C

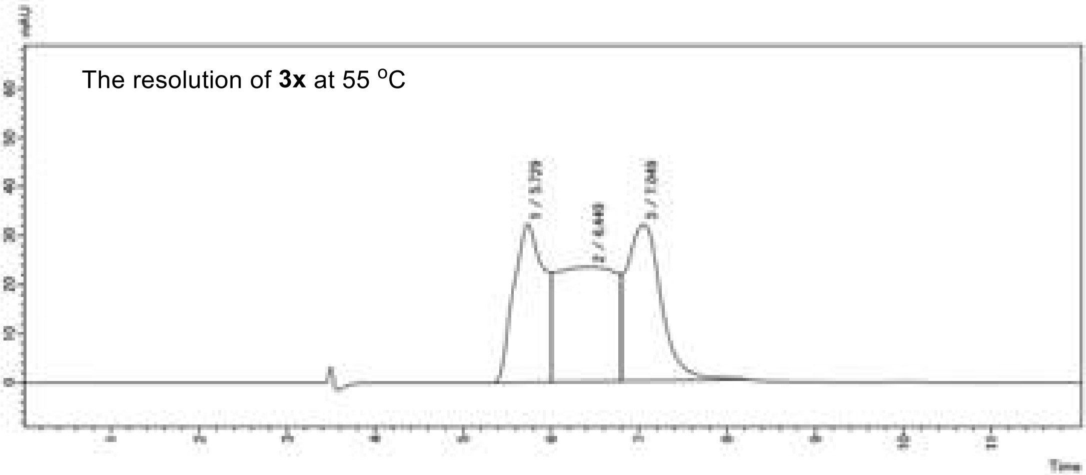
The resolution of **3x** at 55 °C

15. Free energy of activation for racemization of 3n

The activation barrier for racemization of **3n** was determined according to the literature methods[8] ($\Delta G^{\ddagger}$ = 126 kJ mol^{-1} at 100 °C (373.15 K) in toluene). HPLC analysis was conducted on a Shimadzu Prominence 2000 instrument equipped with equipped with a CHIRALPAK® IE column (eluent: *n*-hexane/dichloromethane = 95:5, 1.0 mL·s^{-1}, 25 °C, Detector: PDA Ch2 315nm 4nm).

Table: Erosion in *e.r.* of **3n** over time at 100 °C (373.15 K) in toluene

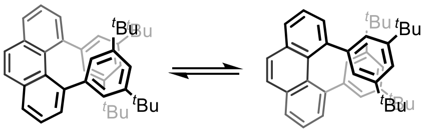

3n (P) **3n** (M)

Time/h	Time/s	% major ent.	% minor ent.	$\ln\frac{(1+[\min]/[\mathrm{maj}])}{(1-[\min]/[\mathrm{maj}])}$
0	0	96.7	3.3	0.0683
1	3600	91.0	9.0	0.198
6	21600	71.2	28.8	0.858
24	86400	52.3	47.7	3.079
33	118800	51.7	49.3	3.740

Figure: $\ln\frac{(1+[\min]/[\mathrm{maj}])}{(1-[\min]/[\mathrm{maj}])}$ vs. time

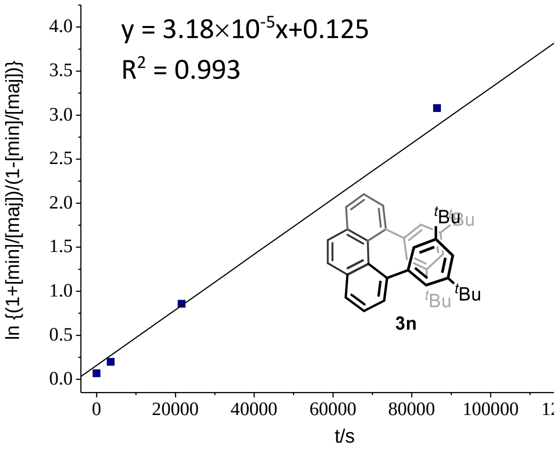

$$\ln\frac{(1+[min]/[maj])}{(1-[min]/[maj])} = 2k_{\mathrm{ent}}t + C$$

Therefore, k_{ent} = ½ slope = 1.59×10^{-5} s^{-1}

$k_{\mathrm{rac}} = 2k_{\mathrm{ent}}$ = 3.18 $\times10^{-5}$ s^{-1}

$t_{1/2}^{\mathrm{rac}} = \ln(2)/k_{\mathrm{rac}}$ = 6.05 h

Employing the Eyring equation:

$$\Delta G^{\ddagger} = -RT\ \ln\frac{\mathrm{kent}\times h}{\mathrm{kB}\times\mathrm{T}} = 126\ \mathrm{kJ\ mol^{-1}}$$

The HPLC analysis spectra

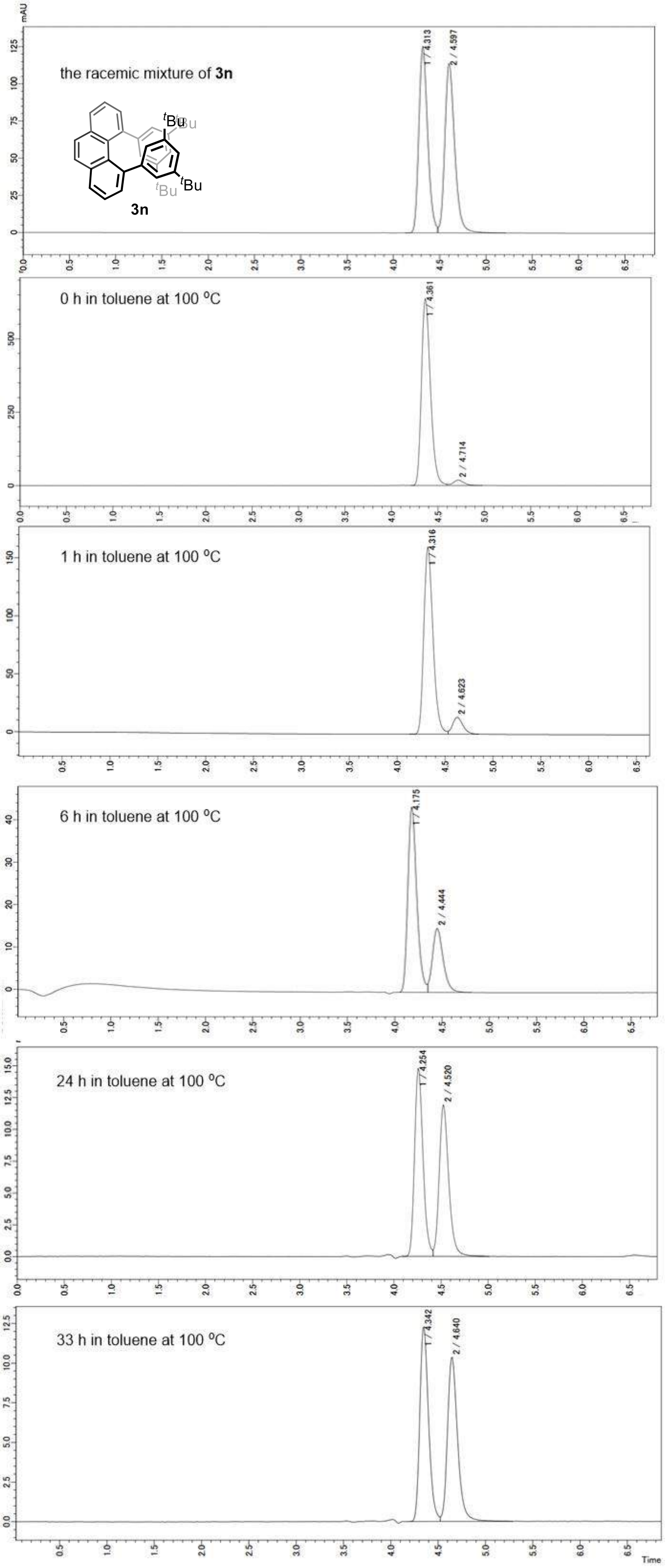

16. Erosion in e.r. of 3n at 70 °C in hexane and 85 °C in 1,2-dichloroethane

HPLC analysis was conducted on a Shimadzu Prominence 2000 instrument equipped with equipped with a CHIRALPAK® IE column (eluent: *n*-hexane/DCM = 95:5, 1.0 mL·s^{-1}, 25 °C, Detector: PDA Ch2 315nm 4nm).

Table: Erosion in *e.r.* of **3n**

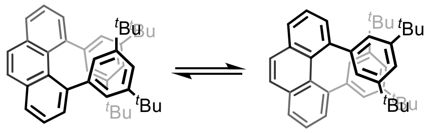

3n (P) **3n** (M)

Enantiomer 2 at 70 °C	0 h	5 h	28 h
in hexane	72.3:27.7	71.4:28.6	69.3:30.7
Enantiomer 1 at 85 °C	0 h	1 h	3 h
in DCE	96.8:3.2	95.8:4.2	94.0:6.0

The HPLC analysis spectra

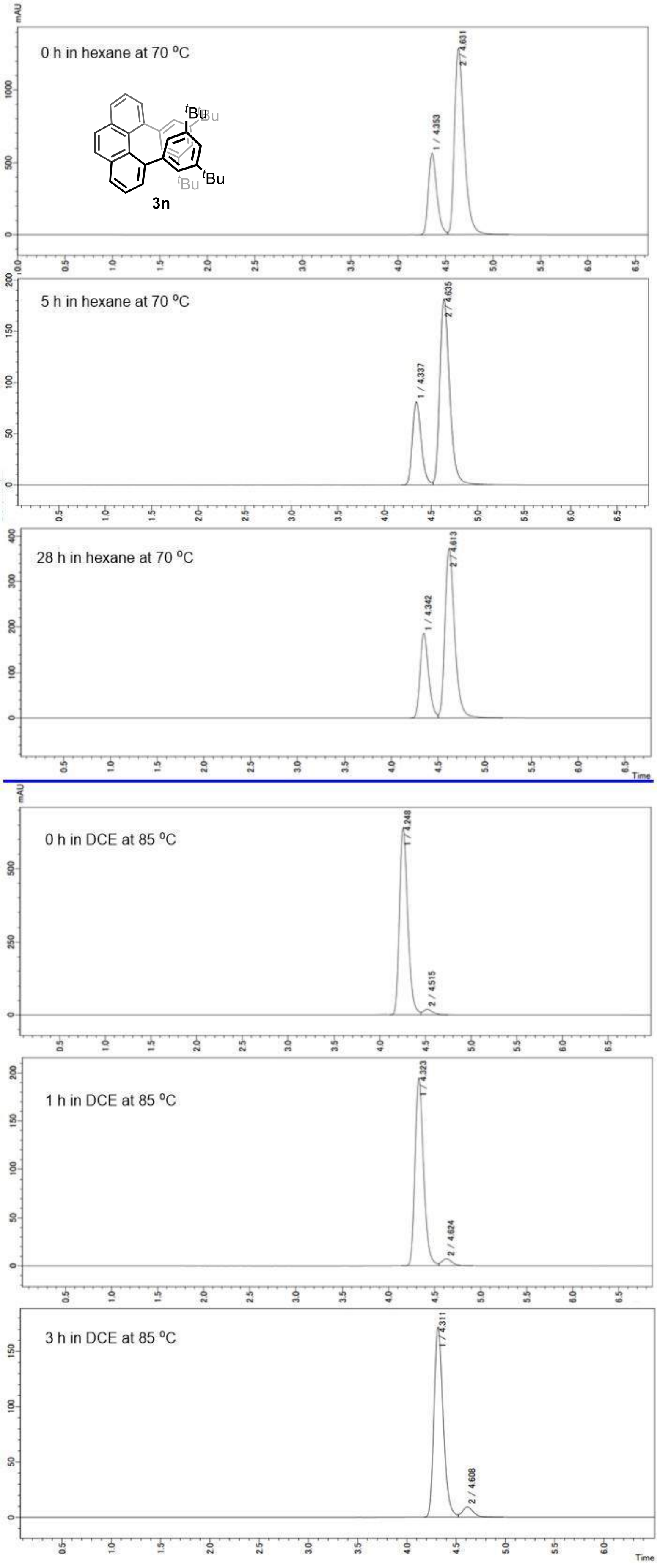

17. The optical rotation and CD spectrum of 3n.

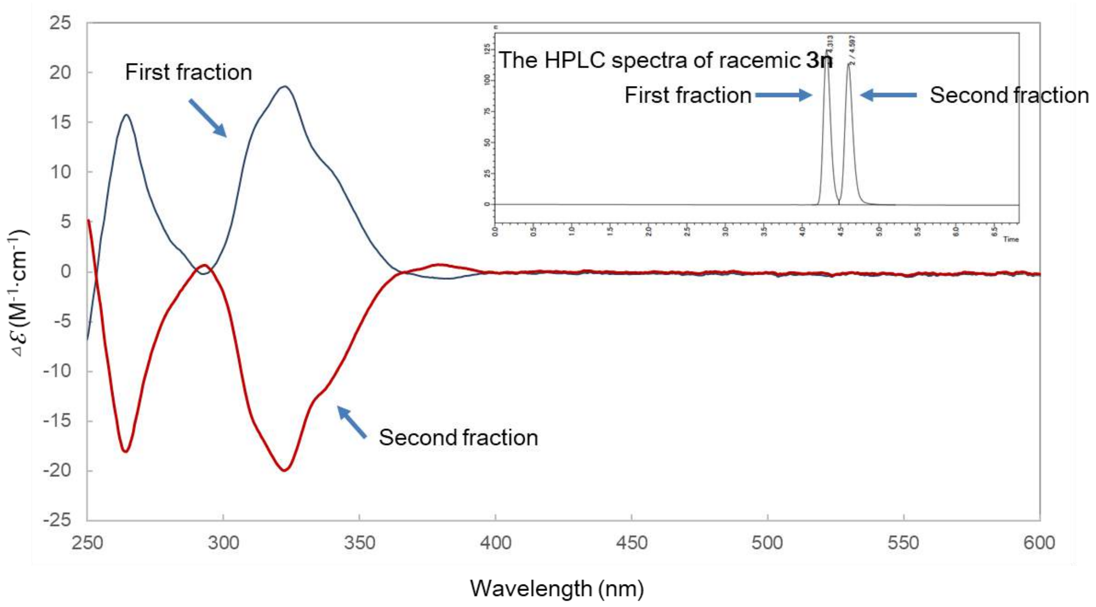

Figure 2: CD spectra of enantiopure-**3n1** (blue line, the first fraction) and enantiopure-**3n2** (red line, the second fraction) (CHIRALPAK® IE column, eluent: *n*-hexane/DCM = 95:5, 1.0 mL·s^{-1}, 25 °C, Detector: PDA Ch2 315nm 4nm).

Second fraction (ee: 97.6:2.4): $[\alpha]_D^{20}$ = -2026 (c = 0.000533 in CH_2Cl_2).

18. References

1 J. Carreras, G. Gopakumar, L. Gu, A. Gimeno, P. Linowski, J. Petuškova, W. Thiel and M. Alcarazo, *J. Am. Chem. Soc.*, 2013, **135**, 18815-18823.

2 L. Bonnafoux, R. Gramage-Doria, F. Colobert and F. R. Leroux, *Chem. Eur. J.*, 2011, **17**, 11008-11016.

3 X. Wang, P. Guo, Z. Han, X. Wang, Z. Wang and K. Ding, *J. Am. Chem. Soc.*, 2014, **136**, 405-411.

4 D. X. Hu, P. Grice and S. V. Ley, *J. Org. Chem.*, 2012, **77**, 5198-5202.

5 A. Altomare, M. C. Burla, M. Camalli, G. L. Cascarano, C. Giacovazzo, A. Guagliardi, A. G. G. Moliterni, G. Polidori and R. Spagna, *J. Appl. Crystallogr.*, 1999, **32**, 115-119.

6 G. Sheldrick, *Acta Crystallogr. A*, 2008, **64**, 112-122.

7 (a) Yadokari-XG, *Software for Crystal Structure Analyses*, K. Wakita 2001; (b) K. Chizuko, A. Shigehisa and K. Eunsang, *J. Cryst. Soc. Jpn.*, 2009, **51**, 218-224.

8 J. D. Jolliffe, R. J. Armstrong and M. D. Smith, *Nat. Chem.*, 2017, **9**, 558-562.

19. NMR spectra of all unknown compounds

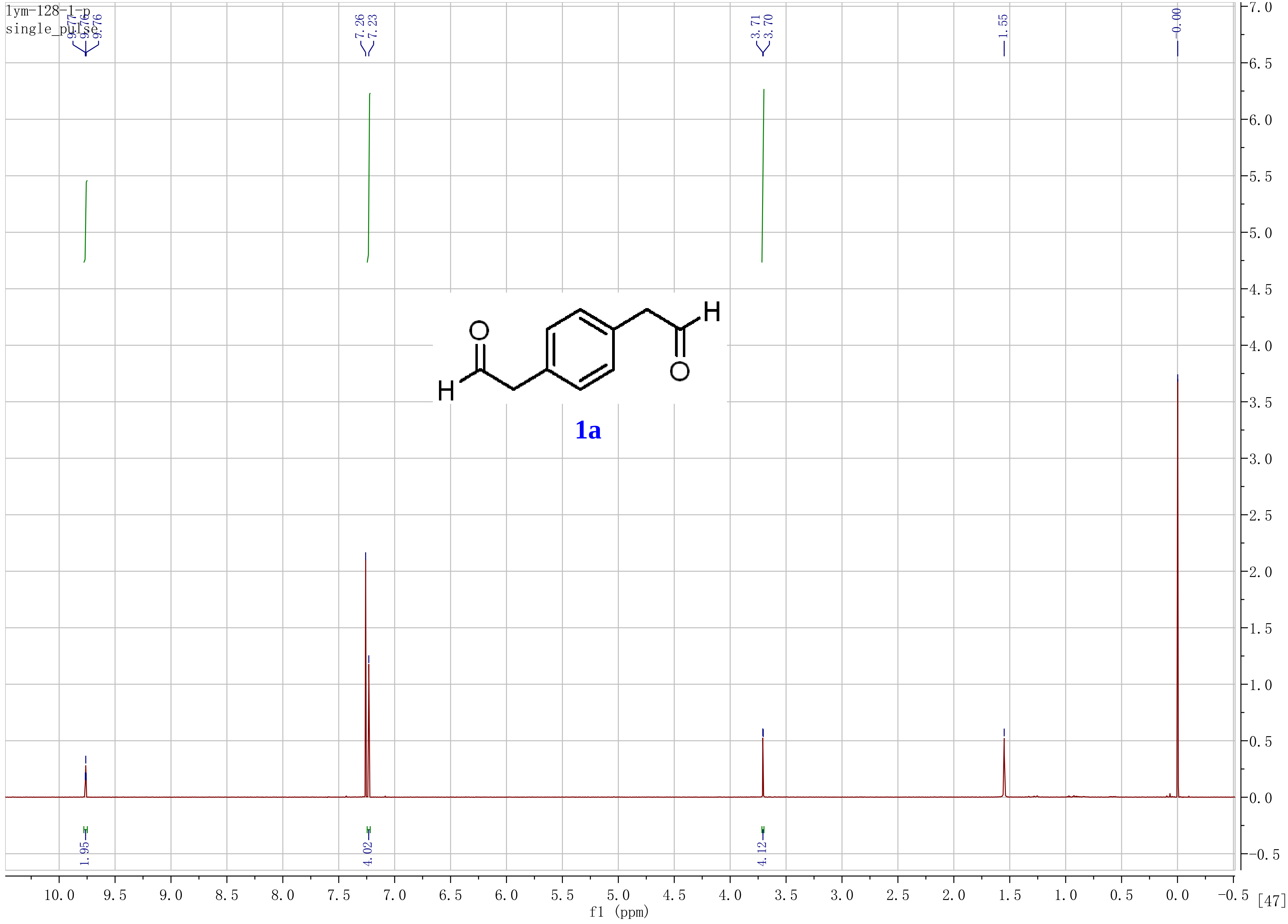
lym-128-1-p
single_pulse
9.777
9.776
9.776
7.26
7.23
3.71
3.70
1.55
-0.00
1a
1.95
4.02
4.12
f1 (ppm)

lym-340p
single pulse decoupled gated NOE

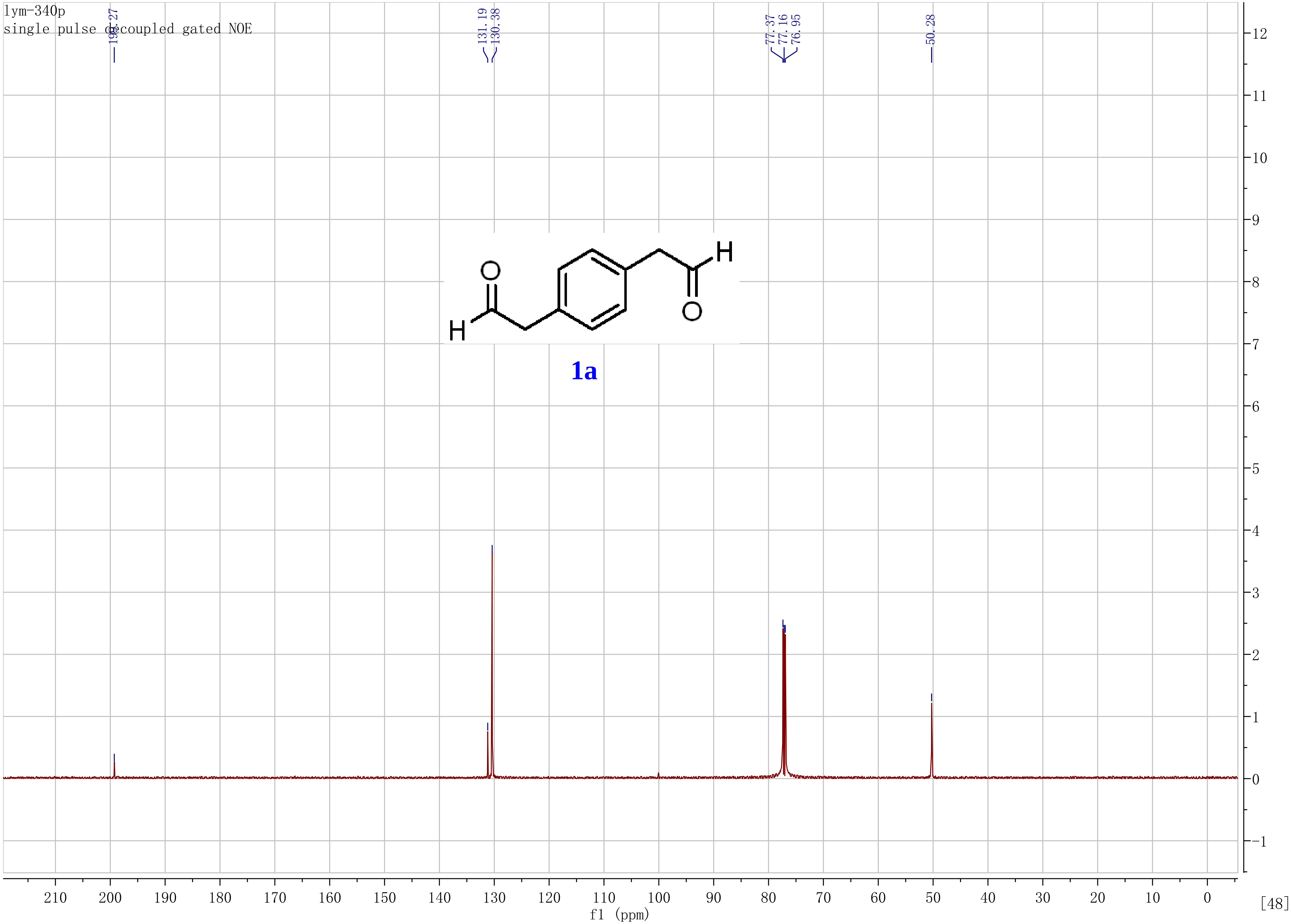

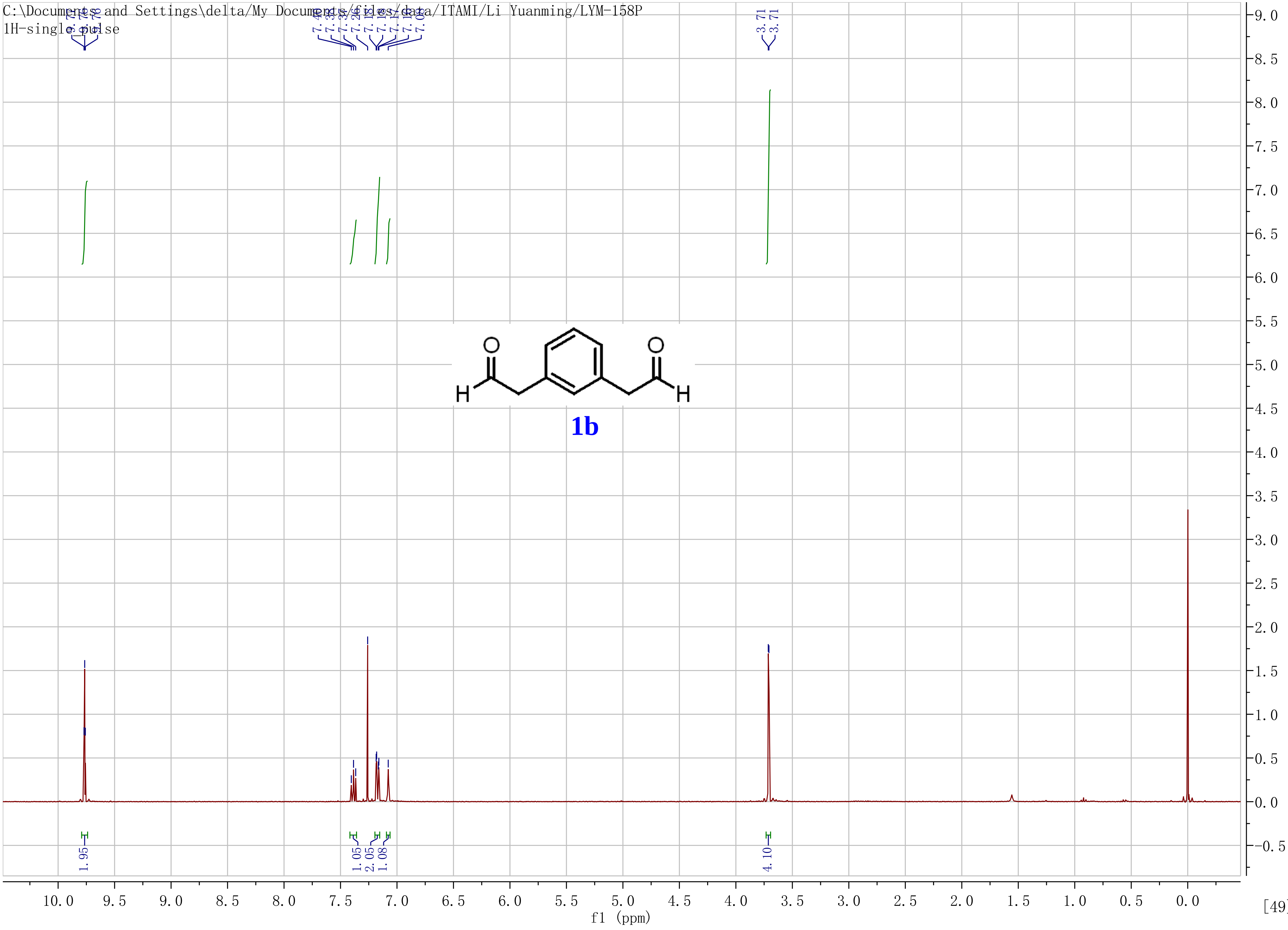
C:\Documents and Settings\delta/My Documents/files/data/ITAMI/Li Yuanming/LYM-158P
1H-single pulse
3.71
3.71
1b
1.95
1.05
2.05
1.08
4.10
f1 (ppm)

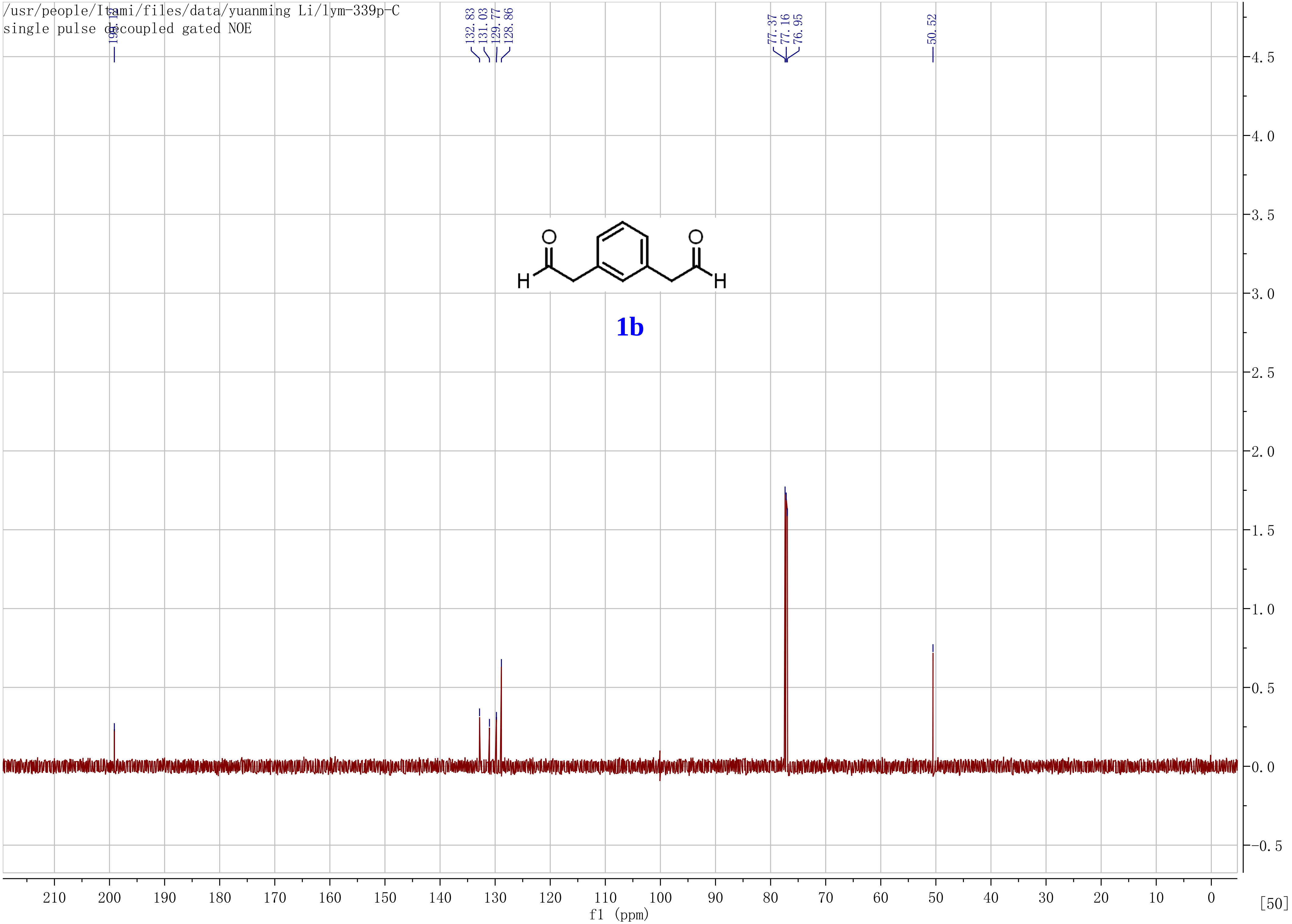

/usr/people/Itami/files/data/yuanming Li/lym-339p-C
single pulse decoupled gated NOE
199.12
132.83
131.03
129.77
128.86
77.37
77.16
76.95
50.52
1b
H
O
f1 (ppm)
[50]

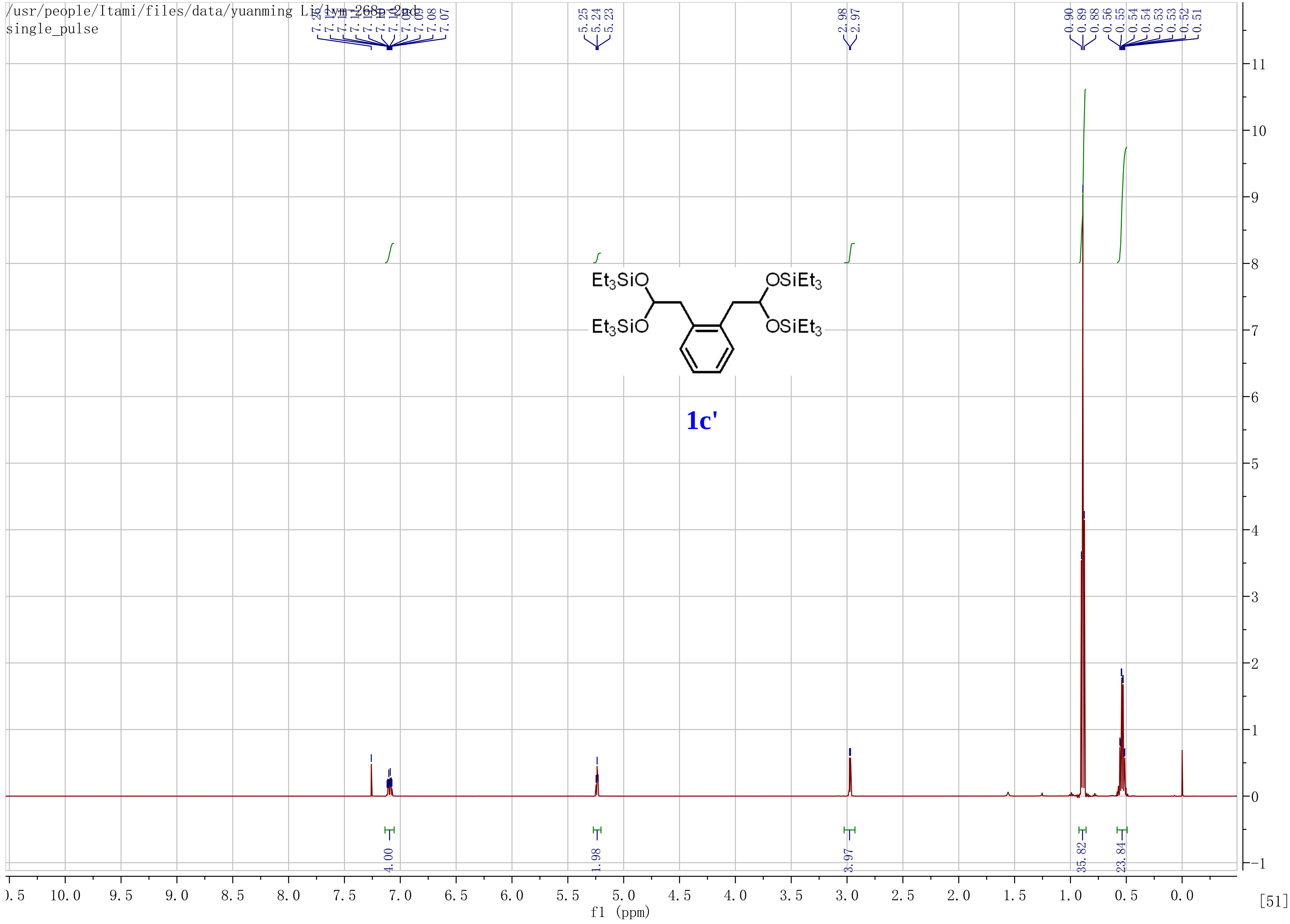
/usr/people/Itami/files/data/yuanming Li/lym-268p-2nd
single_pulse
7.08
7.07
5.25
5.24
5.23
2.98
2.97
0.90
0.89
0.88
0.56
0.55
0.54
0.54
0.53
0.53
0.52
0.51
Et3SiO
Et3SiO
OSiEt3
OSiEt3
1c'
4.00
1.98
3.97
35.82
23.84
f1 (ppm)

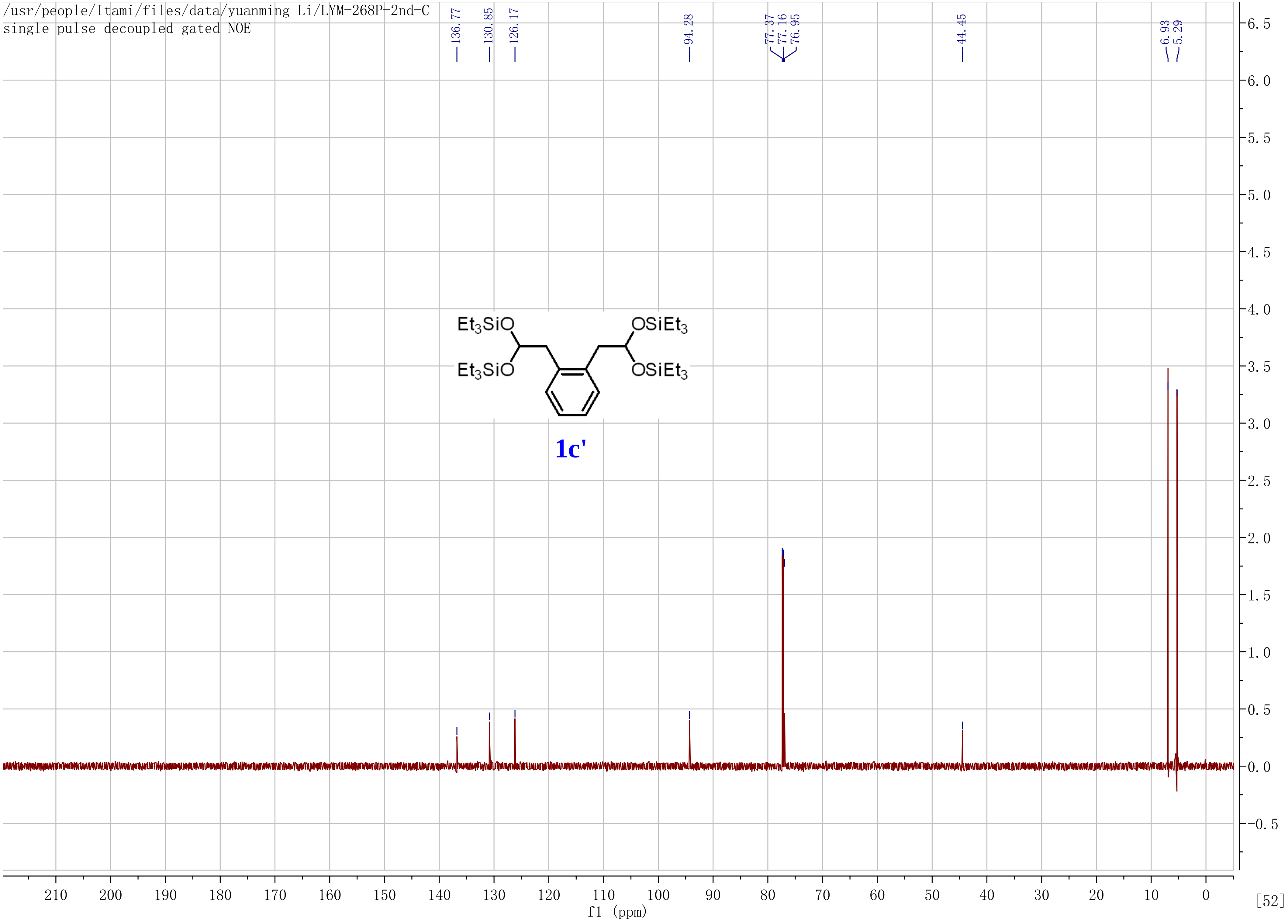

/usr/people/Itami/files/data/yuanming Li/LYM-268P-2nd-C
single pulse decoupled gated NOE
136.77
130.85
126.17
94.28
77.37
77.16
76.95
44.45
6.93
5.29
Et3SiO
Et3SiO
OSiEt3
OSiEt3
1c'
f1 (ppm)

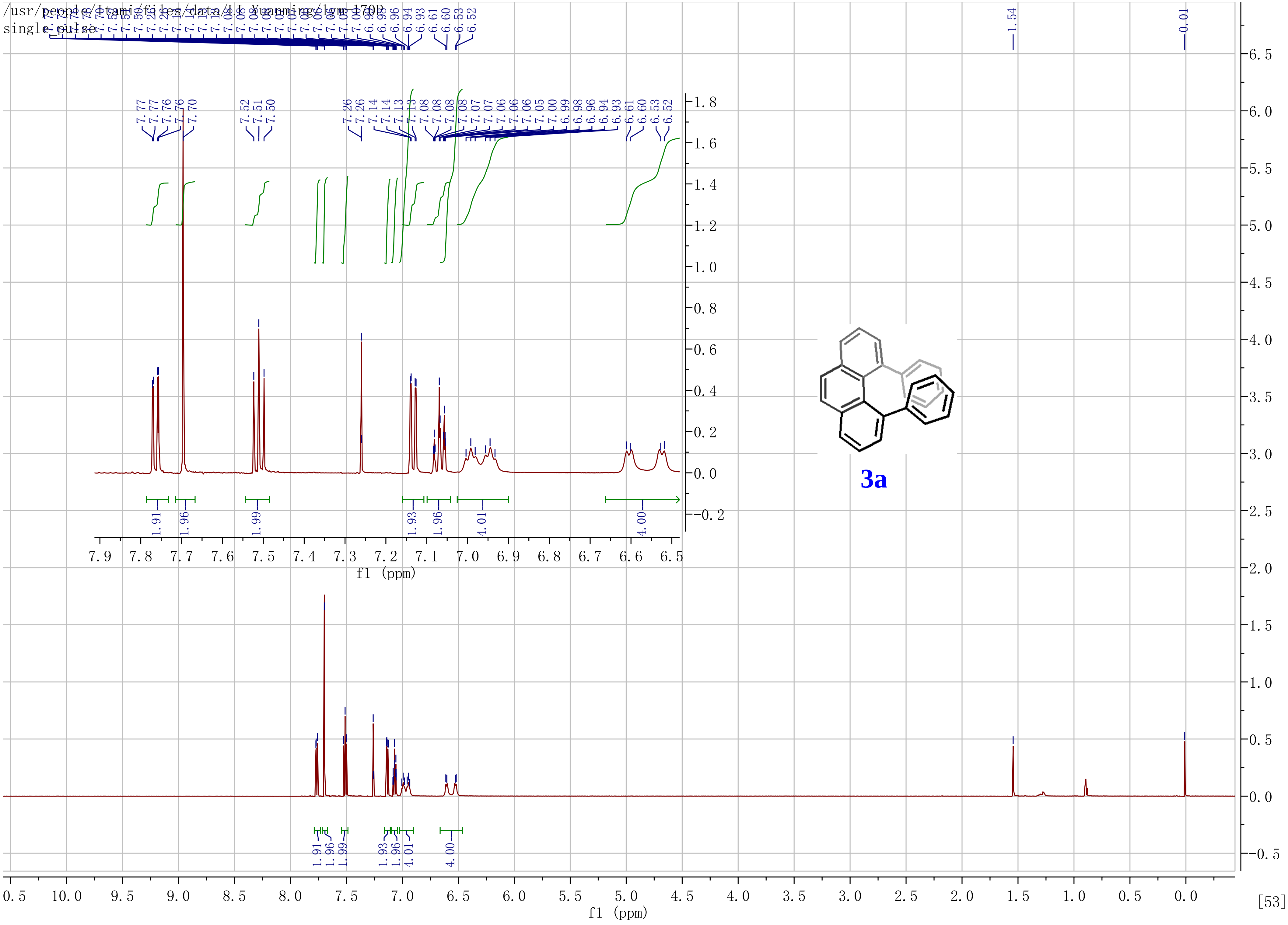

/usr/people/Itami/files/data/Li Yuanming/lym-170D
single pulse
7.77
7.77
7.76
7.76
7.70
7.52
7.51
7.50
7.26
7.26
7.14
7.14
7.13
7.13
7.08
7.08
7.08
7.08
7.07
7.07
7.06
7.06
7.05
7.00
6.99
6.98
6.96
6.94
6.93
6.61
6.60
6.53
6.52
1.54
0.01
1.91
1.96
1.99
1.93
1.96
4.01
4.00
f1 (ppm)

3a

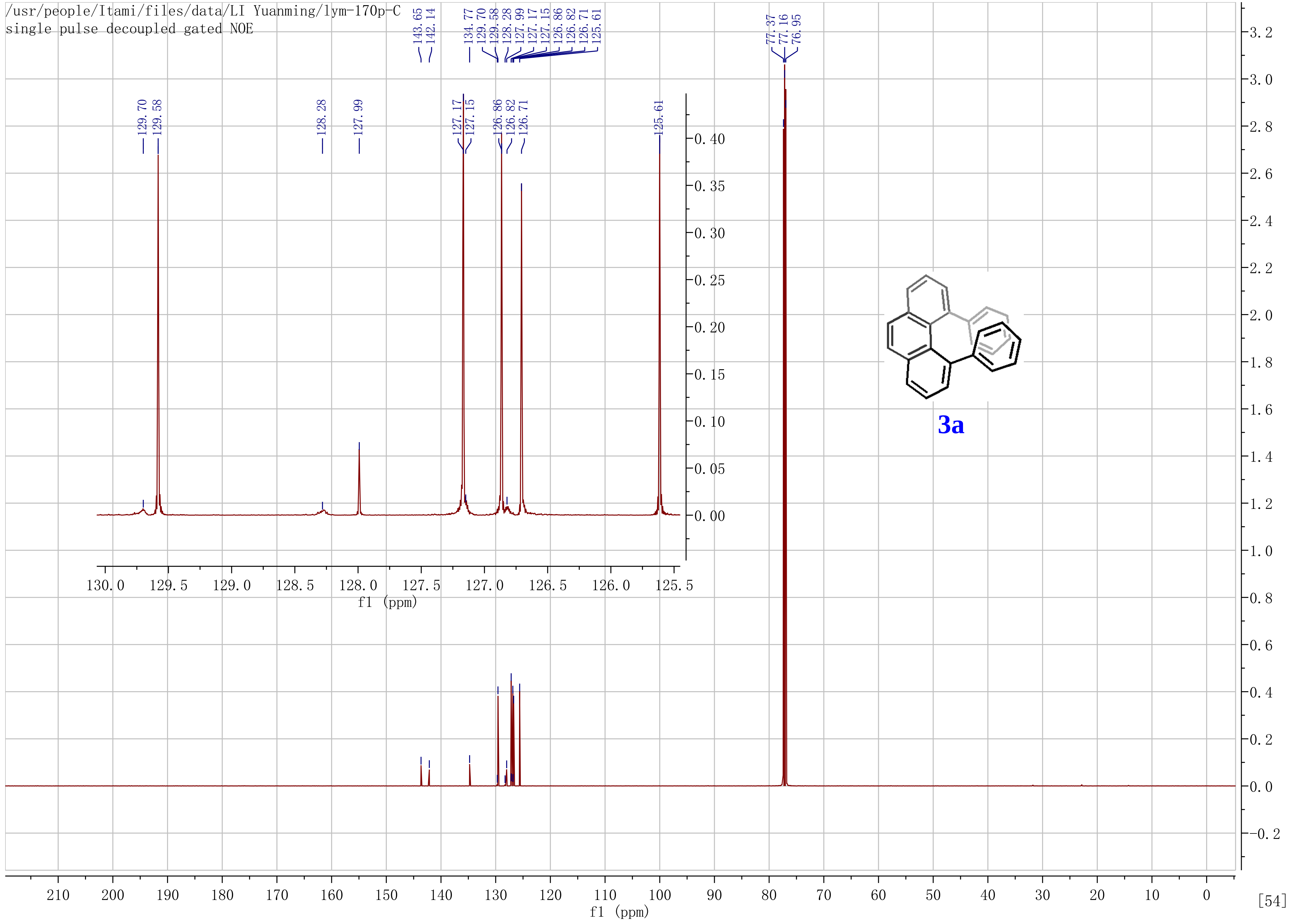
/usr/people/Itami/files/data/LI Yuanming/lym-170p-C
single pulse decoupled gated NOE
143.65
142.14
134.77
129.70
129.58
128.28
127.99
127.17
127.15
126.86
126.82
126.71
125.61
77.37
77.16
76.95
f1 (ppm)
3a

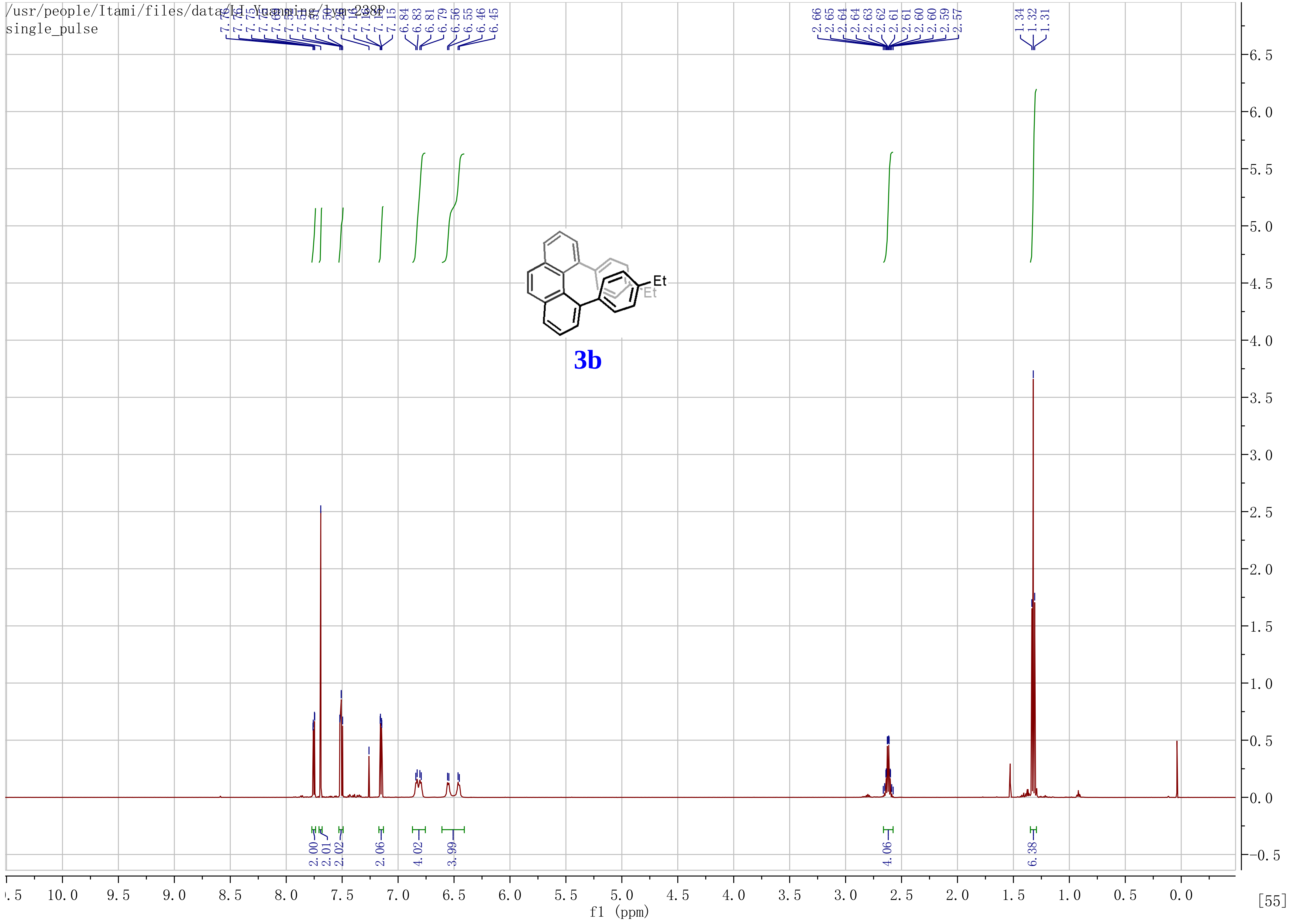

single_pulse
6.84
6.83
6.81
6.79
6.56
6.55
6.46
6.45
2.66
2.65
2.64
2.64
2.63
2.62
2.61
2.61
2.60
2.60
2.59
2.57
1.34
1.32
1.31
Et
3b
2.00
2.01
2.02
2.06
4.02
3.99
4.06
6.38
f1 (ppm)

[55]

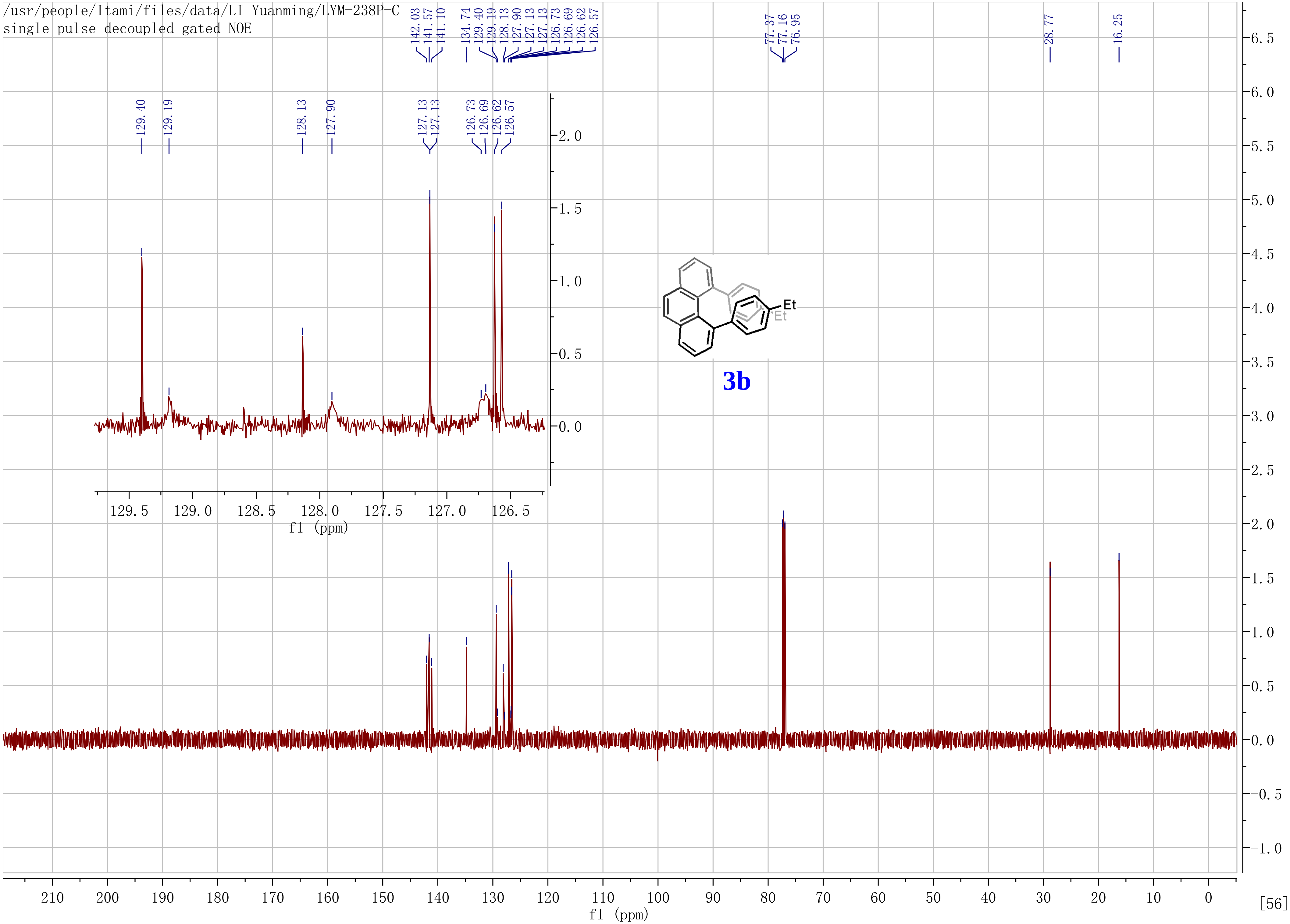

/usr/people/Itami/files/data/LI Yuanming/LYM-238P-C
single pulse decoupled gated NOE
142.03
141.57
141.10
134.74
129.40
129.19
128.13
127.90
127.13
127.13
126.73
126.69
126.62
126.57
77.37
77.16
76.95
28.77
16.25
Et
Et
3b
f1 (ppm)
f1 (ppm)

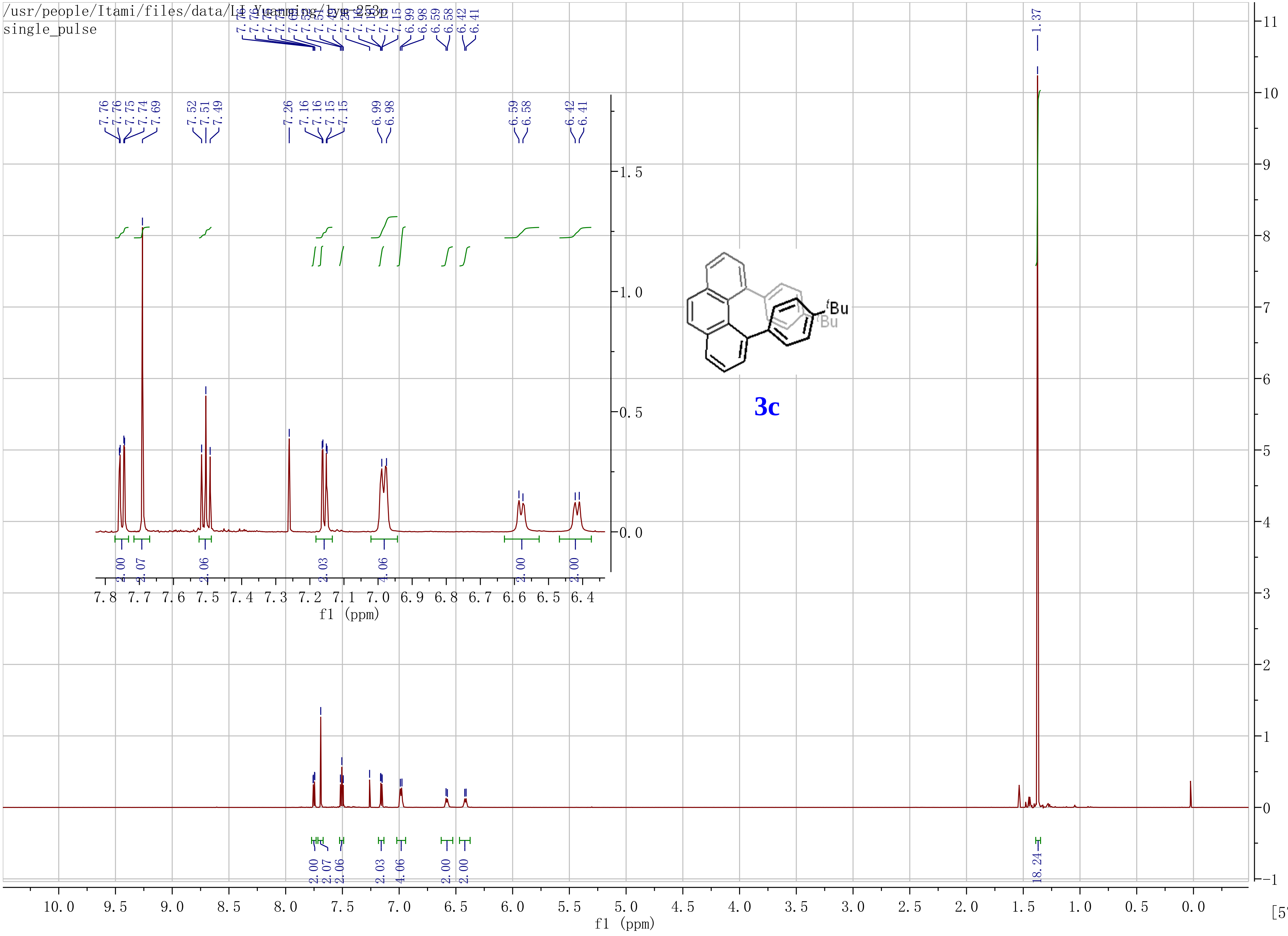
/usr/people/Itami/files/data/Li Yuanming/lym-253p
single_pulse
3c
f1 (ppm)

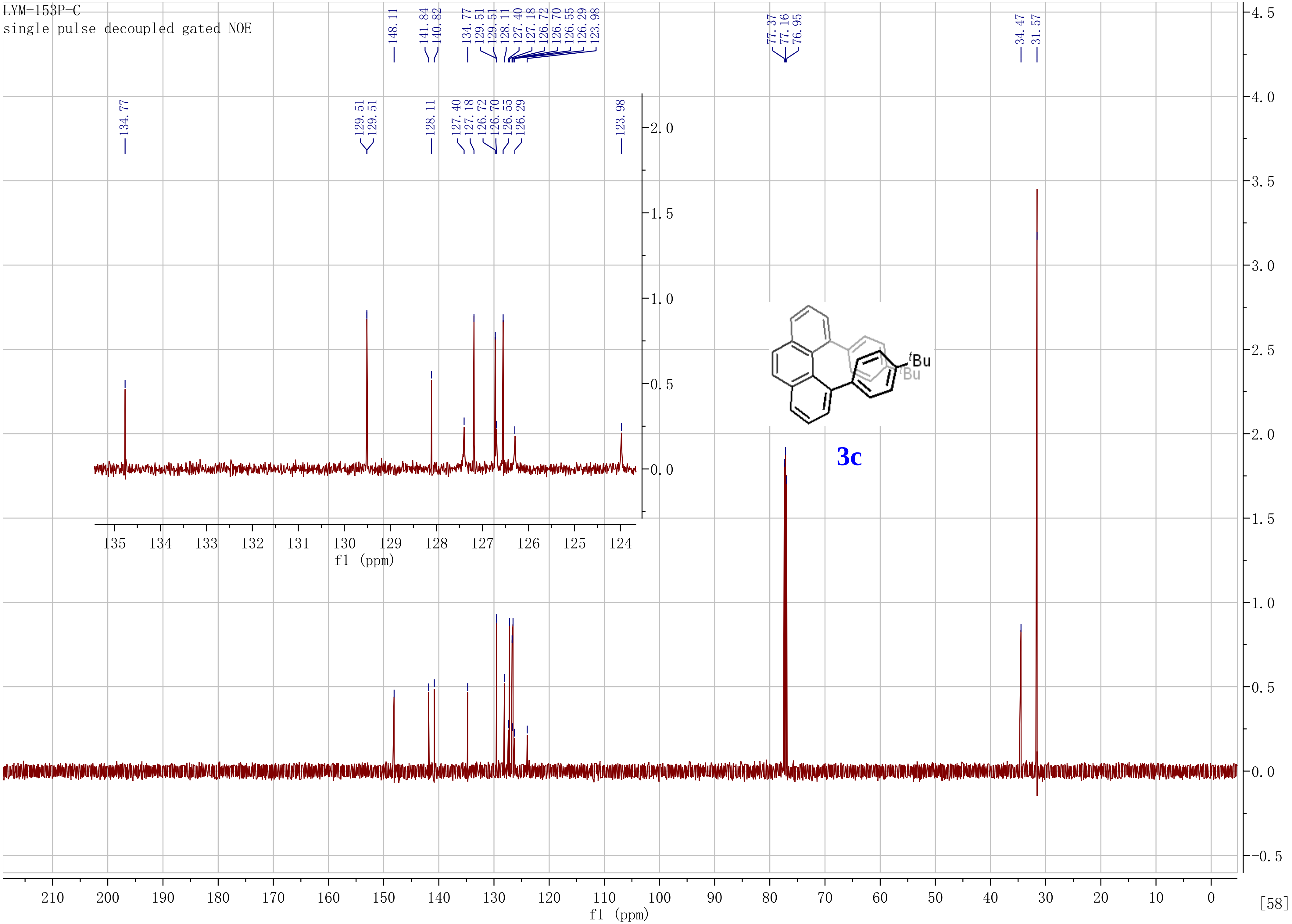
LYM-153P-C
single pulse decoupled gated NOE
148.11
141.84
140.82
134.77
129.51
129.51
128.11
127.40
127.18
126.72
126.70
126.55
126.29
123.98
77.37
77.16
76.95
34.47
31.57
f1 (ppm)
tBu
tBu

3c

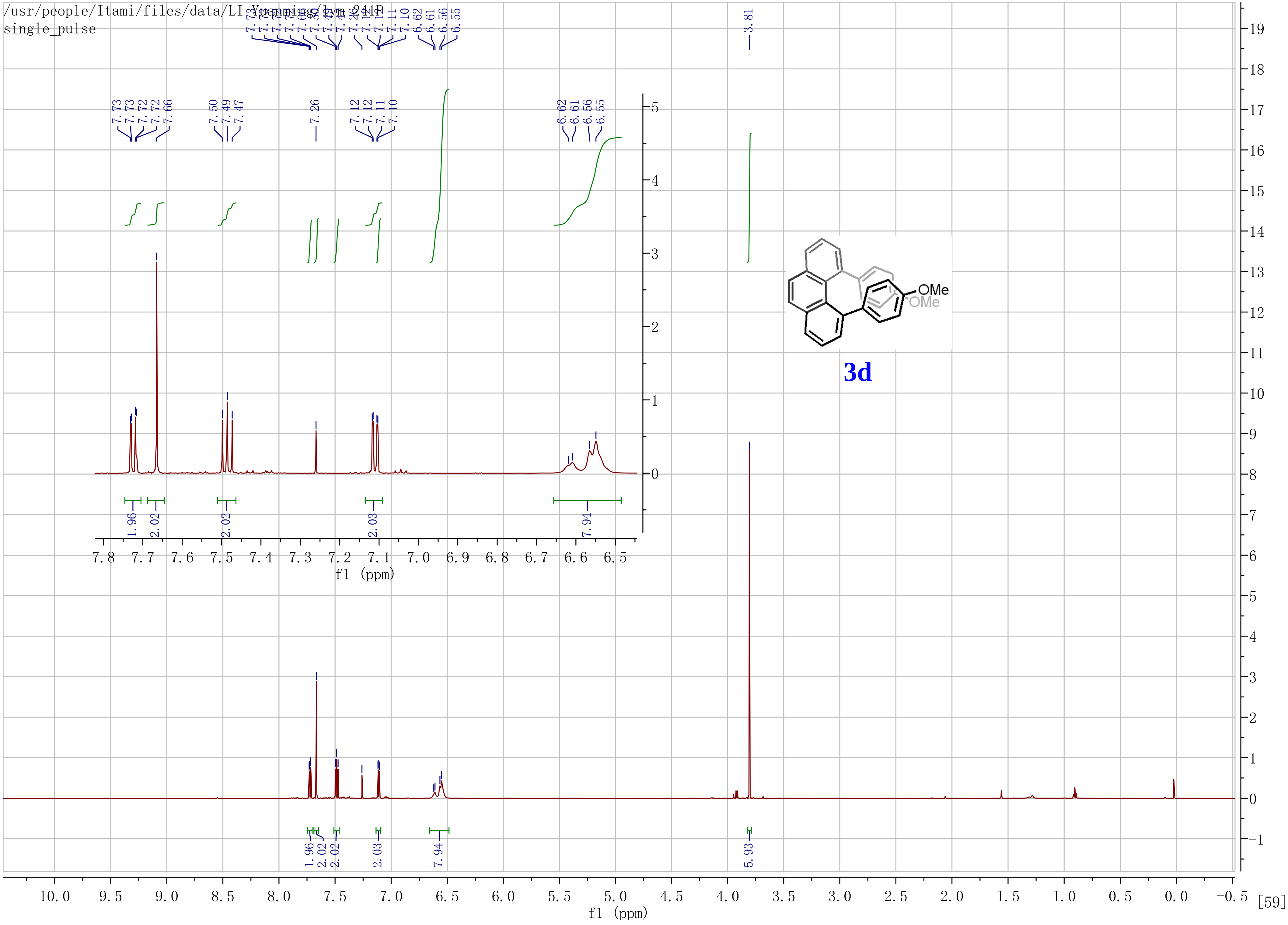

/usr/people/Itami/files/data/LI
single_pulse
7.73
7.73
7.72
7.72
7.66
7.50
7.49
7.47
7.26
7.12
7.12
7.11
7.10
6.62
6.61
6.56
6.55
3.81
1.96
2.02
2.02
2.03
7.94
5.93
OMe
OMe
3d
f1 (ppm)

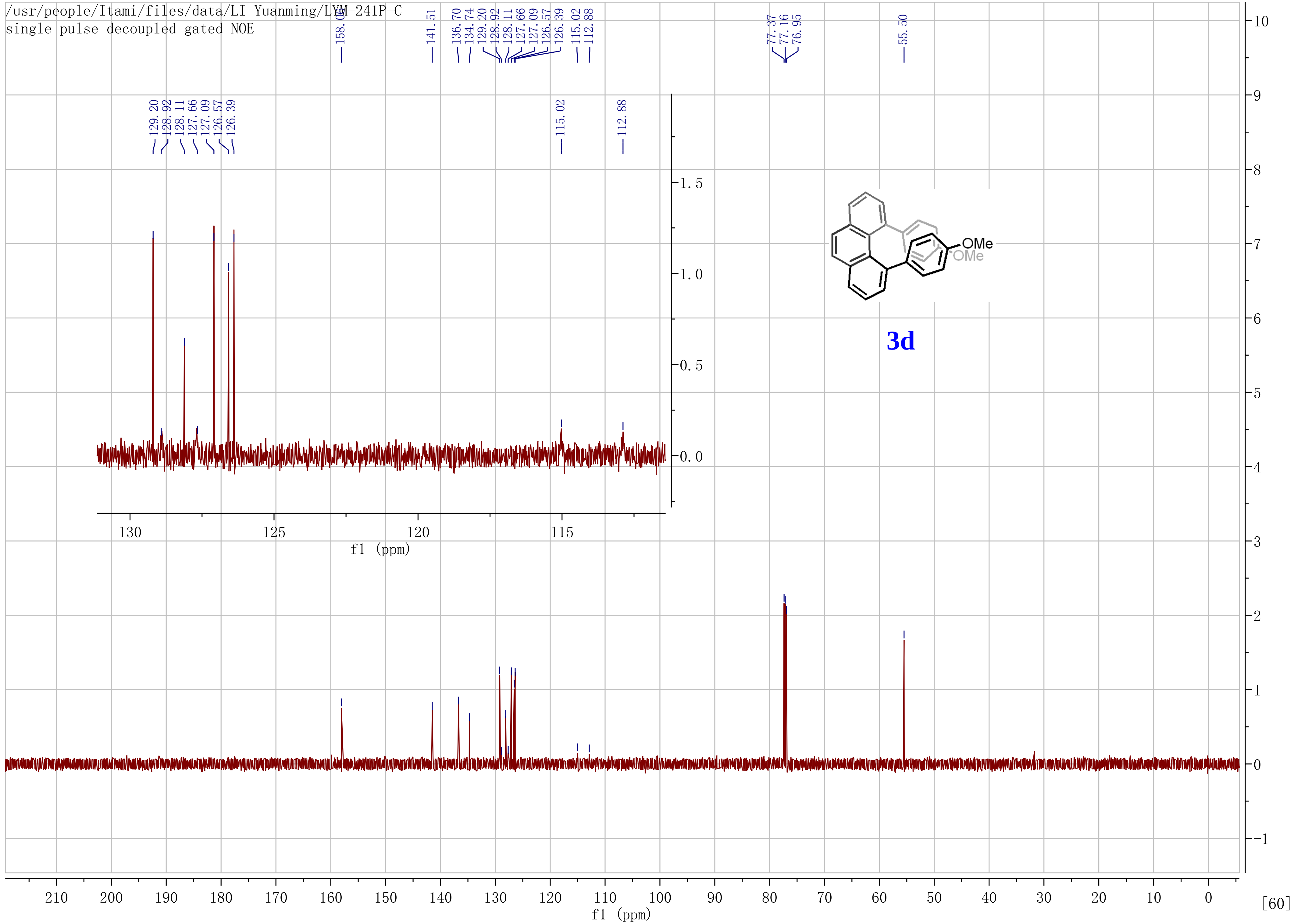

/usr/people/Itami/files/data/LI Yuanming/LYM-241P-C
single pulse decoupled gated NOE
158.0[illegible]
141.51
136.70
134.74
129.20
128.92
128.11
127.66
127.09
126.57
126.39
115.02
112.88
77.37
77.16
76.95
55.50
OMe
OMe
3d
f1 (ppm)

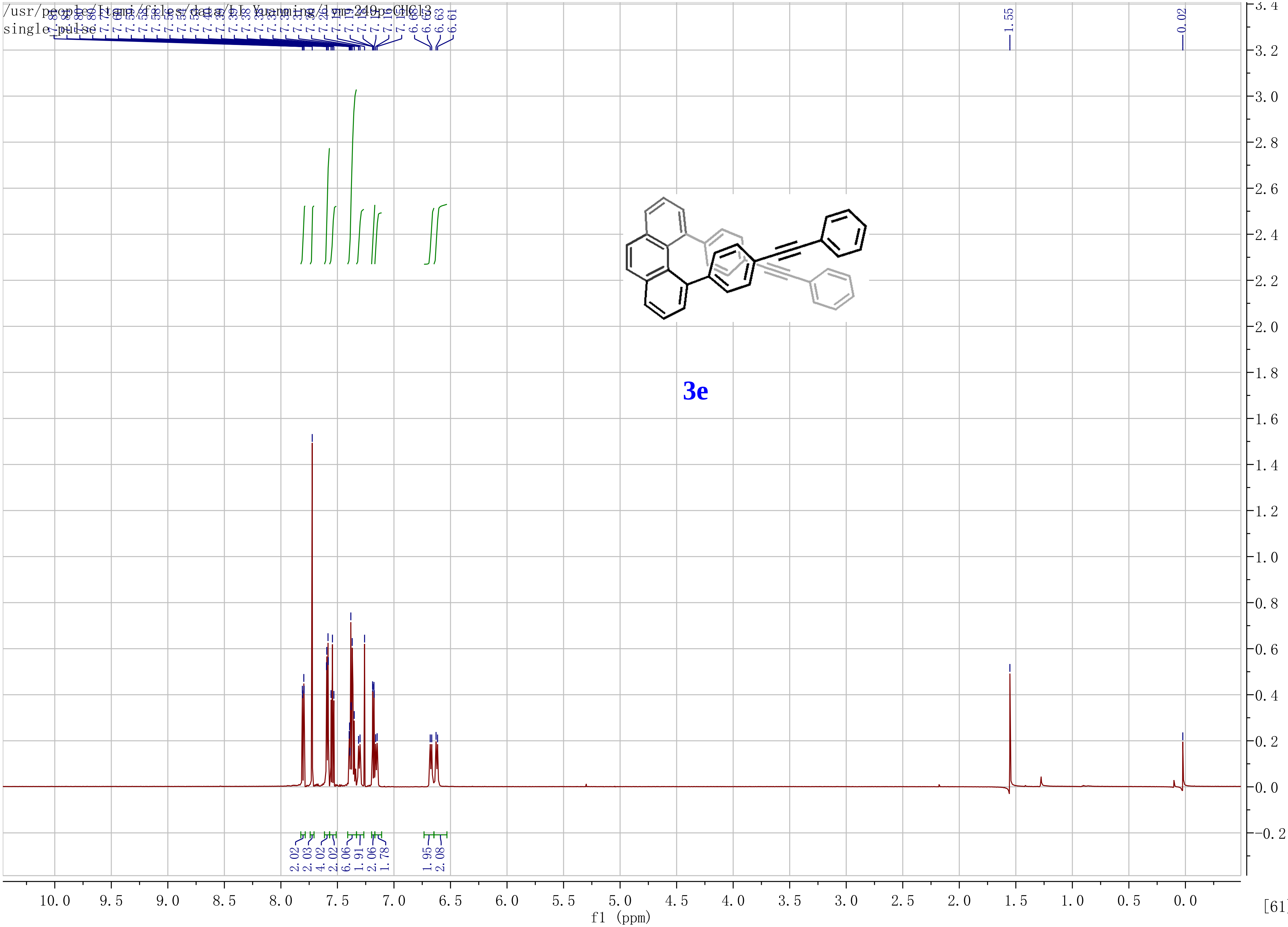
1.55
0.02
3e
2.02
2.03
4.02
2.02
6.06
1.91
2.06
1.78
1.95
2.08
f1 (ppm)

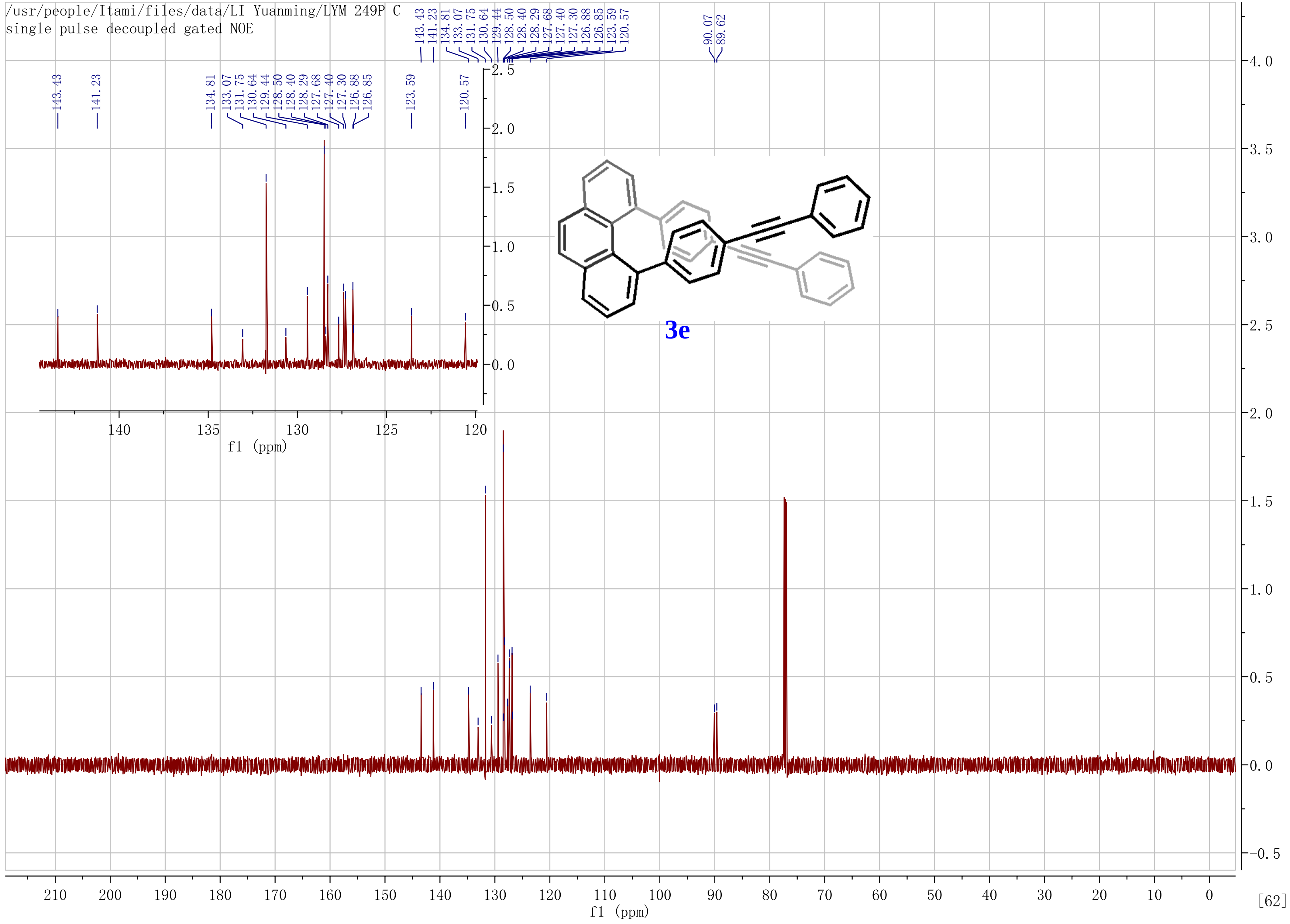
/usr/people/Itami/files/data/LI Yuanming/LYM-249P-C
single pulse decoupled gated NOE
143.43
141.23
134.81
133.07
131.75
130.64
129.44
128.50
128.40
128.29
127.68
127.40
127.30
126.88
126.85
123.59
120.57
90.07
89.62
3e
f1 (ppm)
f1 (ppm)

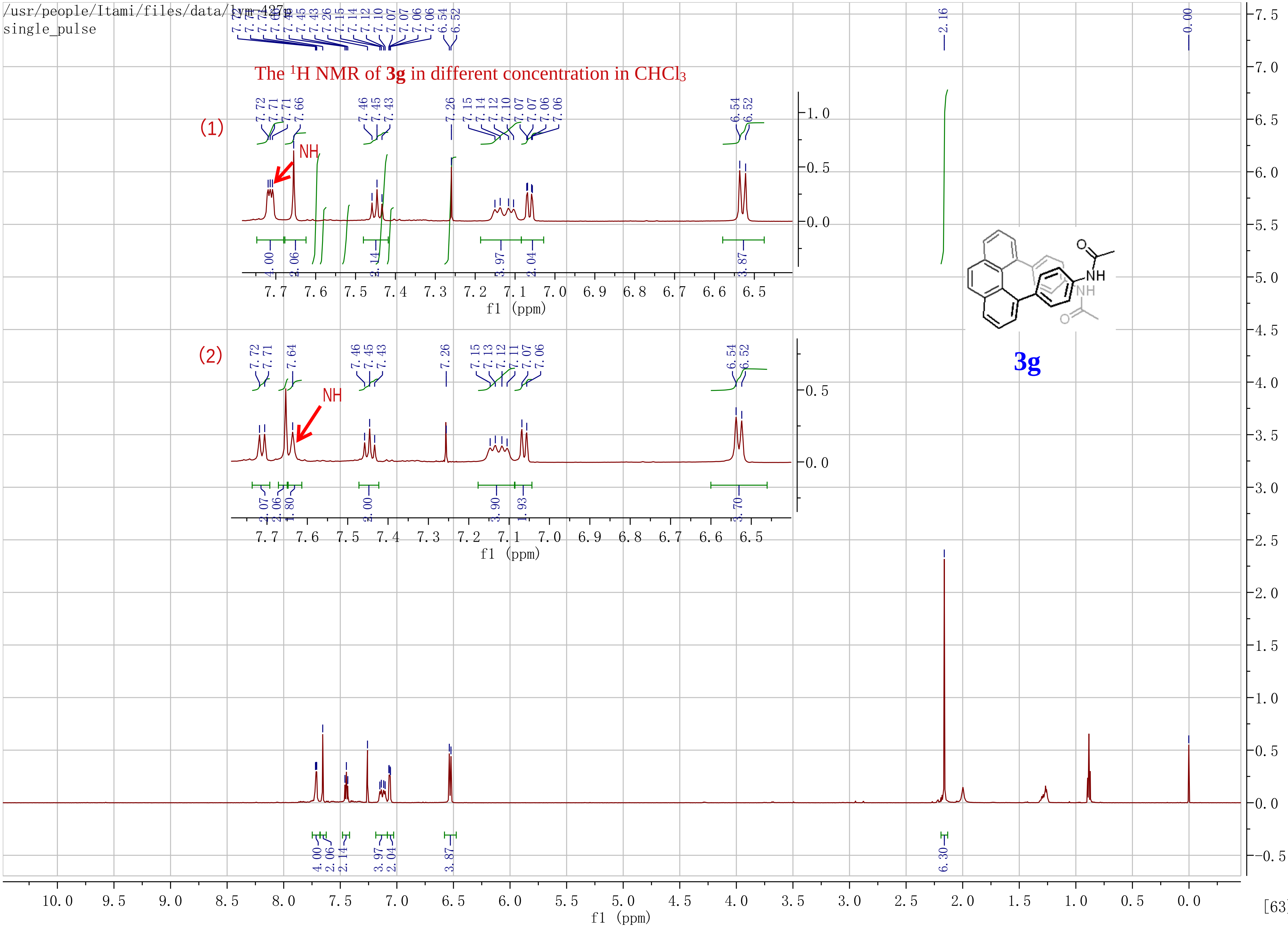

/usr/people/Itami/files/data/lym-427p
single_pulse
The ¹H NMR of 3g in different concentration in CHCl3
(1)
(2)
NH
NH
3g
f1 (ppm)

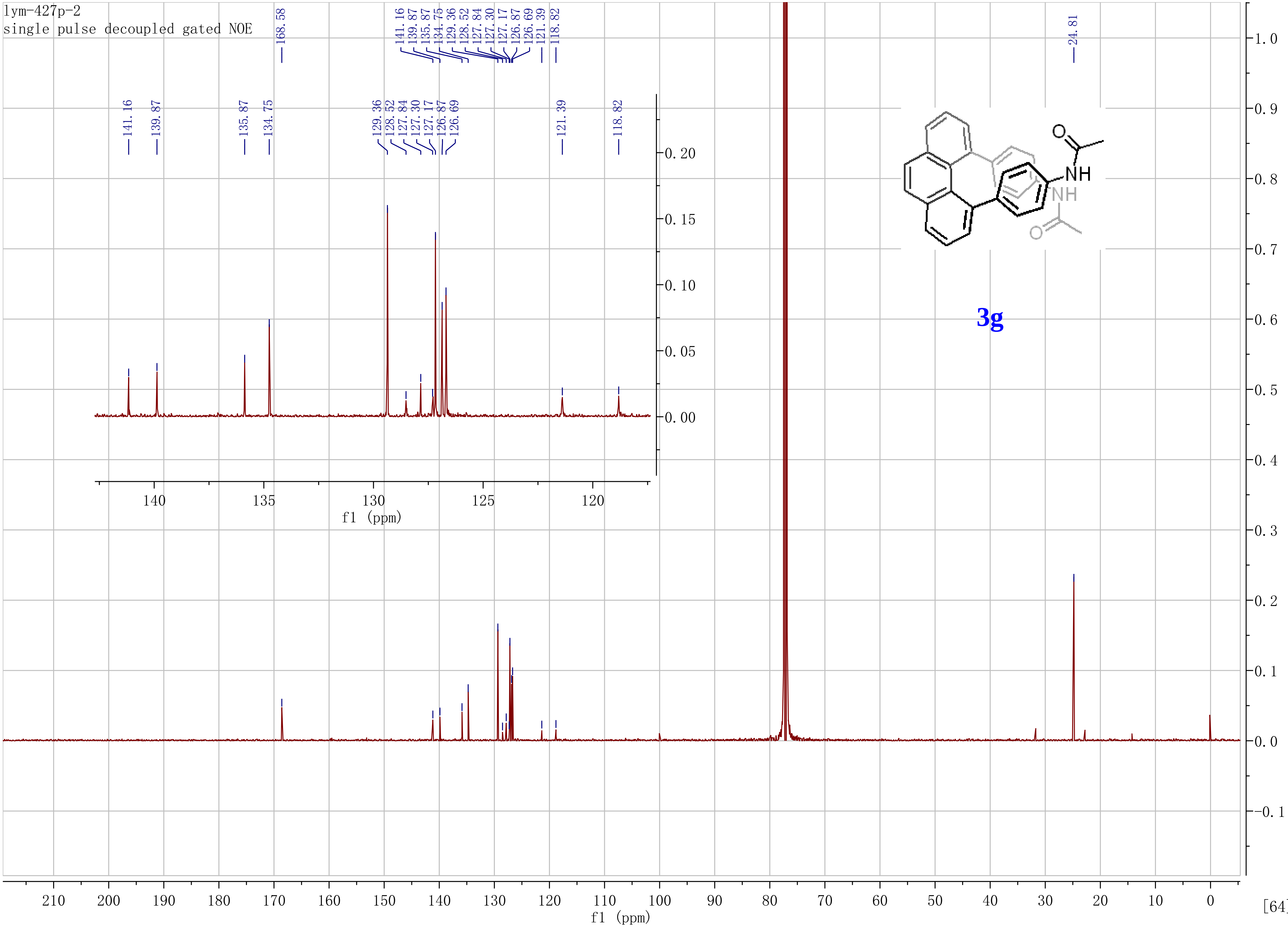
lym-427p-2
single pulse decoupled gated NOE
168.58
141.16
139.87
135.87
134.75
129.36
128.52
127.84
127.30
127.17
126.87
126.69
121.39
118.82
24.81
NH
NH
O
O
3g
f1 (ppm)
f1 (ppm)

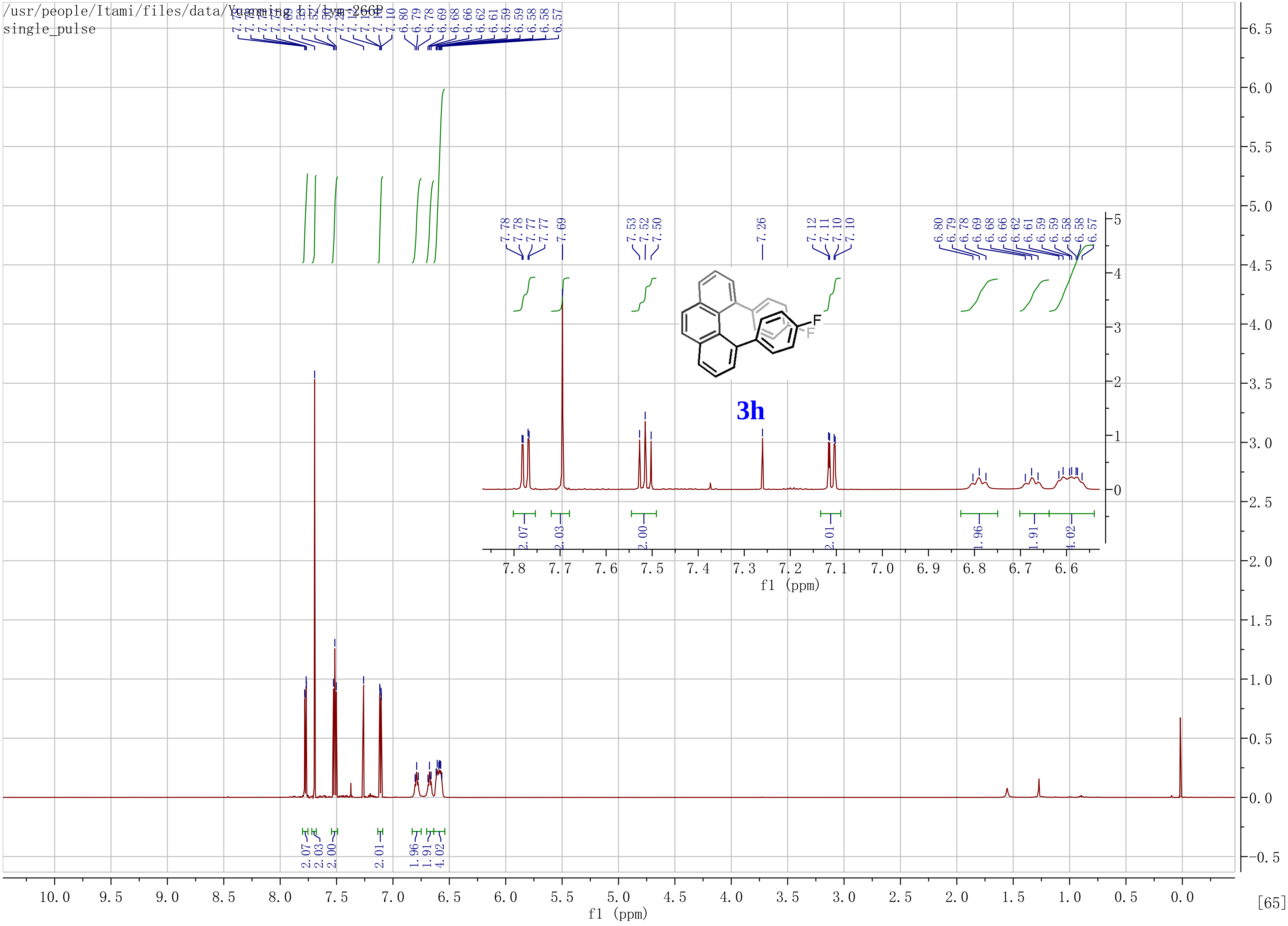

/usr/people/Itami/files/data/Yuanming Li/lym-266P
single_pulse
7.78 7.78 7.77 7.77 7.69
7.53 7.52 7.50
7.26
7.12 7.11 7.10 7.10
6.80 6.79 6.78 6.69 6.68 6.66 6.62 6.61 6.59 6.58 6.58 6.57
F
F
3h
2.07
2.03
2.00
2.01
1.96
1.91
4.02
f1 (ppm)

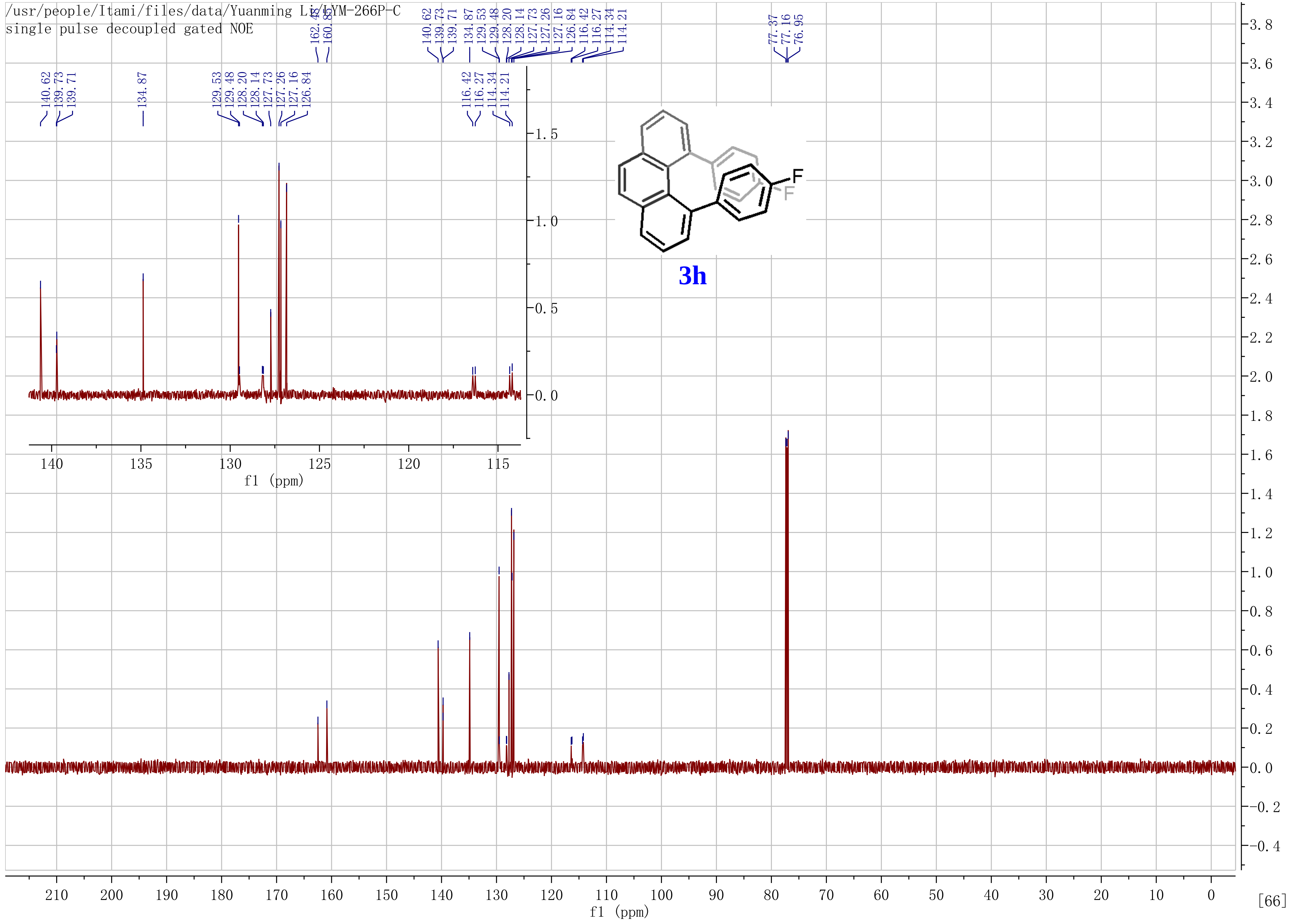
/usr/people/Itami/files/data/Yuanming Li/LYM-266P-C
single pulse decoupled gated NOE
162.41
160.85
140.62
139.73
139.71
134.87
129.53
129.48
128.20
128.14
127.73
127.26
127.16
126.84
116.42
116.27
114.34
114.21
77.37
77.16
76.95
3h
F
f1 (ppm)

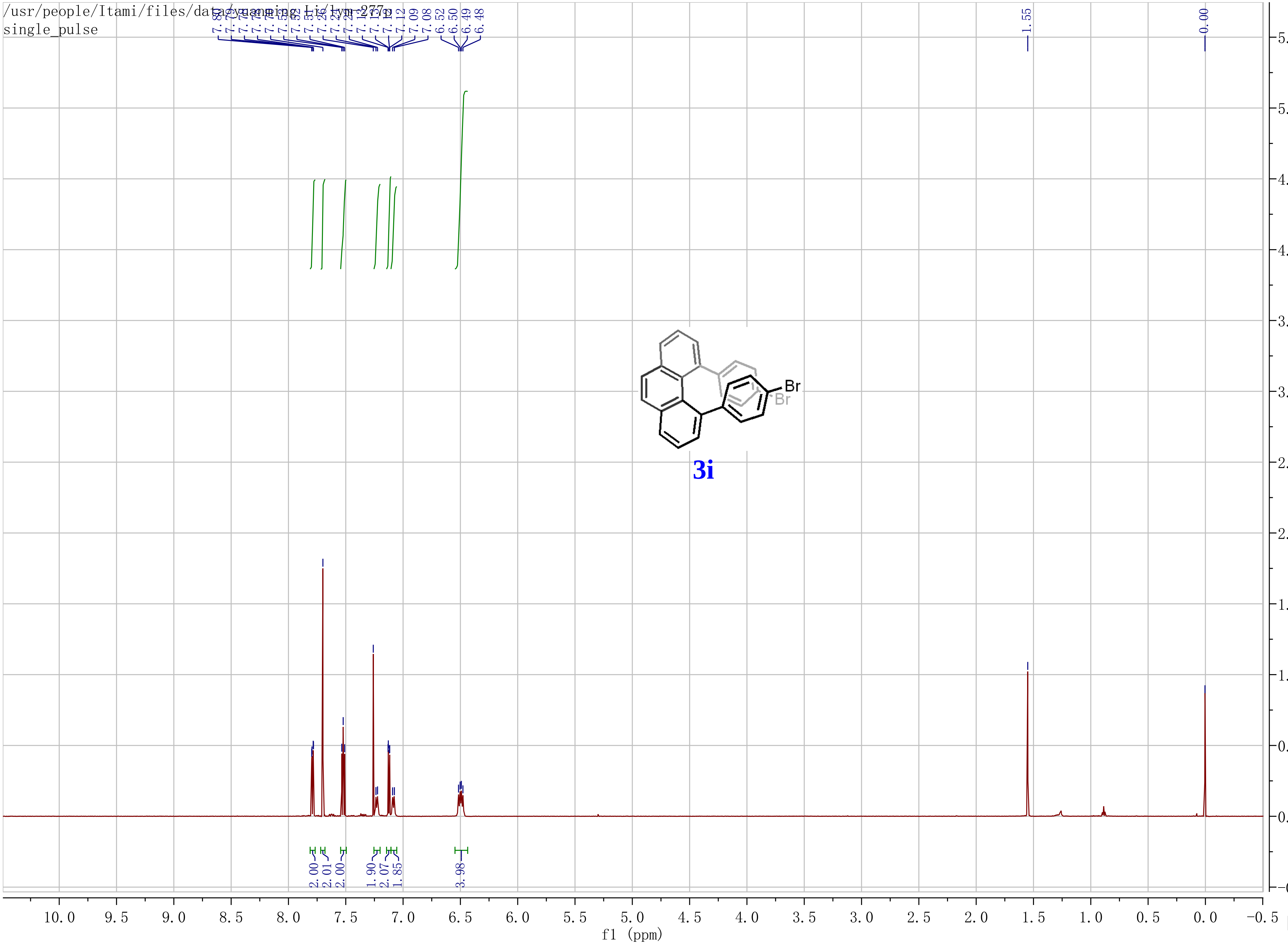
/usr/people/Itami/files/data/yuanming Li/lym-277p
single_pulse
7.12
7.09
7.08
6.52
6.50
6.49
6.48
1.55
0.00
Br
Br
3i
2.00
2.01
2.00
1.90
2.07
1.85
3.98
f1 (ppm)

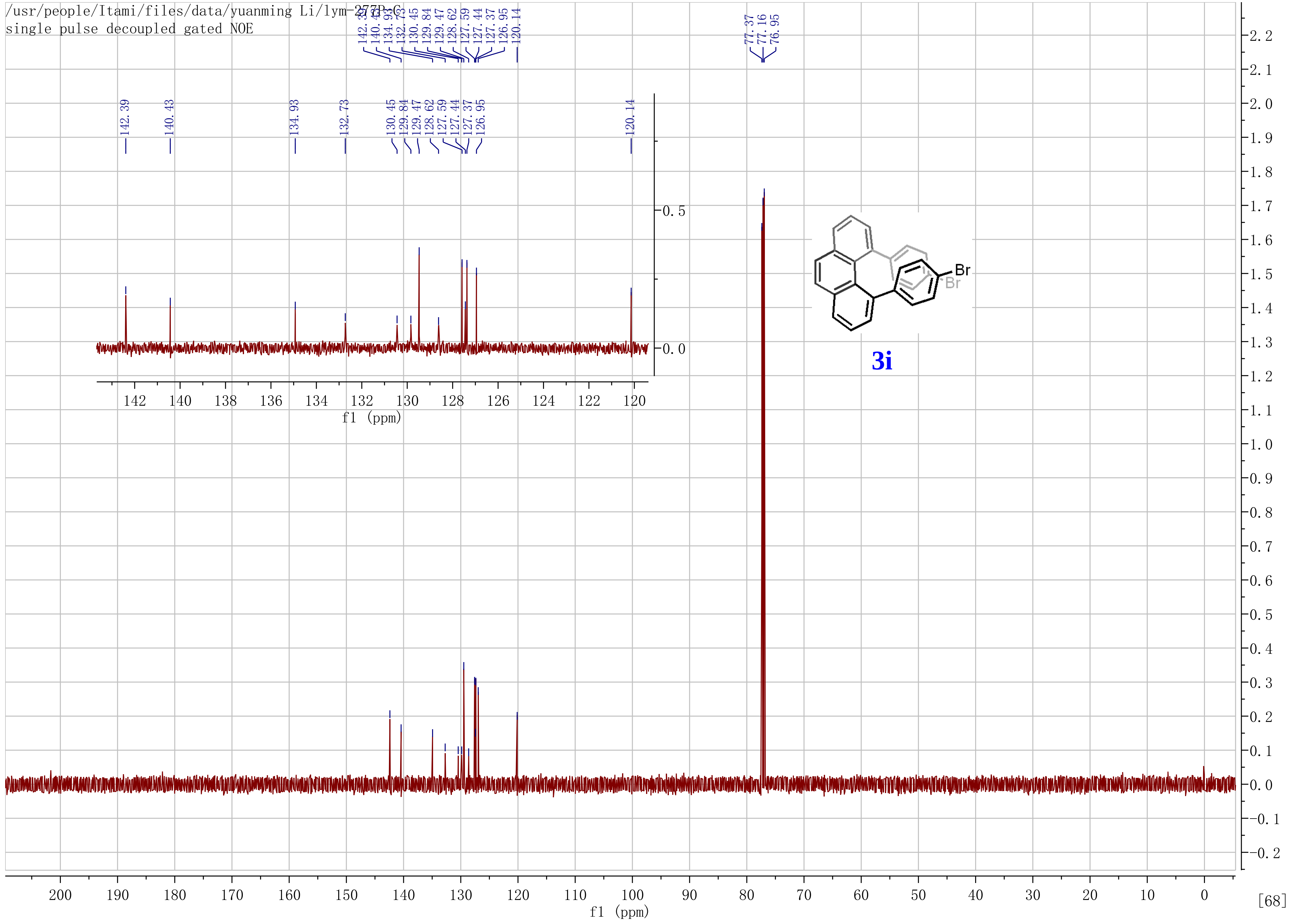
/usr/people/Itami/files/data/yuanming Li/lym-277P-C
single pulse decoupled gated NOE
142.39
140.43
134.93
132.73
130.45
129.84
129.47
128.62
127.59
127.44
127.37
126.95
120.14
77.37
77.16
76.95
Br
3i
f1 (ppm)
f1 (ppm)

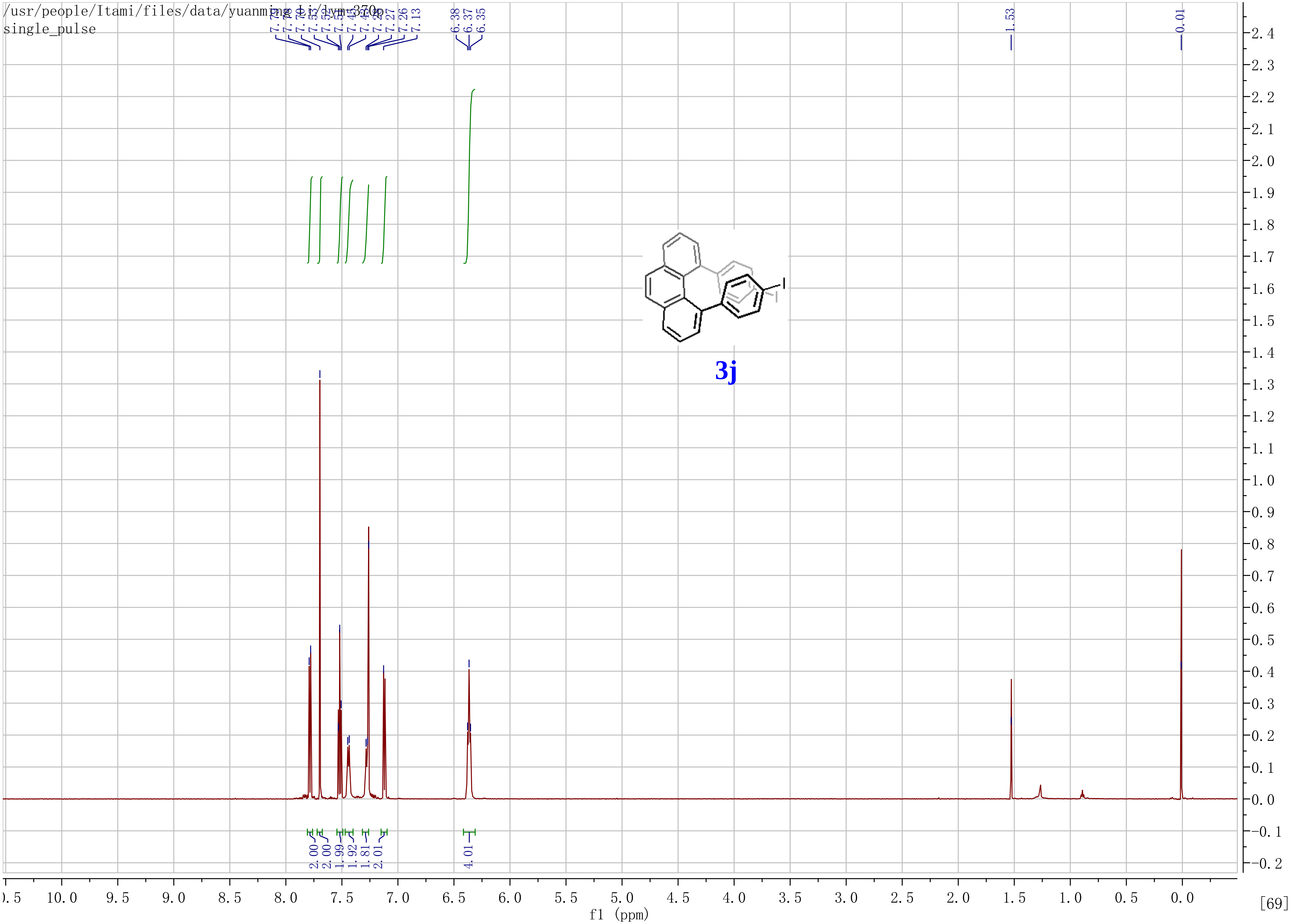
/usr/people/Itami/files/data/yuanming li/lym-370p
single_pulse
6.38
6.37
6.35
1.53
0.01
7.27
7.26
7.13
2.00
2.00
1.99
1.92
1.81
2.01
4.01
3j
f1 (ppm)

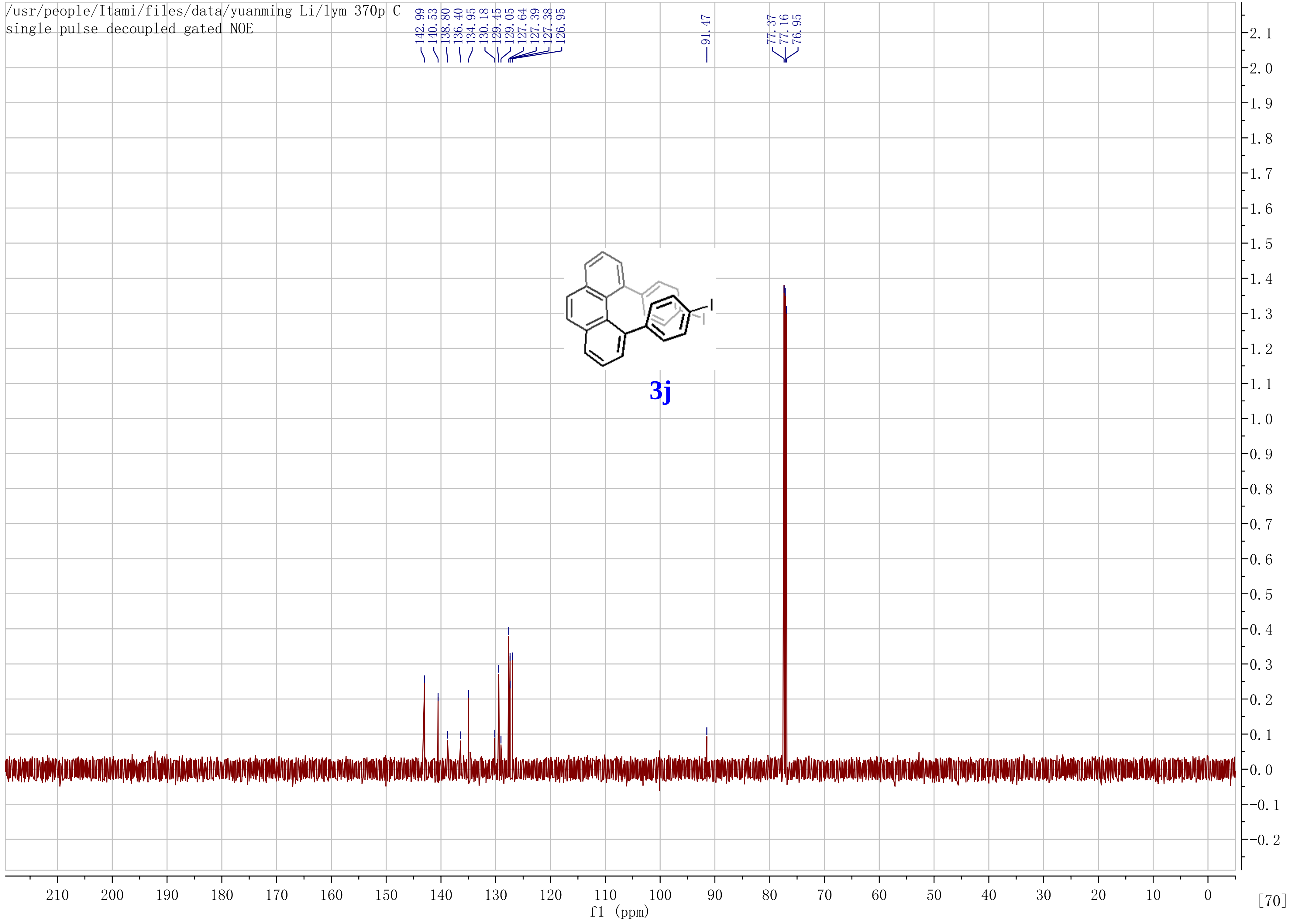

/usr/people/Itami/files/data/yuanming Li/lym-370p-C
single pulse decoupled gated NOE
142.99
140.53
138.80
136.40
134.95
130.18
129.45
129.05
127.64
127.39
127.38
126.95
91.47
77.37
77.16
76.95
3j
f1 (ppm)

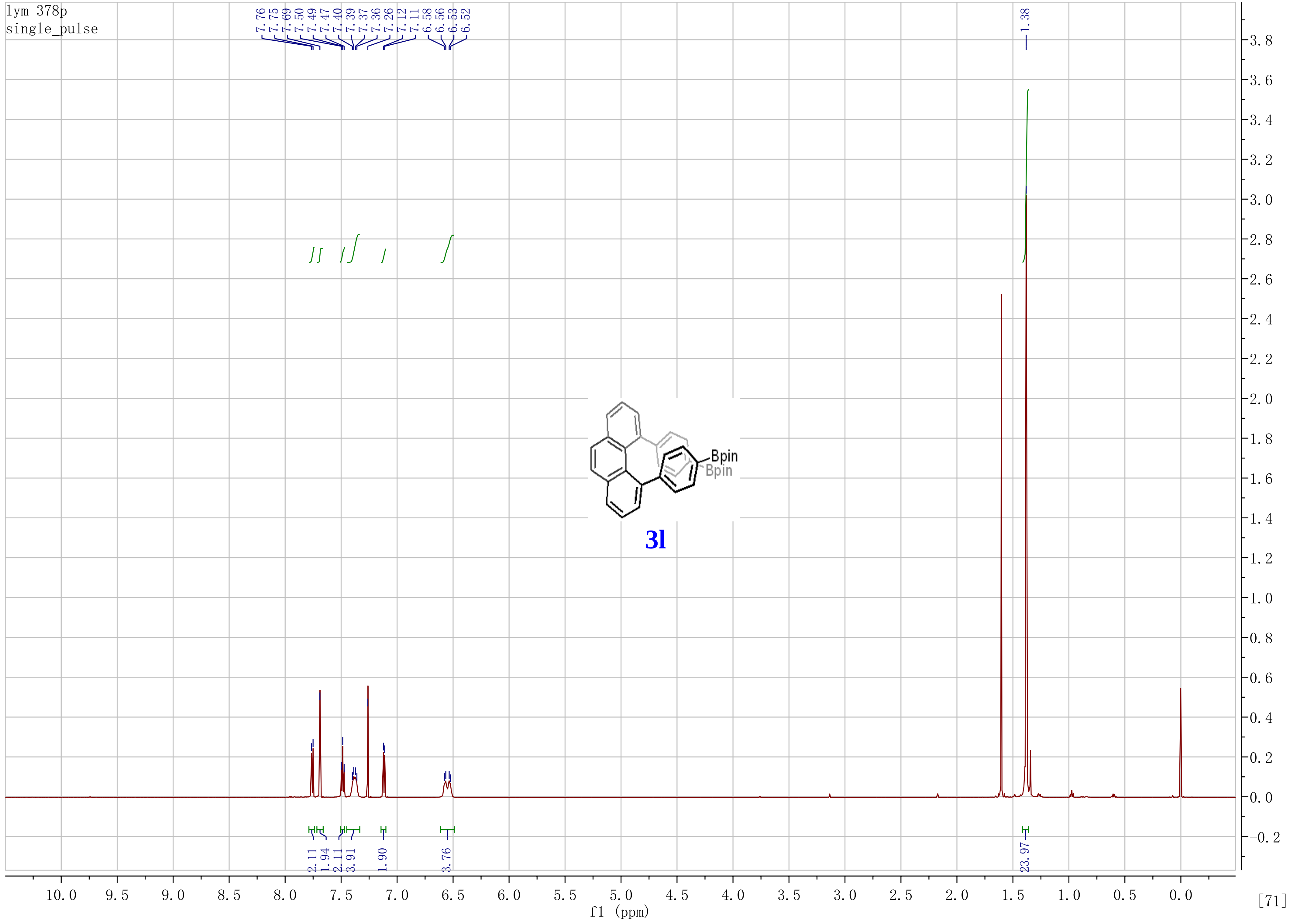
lym-378p
single_pulse
7.76 7.75 7.69 7.50 7.49 7.47 7.40 7.39 7.37 7.36 7.26 7.12 7.11 6.58 6.56 6.53 6.52
1.38
2.11 1.94 2.11 3.91 1.90 3.76
23.97
Bpin
Bpin
3l
f1 (ppm)

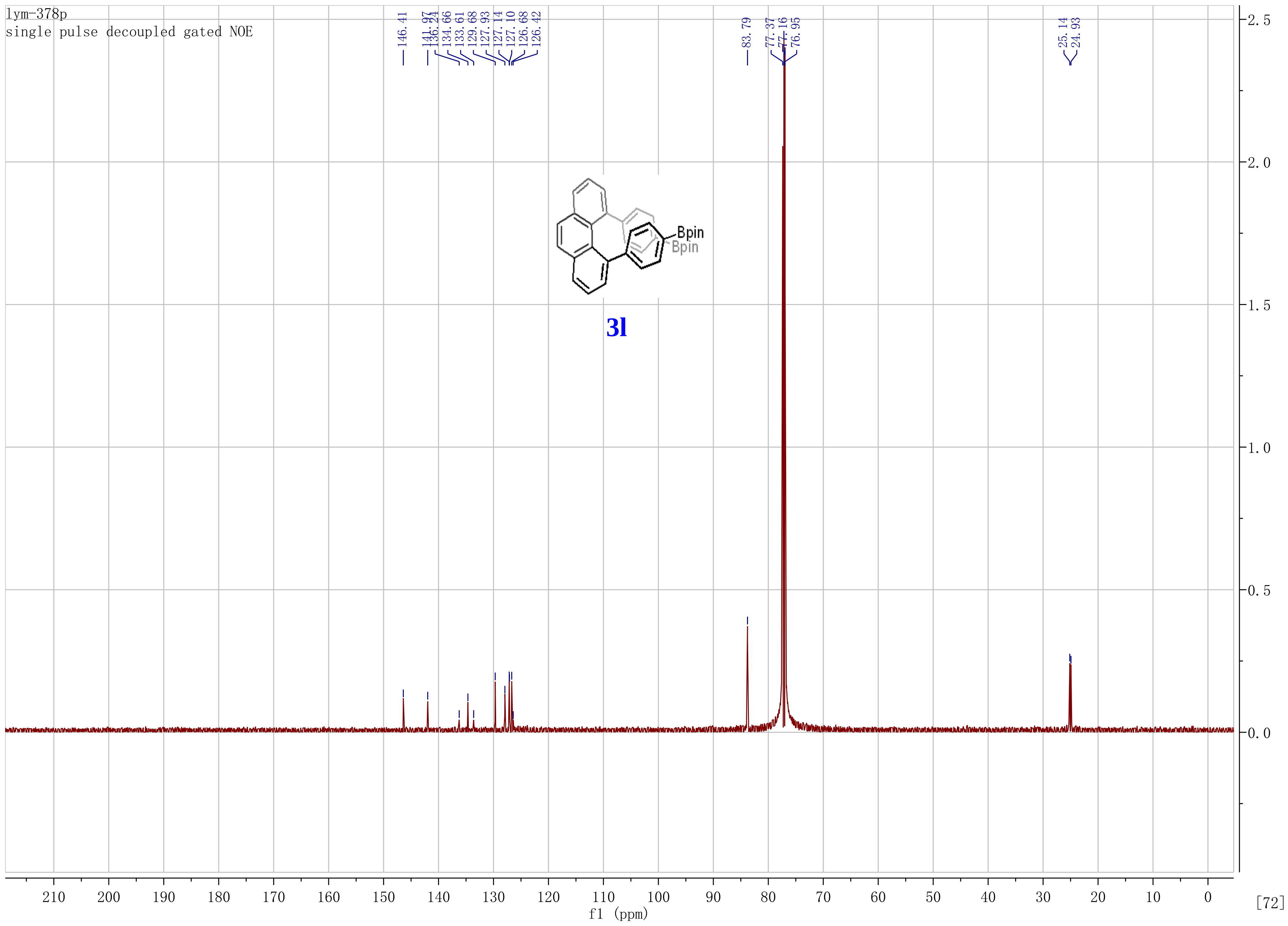

lym-378p
single pulse decoupled gated NOE
146.41
141.97
136.24
134.66
133.61
129.68
127.93
127.14
127.10
126.68
126.42
83.79
77.37
77.16
76.95
25.14
24.93
Bpin
Bpin
3l
f1 (ppm)

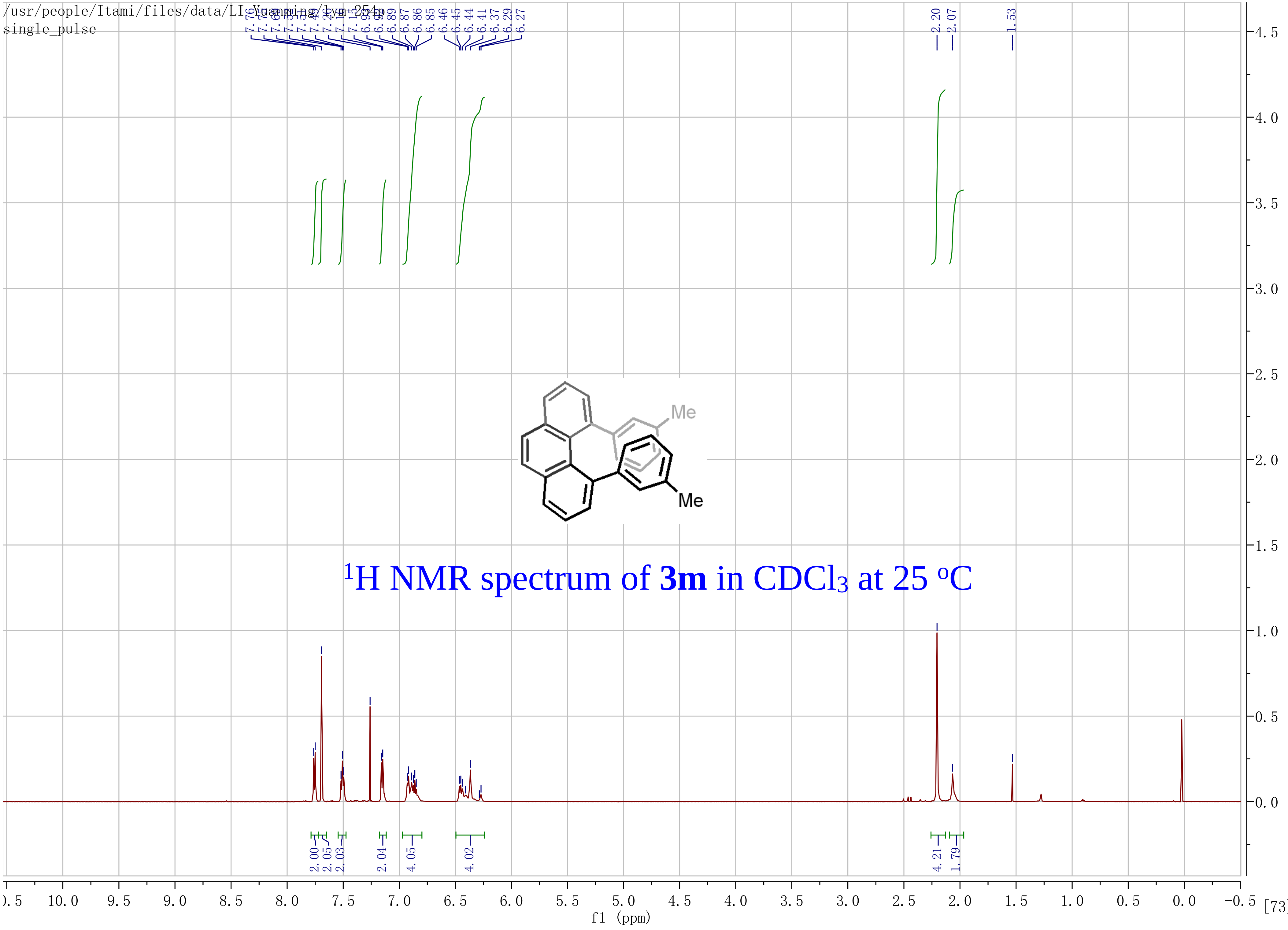

^{1}H NMR spectrum of **3m** in $CDCl_3$ at 25 °C

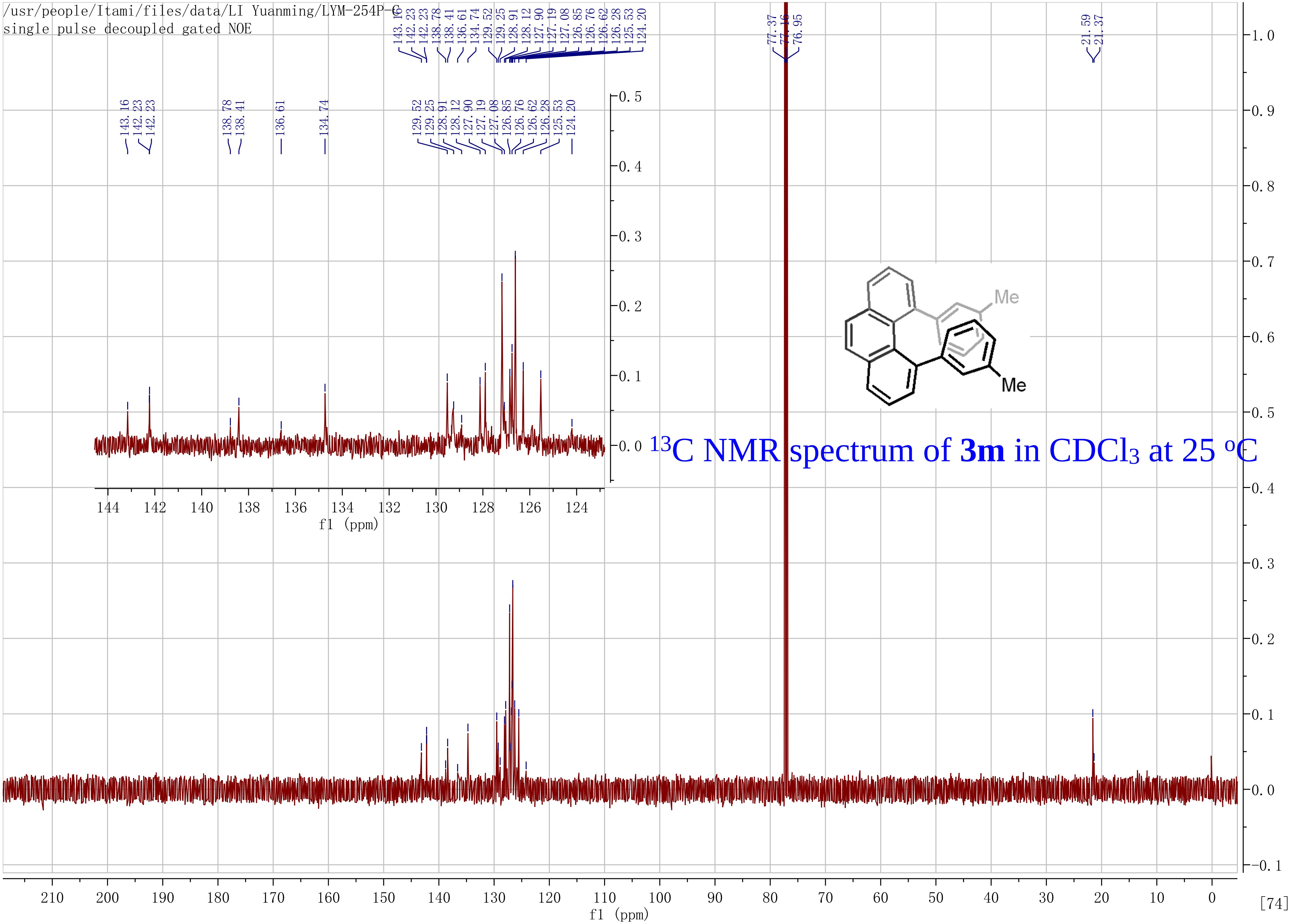

/usr/people/Itami/files/data/LI Yuanming/LYM-254P-C
single pulse decoupled gated NOE
143.16 142.23 142.23 138.78 138.41 136.61 134.74 129.52 129.25 128.91 128.12 127.90 127.19 127.08 126.85 126.76 126.62 126.28 125.53 124.20
77.37 77.16 76.95
21.59 21.37
Me
Me
^{13}C NMR spectrum of 3m in $CDCl_3$ at 25 °C
f1 (ppm)

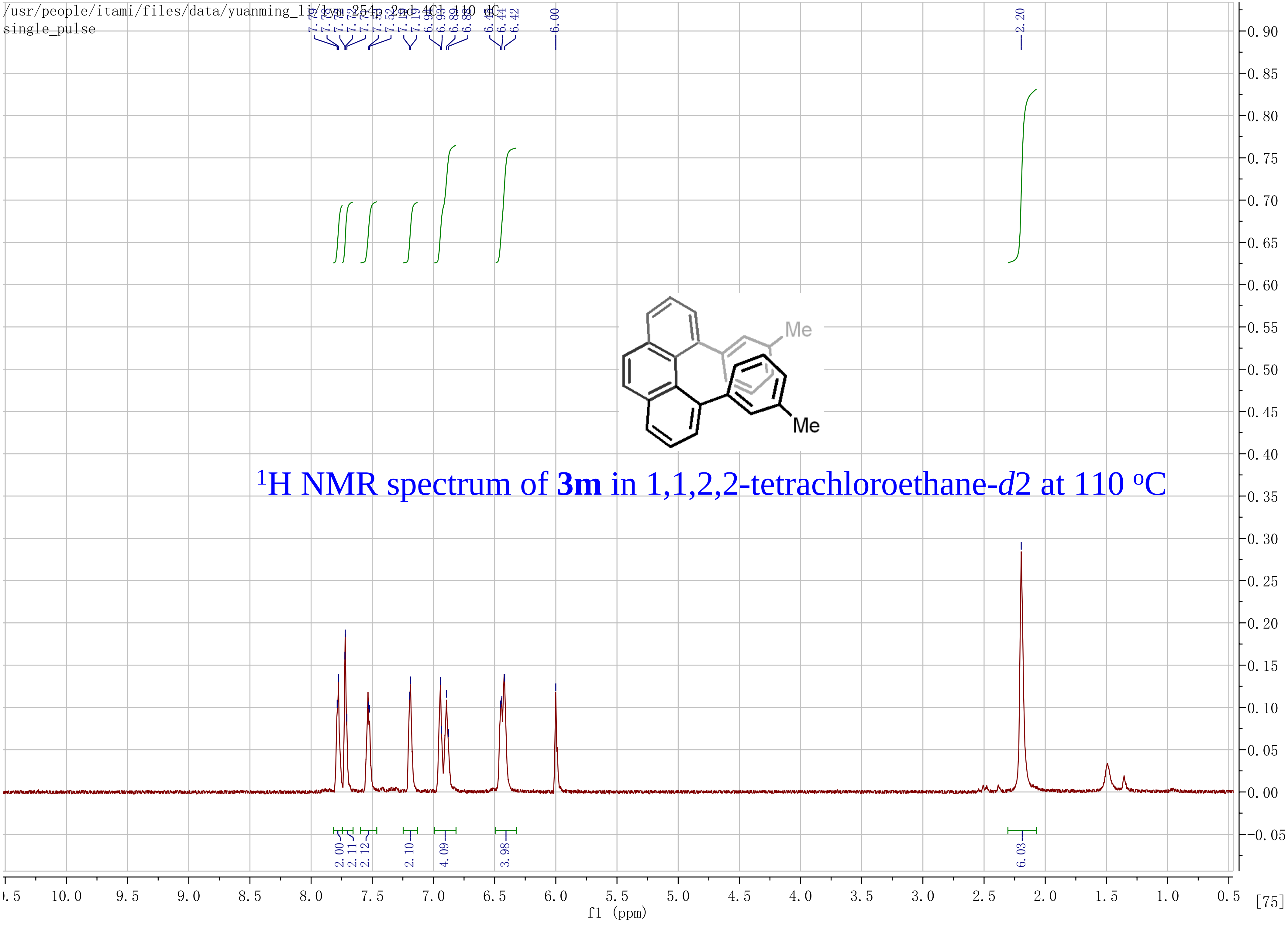

/usr/people/itami/files/data/yuanming_li/lym-254p-2nd-4Cl-110 dC
single_pulse
6.00
2.20
Me
Me
^{1}H NMR spectrum of **3m** in 1,1,2,2-tetrachloroethane-*d*2 at 110 °C
2.00
2.11
2.12
2.10
4.09
3.98
6.03
f1 (ppm)

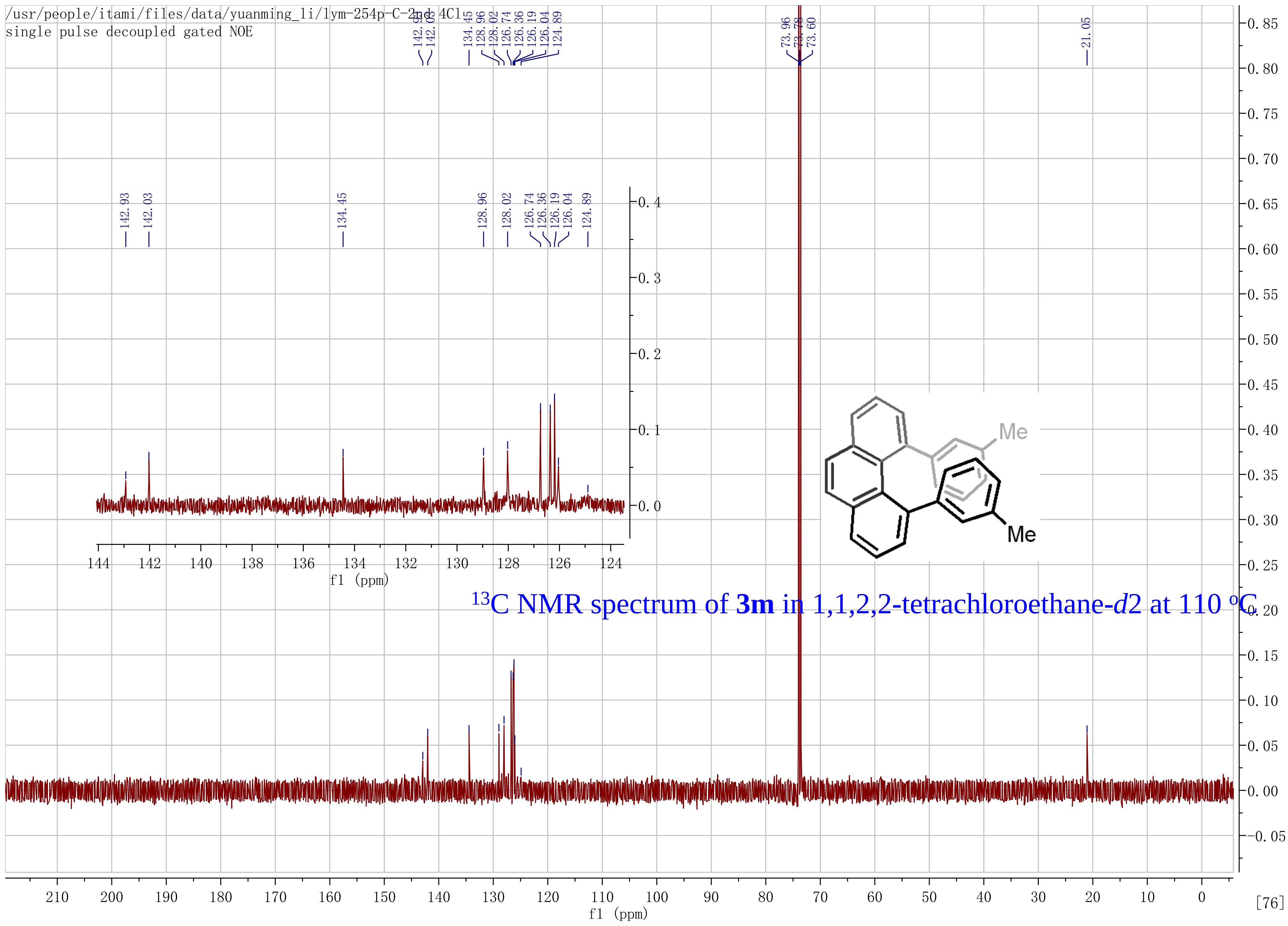

^{13}C NMR spectrum of **3m** in 1,1,2,2-tetrachloroethane-*d*2 at 110 °C

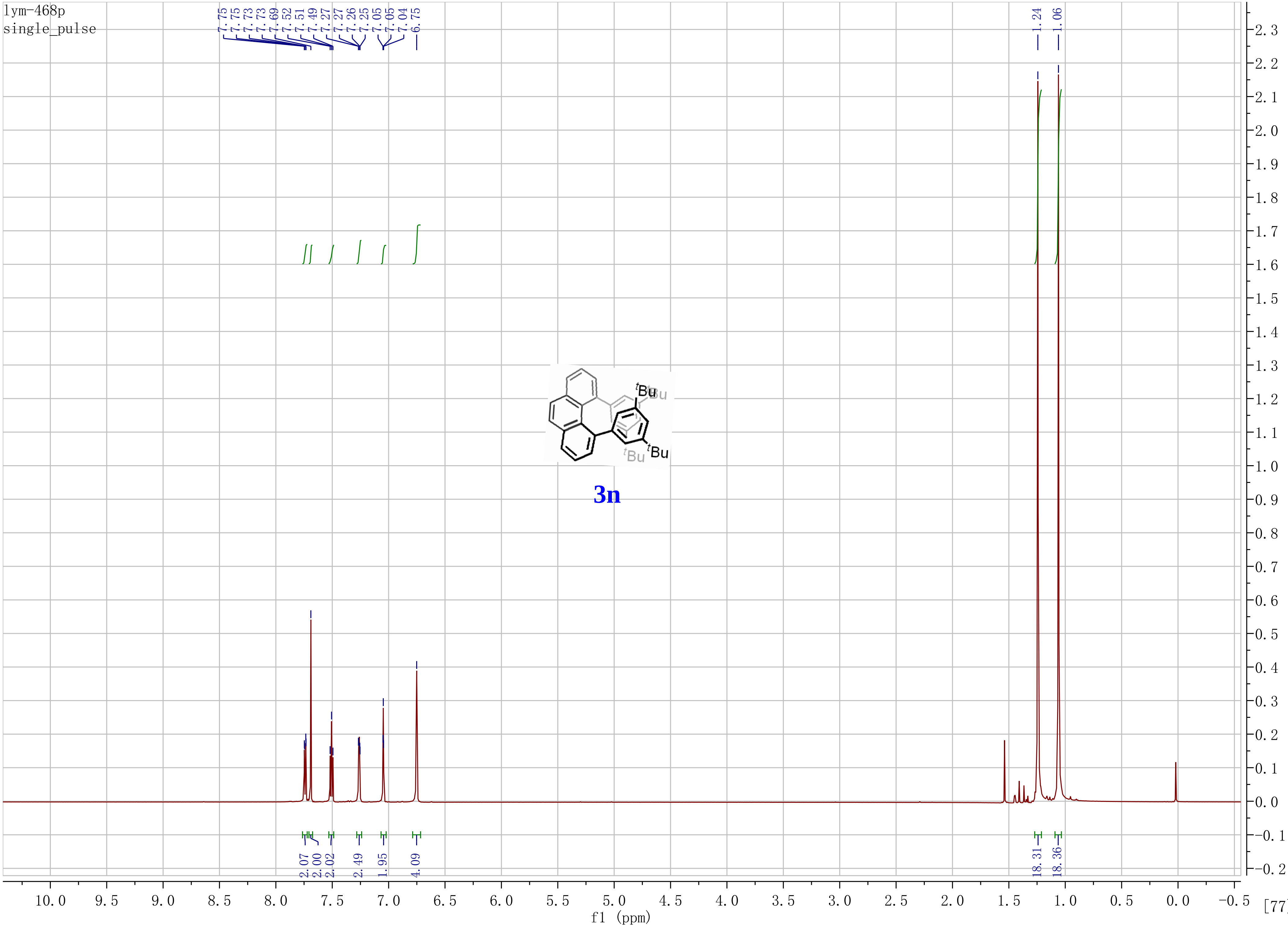
lym-468p
single_pulse
7.75
7.75
7.73
7.73
7.69
7.52
7.51
7.49
7.27
7.26
7.25
7.05
7.05
7.04
6.75
1.24
1.06
tBu
tBu
tBu
tBu
3n
2.07
2.00
2.02
2.49
1.95
4.09
18.31
18.36
f1 (ppm)

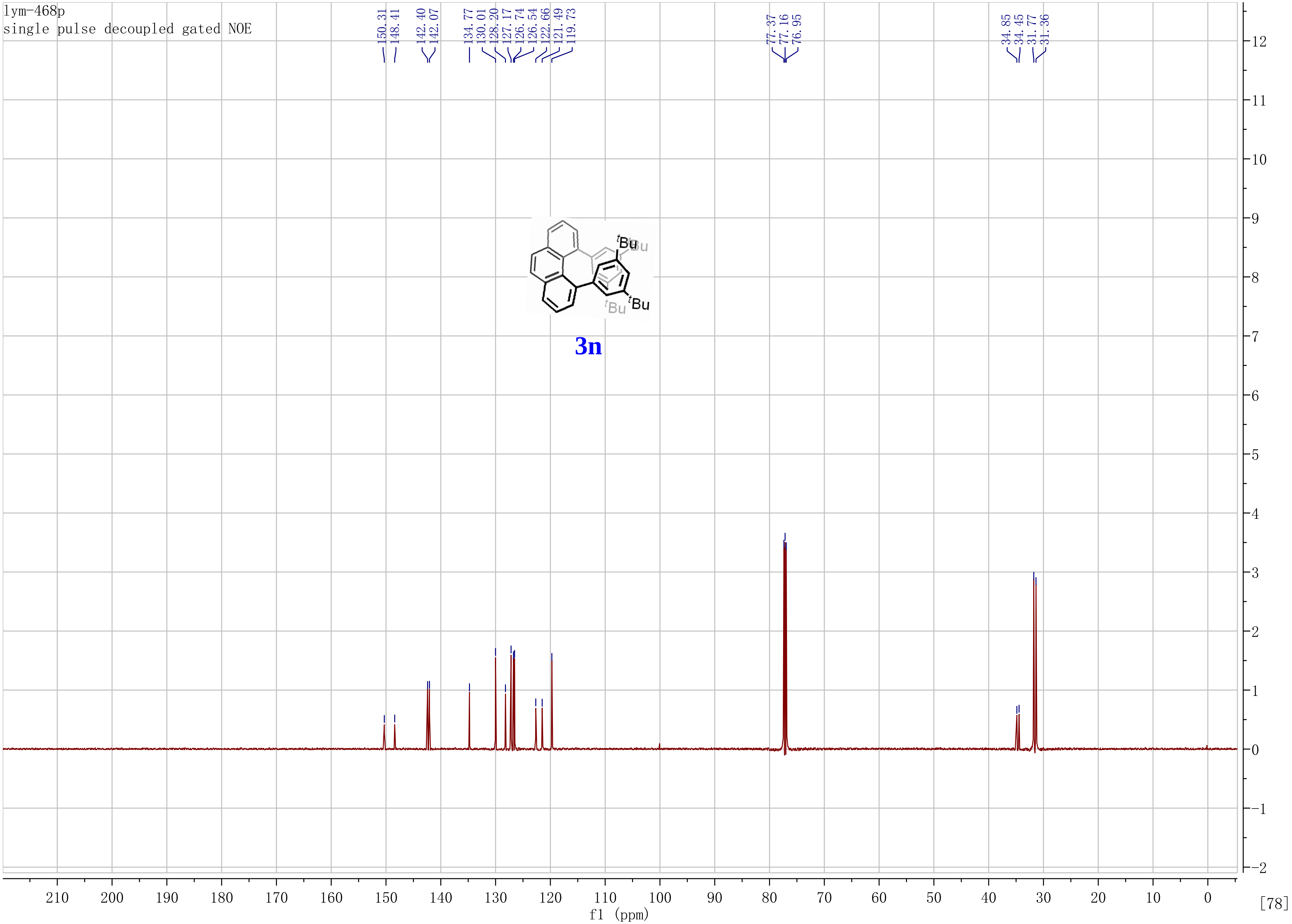

lym-468p
single pulse decoupled gated NOE
150.31
148.41
142.40
142.07
134.77
130.01
128.20
127.17
126.74
126.54
122.66
121.49
119.73
77.37
77.16
76.95
34.85
34.45
31.77
31.36
tBu
tBu
tBu
tBu
f1 (ppm)

3n

[78]

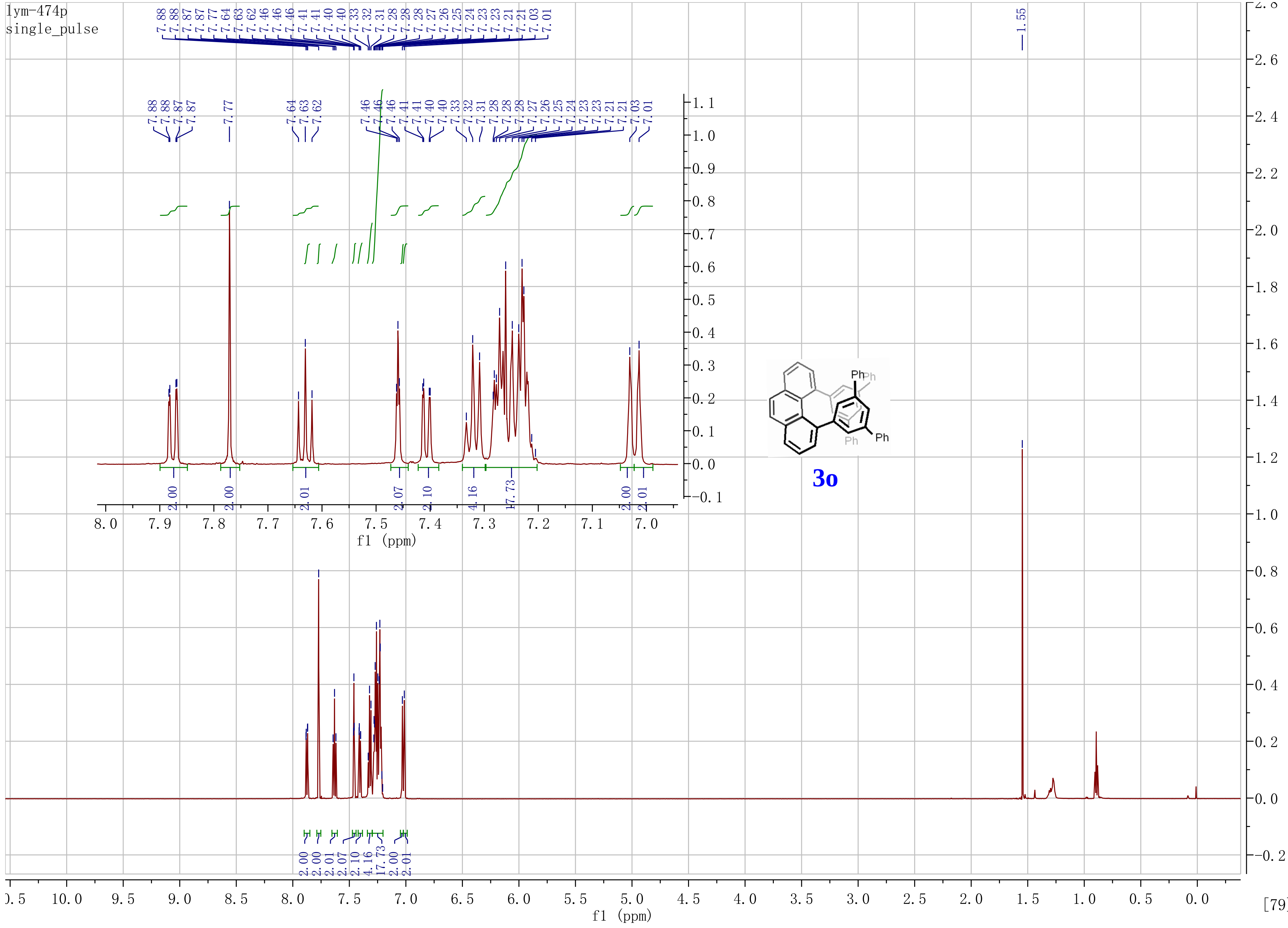
lym-474p
single_pulse
7.88 7.88 7.87 7.87 7.77 7.64 7.63 7.62 7.46 7.46 7.46 7.41 7.41 7.40 7.40 7.33 7.32 7.31 7.28 7.28 7.28 7.27 7.26 7.25 7.24 7.23 7.23 7.21 7.21 7.03 7.01
1.55
2.00 2.00 2.01 2.07 2.10 4.16 17.73 2.00 2.01
Ph
Ph
Ph
3o
f1 (ppm)
f1 (ppm)

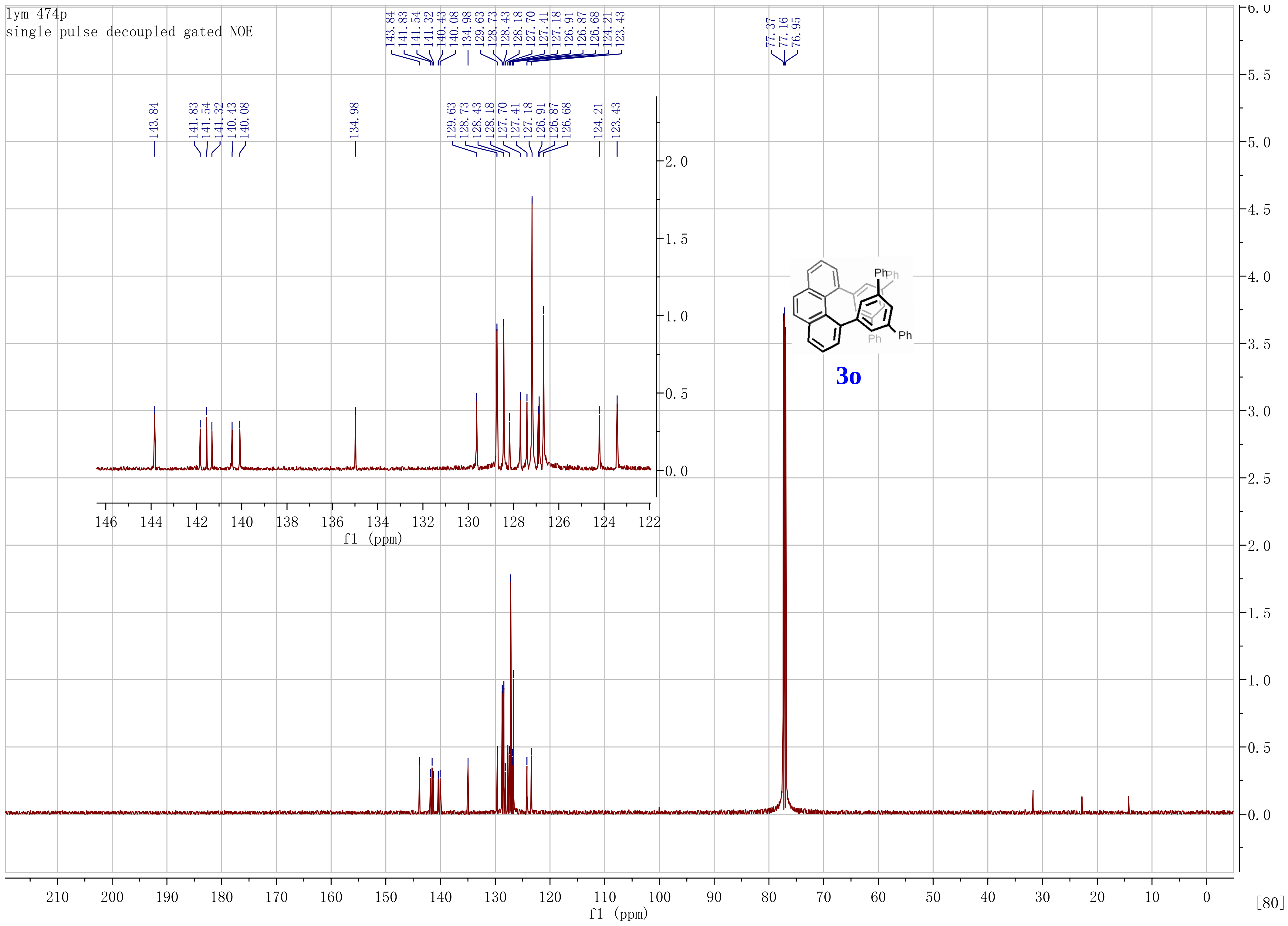
lym-474p
single pulse decoupled gated NOE
143.84
141.83
141.54
141.32
140.43
140.08
134.98
129.63
128.73
128.43
128.18
127.70
127.41
127.18
126.91
126.87
126.68
124.21
123.43
77.37
77.16
76.95
Ph
Ph
Ph
Ph
3o
f1 (ppm)
f1 (ppm)

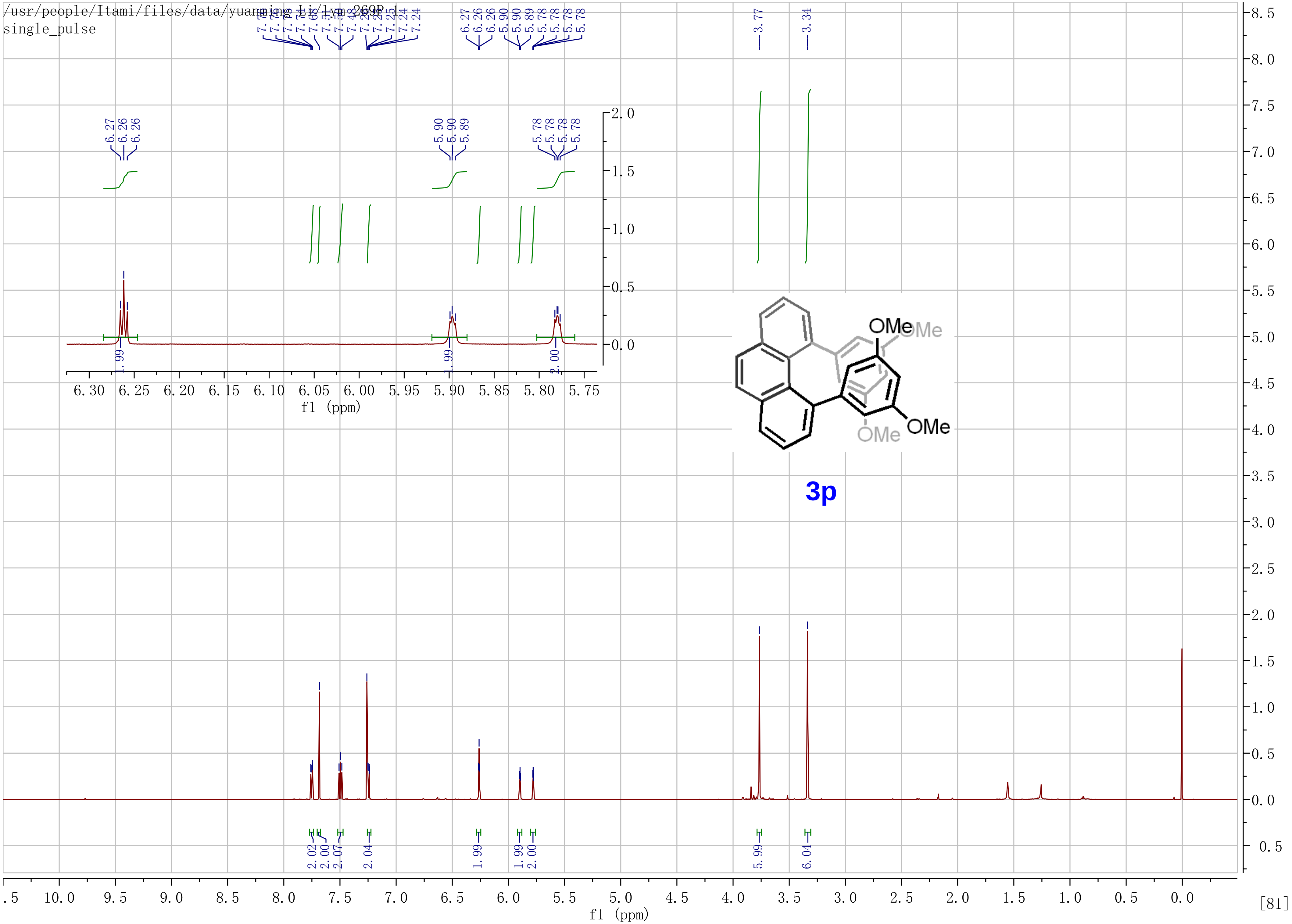
/usr/people/Itami/files/data/yuanming-Li/lym-269P-1
single_pulse
7.74 7.74 7.73 7.73 7.68 7.50 7.49 7.49 7.48 7.26 7.25 7.25 7.24 7.24
6.27 6.26 6.26 5.90 5.90 5.89 5.78 5.78 5.78 5.78 5.78
3.77
3.34
1.99
1.99
2.00
f1 (ppm)
2.02 2.00 2.07 2.04 1.99 1.99 2.00 5.99 6.04
f1 (ppm)
OMe
OMe
OMe
OMe

3p

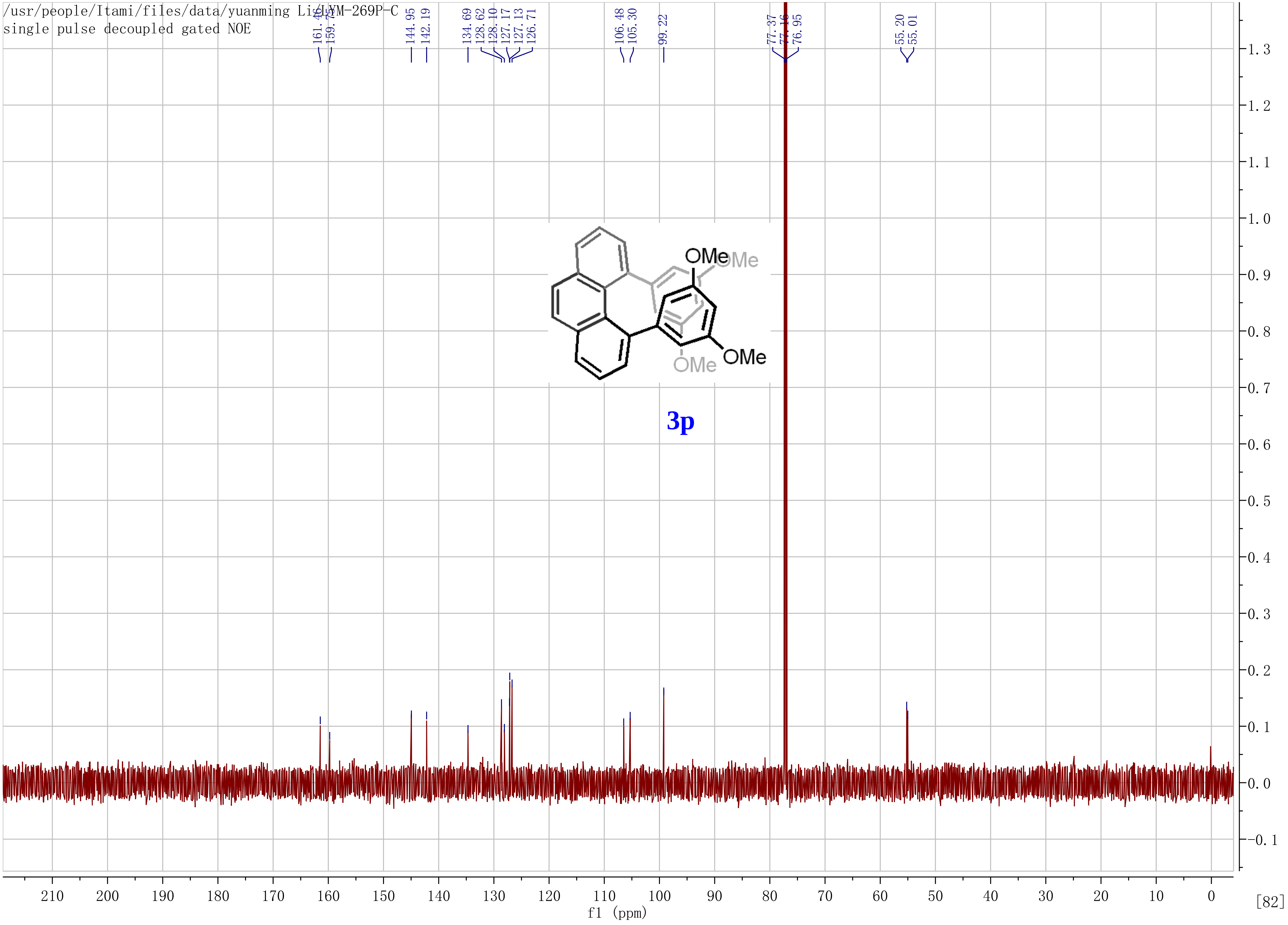
/usr/people/Itami/files/data/yuanming Li/LYM-269P-C
single pulse decoupled gated NOE
144.95
142.19
134.69
128.62
128.10
127.17
127.13
126.71
106.48
105.30
99.22
77.37
77.16
76.95
55.20
55.01
3p
f1 (ppm)

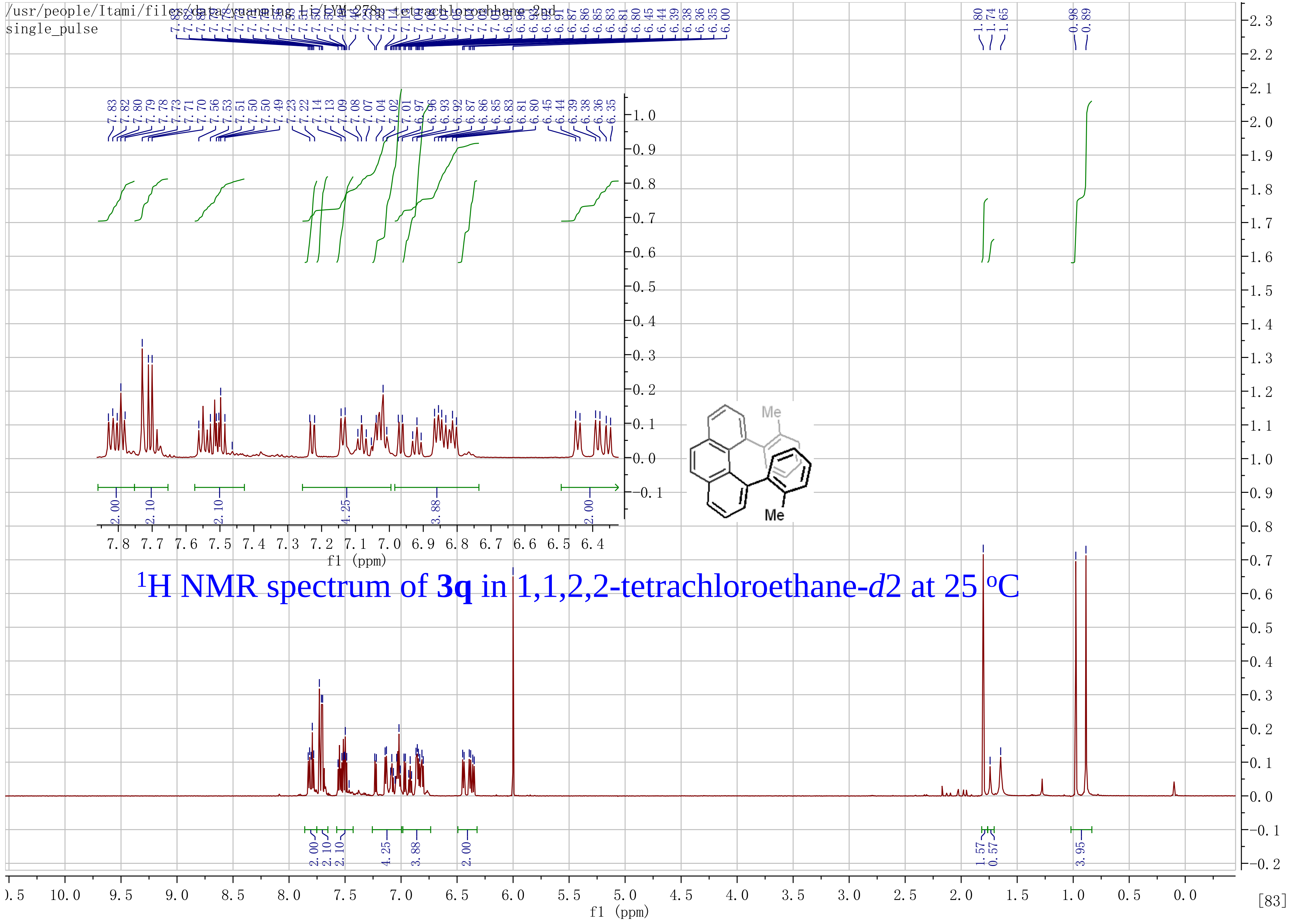

^{1}H NMR spectrum of **3q** in 1,1,2,2-tetrachloroethane-*d*2 at 25 °C

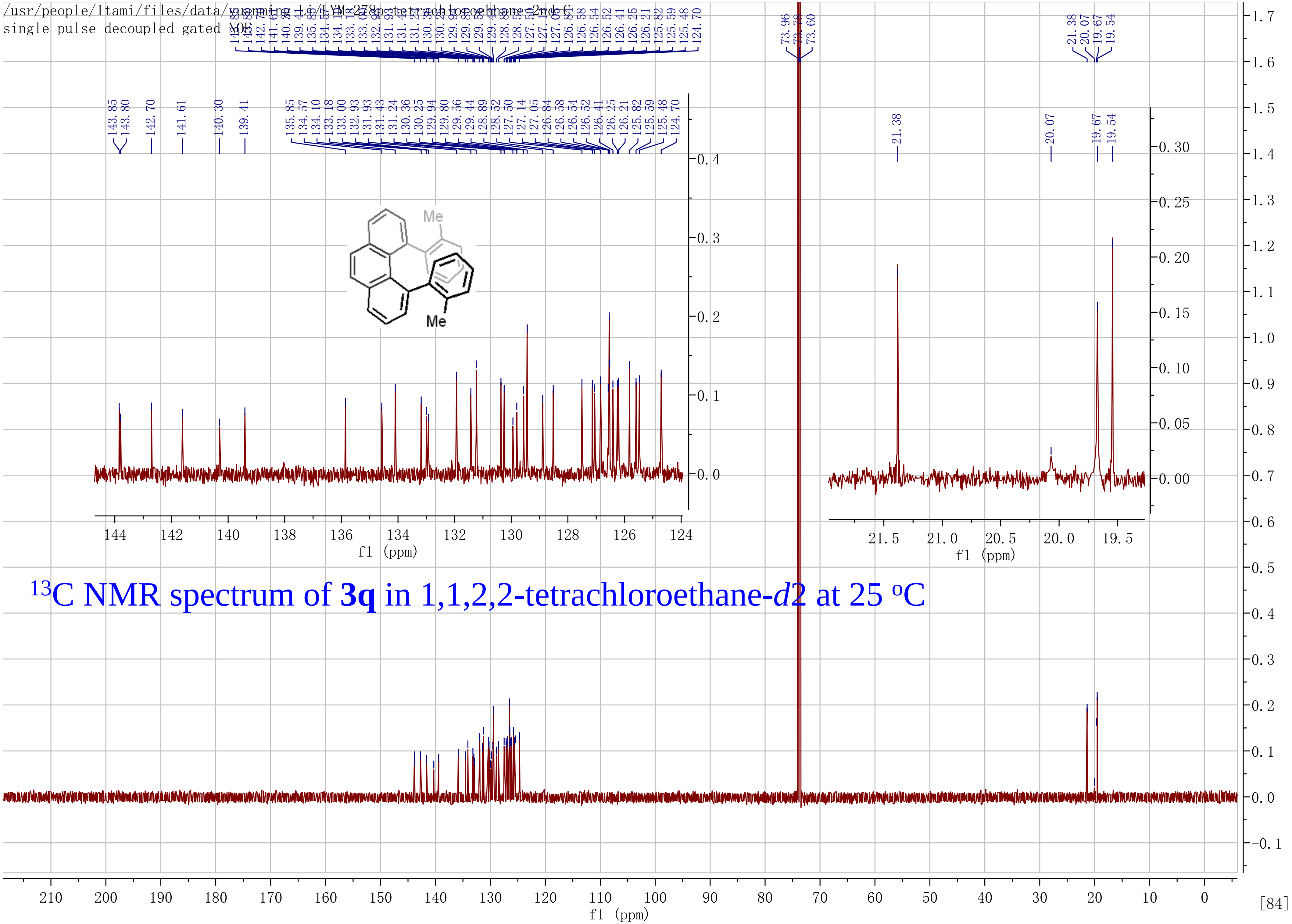

^{13}C NMR spectrum of **3q** in 1,1,2,2-tetrachloroethane-*d2* at 25 ^{o}C

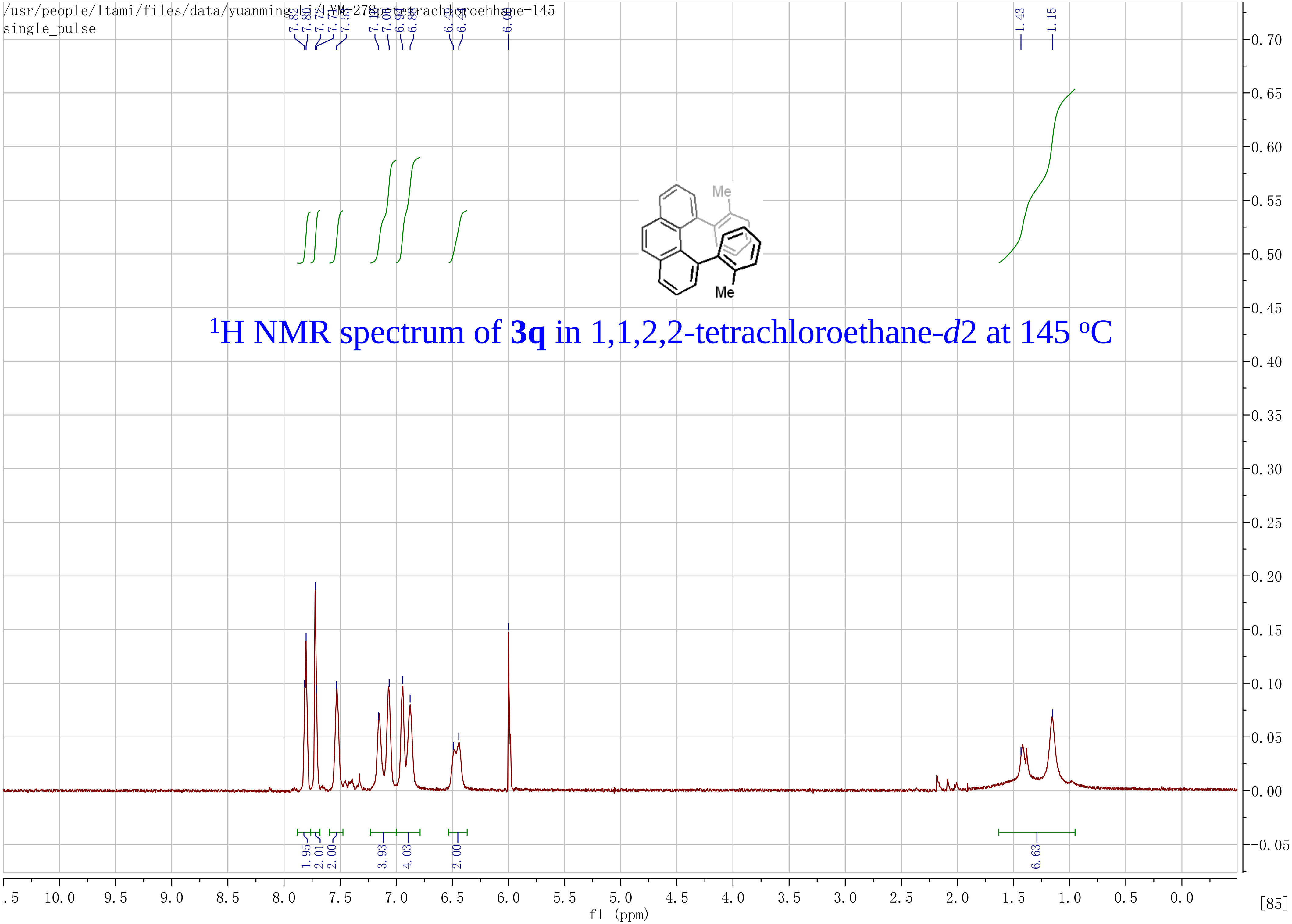

^{1}H NMR spectrum of **3q** in 1,1,2,2-tetrachloroethane-*d*2 at 145 °C

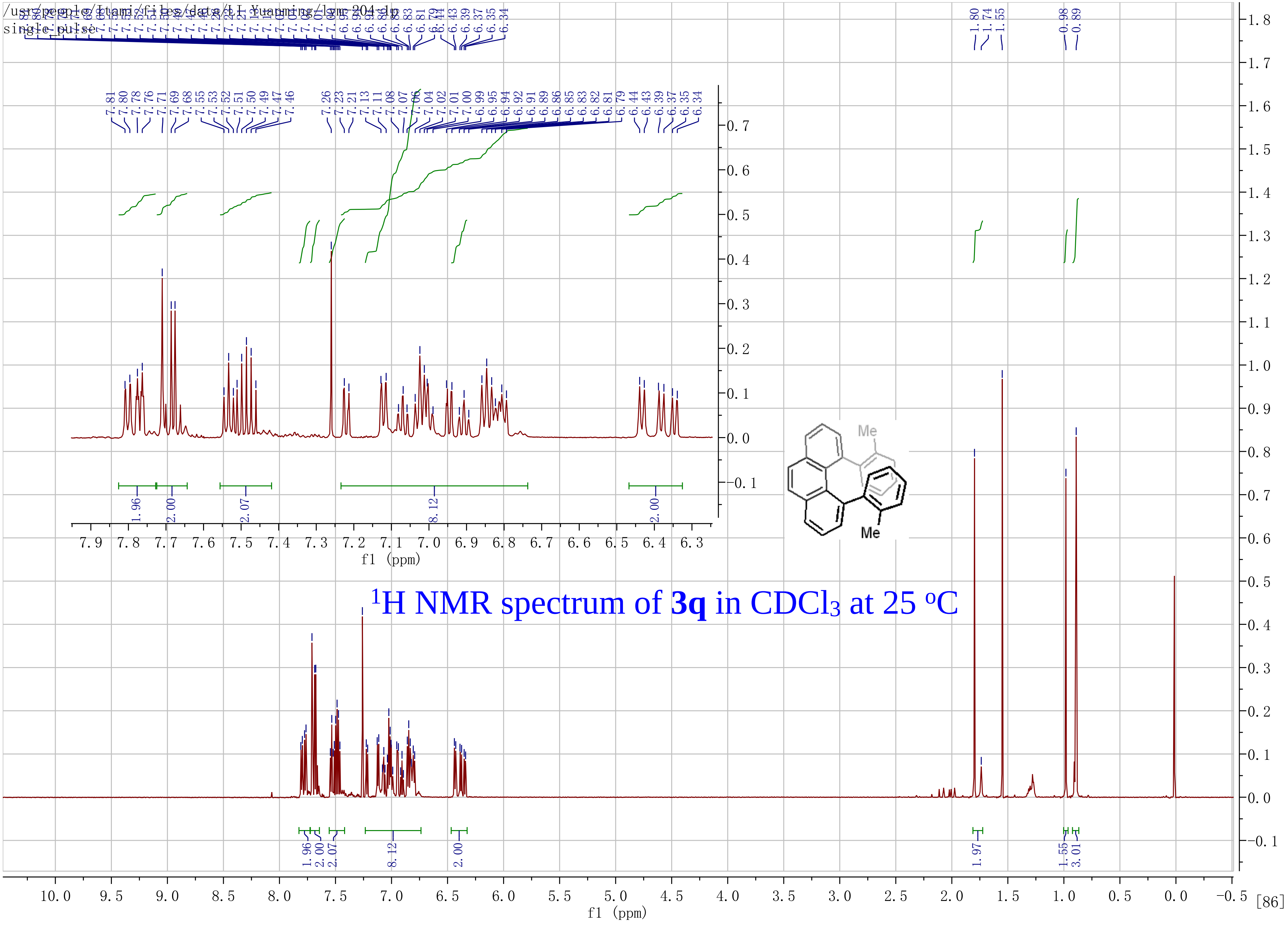

^{1}H NMR spectrum of **3q** in $CDCl_3$ at 25 ^{o}C

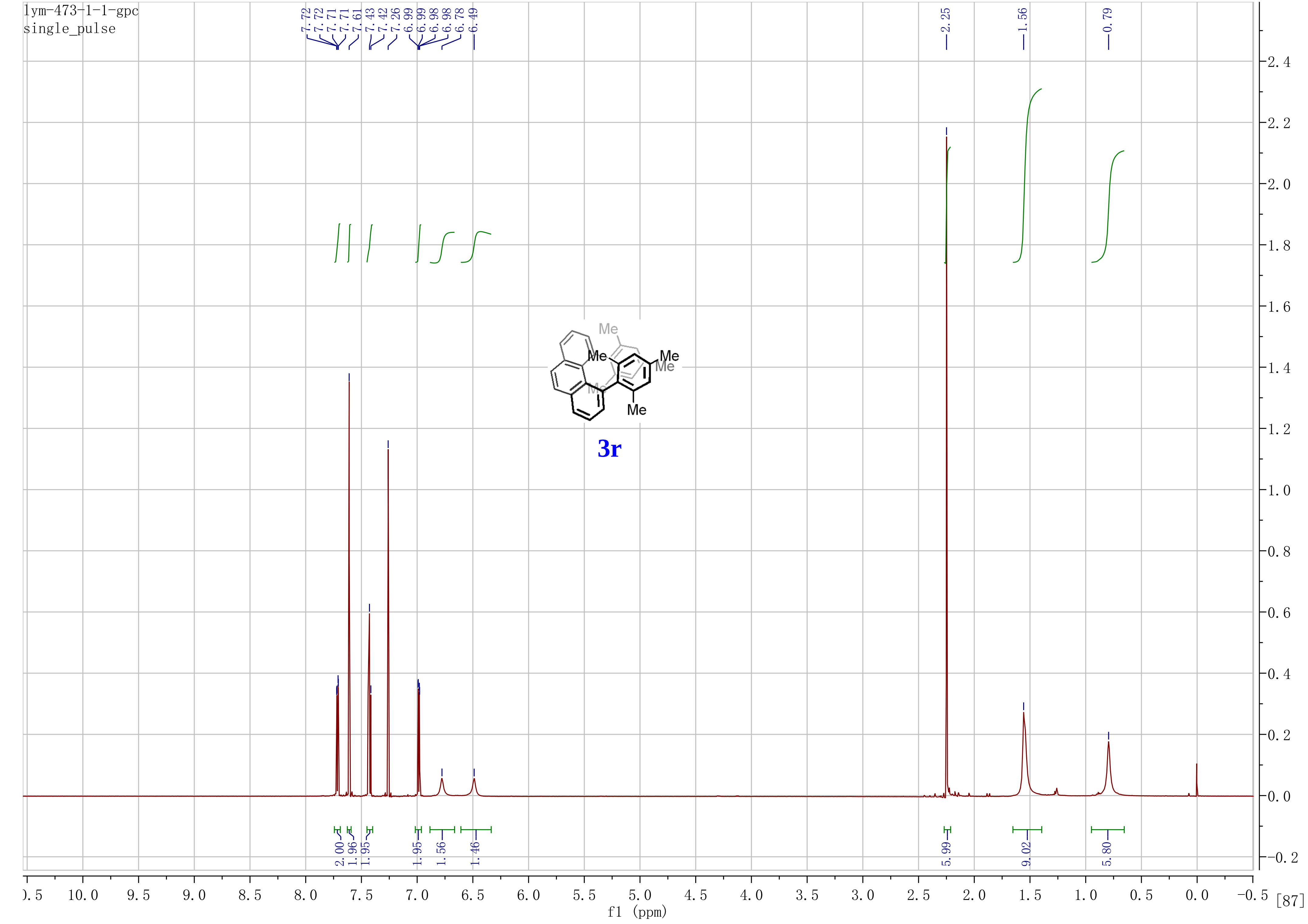
lym-473-1-1-gpc
single_pulse
7.72
7.72
7.71
7.71
7.61
7.43
7.42
7.26
6.99
6.99
6.98
6.98
6.78
6.49
2.25
1.56
0.79
Me
Me
Me
Me
Me
3r
2.00
1.96
1.95
1.95
1.56
1.46
5.99
9.02
5.80
f1 (ppm)

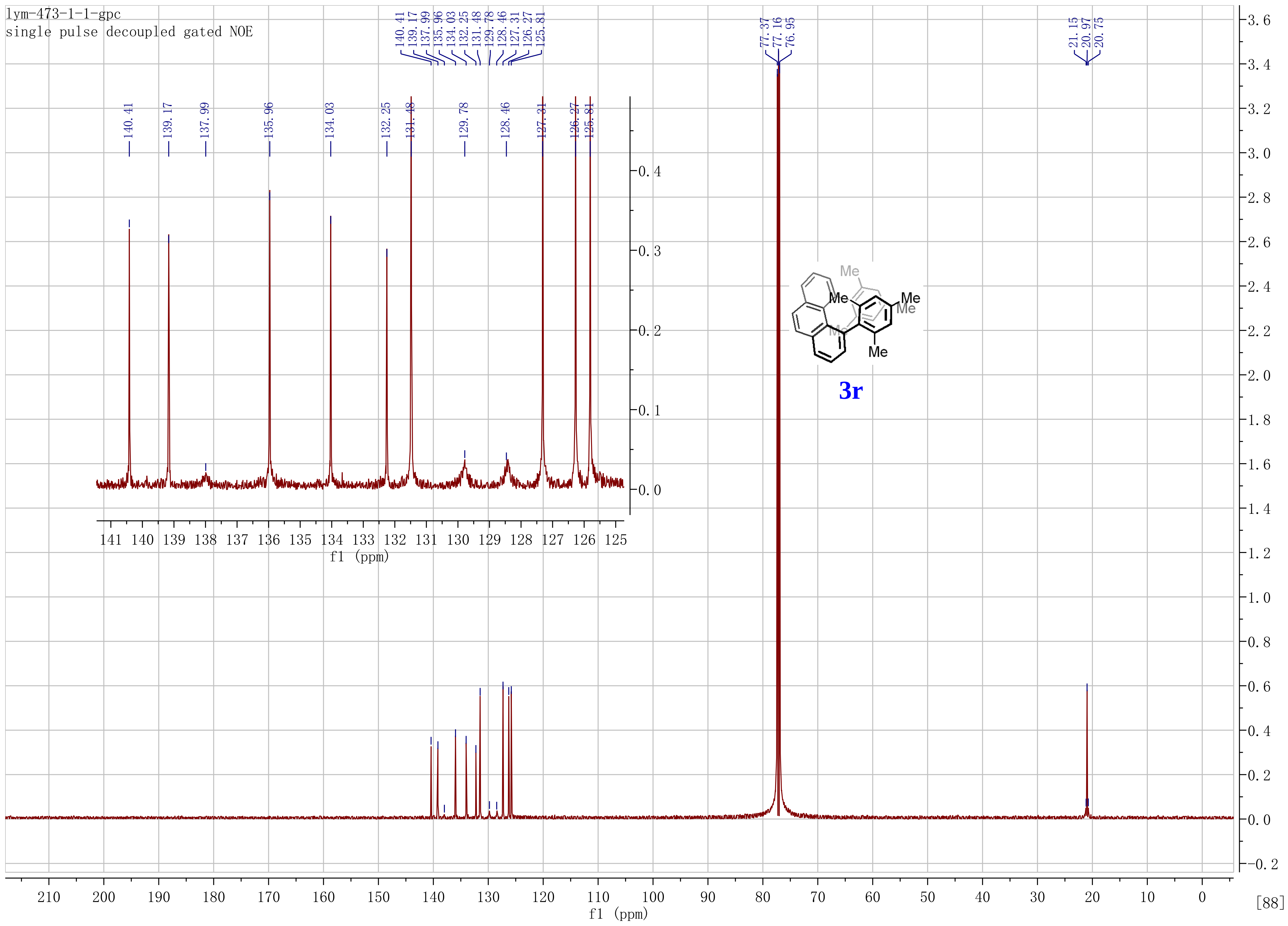

lym-473-1-1-gpc
single pulse decoupled gated NOE
140.41
139.17
137.99
135.96
134.03
132.25
131.48
129.78
128.46
127.31
126.27
125.81
77.37
77.16
76.95
21.15
20.97
20.75
Me
Me
Me
Me
Me
Me
3r
f1 (ppm)
f1 (ppm)

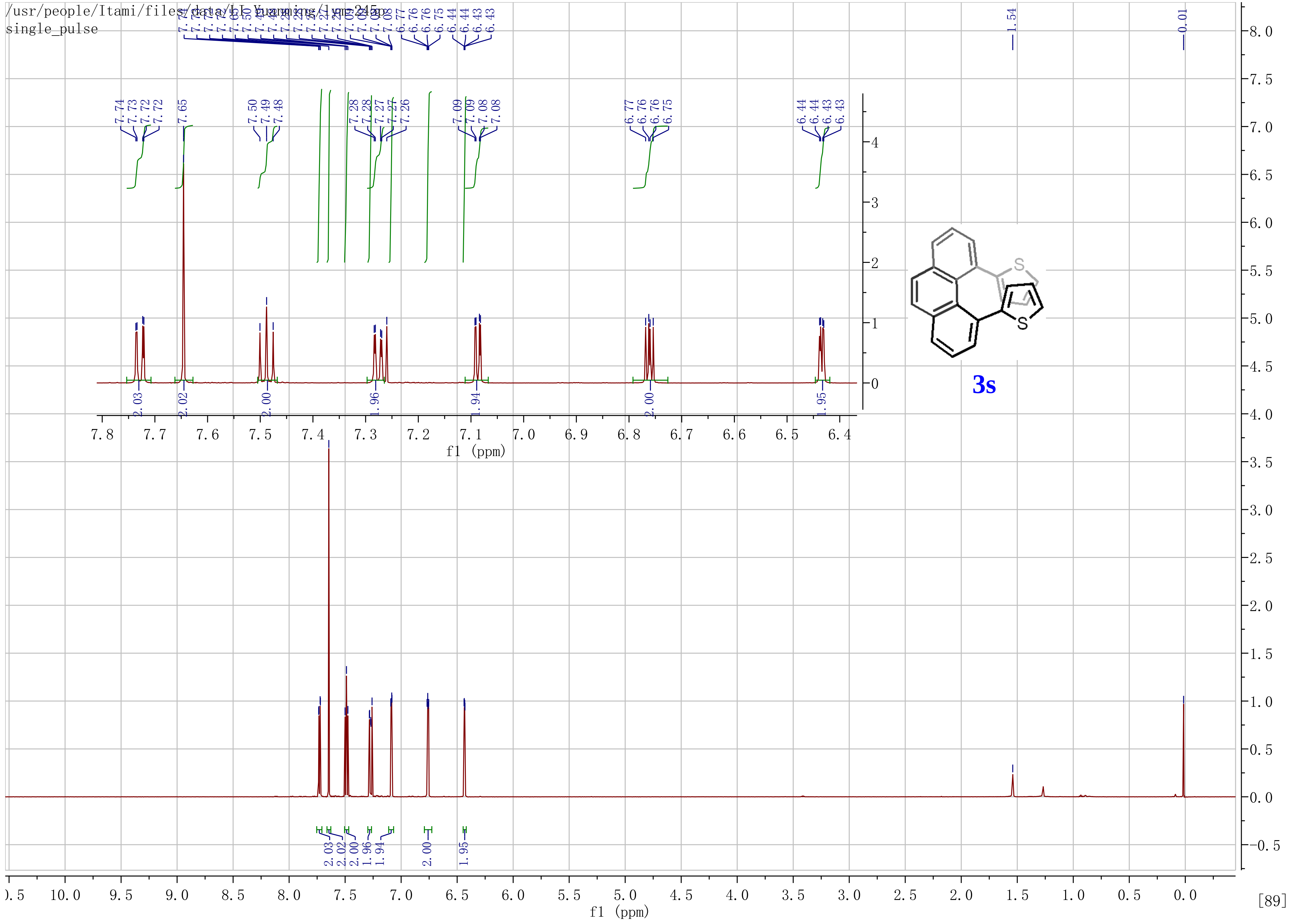
/usr/people/Itami/files/data/LI-Yuanming/lym-245
single_pulse
1.54
0.01
7.74
7.73
7.72
7.72
7.65
7.50
7.49
7.48
7.28
7.28
7.27
7.27
7.26
7.09
7.09
7.08
7.08
6.77
6.76
6.76
6.75
6.44
6.44
6.43
6.43
2.03
2.02
2.00
1.96
1.94
2.00
1.95
f1 (ppm)
f1 (ppm)
S
S

3s

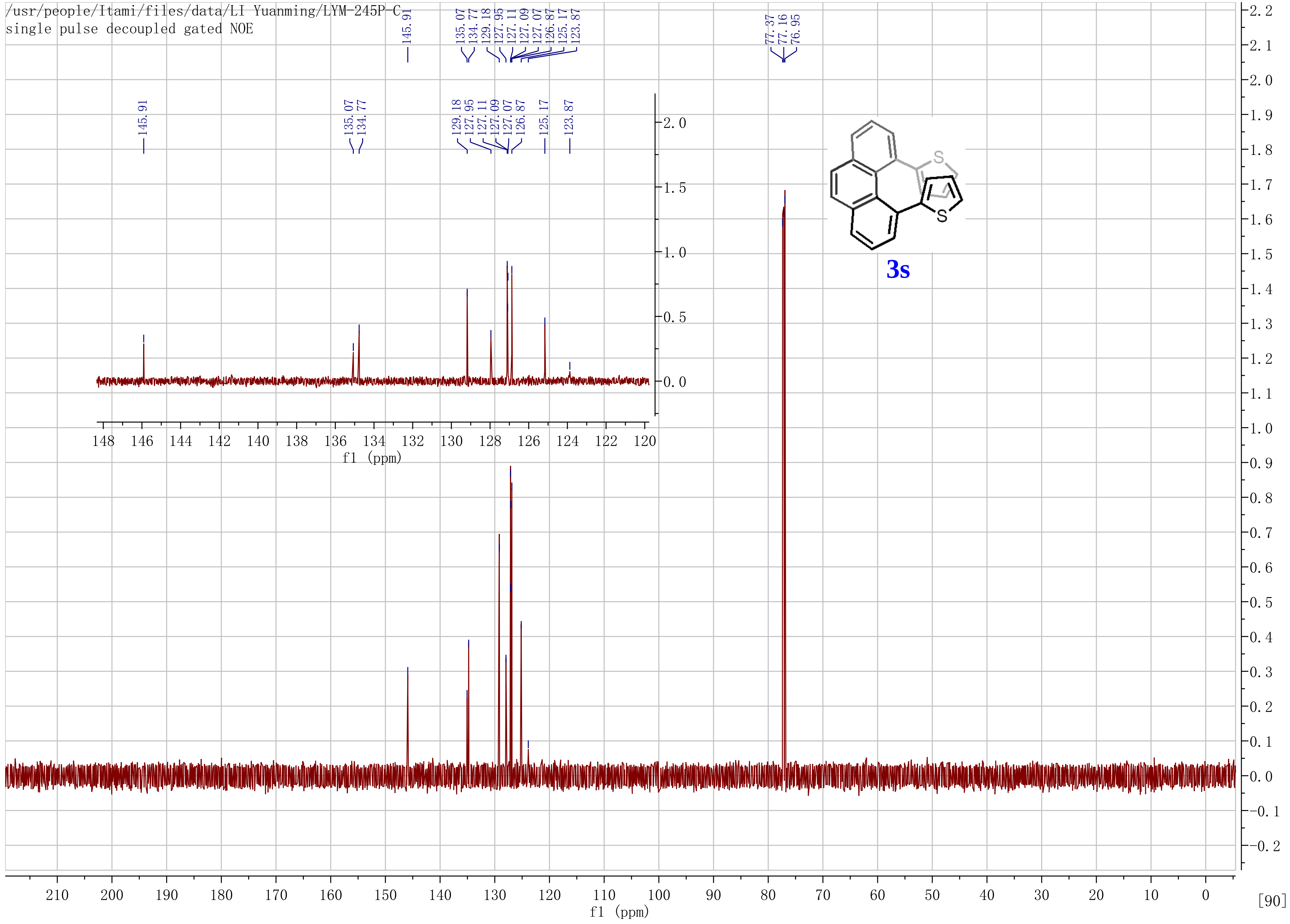
/usr/people/Itami/files/data/LI Yuanming/LYM-245P-C
single pulse decoupled gated NOE
145.91
135.07
134.77
129.18
127.95
127.11
127.09
127.07
126.87
125.17
123.87
77.37
77.16
76.95
S
S
3s
f1 (ppm)
f1 (ppm)

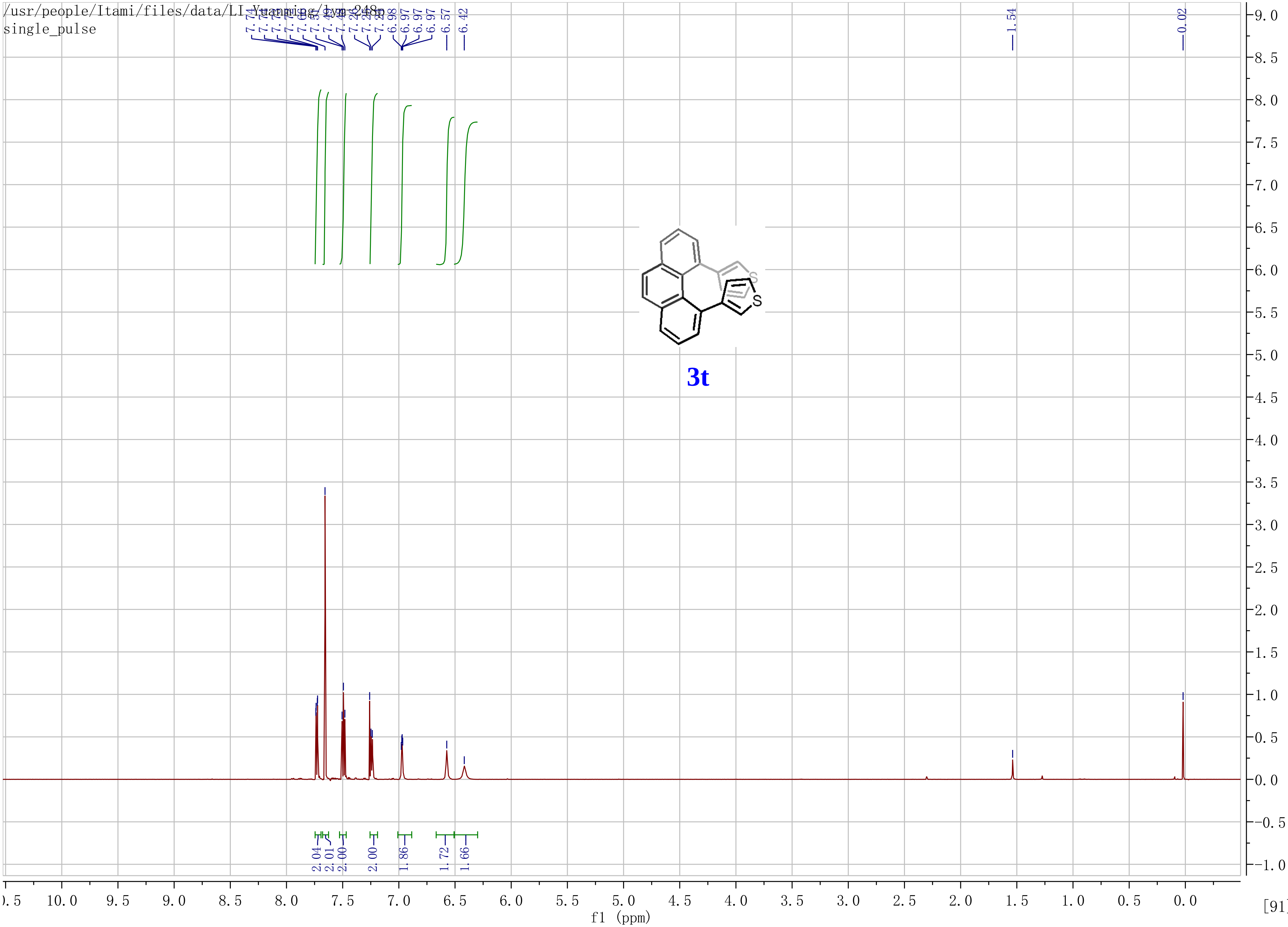
/usr/people/Itami/files/data/LI-Yuanming/lym-248n
single_pulse
6.98
6.97
6.97
6.97
6.57
6.42
1.54
0.02
3t
2.04
2.01
2.00
2.00
1.86
1.72
1.66
f1 (ppm)

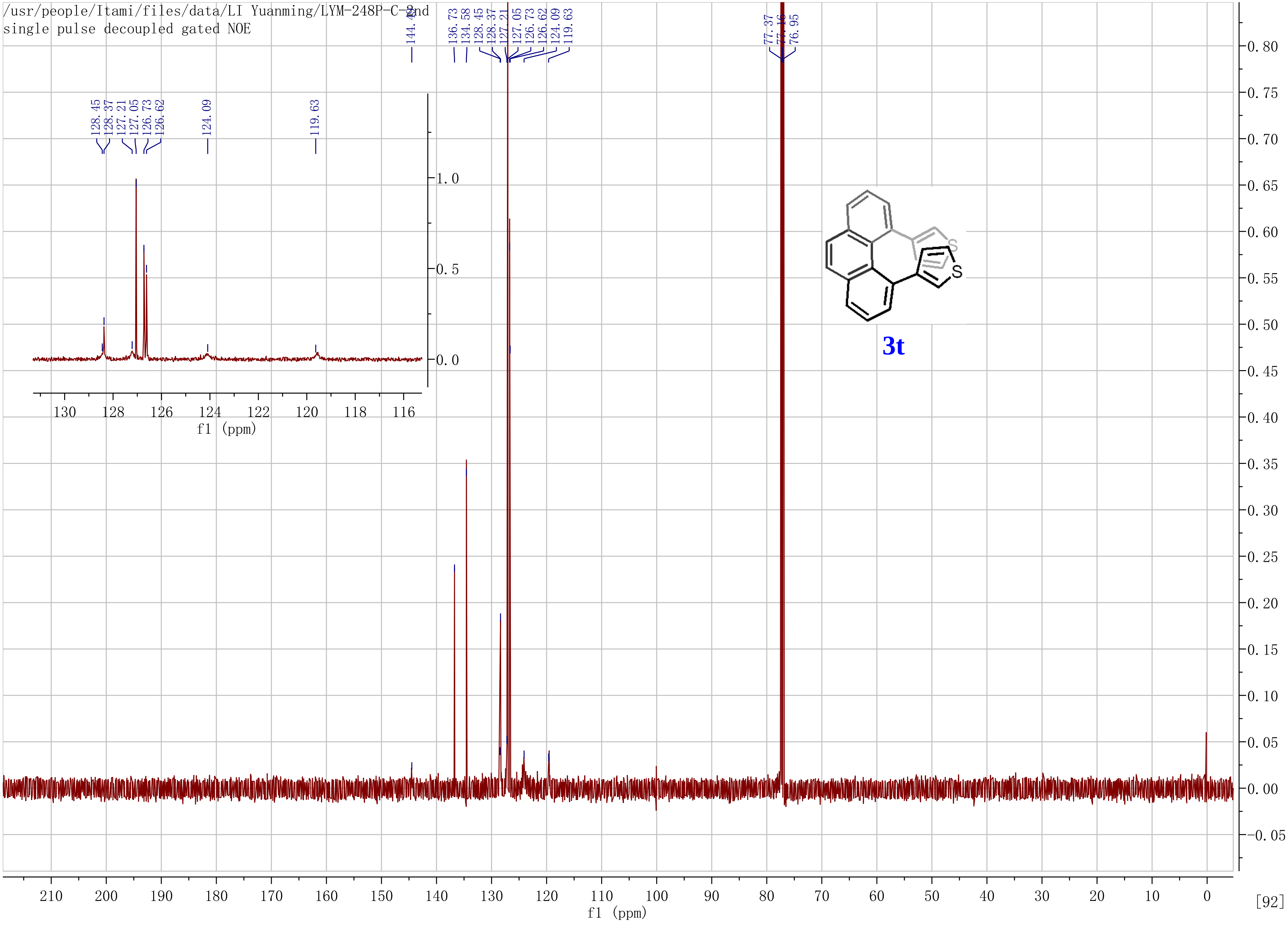

/usr/people/Itami/files/data/LI Yuanming/LYM-248P-C-2nd
single pulse decoupled gated NOE
144.49
136.73
134.58
128.45
128.37
127.21
127.05
126.73
126.62
124.09
119.63
77.37
77.16
76.95
3t
f1 (ppm)

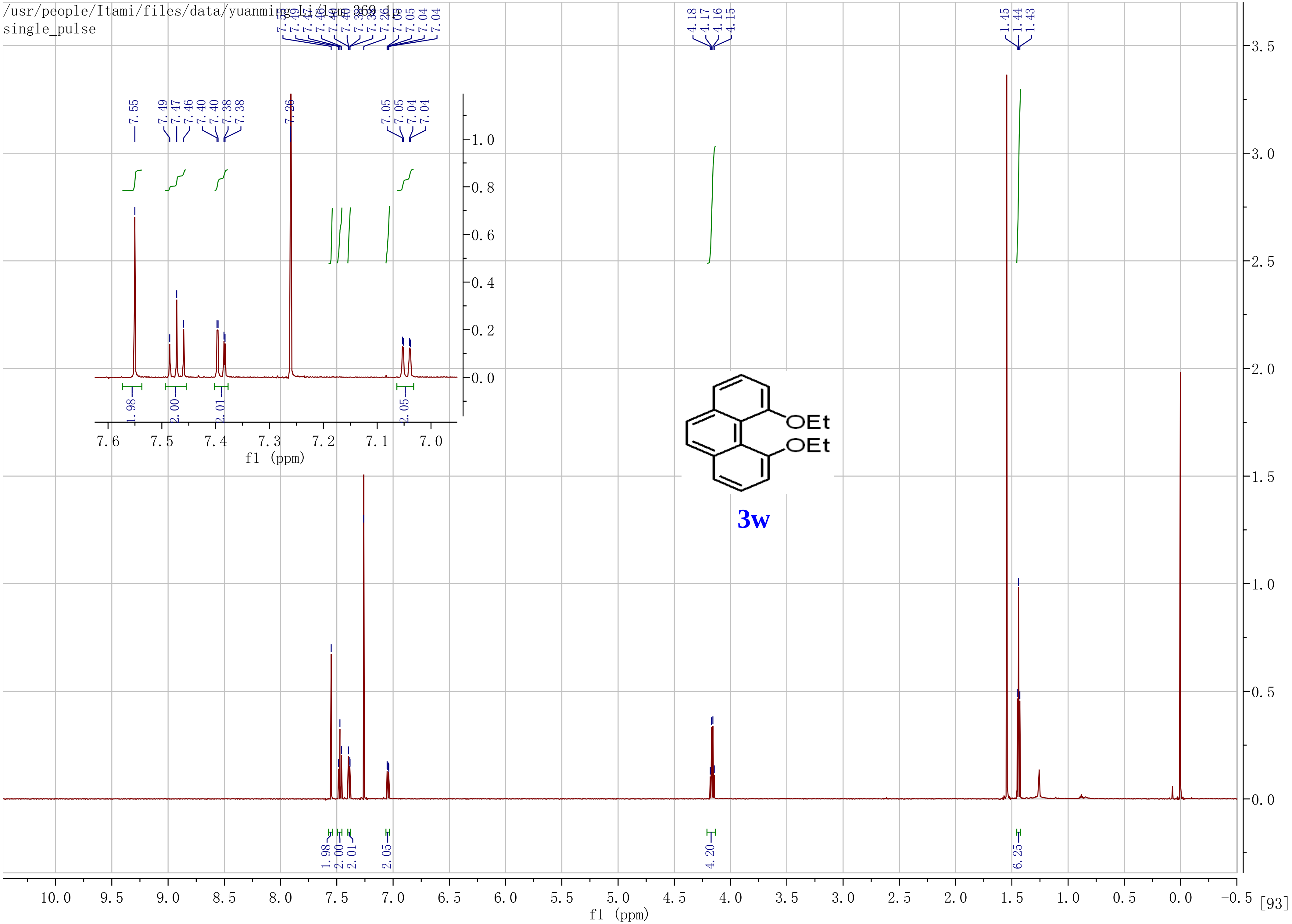

/usr/people/Itami/files/data/yuanming-Li/lym-369-1.jp
single_pulse
OEt
OEt
3w
f1 (ppm)

[93]

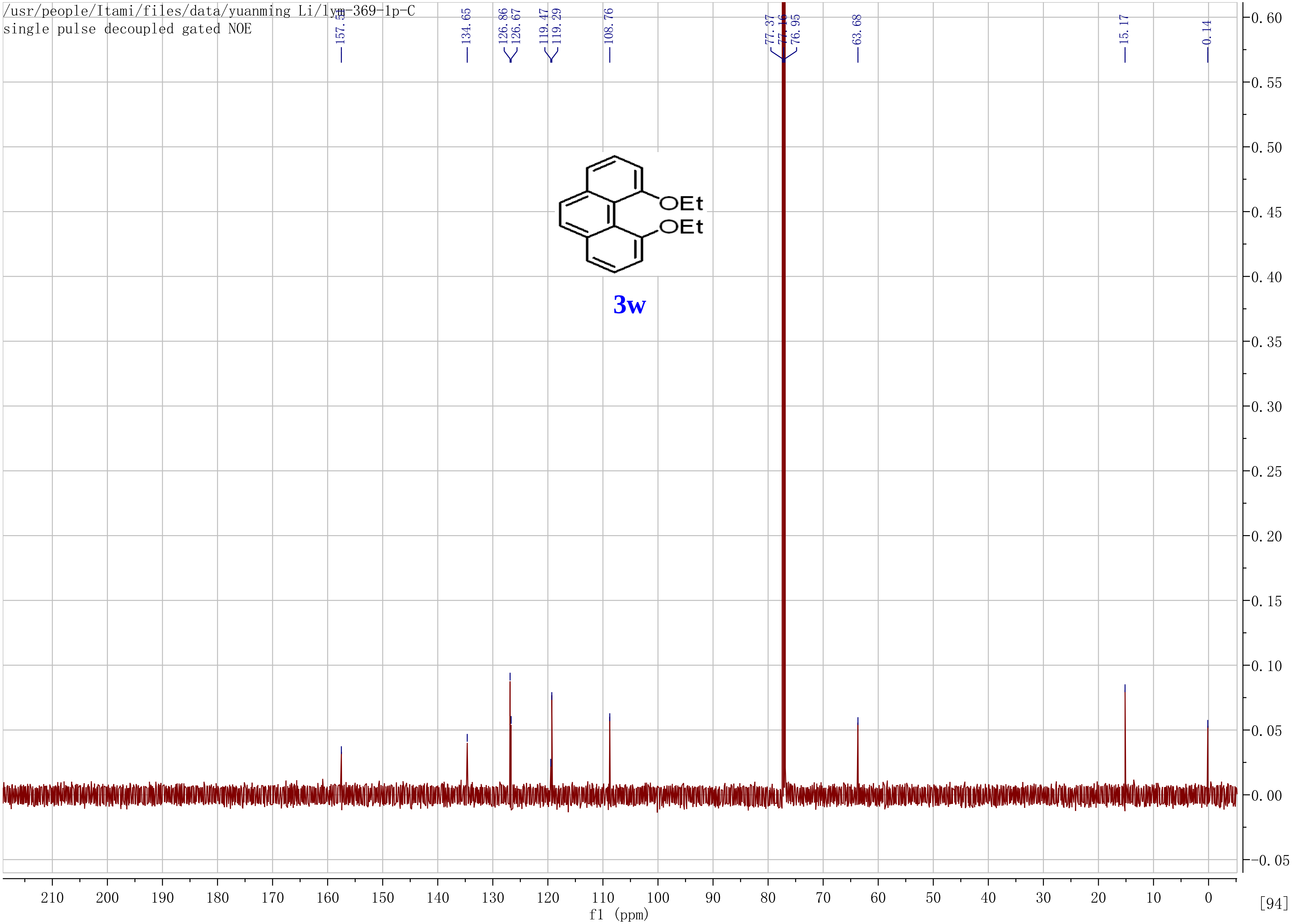
/usr/people/Itami/files/data/yuanming Li/lym-369-1p-C
single pulse decoupled gated NOE
157.51
134.65
126.86
126.67
119.47
119.29
108.76
77.37
77.16
76.95
63.68
15.17
0.14
OEt
OEt
3w
210 200 190 180 170 160 150 140 130 120 110 100 90 80 70 60 50 40 30 20 10 0
f1 (ppm)
0.60 0.55 0.50 0.45 0.40 0.35 0.30 0.25 0.20 0.15 0.10 0.05 0.00 −0.05

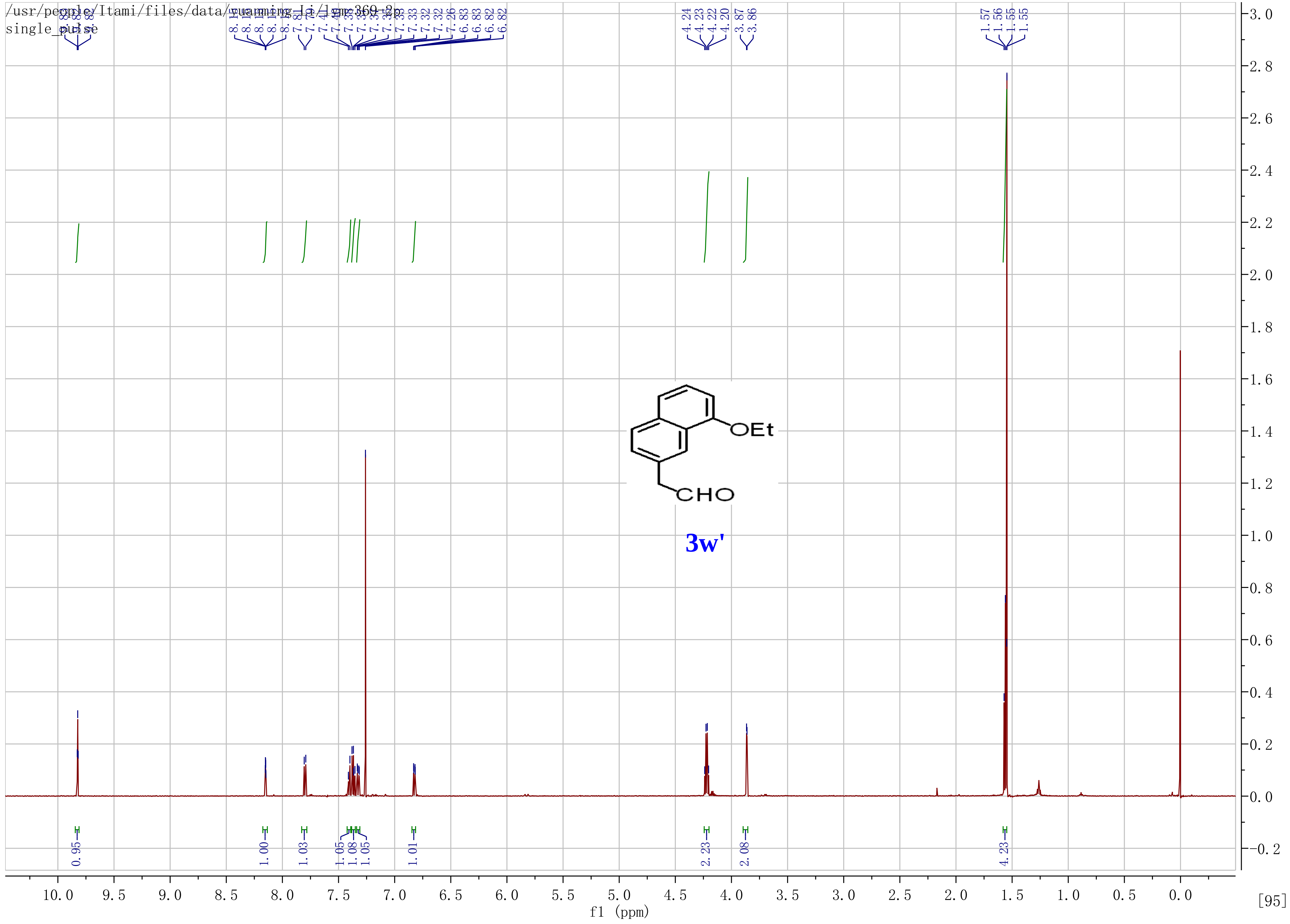

/usr/people/Itami/files/data/yuanming-li/lym-369-2p
single_pulse
OEt
CHO
3w'
f1 (ppm)

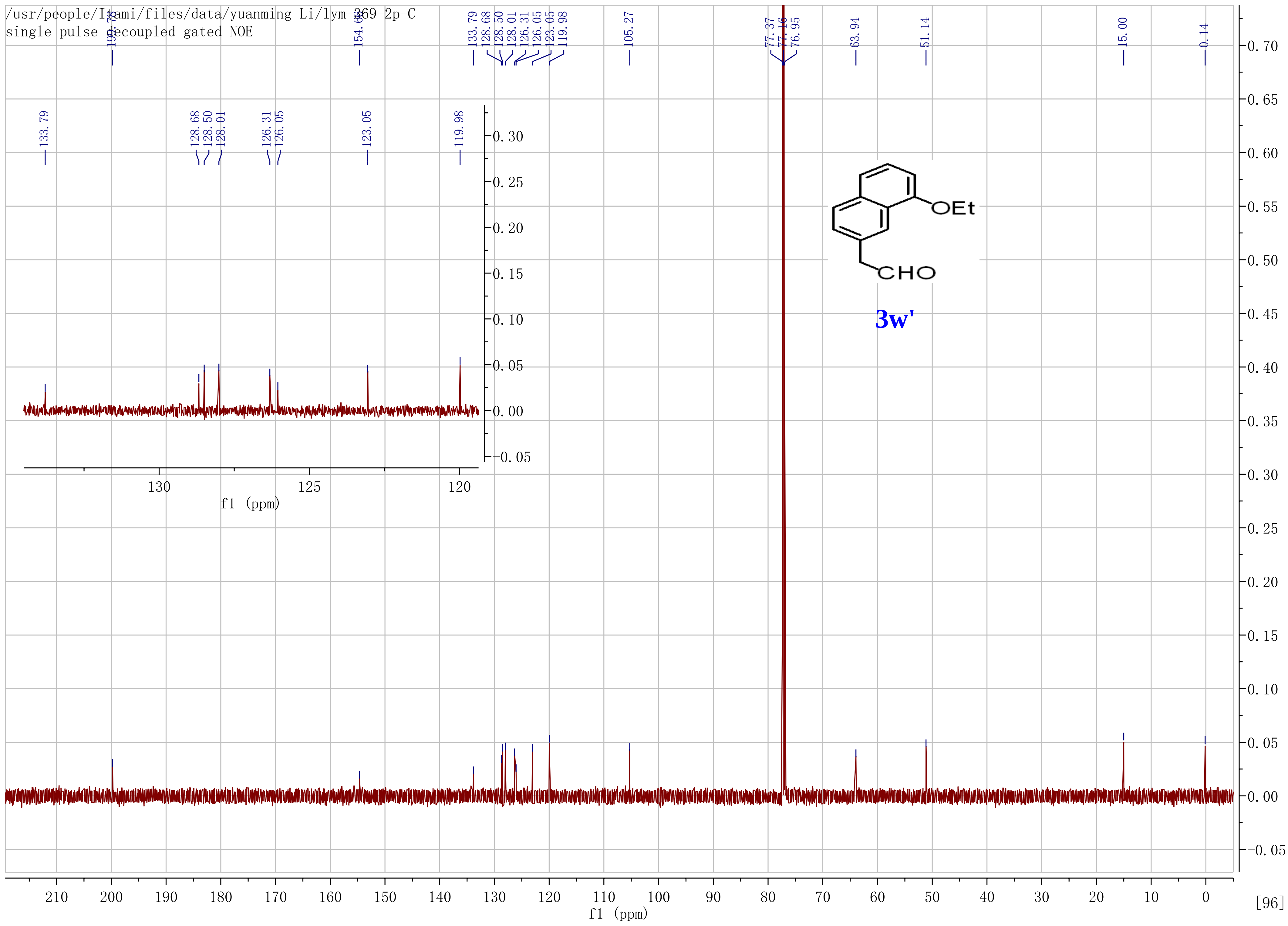
/usr/people/Itami/files/data/yuanming Li/lym-369-2p-C
single pulse decoupled gated NOE
199.78
154.69
133.79
128.68
128.50
128.01
126.31
126.05
123.05
119.98
105.27
77.37
77.16
76.95
63.94
51.14
15.00
0.14
OEt
CHO
3w'
f1 (ppm)

[96]

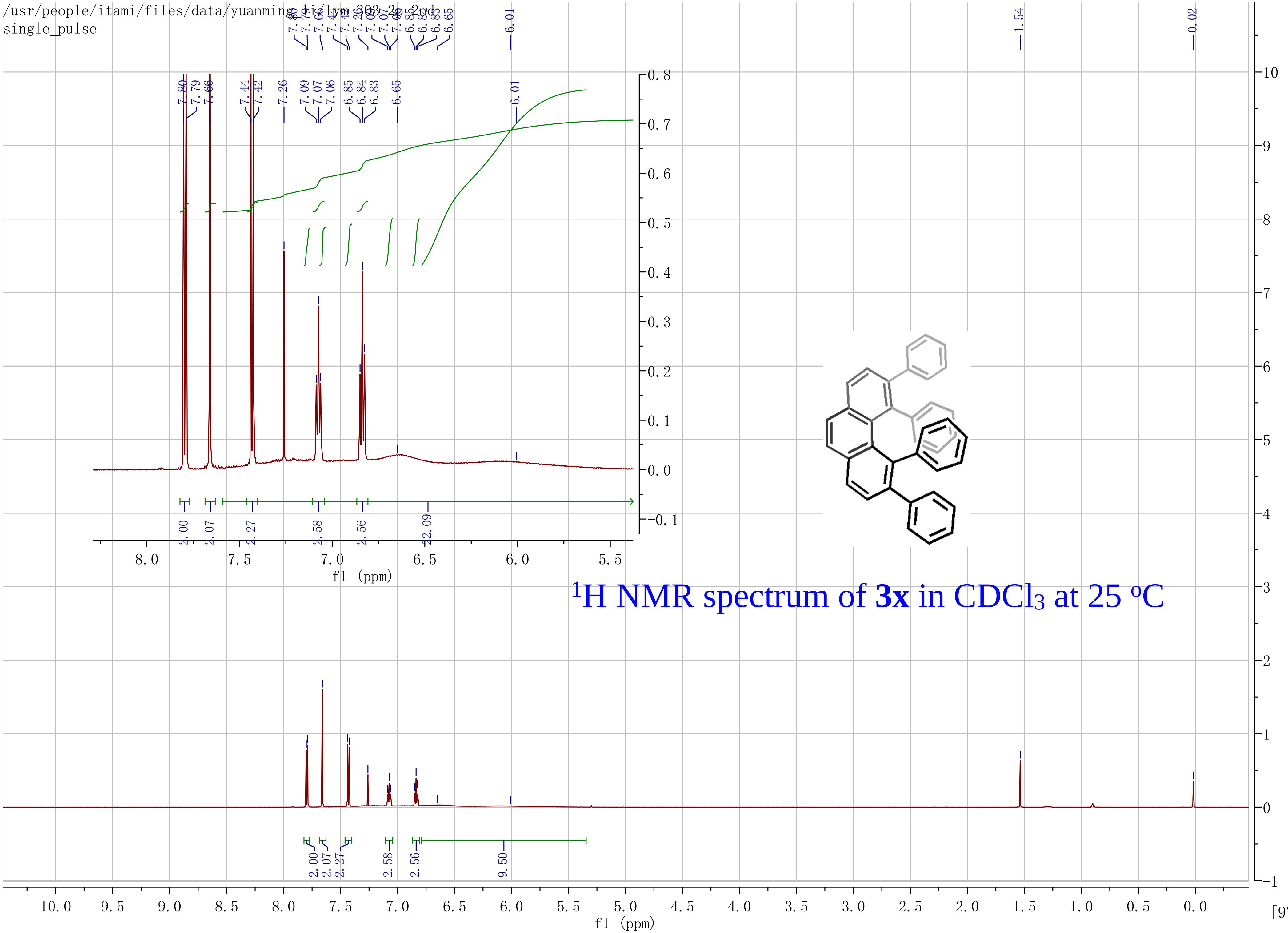

^{1}H NMR spectrum of **3x** in $CDCl_3$ at 25 ^{o}C

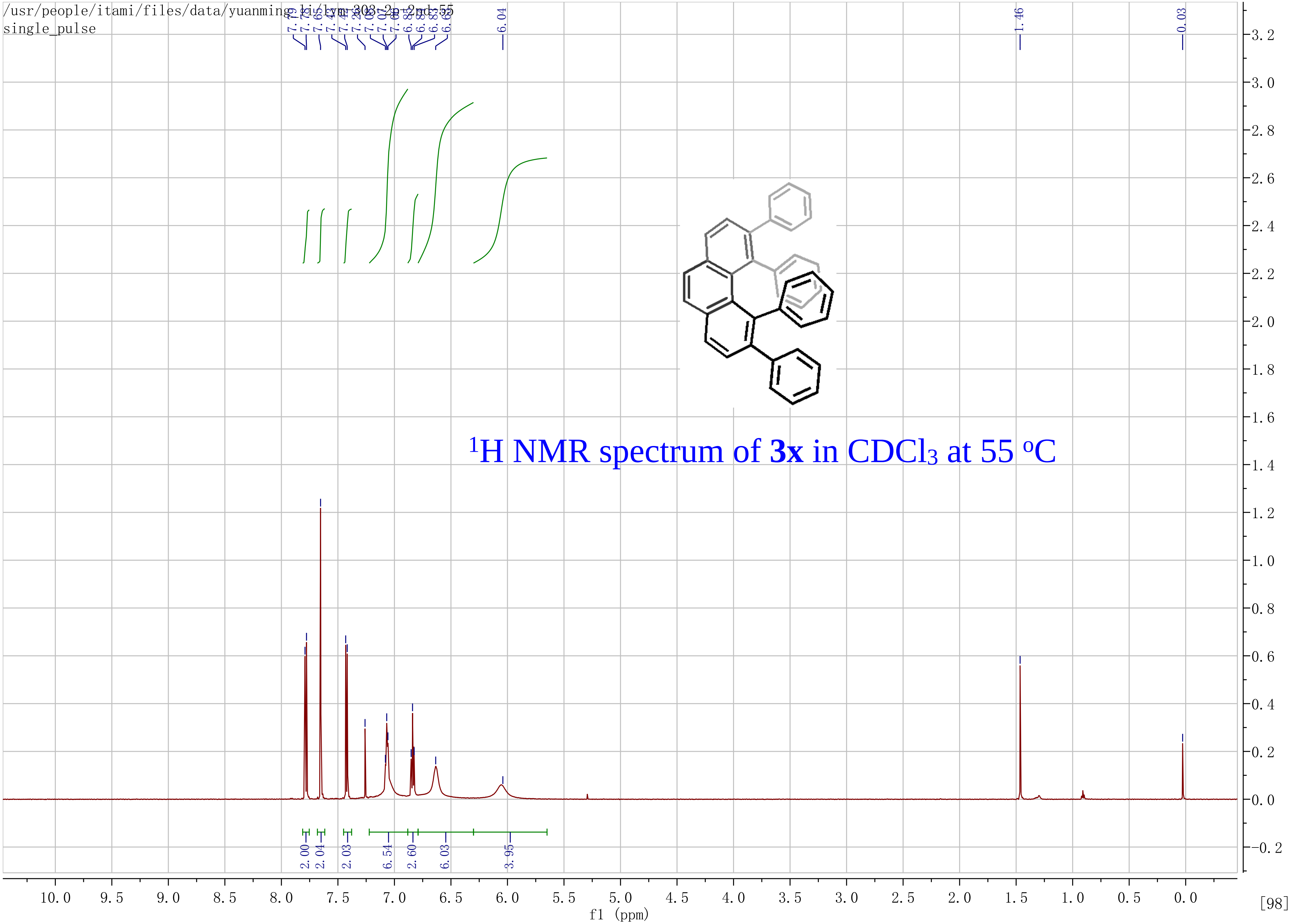

/usr/people/itami/files/data/yuanming
single_pulse
6.04
1.46
0.03
^{1}H NMR spectrum of **3x** in $CDCl_3$ at 55 ^oC
2.00
2.04
2.03
6.54
2.60
6.03
3.95
f1 (ppm)

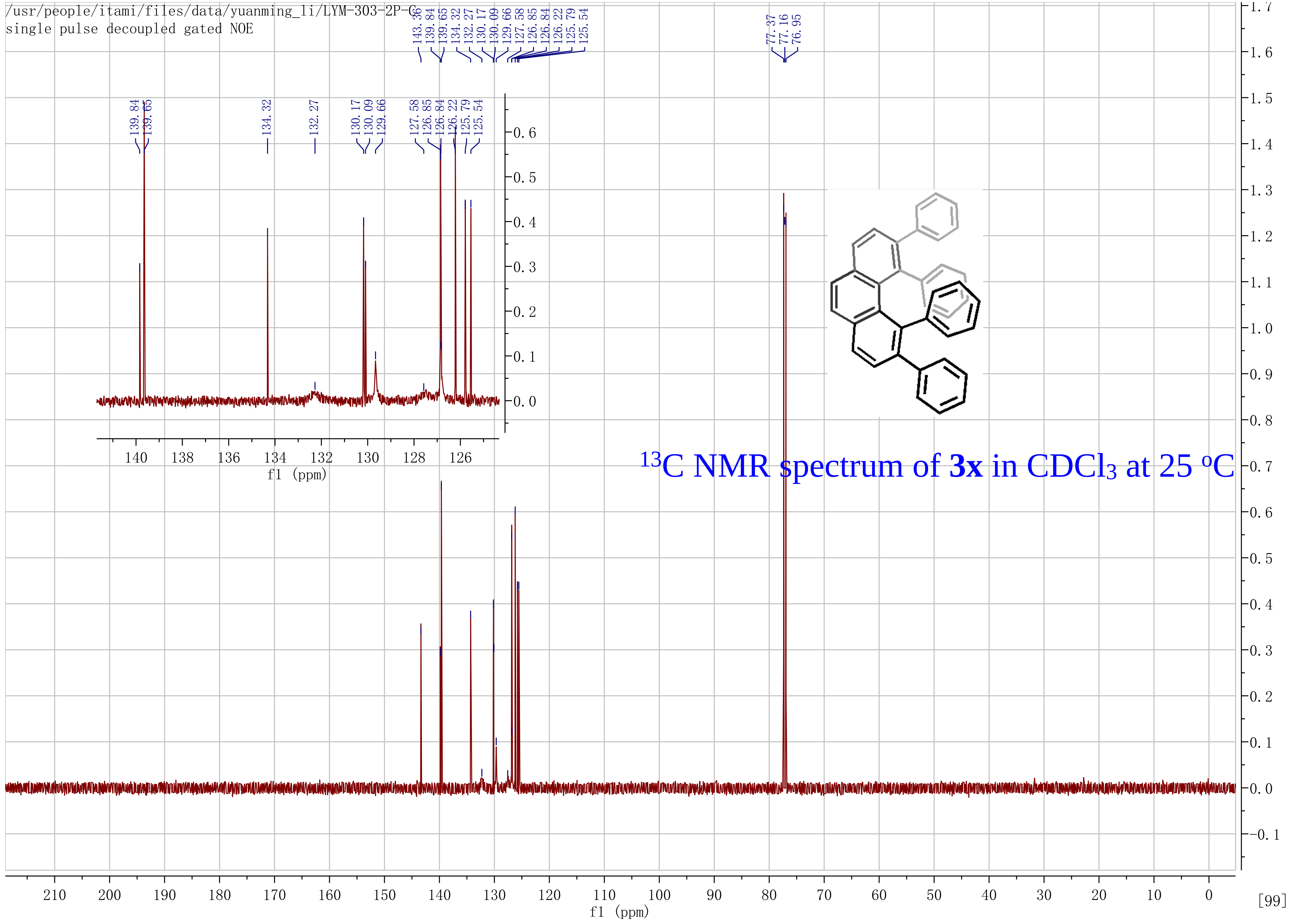

^{13}C NMR spectrum of **3x** in $CDCl_3$ at 25 °C

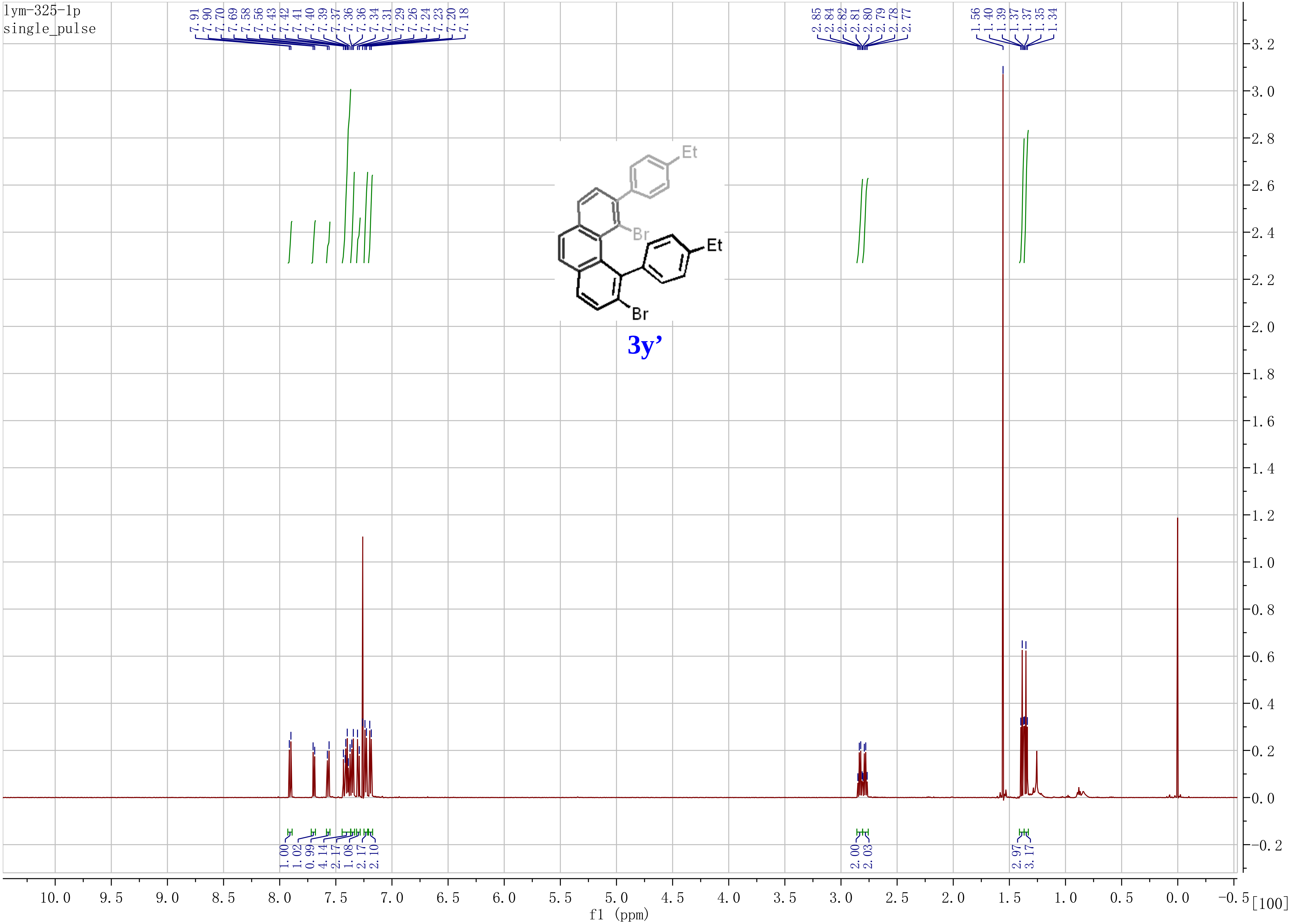

lym-325-1p
single_pulse
7.91 7.90 7.70 7.69 7.58 7.56 7.43 7.42 7.41 7.40 7.39 7.37 7.36 7.36 7.34 7.31 7.29 7.26 7.24 7.23 7.20 7.18
2.85 2.84 2.82 2.81 2.80 2.79 2.78 2.77
1.56 1.40 1.39 1.37 1.37 1.35 1.34
Et
Br
Et
Br
3y'
1.00 1.02 0.99 4.14 2.17 1.08 2.17 2.10
2.00 2.03
2.97 3.17
f1 (ppm)
[100]

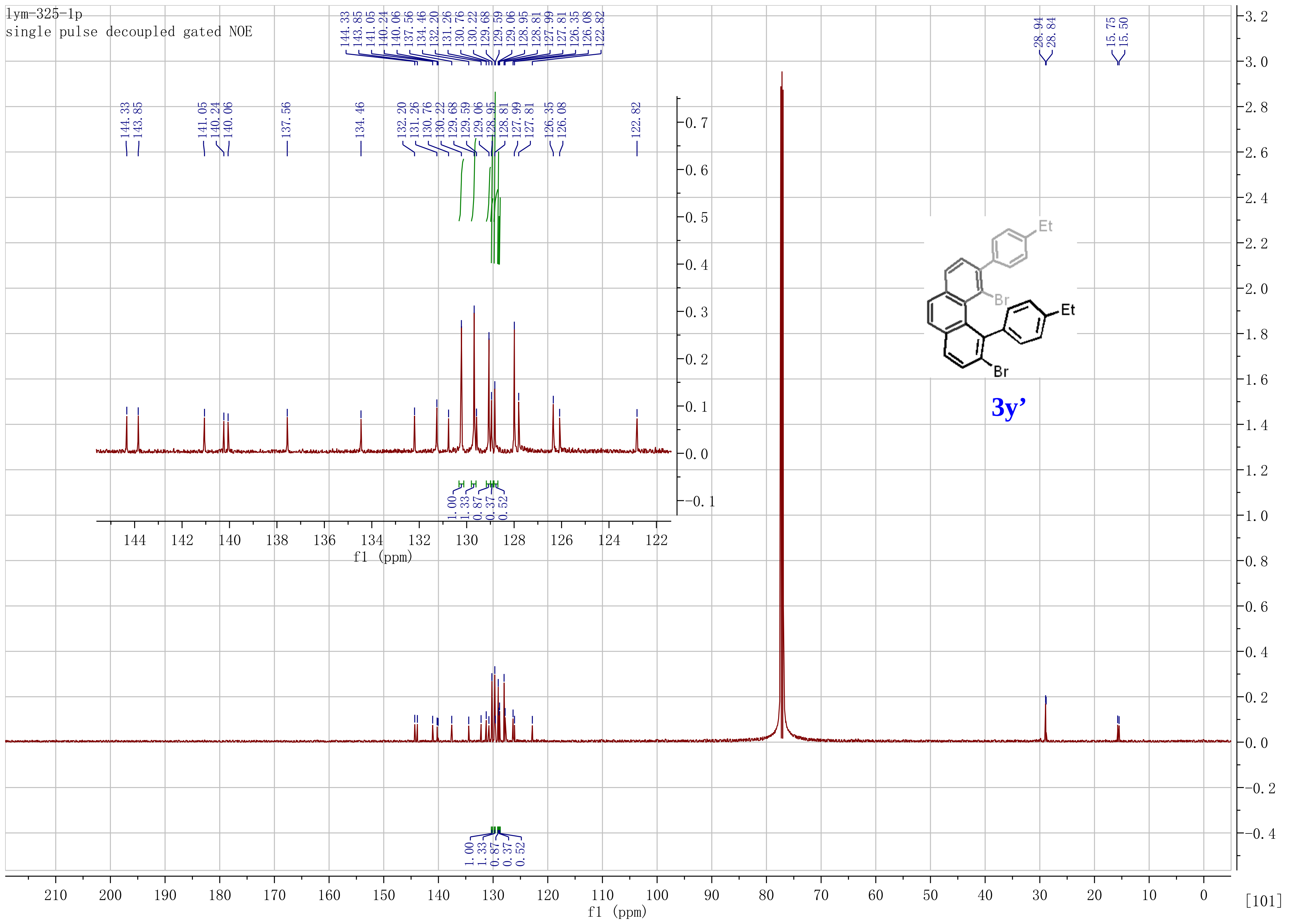
lym-325-1p
single pulse decoupled gated NOE
144.33
143.85
141.05
140.24
140.06
137.56
134.46
132.20
131.26
130.76
130.22
129.68
129.59
129.06
128.95
128.81
127.99
127.81
126.35
126.08
122.82
28.94
28.84
15.75
15.50
Et
Br
Et
Br
1.00
1.33
0.87
0.37
0.52
f1 (ppm)
f1 (ppm)

3y'

[101]

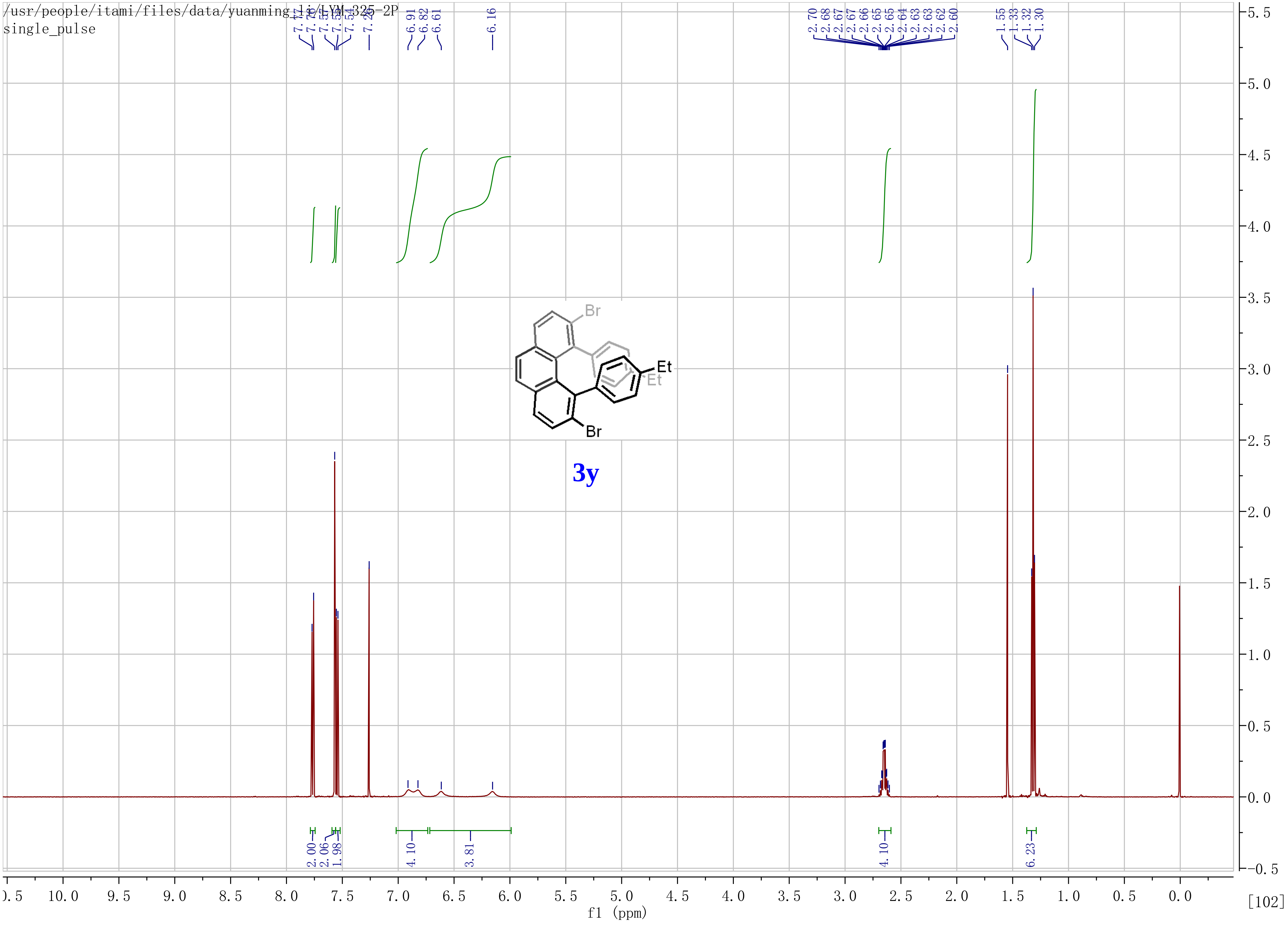
/usr/people/itami/files/data/yuanming
single_pulse
7.26
6.91
6.82
6.61
6.16
2.70
2.68
2.67
2.67
2.66
2.65
2.65
2.64
2.63
2.63
2.62
2.60
1.55
1.33
1.32
1.30
Br
Et
Et
Br
3y
2.00
2.06
1.98
4.10
3.81
4.10
6.23
f1 (ppm)

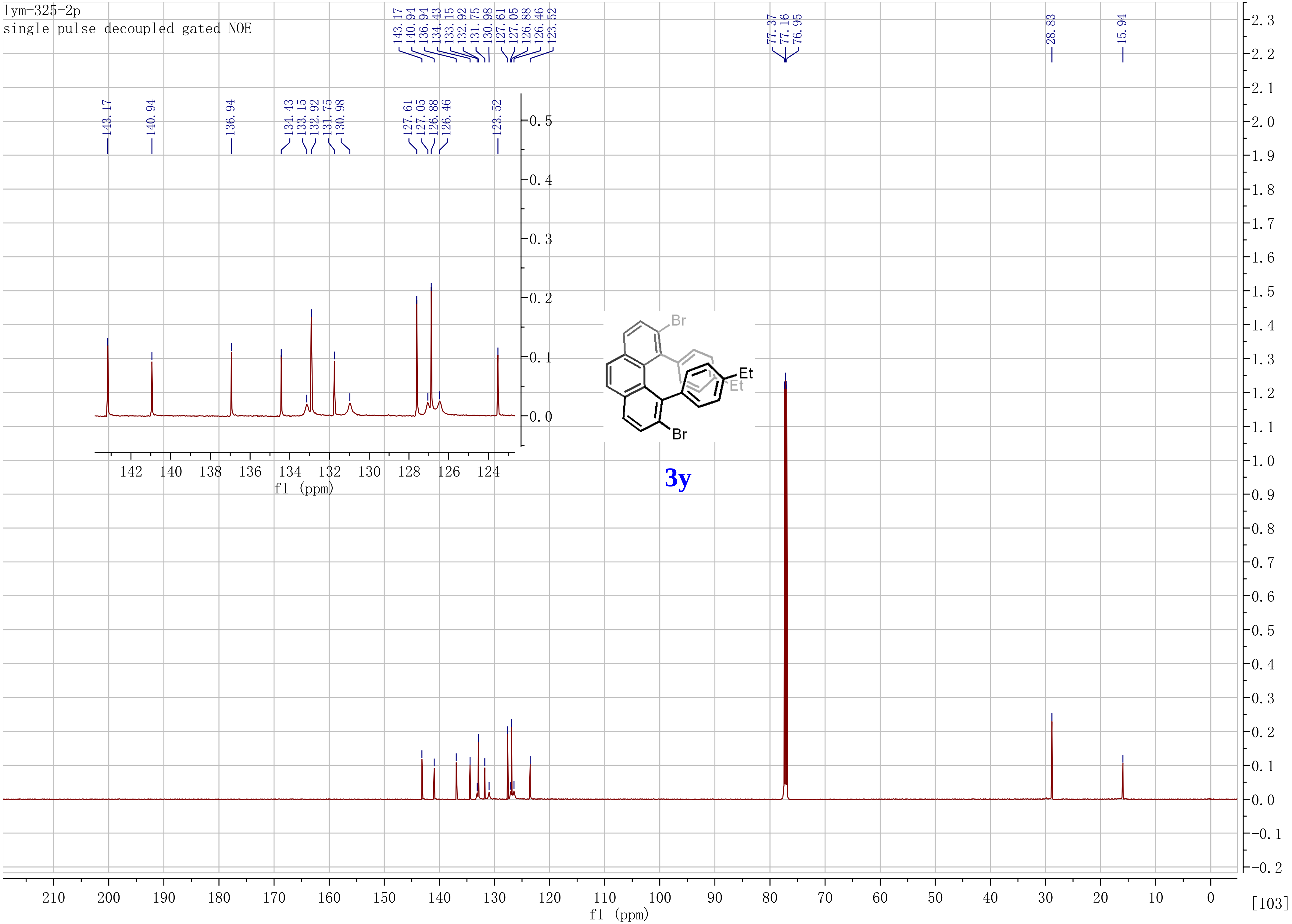

lym-325-2p
single pulse decoupled gated NOE
143.17
140.94
136.94
134.43
133.15
132.92
131.75
130.98
127.61
127.05
126.88
126.46
123.52
77.37
77.16
76.95
28.83
15.94
Br
Et
Et
Br
3y
f1 (ppm)

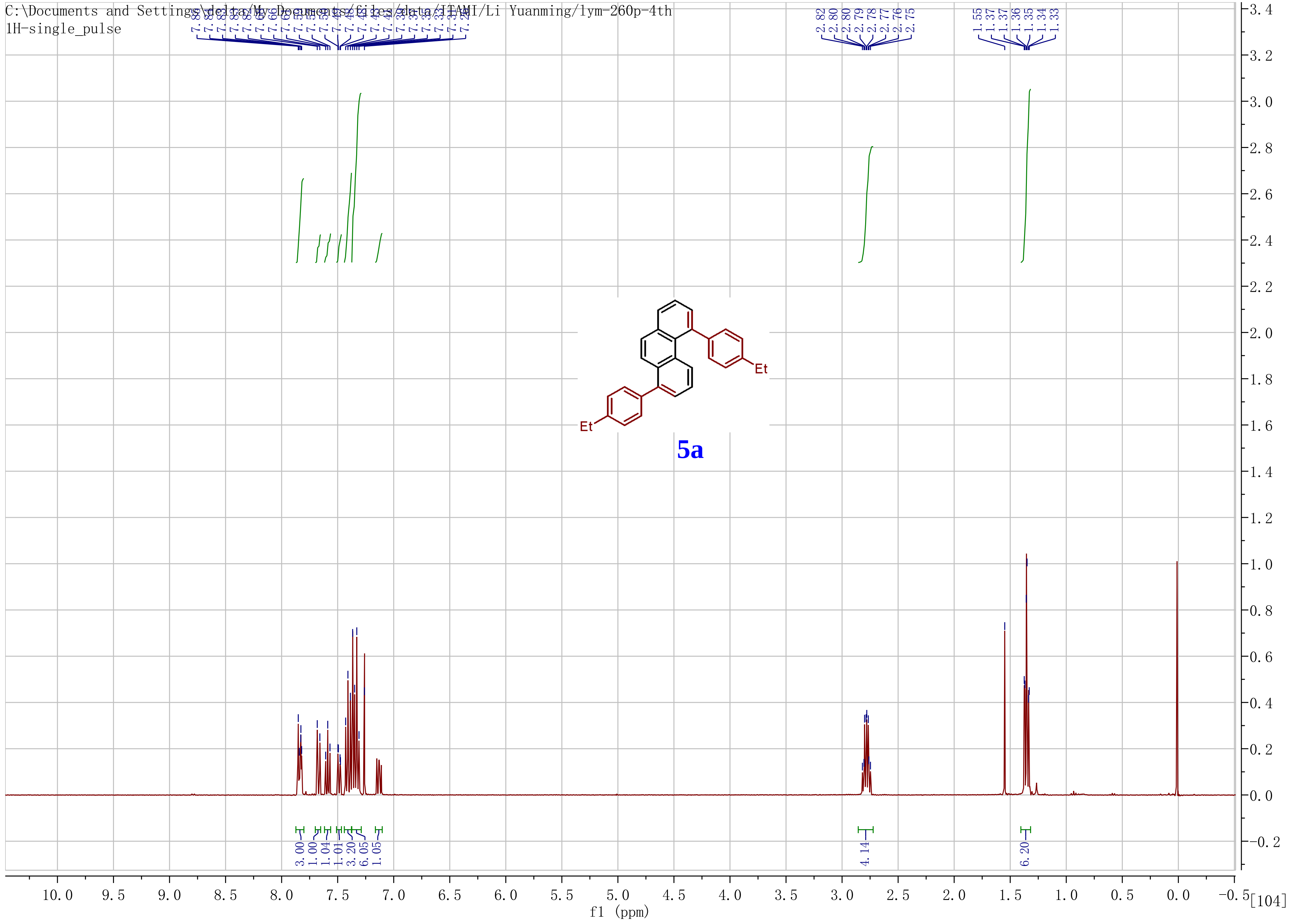

C:\Documents and Settings\delta\My Documents\files\data\ITAMI\Li Yuanming\lym-260p-4th
1H-single_pulse
2.82 2.80 2.80 2.79 2.78 2.77 2.76 2.75
1.55 1.37 1.37 1.36 1.35 1.34 1.33
Et
Et
5a
3.00 1.00 1.04 1.01 3.20 6.05 1.05
4.14
6.20
10.0 9.5 9.0 8.5 8.0 7.5 7.0 6.5 6.0 5.5 5.0 4.5 4.0 3.5 3.0 2.5 2.0 1.5 1.0 0.5 0.0 -0.5
f1 (ppm)
3.4 3.2 3.0 2.8 2.6 2.4 2.2 2.0 1.8 1.6 1.4 1.2 1.0 0.8 0.6 0.4 0.2 0.0 -0.2

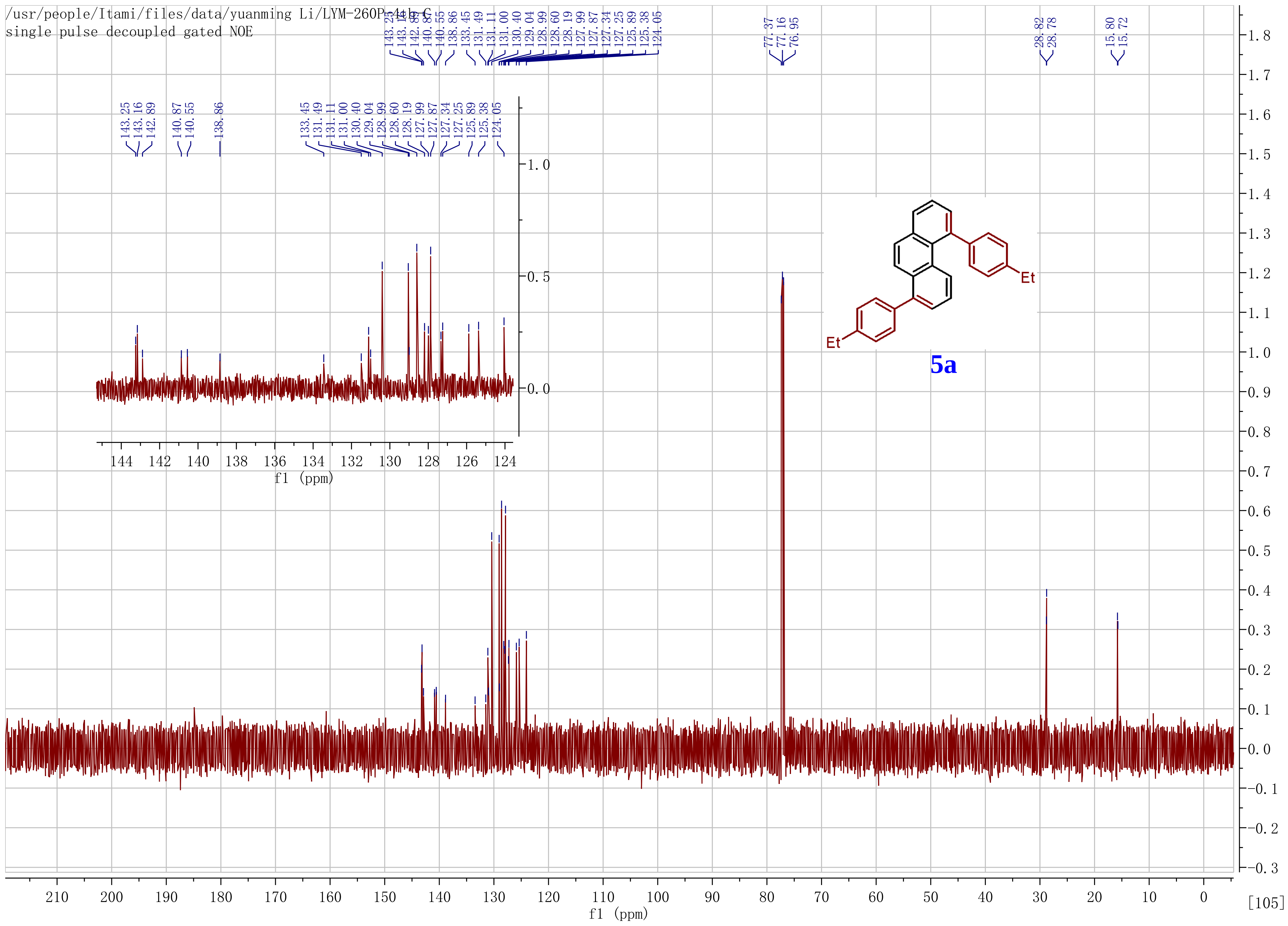
/usr/people/Itami/files/data/yuanming Li/LYM-260P-4th-C
single pulse decoupled gated NOE
77.37
77.16
76.95
28.82
28.78
15.80
15.72
143.25
143.16
142.89
140.87
140.55
138.86
133.45
131.49
131.11
131.00
130.40
129.04
128.99
128.60
128.19
127.99
127.87
127.34
127.25
125.89
125.38
124.05
Et
Et
5a
f1 (ppm)
f1 (ppm)

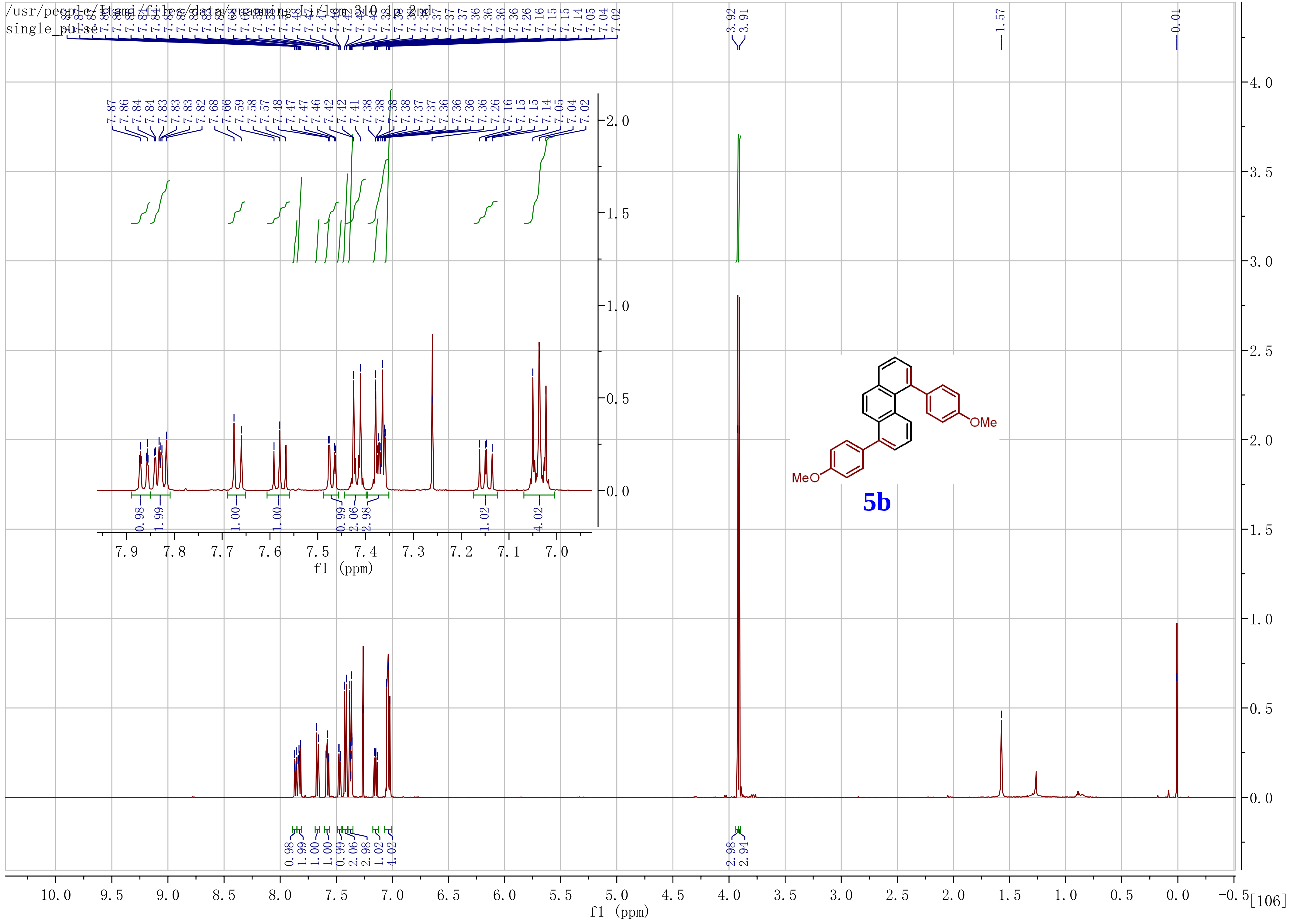
single_pulse
7.87 7.86 7.84 7.84 7.83 7.83 7.83 7.82 7.68 7.66 7.59 7.58 7.57 7.48 7.47 7.47 7.46 7.42 7.41 7.38 7.38 7.38 7.37 7.37 7.36 7.36 7.36 7.26 7.16 7.15 7.15 7.14 7.05 7.04 7.02
3.92 3.91
1.57
-0.01
0.98 1.99 1.00 1.00 0.99 2.06 2.98 1.02 4.02
2.98 2.94
MeO
OMe
5b
f1 (ppm)

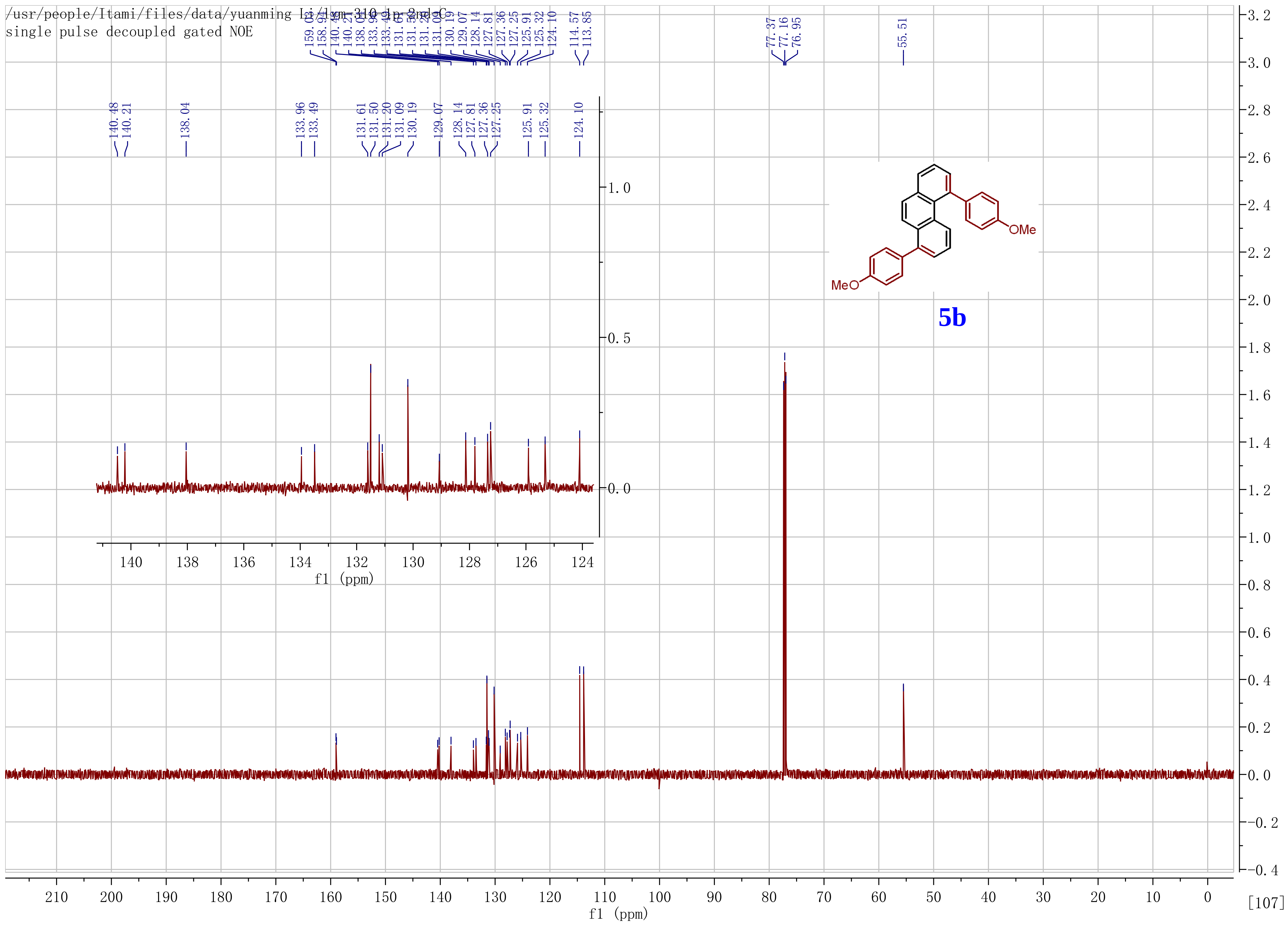

/usr/people/Itami/files/data/yuanming li/lym-310-1p-2nd-C
single pulse decoupled gated NOE
5b
MeO
OMe
f1 (ppm)
[107]

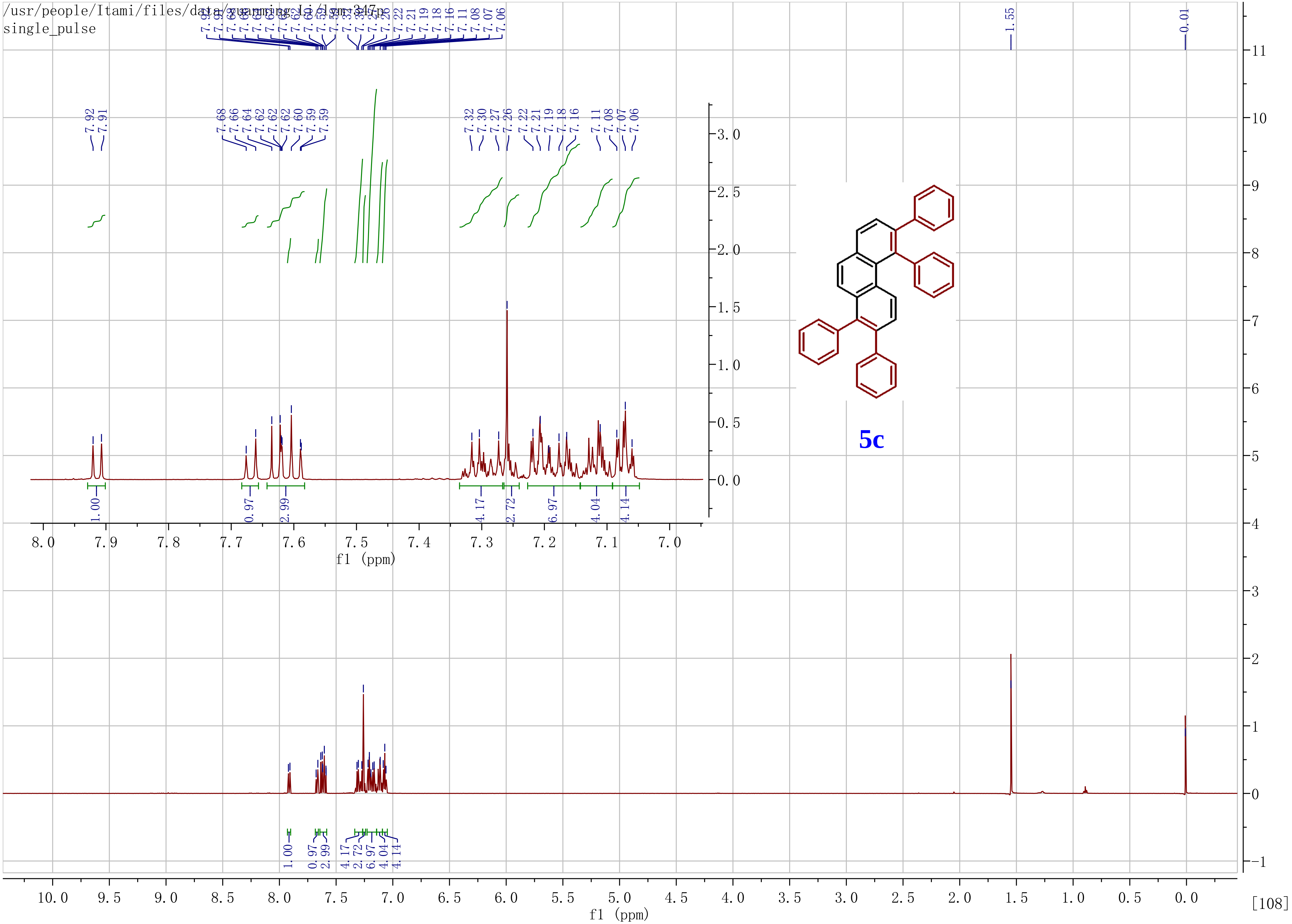
/usr/people/Itami/files/data/xueming-li/lym-347p
single_pulse
7.92
7.91
7.68
7.66
7.64
7.62
7.62
7.62
7.60
7.59
7.59
7.32
7.30
7.27
7.26
7.22
7.21
7.19
7.18
7.16
7.11
7.08
7.07
7.06
1.55
0.01
1.00
0.97
2.99
4.17
2.72
6.97
4.04
4.14
5c
f1 (ppm)

lym-335-2p
single pulse decoupled gated NOE

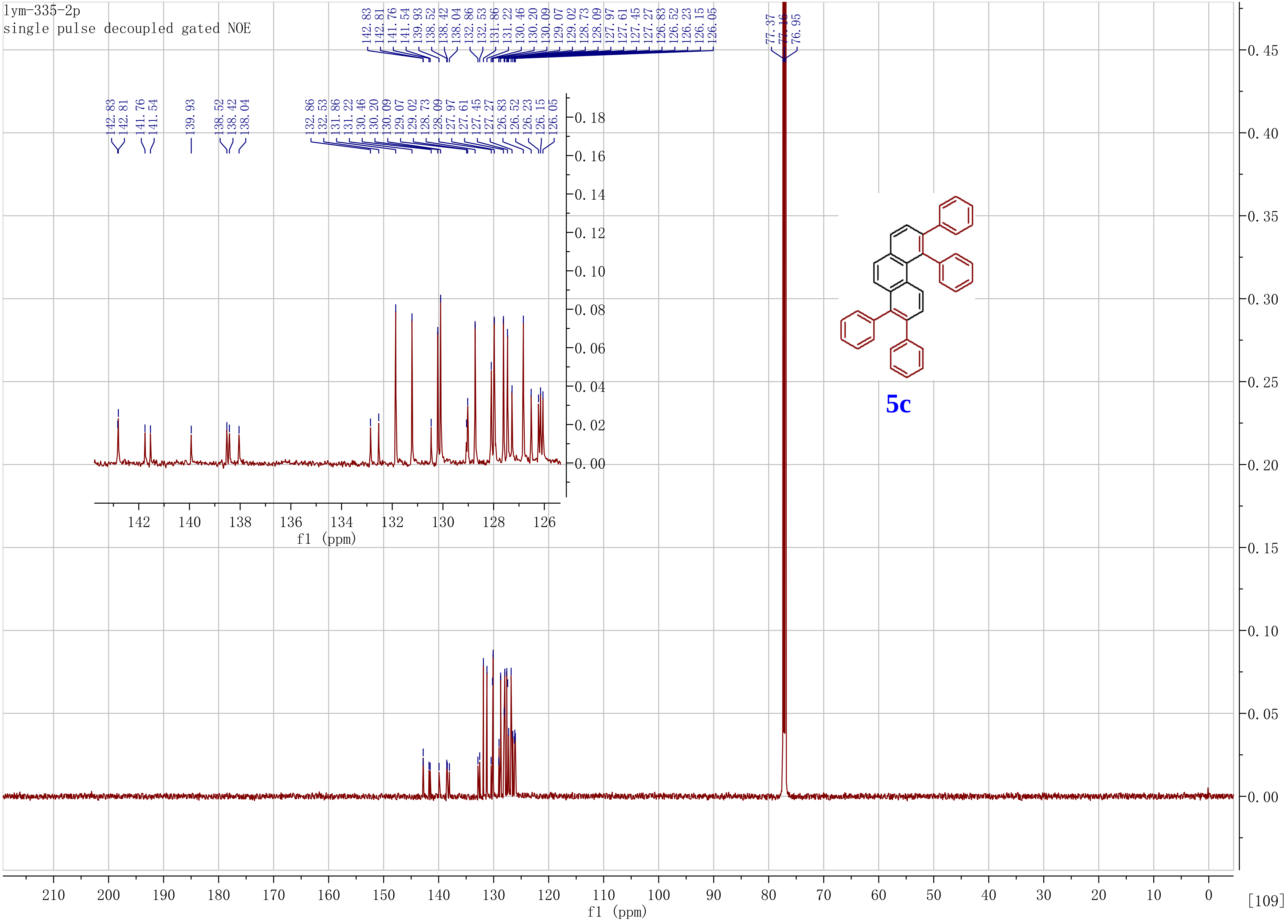

5c

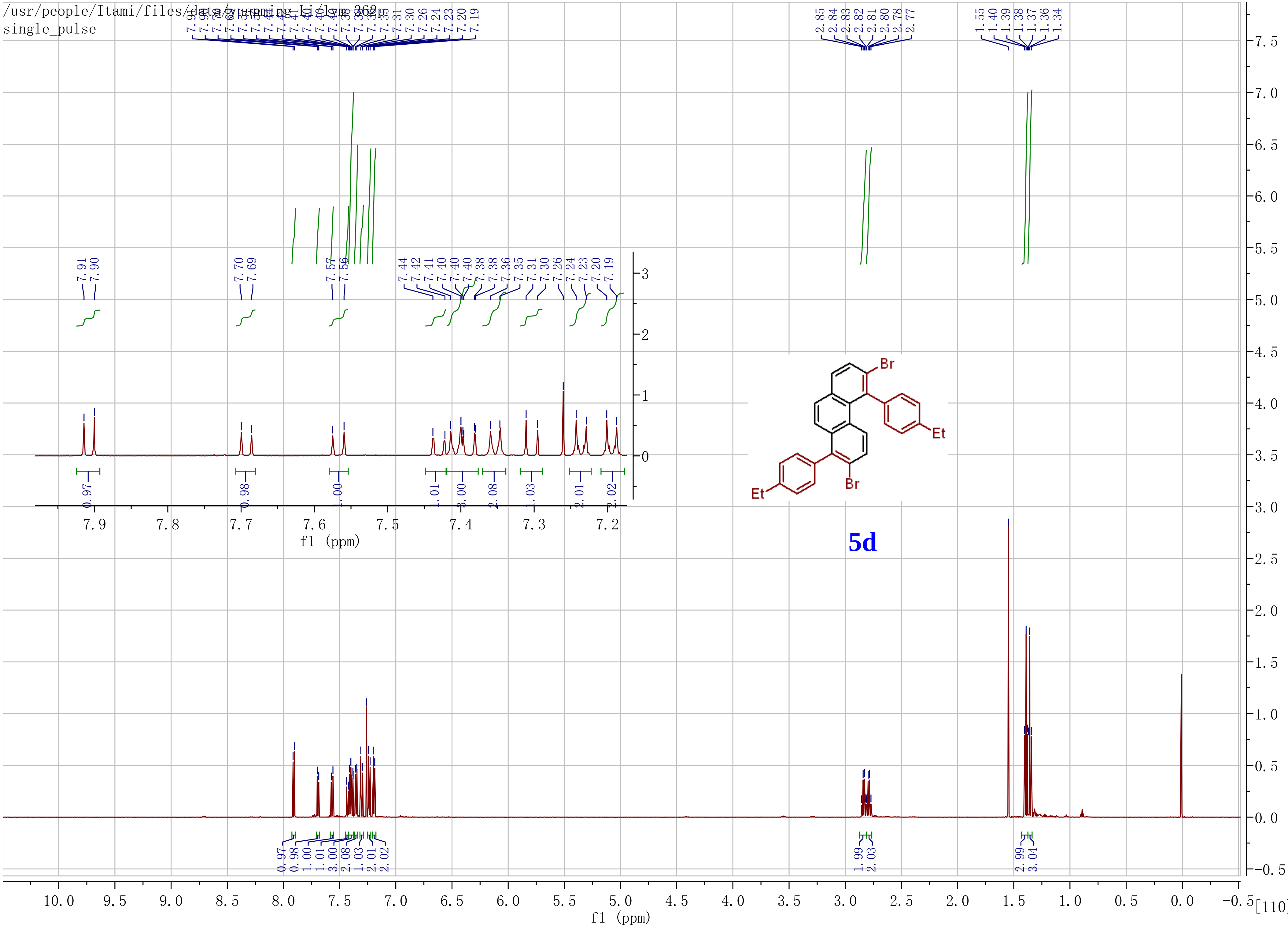
/usr/people/Itami/files/data/yueming_li/lym-362p
single_pulse
Br
Br
Et
Et
5d
f1 (ppm)
f1 (ppm)

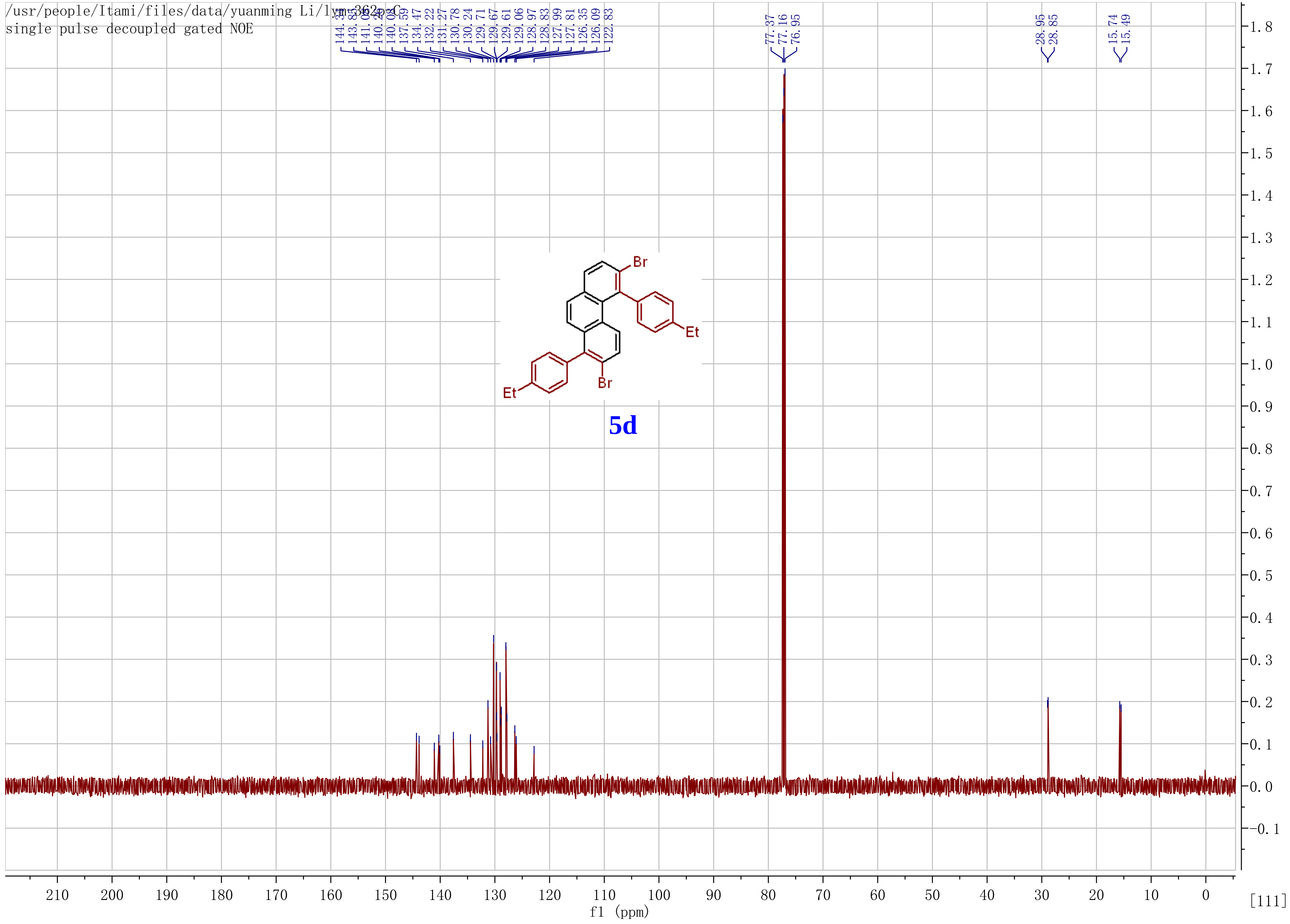
/usr/people/Itami/files/data/yuanming Li/lym-362p-C
single pulse decoupled gated NOE
140.08
137.59
134.47
132.22
131.27
130.78
130.24
129.71
129.67
129.61
129.06
128.97
128.83
127.99
127.81
126.35
126.09
122.83
77.37
77.16
76.95
28.95
28.85
15.74
15.49
Br
Et
Et
Br
5d
f1 (ppm)

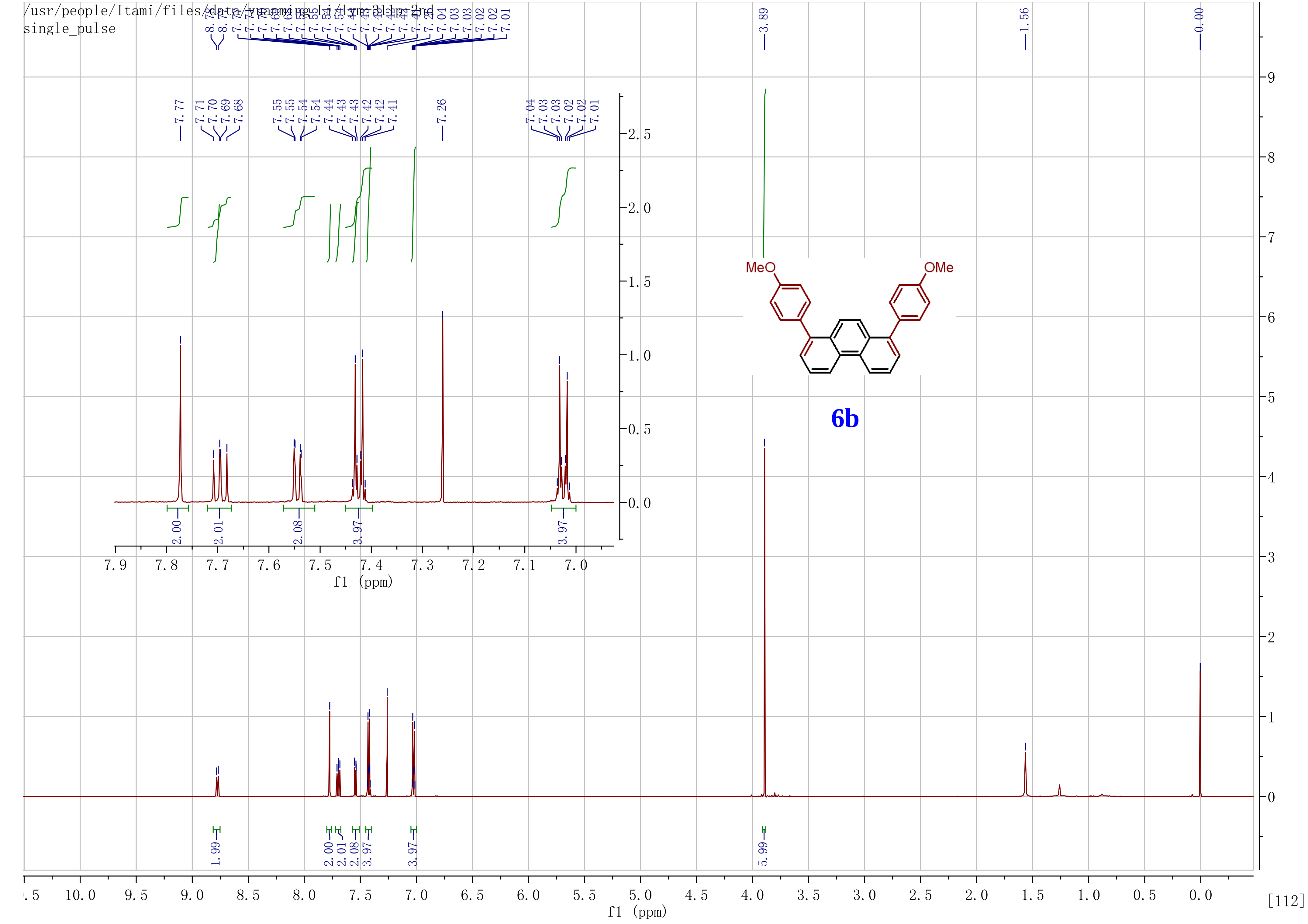
single_pulse
7.77
7.71
7.70
7.69
7.68
7.55
7.55
7.54
7.54
7.44
7.43
7.43
7.42
7.42
7.41
7.26
7.04
7.03
7.03
7.02
7.02
7.01
3.89
1.56
0.00
2.00
2.01
2.08
3.97
3.97
1.99
5.99
MeO
OMe
6b
f1 (ppm)

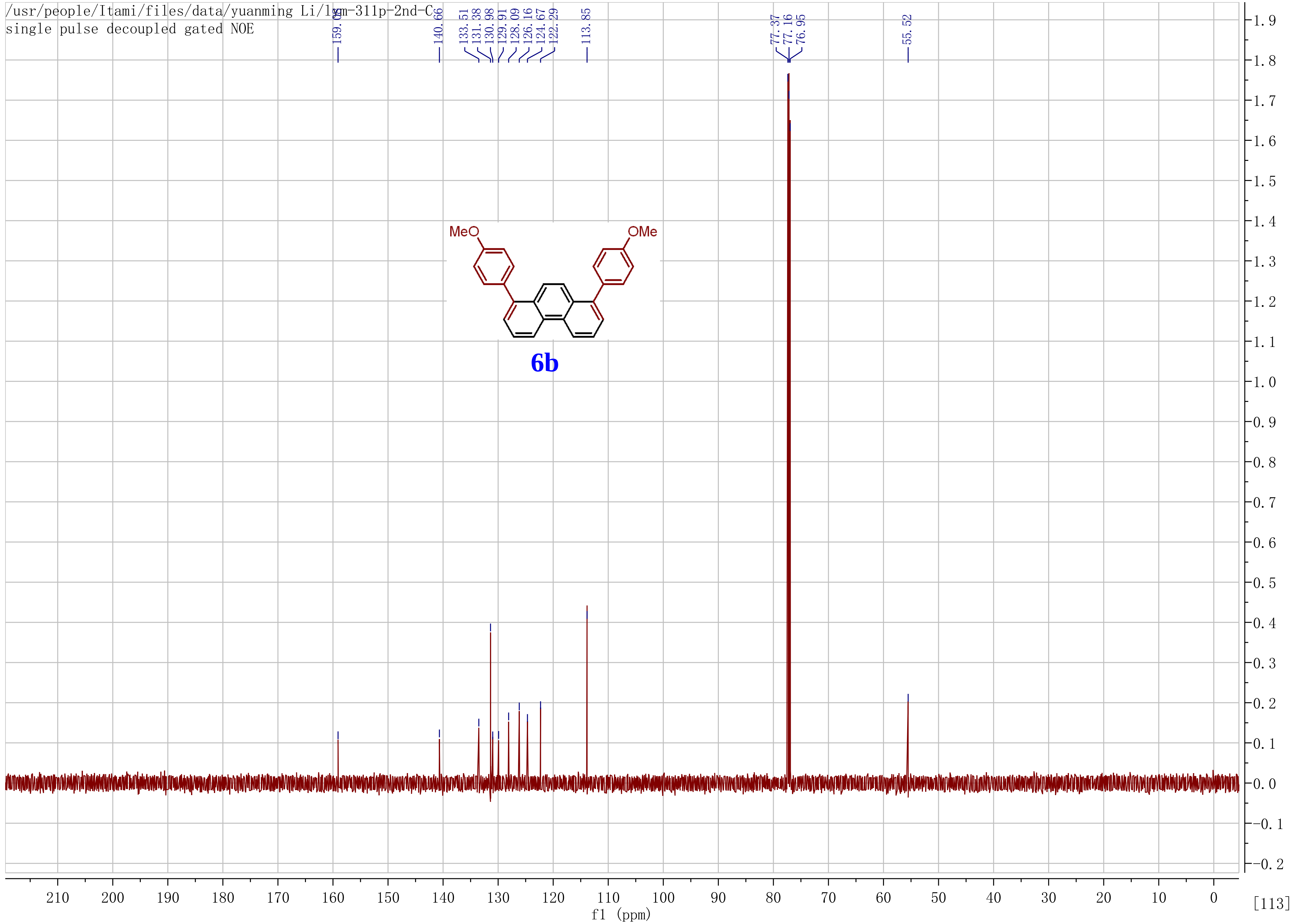
/usr/people/Itami/files/data/yuanming Li/lgm-311p-2nd-C
single pulse decoupled gated NOE
140.66
133.51
131.38
130.98
129.91
128.09
126.16
124.67
122.29
113.85
77.37
77.16
76.95
55.52
MeO
OMe
6b
f1 (ppm)

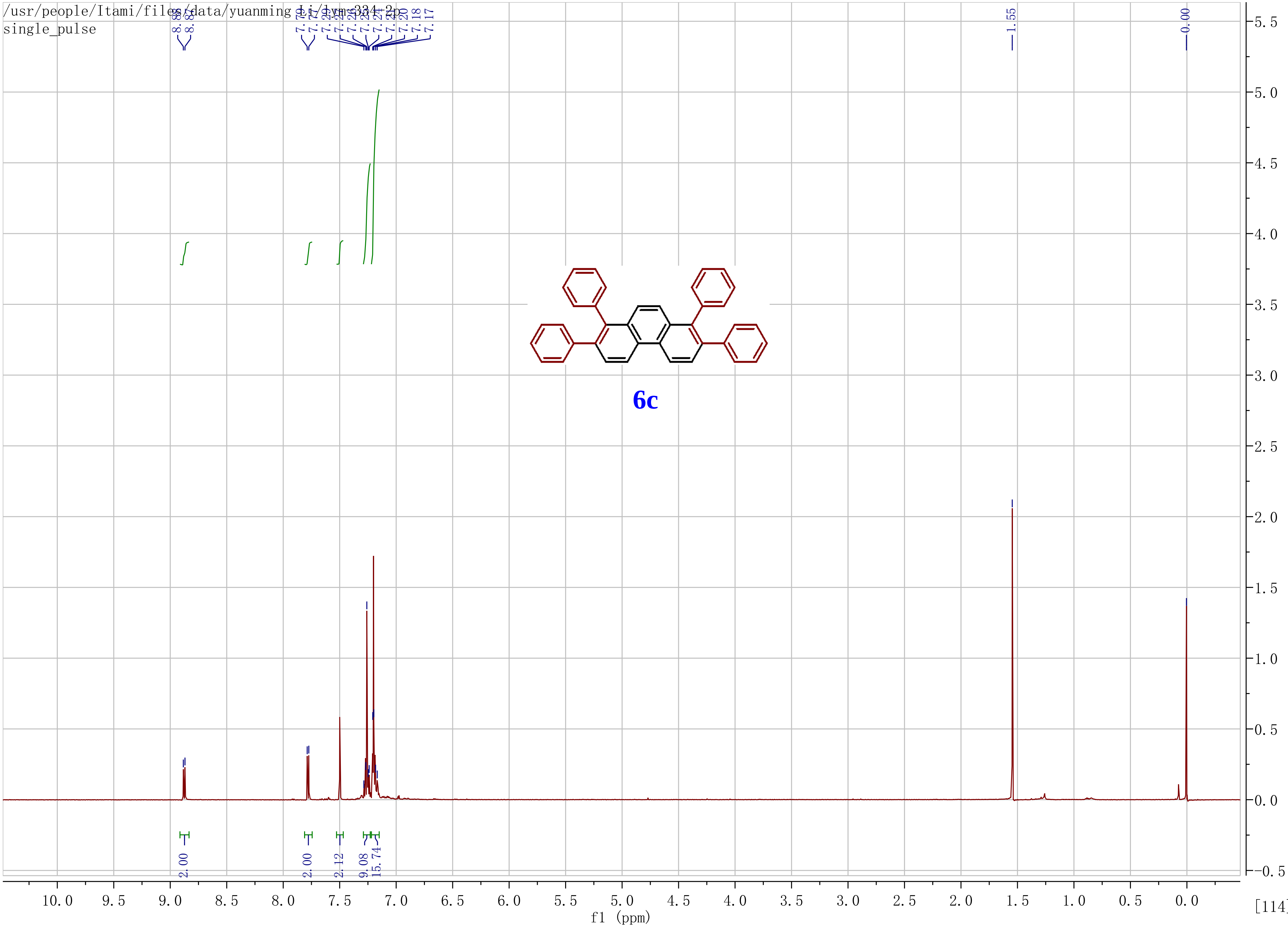

/usr/people/Itami/files/data/yuanming li/lym-334-2p
single_pulse
7.18
7.17
1.55
0.00
6c
2.00
2.00
2.12
9.08
15.74
f1 (ppm)

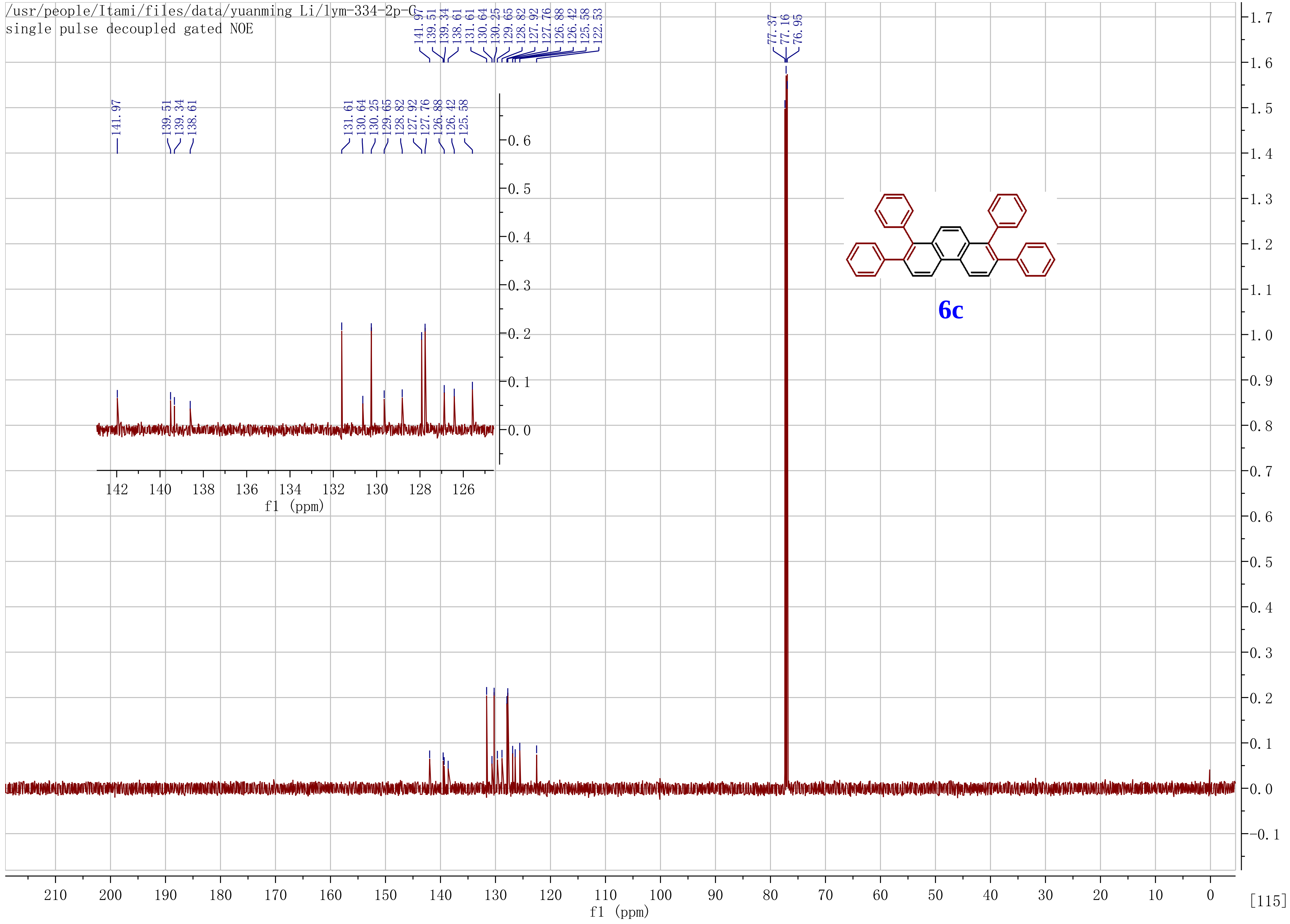
/usr/people/Itami/files/data/yuanming Li/lym-334-2p-C
single pulse decoupled gated NOE
141.97
139.51
139.34
138.61
131.61
130.64
130.25
129.65
128.82
127.92
127.76
126.88
126.42
125.58
122.53
77.37
77.16
76.95
6c
f1 (ppm)

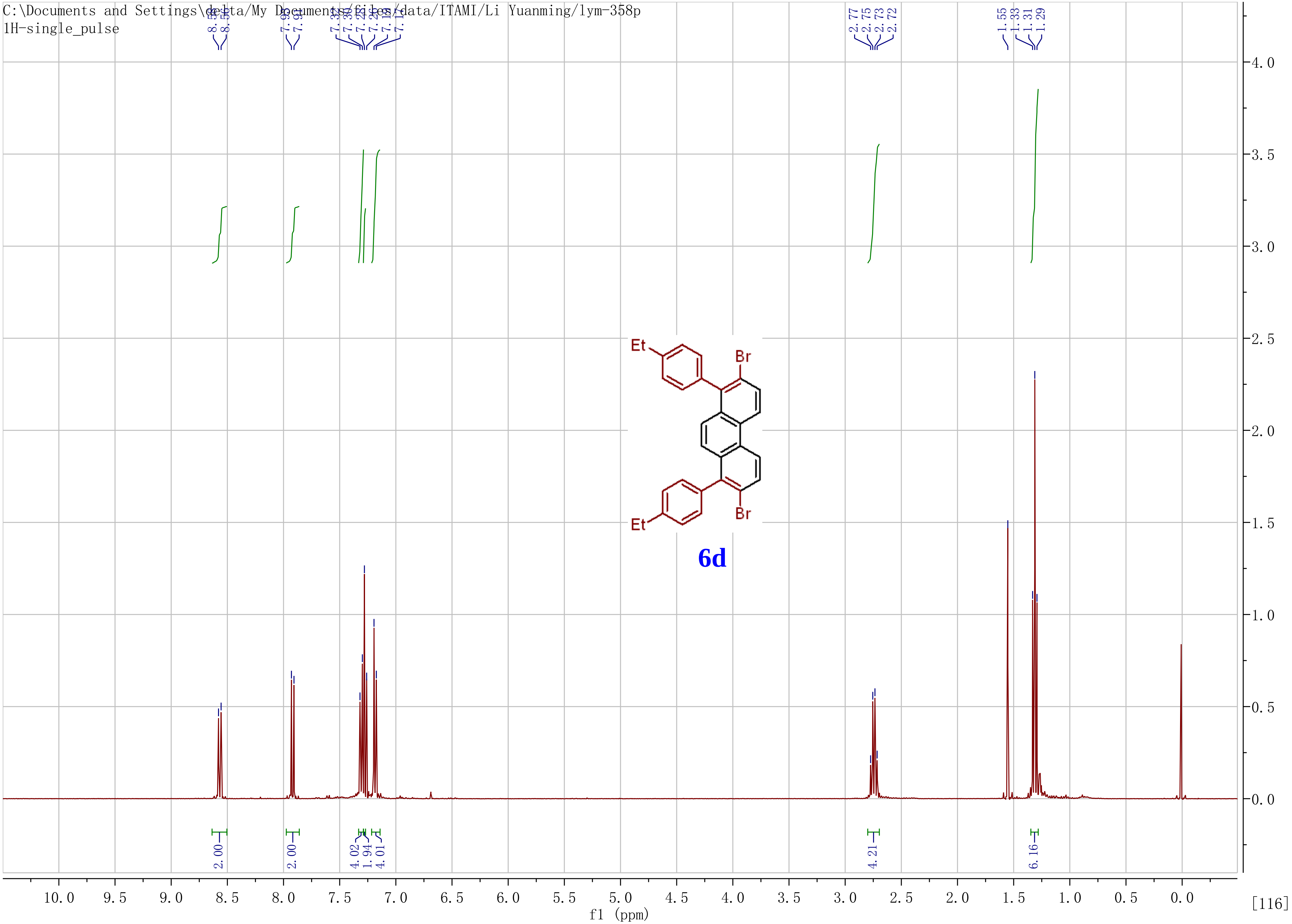

C:\Documents and Settings\delta/My Documents/files/data/ITAMI/Li Yuanming/lym-358p
1H-single_pulse
2.77
2.75
2.73
2.72
1.55
1.33
1.31
1.29
Et
Br
Et
Br
6d
2.00
2.00
4.02
1.94
4.01
4.21
6.16
f1 (ppm)

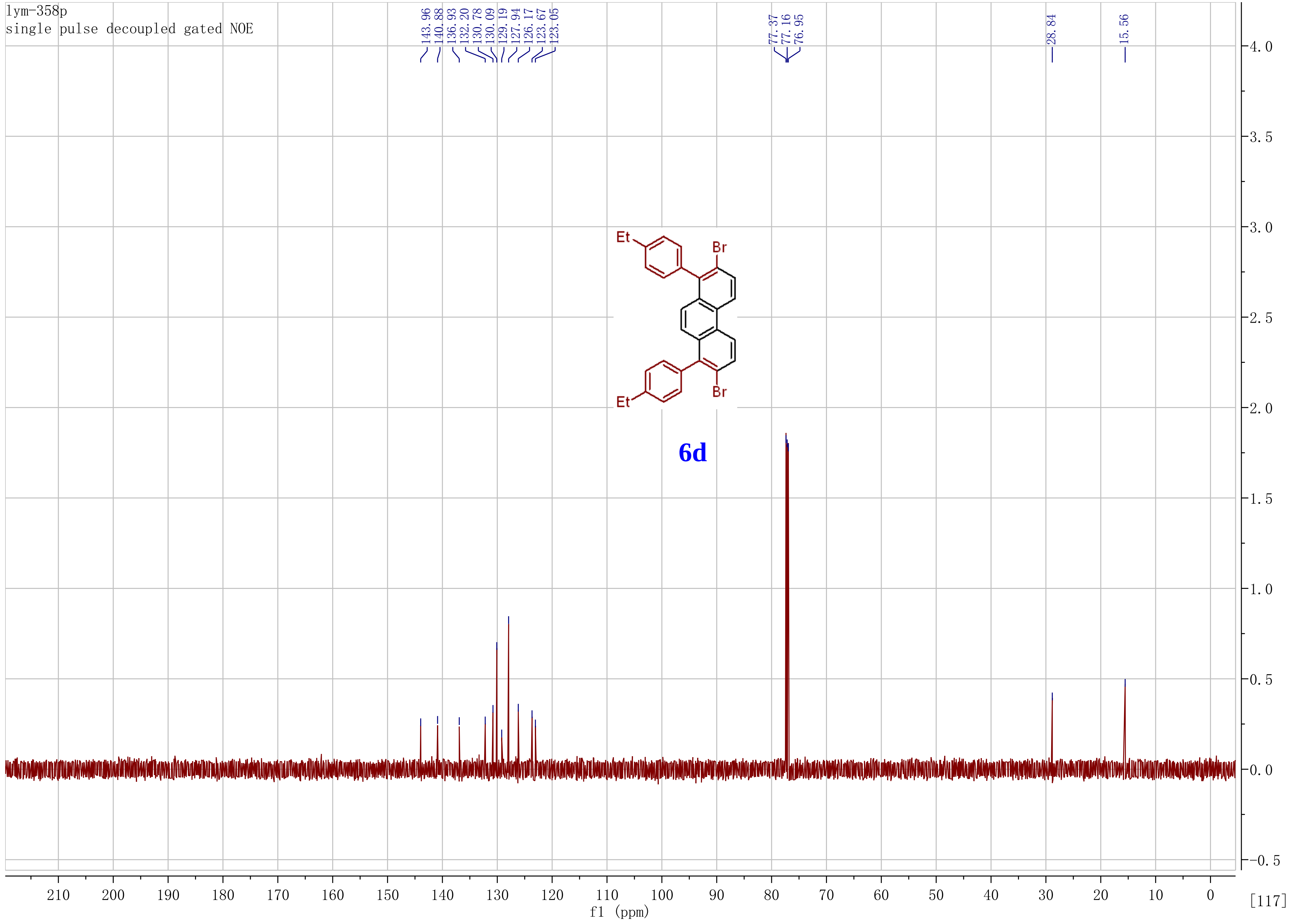

lym-358p
single pulse decoupled gated NOE
143.96
140.88
136.93
132.20
130.78
130.09
129.19
127.94
126.17
123.67
123.05
77.37
77.16
76.95
28.84
15.56
Et
Br
Et
Br
6d
f1 (ppm)

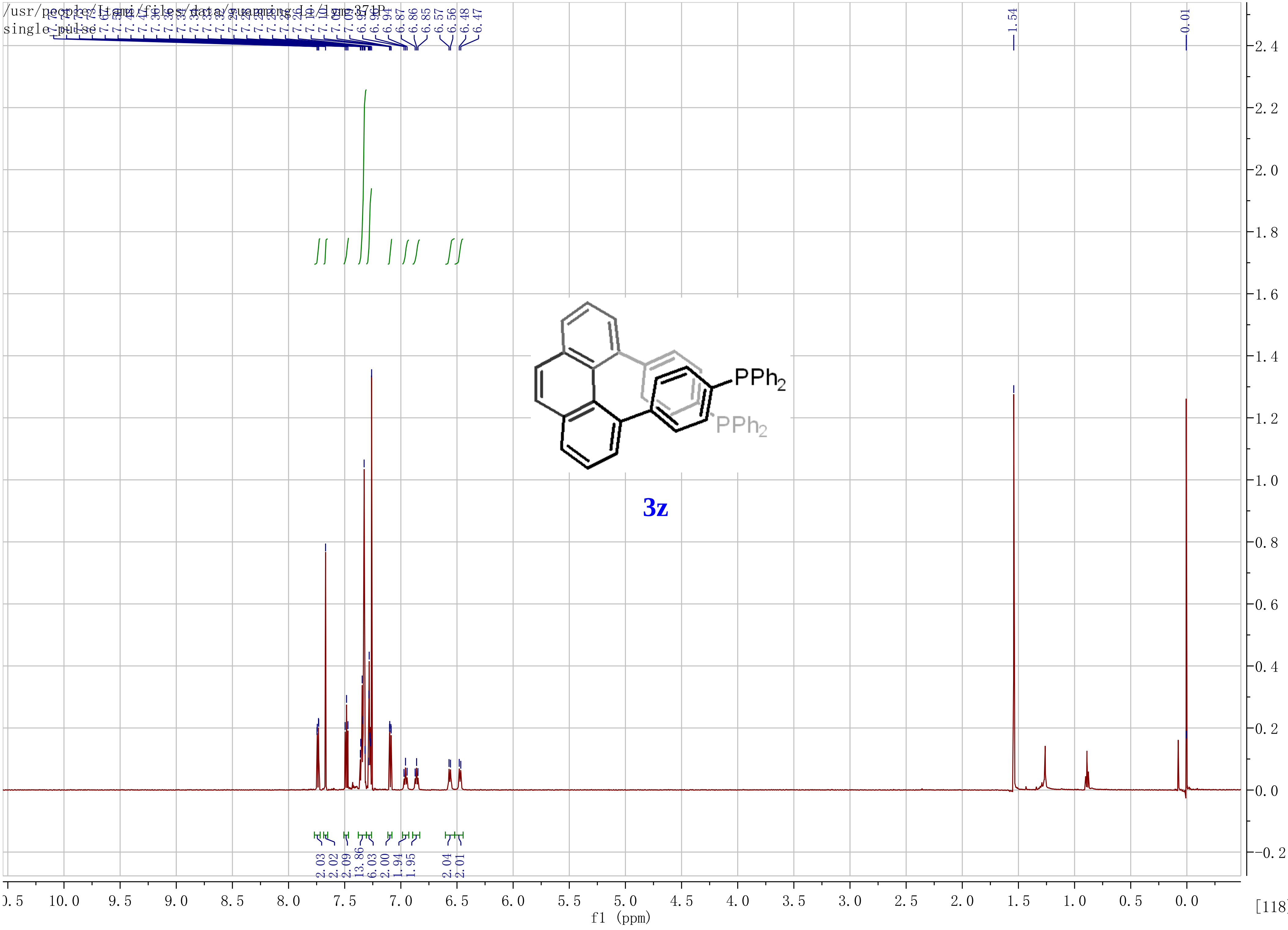

single_pulse
1.54
0.01
PPh2
PPh2
3z
2.03
2.02
2.09
13.86
6.03
2.00
1.94
1.95
2.04
2.01
f1 (ppm)

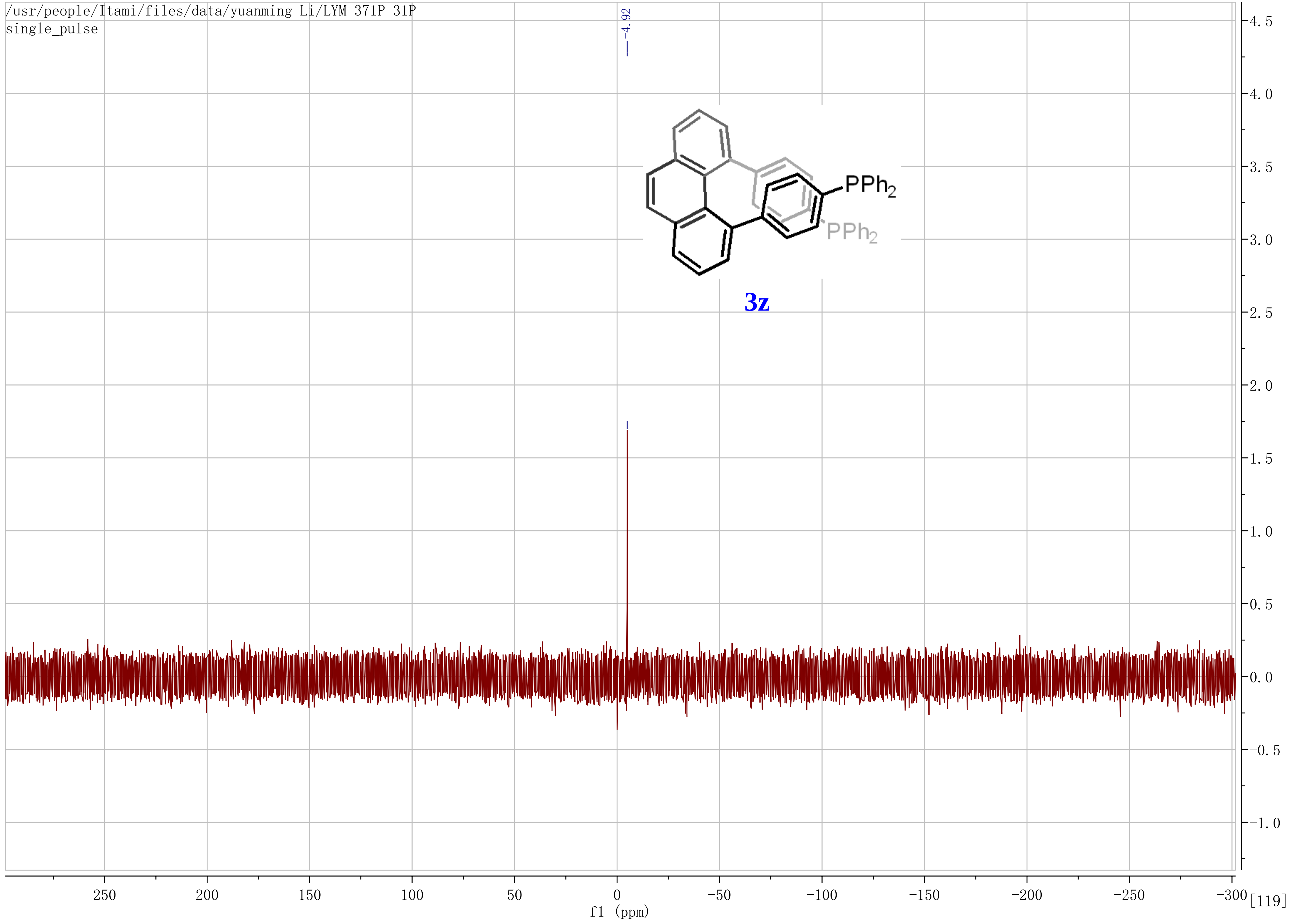
/usr/people/Itami/files/data/yuanming Li/LYM-371P-31P
single_pulse
-4.92
PPh2
PPh2
250 200 150 100 50 0 -50 -100 -150 -200 -250 -300
f1 (ppm)
4.5 4.0 3.5 3.0 2.5 2.0 1.5 1.0 0.5 0.0 -0.5 -1.0

3z

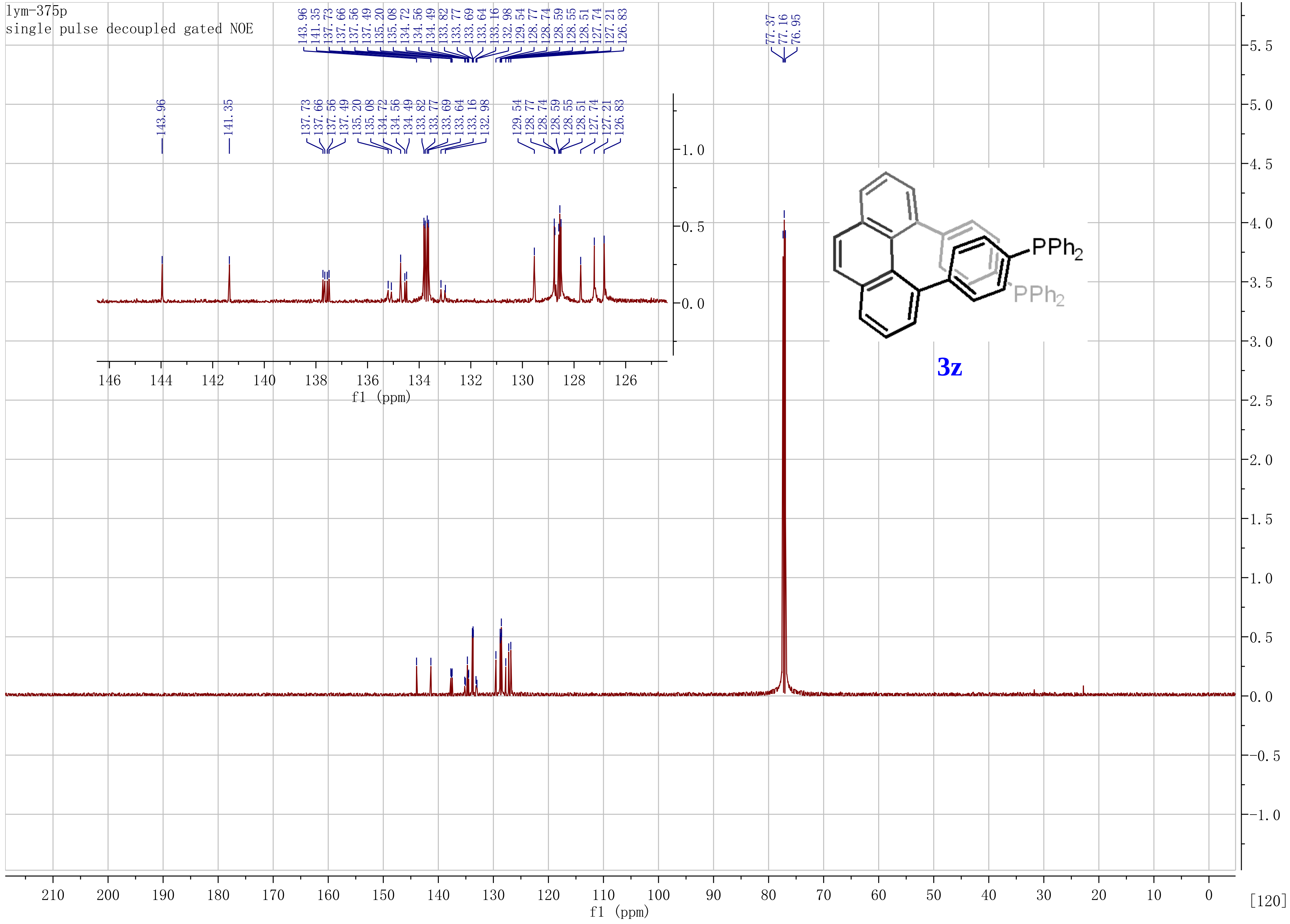
lym-375p
single pulse decoupled gated NOE
143.96
141.35
137.73
137.66
137.56
137.49
135.20
135.08
134.72
134.56
134.49
133.82
133.77
133.69
133.64
133.16
132.98
129.54
128.77
128.74
128.59
128.55
128.51
127.74
127.21
126.83
77.37
77.16
76.95
PPh2
PPh2
3z
f1 (ppm)
f1 (ppm)

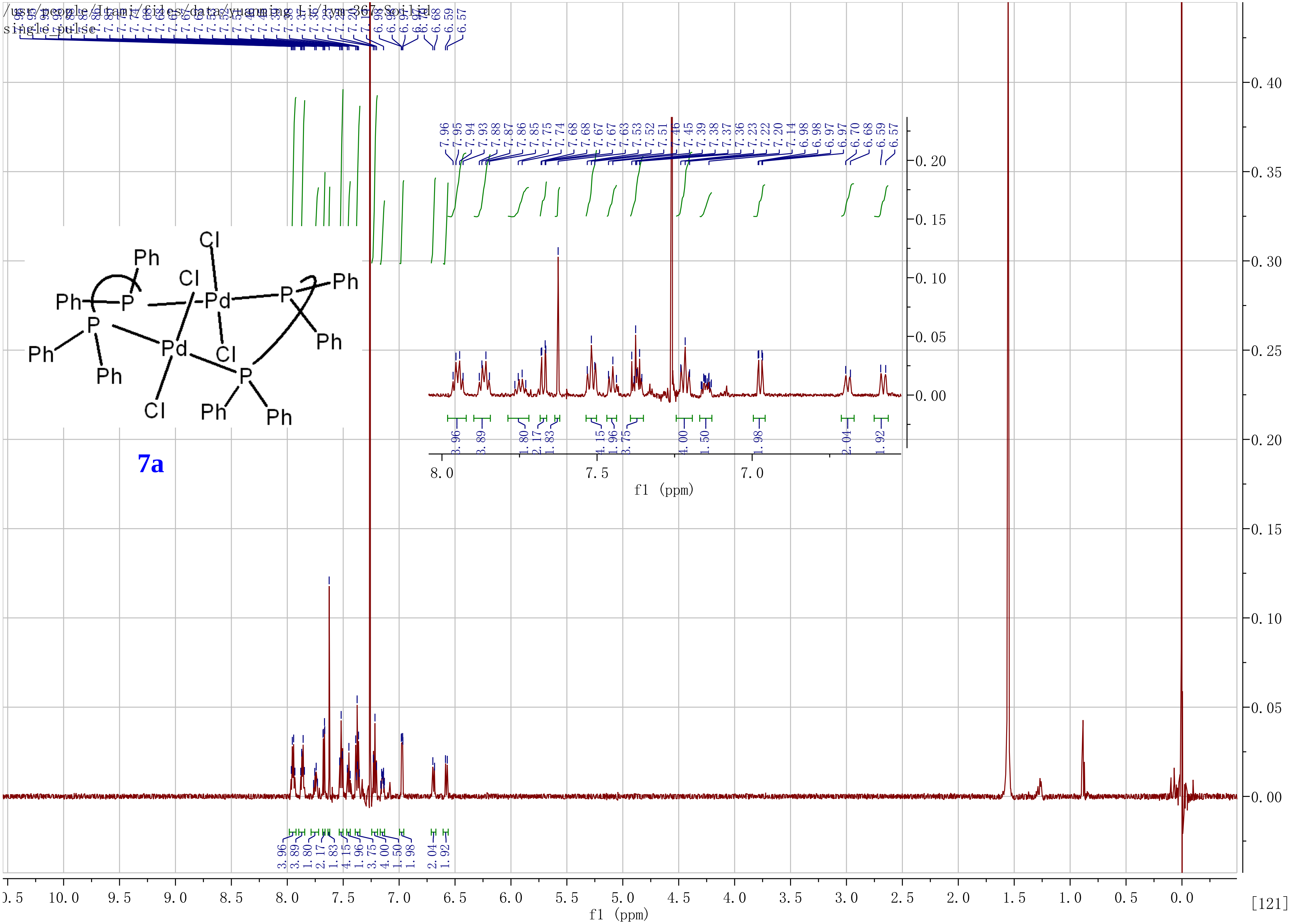
single-pulse
Cl
Ph
Cl
Pd
P
Ph
Ph
P
P
Ph
Pd
Cl
Ph
Ph
P
Ph
Cl
Ph
Ph
7a
7.96 7.95 7.94 7.93 7.88 7.87 7.86 7.85 7.75 7.74 7.68 7.68 7.67 7.67 7.63 7.53 7.52 7.51 7.46 7.45 7.39 7.38 7.37 7.36 7.23 7.22 7.20 7.14 6.98 6.98 6.97 6.97 6.70 6.68 6.59 6.57
3.96 3.89 1.80 2.17 1.83 4.15 1.96 3.75 4.00 1.50 1.98 2.04 1.92
f1 (ppm)

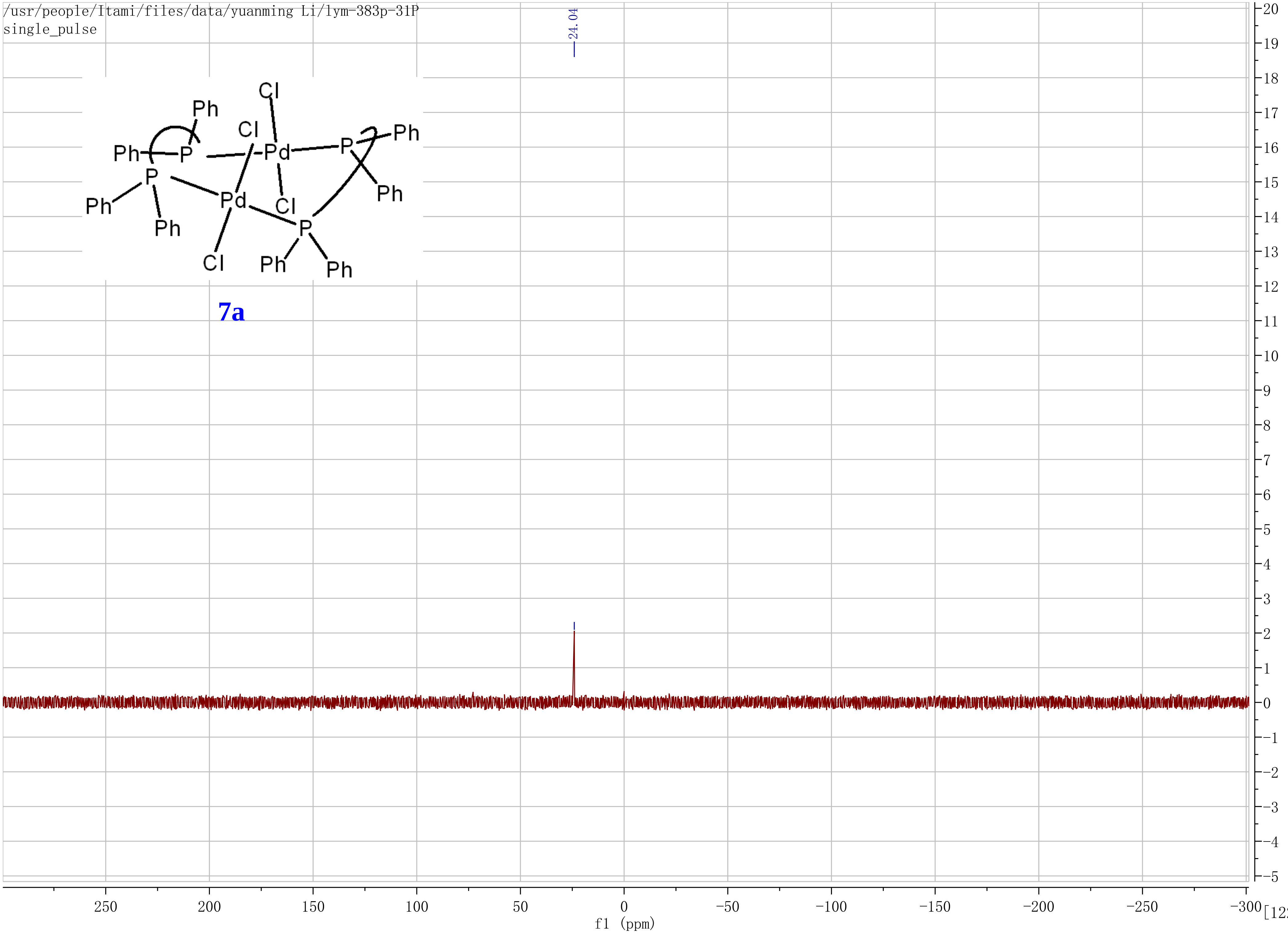
/usr/people/Itami/files/data/yuanming Li/lym-383p-31P
single_pulse
24.04
Cl
Ph
Cl
Ph
Ph
P
Pd
P
P
Pd
Cl
Ph
Ph
Ph
Cl
P
Ph
Ph
7a
250 200 150 100 50 0 −50 −100 −150 −200 −250 −300
f1 (ppm)
20 19 18 17 16 15 14 13 12 11 10 9 8 7 6 5 4 3 2 1 0 −1 −2 −3 −4 −5

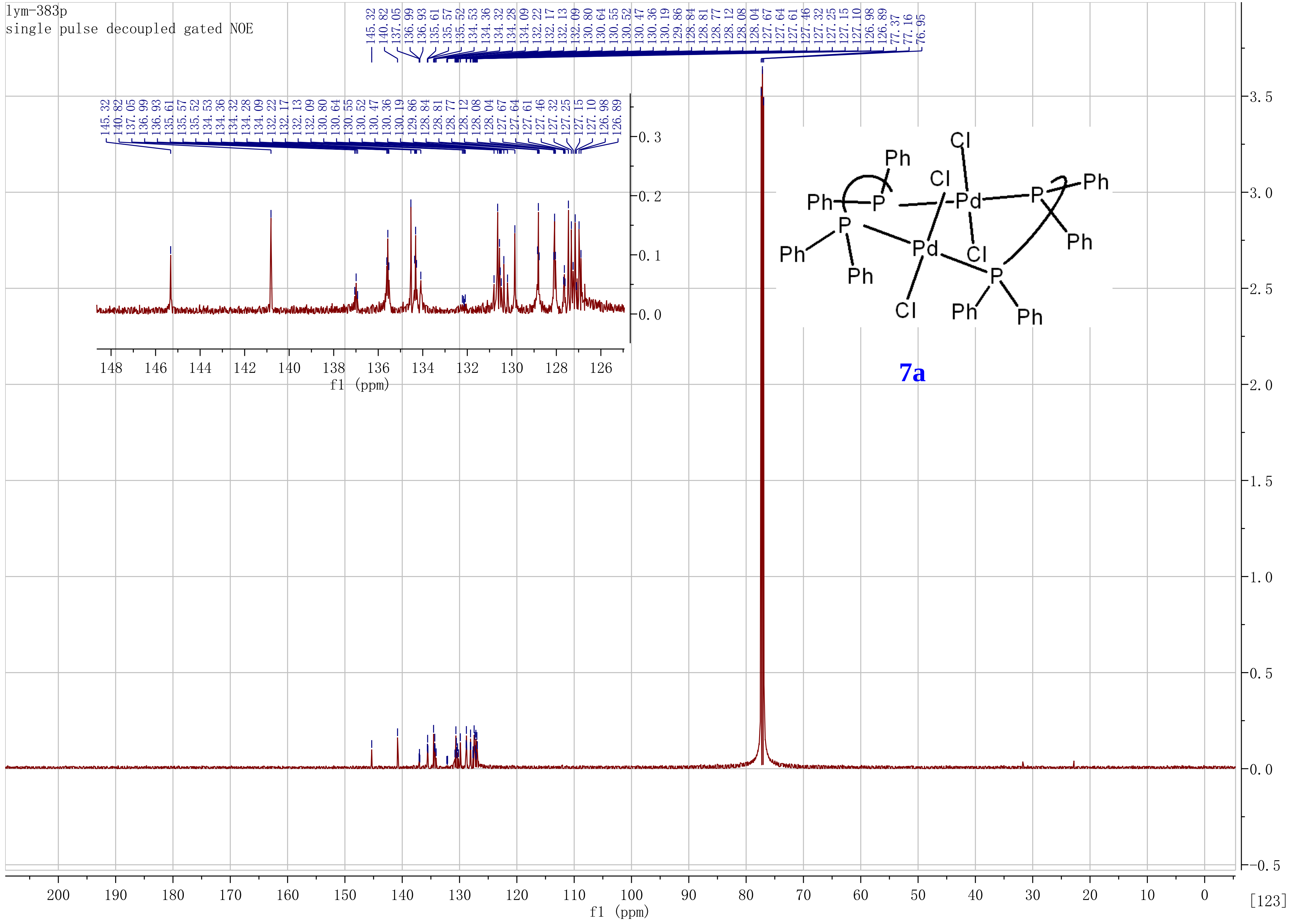
lym-383p
single pulse decoupled gated NOE
145.32 140.82 137.05 136.99 136.93 135.61 135.57 135.52 134.53 134.36 134.32 134.28 134.09 132.22 132.17 132.13 132.09 130.80 130.64 130.55 130.52 130.47 130.36 130.19 129.86 128.84 128.81 128.77 128.12 128.08 128.04 127.67 127.64 127.61 127.46 127.32 127.25 127.15 127.10 126.98 126.89 77.37 77.16 76.95
f1 (ppm)
Cl
Ph
Cl
Ph
P
Pd
P
Ph
P
Ph
Pd
Cl
Ph
Ph
P
Cl
Ph
Ph
7a
f1 (ppm)
